# Multiple Model-Informed Open-Loop Control of Uncertain Intracellular Signaling Dynamics

**DOI:** 10.1371/journal.pcbi.1003546

**Published:** 2014-04-10

**Authors:** Jeffrey P. Perley, Judith Mikolajczak, Marietta L. Harrison, Gregery T. Buzzard, Ann E. Rundell

**Affiliations:** 1 Weldon School of Biomedical Engineering, Purdue University, West Lafayette, Indiana, United States of America; 2 Department of Medicinal Chemistry & Molecular Pharmacology, Purdue University, West Lafayette, Indiana, United States of America; 3 Department of Mathematics, Purdue University, West Lafayette, Indiana, United States of America; University of Minnesota, United States of America

## Abstract

Computational approaches to tune the activation of intracellular signal transduction pathways both predictably and selectively will enable researchers to explore and interrogate cell biology with unprecedented precision. Techniques to control complex nonlinear systems typically involve the application of control theory to a descriptive mathematical model. For cellular processes, however, measurement assays tend to be too time consuming for real-time feedback control and models offer rough approximations of the biological reality, thus limiting their utility when considered in isolation. We overcome these problems by combining nonlinear model predictive control with a novel adaptive weighting algorithm that blends predictions from multiple models to derive a compromise open-loop control sequence. The proposed strategy uses weight maps to inform the controller of the tendency for models to differ in their ability to accurately reproduce the system dynamics under different experimental perturbations (i.e. control inputs). These maps, which characterize the changing model likelihoods over the admissible control input space, are constructed using preexisting experimental data and used to produce a model-based open-loop control framework. In effect, the proposed method designs a sequence of control inputs that force the signaling dynamics along a predefined temporal response without measurement feedback while mitigating the effects of model uncertainty. We demonstrate this technique on the well-known Erk/MAPK signaling pathway in T cells. *In silico* assessment demonstrates that this approach successfully reduces target tracking error by 52% or better when compared with single model-based controllers and non-adaptive multiple model-based controllers. *In vitro* implementation of the proposed approach in Jurkat cells confirms a 63% reduction in tracking error when compared with the best of the single-model controllers. This study provides an experimentally-corroborated control methodology that utilizes the knowledge encoded within multiple mathematical models of intracellular signaling to design control inputs that effectively direct cell behavior in open-loop.

## Author Summary

Most cell behavior arises as a response to external forces. Signals from the extracellular environment are passed to the cell's nucleus through a complex network of interacting proteins. Perturbing these pathways can change the strength or outcome of the signals, which could be used to treat or prevent a pathological response. While manipulating these networks can be achieved using a variety of methods, the ability to do so predictably over time would provide an unprecedented level of control over cell behavior and could lead to new therapeutic design and research tools in medicine and systems biology. Hence, we propose a practical computational framework to aid in the design of experimental perturbations to force cell signaling dynamics to follow a predefined response. Our approach represents a novel merger of model-based control and information theory to blend the predictions from multiple mathematical models into a meaningful compromise solution. We verify through simulation and experimentation that this solution produces excellent agreement between the cell readouts and several predefined trajectories, even in the presence of significant modeling uncertainty and without measurement feedback. By combining elements of information and control theory, our approach will help advance the best practices in model-based control applications for medicine.

## Introduction

The ability to predictably manipulate intracellular signaling pathways would provide an unprecedented level of control of cellular processes and could potentially generate new approaches for therapeutic design and research tools in medicine and systems biology. Intracellular signaling networks are complex assemblies of interconnected molecular components that relay information and coordinate responses to environmental cues. For example, T lymphocytes are critical regulators of the immune response against the threats of invading pathogens and cancerous host cells. Their response to external stimuli is coordinated through several mediators including extracellular signal-regulated kinases (Erk), which are particularly noteworthy as they have been implicated in a number of autoimmune diseases and cancers [1]–[4]. Phenotypic change due to extracellular perturbation is a robust property of normal cell behavior and involves considerable feedback and crosstalk and is highly nonlinear. To help resolve the uncertainty and understand the complexity inherent within these signaling pathways, many researchers have developed mathematical models of signaling processes [5]–[10]. These models can be used to inform control strategies that try to predictably manipulate the intracellular signaling response, but also give rise to a new set of challenges in systems biology and control engineering.

To date, the majority of model-based control of cellular processes and systems has focused on biomass production in bioreactors [11], [12] or were largely theoretical. Within the past decade, research has started to evaluate engineered control strategies for single and multiple cell signaling processes within experiments. Noble and Rundell [13] used closed-loop (i.e. *in silico* feedback) control to direct HL60 cell differentiation through periodic boluses of a differentiation-inducing agent determined by nonlinear model predictive control (MPC). In 2012, they revised the initial approach to improve the transient response of the differentiating cells over 20 days by using a multi-scenario adaptive model predictive control [14]. Uhlendorf *et al.* [15] applied open-loop (i.e. non-feedback) and closed-loop control to provide long-term regulation of gene expression on the single-cell and population level by manipulating the osmotic stress on cells in a microfluidic environment. The open-loop approach failed to regulate mean fluorescence to the desired set points in the laboratory. On the other hand, attempts using measurement feedback proved to be more successful at coping with modeling inaccuracies and inherent intracellular fluctuations. Opto-genetics and synthetic biology provide effective methods for control theory to interface with cellular processes at the genetic and signal transduction level. Milias-Argeitis *et al.* [16] attempted to control the activation of a light-responsive Phy/PIF module that altered the expression of a yellow florescent protein (YFP) activated through the Gal1 promoter in *Saccharomyces*. As in [15], the closed-loop approach proved more successful in driving YFP intensity to the desired set points in the laboratory. Tettcher *et al.* [17], [18] also used light as the control input to modulate the localization of PIF-tagged proteins to the cell membrane. This has the ability to alter intracellular signaling through coordination of the localization. Both of these studies achieve long-term regulation of cell activities through transcriptional control. Another closed-loop control study was proposed in Menolascina *et al.* [19] using pulse width modulation to specify the duration of pulses of galactose administered via microfluidics. Their experimental results confirm the predictive capabilities of their model for the synthetic gene network in *S. Cerevisiae*.

All of the aforementioned studies rely upon computer-based feedback control: a computer in the loop uses system measurements to inform control decisions. While closed-loop control is widely understood to be more robust to disturbance and uncertainty, in many cases the rapid dynamics, scale and complexity of intracellular signaling events and absence of real-time measurement assays prohibit its use. In these cases, any control strategy must design the control inputs in advance without measurements to inform future steps and rely solely upon prior information gleaned from existing experimental data. However, this may introduce unwanted degradation in control performance due to discrepancies between the actual system and the prediction model (i.e. plant-model mismatch). Methods to systematically and optimally combine this prior information with the predictive capacity of multiple mathematical models are needed.

Because mathematical models are abstractions of biological reality, they may differ in dominant species, network structure, parameter values, and functional representation. For most signaling pathways, limited preexisting quantitative data and qualitative observations are insufficient to discriminate unambiguously between the mathematical models. When applying control theory techniques, the experimental perturbations (i.e. control inputs) predicted to elicit a desired behavior from a system may be different for each model. Selecting the “best” of these models is an important challenge to control theorists for systems biology applications [20]. Apgar *et al.* [21] applied control theory to discriminate among mechanism-based chemical kinetic models of epidermal growth factor receptor signaling. The method designs dynamic stimuli to delineate the system's response to subtle differences in the network topology. The model associated with the best controller is deemed the best representative of the original system. However, in using this model alone, we would implicitly assume that the model is also the most accurate in alternate operating regions, which may or may not be the case. Without performing the experiments there is no way to know *a priori* which model is best; furthermore, the best model may change depending upon the experiment planned. How to optimally combine information from these network models to design control inputs that, when applied to the cell, force the signaling dynamics along a desired path is the subject of much debate.

Growing attention in systems biology has been given to control methodologies considering multiple prediction models. Multiple models, or scenarios, have been previously used to improve robustness to parametric uncertainty in closed-loop model-based control [14], [22]–[25]. The approach proposed by Rao *et al.* [24] computed control inputs by weighting multiple step-response models using a Bayesian algorithm to control hemodynamic variables in hypertensive subjects. These concepts were extended for disturbance rejection in a van de Vusse reactor using Bayesian methods to produce a weighted-average linear prediction model [25]. The recursive weighting system was effective at eliminating the “hard switch” between controllers. Noble *et al.* [14] employed adaptive nonlinear MPC based on multiple data-consistent parameter characterizations to manipulate cell differentiation experimentally. Control inputs were chosen such that the average tracking error and resource efficiency were optimized, which resulted in superior controller performance over single-model MPC. However, this approach assumes all parameter scenarios are equally likely and does not consider that their accuracy in predicting actual system dynamics often varies between distinct regions of the state and control input spaces. Furthermore, the aforementioned approaches consider essentially single model structures with little to no variation in the mathematical equation structures. While methods considering model uncertainty explicitly are numerous, it is generally in the form of disturbances and process noise with very little consideration given to qualitatively distinct biological hypotheses. This is a critical flaw as these hypotheses could translate into qualitatively distinct equation structures and input/output and state/output relationships. Finally, all of these approaches employ real-time feedback that is not available for most intracellular signaling pathways because the dynamics are often too rapid for standard measurement assays.

In this paper, we present a practical open-loop control framework with a novel method for employing existing experimental data to confidently combine multiple model predictions to form effective control inputs. That is, an automated control input selection process is developed for the open-loop case; this process is advised by information regarding model accuracy in regions of the input space where potential control inputs are likely to be present. Akaike weights, based on an information-theoretic metric penalizing model complexity and lack-of-fitness used for model discrimination [26], [27], are employed for this purpose. In the results, we successfully demonstrate the algorithm with several simulated test cases and corroborate a subset of these with *in vitro* experiments in Jurkat T lymphocytes. Conclusions and future work are presented in the discussion and detailed and illustrated descriptions of the algorithm and experimental protocols are provided in the materials and methods.

## Results

### Controlled System: T Cell Activation

**Figure 1:**
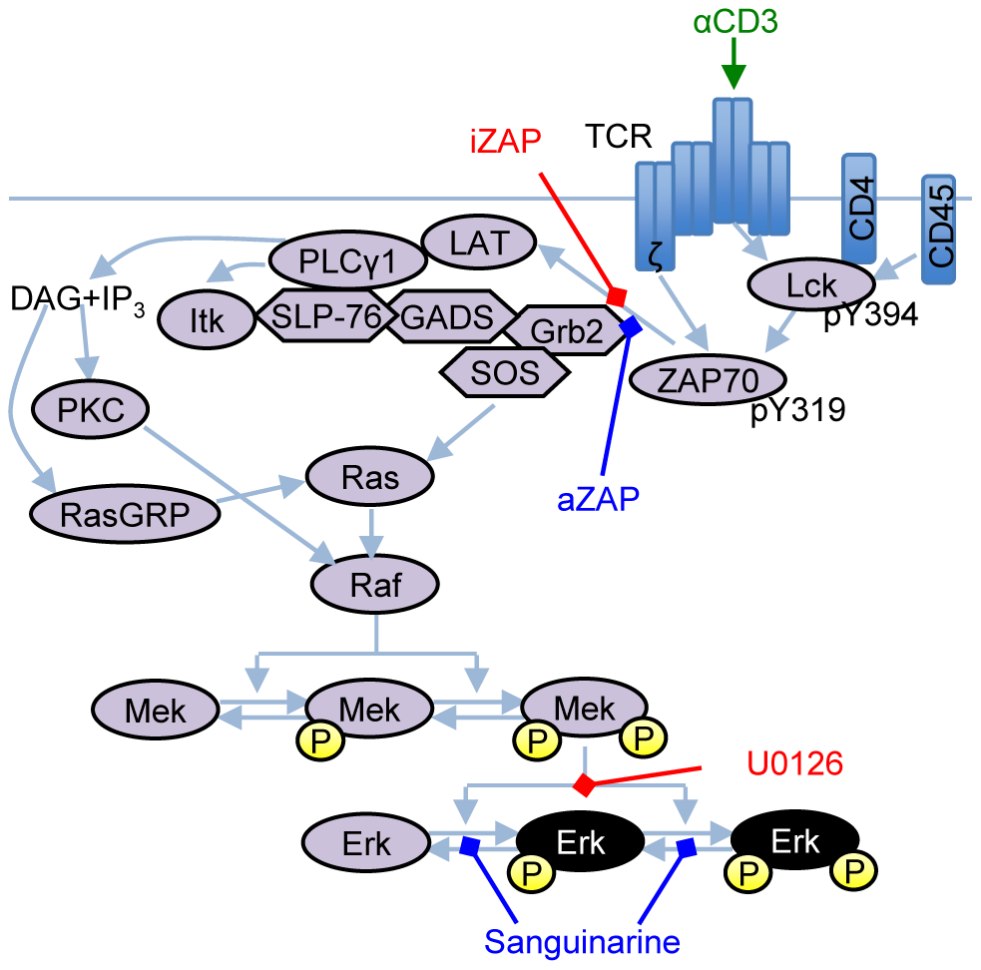
Generalized illustration of the TCR-mediated signaling pathway through the Erk-MAPK cascade. Arrow- and diamond-heads denote activation and inhibition of substrate molecules, respectively. TCR stimulation is achieved through αCD3 (green) binding. Subsequent Erk activation (black) controlled using small molecule inhibitors sanguinarine (blue) and U0126 (red).

The signaling pathway considered herein is the T cell receptor (TCR)-activated extracellular signal-regulated kinase (Erk, or MAPK) pathway (generalized in Figure 1). Activated Erk is an important condition in lymphocyte development and activation processes because it is a highly conserved and ubiquitous mechanism for transferring extracellular signals from membrane-bound receptors to the nuclear domain for gene regulation. The stimulation of TCRs by antigenic peptides (e.g. αCD3, shown in green in Figure 1) initiates a number of molecular reactions involved in signal transduction through Erk. During ligand binding, the TCR recruits and is phosphorylated by the tyrosine kinase Lck. A second tyrosine kinase, ZAP70, binds the tyrosine phosphorylated TCR subunits and is phosphorylated and activated by Lck. The receptor-kinase complex recruits and phosphorylates the adapter proteins LAT and Grb2 and phosphorylates and activates PLCγ leading to the formation of GTP-bound Ras. Activated Ras initiates the canonical Raf/Mek/Erk signaling cascade which results in T cells in the activation of the gene for interleukin-2.

### Prediction Model Bank

Three mathematical models of the TCR-mediated signaling cascade are used for the basis of the prediction model bank [5]–[7]. The model proposed by Zheng [5] (herein referred to as Model *Z*) contains primarily first- and second-order mass action kinetics with 24 ODEs and 53 reaction parameters. The second model (herein referred to as Model *L*) is the deterministic version of the model proposed by Lipniacki *et al.* [6], which explicitly incorporates SHP-mediated negative feedback and Erk-mediated positive feedback. Model *L* consists of 32 reaction parameters and 37 ODEs derived from mass action kinetics. The original version of the third model, presented by Klamt *et al.* [7], uses Boolean logic to describe the main steps involved in the activation of CD4^+^ helper T cells. *CellNetAnalyzer*, a Matlab software package [7] for structural and functional analysis of signaling networks, and the *Odefy* toolbox [28] were used to convert the logical model to a continuous homologue (herein referred to as Model *K*) with 40 states and 147 reaction parameters. Values for these reaction parameters were taken from Table 1 in [29]. All prediction models were modified to contain control inputs that simulate the actions of sanguinarine and U0126. In addition, model outputs (i.e. total concentration of phosphorylated Erk) are normalized so that the peak uncontrolled response scaled to unity in order to account for the differences in scale (see Section 1 in Text S1 for further details). All programming and simulation was performed in Matlab R2011b (7.13.0) and code is available in Dataset S1.

Herein, the computational burden of repeatedly evaluating large nonlinear ODE models is mitigated by using sparse grid interpolation. Sparse grids have been used as computational cost-cutting tools for control applications in systems biology [14], [30] by serving as surrogates for slow-evaluating models and objective functions to allow rapid screening of the design space.

### Open-Loop Multiple-Model Control with Adaptive Akaike Weights

**Figure 2:**
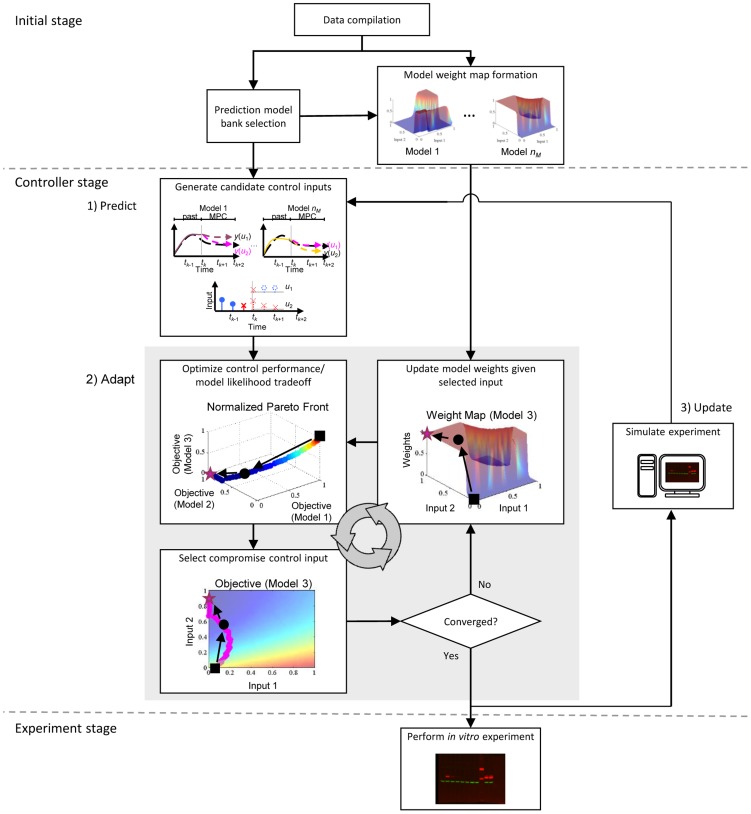
A framework for multiple-model open-loop control of uncertain intracellular signaling in the laboratory. First, the model bank is populated with a set of relevant models that predict the system response to possible control inputs. During the initial stage, training data are used to generate weights maps. These maps inform the controller of the tendency for models to differ in their ability to accurately reproduce the system dynamics under different control inputs. During each time interval of the controller stage, the performance metrics for the models are optimized simultaneously using a multiobjective technique within a MPC framework to generate a candidate solution set. The tasks involved in the adaptive model weighting strategy are contained within the gray box: control inputs are selected from the solution set by prioritizing them according to the weight maps, then model weights are automatically recalibrated using the portion of training data that most closely corresponds to the proposed control input. Optimization and input selection cycles repeat for subsequent time intervals as the prediction horizon slides along until the entire open-loop control sequence is specified and ready to be applied to the *in vitro* system.

In traditional model predictive control (MPC), also referred to as receding horizon control, the controller surveys the possible trajectories stemming from the current state and selects the control input sequence so that the predicted model outputs track the desired trajectories over a finite prediction horizon. The first control input of the selected sequence is used to update the prediction model state and the procedure is repeated as the prediction horizon slides along for the remaining time intervals. When based on a single model, the controller is at risk of degraded performance because of mismatch between the predicted and actual system behaviors. The proposed control strategy, illustrated in Figure 2, employs multiple prediction models to mitigate the effects of model uncertainty.

In our approach, first, the model bank is populated with a set of relevant models, each of which predicts the system response to possible control inputs (in control theory terms this is referred to as the plant response). During the initial stage, training data and the corrected-Akaike Information Criterion (AICc) are used to generate weight maps for the prediction models. These weight maps give the relative probability that a given model is consistent with training data at any given point in the feasible input space. In essence, these maps inform the controller of the tendency for models to differ in their ability to accurately reproduce the system dynamics under different control inputs. We then use a model predictive control framework in which the performance metrics for the models are optimized simultaneously using a multiobjective technique. Optimal control inputs are selected from the resulting Pareto solution set by prioritizing the solutions according to the pre-computed weight maps. Model weights are automatically recalibrated using the portion of training data that most closely corresponds to the proposed control input. Optimization and input selection cycles repeat for subsequent time intervals as the prediction horizon slides along until the entire open-loop control sequence is specified and ready to be applied (see *Materials and Methods* for further details).

### Description of *In Silico* and *In Vitro* Case Studies

As discussed previously, the purpose of this manuscript is to present a computational strategy to aid in the design of experimental input regimens to elicit predictable dynamical behaviors from biological processes. Herein, we demonstrate our approach using the well-known TCR signaling pathway both through simulation and laboratory experiments. First, we explored two *in silico* case studies, each considering a pair of control reagents acting on different regions of the T cell signaling pathway, to demonstrate the functionality of the proposed control strategy. In both case studies, we performed a series of experiments in which one model was selected from the aforementioned set of three models to serve as the simulated system. This model was also used to design the optimal control input regimen as a controller based on this model would match the actual system exactly and represent the best possible scenario. The remaining two models were then used to form the basis of the control strategies we wished to compare: single-model control (mismatched model), multiple-model control with fixed equal weights, and our proposed multiple-model control with adaptive Akaike weights. For the *in vitro* case study, a subset of the control input regimens derived from the simulated case studies were tested on populations of Jurkat T cells for experimental corroboration.

In each of our case studies of the TCR signaling pathway, we desired the system readout (i.e. total concentration of phosphorylated Erk) to follow a series of predefined time course trajectories. These target trajectories were characterized by the equation 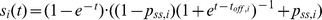, where *t* = 0 refers to the time at which the initial stimulation dose of αCD3 is administered. The term *p_ss_* represents the desired steady-state fraction of maximal activation that is to be achieved by the end of the 30-minute experiment. The term *t_off_* represents the time in minutes, following an initial interval of maximal activation, at which the controller should begin driving the output to the desired steady-state fraction. The ten parameter pairs chosen for this study are (*t_off_*, *p_ss_*) = {(8,0), (15,0), (22,0), (8,0.25), (15,0.25), (22,0.25), (8,0.5), (15,0.5), (22,0.5), (30,1)}. The five possible input dosing times began at 3 min post-αCD3-stimulation and were spaced 5 min apart to accommodate both the rapid dynamics of TCR signaling and the limitations of experimental input dosing and measurement rates.

The control inputs used to perturb the TCR signaling pathway were chosen based on both the need to demonstrate the efficacy of our methodology and for experimental corroboration. For our simulated and experimental case studies, we chose two commercially-available reagents known to control the dynamics of phosphorylated Erk: sanguinarine and U0126. Sanguinarine (Figure 1) is a small-molecule inhibitor of Erk dual-specificity phosphatase-1 (MKP-1) and leads to elevated Erk phosphorylation [31]. U0126 (Figure 1) on the other hand is a Mek inhibitor with high selectivity, which effectively inhibits activation of Erk [32]. To further evaluate controller performance using a variety of objectives, our second simulated case study replaces these reagents with two hypothetical reagents that modulate the function of phosphorylated ZAP70. The two reagents, aZAP and iZAP (Figure 1), act to promote and inhibit the function of pZAP70, respectively. Sanguinarine and U0126 concentrations were constrained to the intervals [0, 50] µM and [0, 10] µM, respectively, the upper limits of which were estimated from experimental results that indicate saturation effects at levels above the specified concentrations (see Figure 8 in *Materials and Methods*). aZAP and iZAP were normalized to the interval [0, 1] as they are hypothetical. For details on our *in silico* implementation of all control reagents, readers are referred to Section 1 in Text S1 and to the Matlab code provided in Dataset S1.

### *In Silico* Experiments

**Figure 3:**
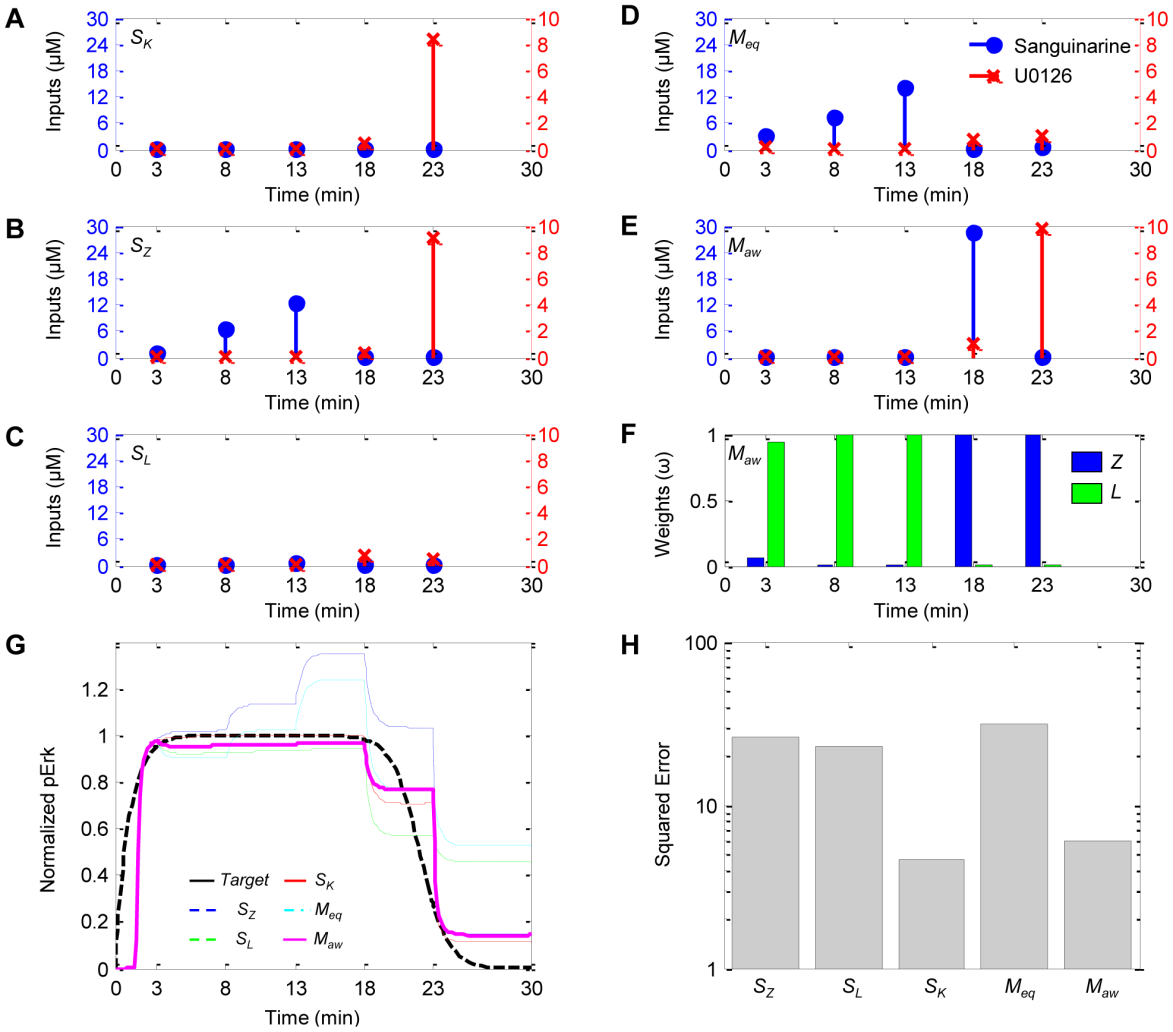
Simulations indicate adaptive weighting strategy significantly improves overall target tracking performance. (**A**–**E**) Control input dosing regimens for the matched (*S_K_*) and mismatched (*S_Z_* and *S_L_*) single-model controllers and the multiple-model controllers with fixed equal weights (*M_eq_*) and with adaptive Akaike weights (*M_aw_*). (**F**) Akaike weights for *M_aw_*. (**G**) Target trajectory (solid black) and simulated system (Model *K*) responses controlled by *S_K_* (dashed red), *S_Z_* (dotted blue), *S_L_* (dotted green), *M_eq_* (dashed cyan) and *M_aw_* (solid magenta). (**H**) The squared error values for all five controllers.

The control strategy was first tested by considering combinations of two models at a time to control an unknown system, which is simulated by the remaining third model. In the following discussion, we will use *S* to denote single-model control with a subscript to denote the model (*Z*, *L*, or *K*) and *M* to denote multiple-model control with the subscript indicating either equal weights (*eq*) or adaptive weights (*aw*). For illustrative purposes, the case in which Model *K* was the simulated system and the target profile corresponded to full termination at 22 min (i.e. (*t_off_*, *p_ss_*) = (22, 0)) is described and illustrated in detail (results for all experiments are provided in Sections 3 and 4 in Text S1). Figure 3 shows the control input dosing regimens for (A) the matched single-model controller *S_K_*, (B–C) the two mismatched single-model controllers *S_Z_* and *S_L_*, (D–E) the multiple-model controller with fixed equal weights (*M_eq_*) and with adaptive Akaike weights (*M_aw_*), (F) the Akaike weights for *M_aw_*, (G) the simulated system responses, and (H) the squared error values for all five control strategies.

The ideal scenario was defined to be one in which the model used to derive the control inputs is the same as the model used to simulate the actual system response, although it is generally not feasible in the laboratory and thus considered only for the purpose of theoretical comparison. For this ideal scenario, the control input regimen necessary to track the target profile included negligible input quantities to maintain initial Erk activation, quickly followed by a large bolus of U0126 at 23 min to promote Erk dephosphorylation near the end of the experiment (Figure 3A). As shown in Figure 3, the single-model controllers specified qualitatively different control inputs profiles, each causing qualitatively different simulated system responses. To achieve the sustained pErk activation phase of the target profile, *S_Z_* required a ramp-up in the sanguinarine doses (Figure 3B), which consequently caused the simulated system to systematically overshoot the target (Figure 3G, blue). On the other hand, *S_L_* predicted that negligible quantities of either reagent are necessary (Figure 3C), which caused the simulated system to track the target moderately well, undershooting the target only slightly (Figure 3G, green). For the rapid dephosphorylation phase, *S_Z_* specified a large dose of U0126 at 23 min while *S_L_* specified relatively small doses over the final two intervals. In contrast to the activation phase, the small doses specified by *S_L_* were insufficient to track the desired rapid transient behavior; the large dose specified by *S_Z_* produced significantly better tracking. Considering the experiment as a whole, however, neither controller adequately controlled the simulated system over the entire time interval.

The multiple-model controller with equal weights (*M_eq_*) considered the predictions from both models equally in specifying the control inputs (Figure 3D), but tracked only marginally better than either of the mismatched controllers *S_Z_* or *S_L_* (Figure 3G, cyan). On the other hand, the adaptive weighting strategy allowed the multiple-model controller to inherit the best characteristics of both single-model controllers (Figure 3E). According to Figure 3F, the weights tended toward Model *L* as it was the better representative of the simulated system initially, then shifted toward Model *Z* as it more accurately described the rapid dephosphorylation dynamics possessed by the simulated system. As a result, *M_aw_* tracked the target significantly better with at least a 74% reduction in the squared error (see Figure 3H) than any of the mismatched single-model and fixed equal weight controllers as indicated in dynamics shown in Figure 3G.

**Figure 4:**
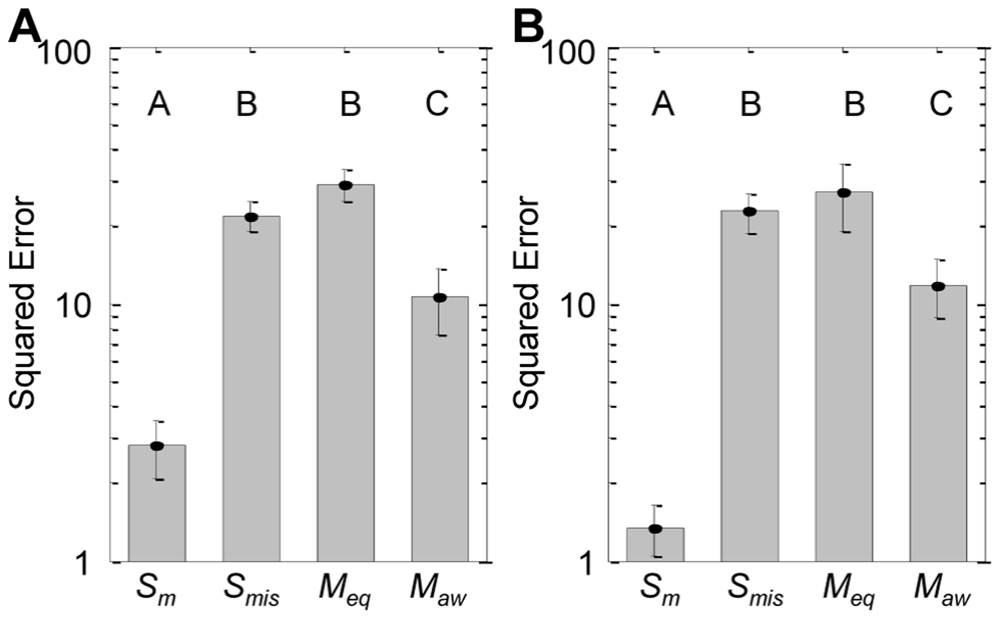
Summary of target tracking performances for the simulated case studies. (A) Summary of experiments involving realistic control reagents sanguinarine and U0126. (B) Summary of experiments involving hypothetical reagents aZAP and iZAP. Target tracking performance is measured by the squared error between target profiles and controlled plants. Data shown are mean ± standard error between matched (*S_m_*, n = 30) and mismatched (*S_mis_*, n = 60) single-model controllers, *M_eq_* (n = 30) and *M_aw_* (n = 30). Group letters denote statistically significant differences between groups (p<0.05) as calculated by one-way ANOVA with Tukey multiple comparisons test (SigmaStat v3.5, Systat Software, Inc).

Controller performances for the simulated case studies involving the real commercially-available control reagents sanguinarine and U0126 and the hypothetical control reagents aZAP and iZAP are summarized in Figures 4A and 4B, respectively. Both plots show the target tracking error between the predicted system dynamics and the target trajectories for all target and system model combinations. Matched single-model scenarios (*S_m_*) are cases when the model used to design the control inputs is identical to that which is used to simulate the system response while the mismatched single-model scenarios (*S_mis_*) use different models in both roles, the latter case tending to be the better representation of biological reality. As shown in both plots of Figure 4, *S_mis_* had relatively large error values and tracked quite poorly. *M_eq_* were able to partially mitigate these effects by averaging out some of the inconsistencies, but still performed no better than *S_mis_*. The proposed controller strategy (*M_aw_*) performed significantly better than *S_mis_* and *M_eq_* by preferentially selecting predictions from the models that were known to match the desire behavior at any point in time. This effect was more pronounced in scenarios where the dynamics differ among the prediction models because the controller was better able to filter out the inadequate models, thus improving tracking performance relative to the other controllers. Notably, the only scenarios not outperformed by *M_aw_* were those in which a matched prediction model was used; however, these cases would be unrealistic in practice.

### *In Vitro* Experiments Using Jurkat T Lymphocytes

**Figure 5:**
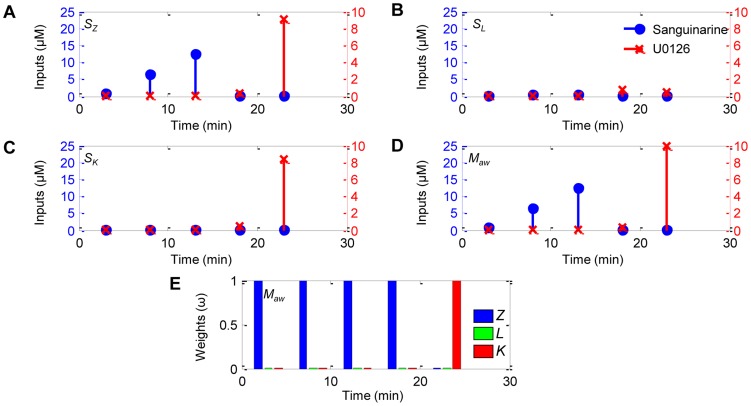
*In vitro* experiments demonstrate superior target tracking performance by *M_aw_*; corroborates observed *in silico* trends. (**A**–**D**) Control input dosing regimens for single-model controllers (*S_Z_*, *S_L_* and *S_K_*) and the multiple-model controller with adaptive Akaike weights (*M_aw_*). (**E**) Akaike weights for *M_aw_*.

**Figure 6:**
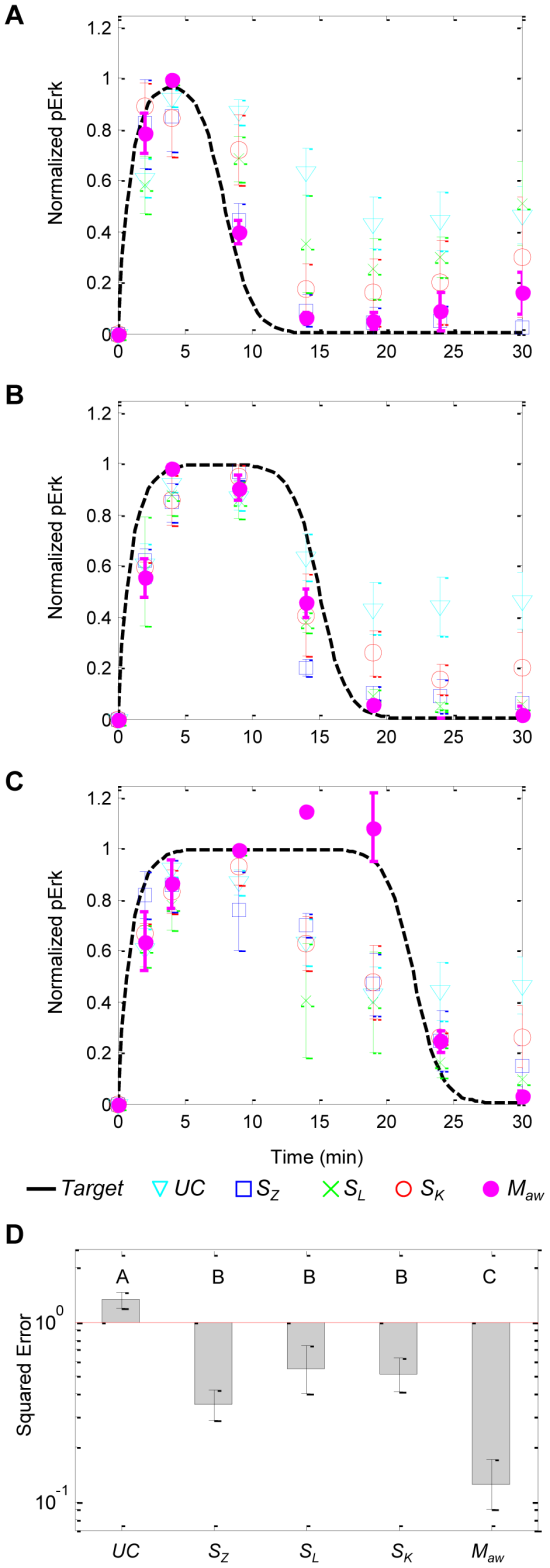
Overall *in vitro* target tracking performances between target profiles and measured Erk phosphorylation. Experiments with target trajectories (solid black) defined by the (*t_off_*, *p_ss_*) pairs of (**A**) (8,0), (**B**) (15,0) and (**C**) (22,0). Data are measurements of plant dynamics that were uncontrolled (*UC*, cyan triangle, n = 12) and controlled by *S_Z_* (blue square, n = 9), *S_L_* (green x, n = 9), *S_K_* (red circle, n = 9) and *M_aw_* (magenta dot, n = 9). Data shown are mean ± standard error. (**D**) Controller performances as measured by squared error between target trajectories and controlled plant dynamics. Group letters denote statistically significant differences between groups (p<0.05) as calculated by one-way ANOVA with Tukey multiple comparisons test (SigmaStat v3.5, Systat Software, Inc).

We conducted a set of experiments where control input regimens were computed considering all three prediction models and implemented *in vitro* in Jurkat T cells according to the experimental protocol described in *Materials and Methods*. Since the equal weighted multiple-model controller design did not improve performance when compared with the single-model controllers for the simulated experiments, we did not include those in the more expensive *in vitro* study. The model weight maps were trained using preexisting experimental data (see Figure 8 in *Materials and Methods*). Figure 5 shows for a representative experiment (A–D) the computed control input dosing regimens for *S_Z_*, *S_L_*, *S_K_* and *M_aw_*, and (E) the Akaike weights for *M_aw_* and (F) quantitative Western blot data. The specified target in the illustrated case required that Erk undergo rapid and sustained phosphorylation for 22 min and then rapidly return to steady state at basal levels. The quantitative Western blot data for this exemplar experiment are shown in Figure 6C. Without any external manipulation, pErk returned to its basal level slower and sooner than desired (Figure 6C, cyan triangle). Of the three single-model controllers, only *S_Z_* (Figure 5A) correctly predicted that an initial ramp-up in sanguinarine was required to sustain pErk levels as desired. Based on the *a priori* information contained in the model weight maps, *M_aw_* was preferential towards the predictions made by Model *Z* because it most accurately reflects the cell behavior (Figures 5D and 5E). However, Model *Z* was unable to replicate the rapid transient behavior of pErk in Jurkat cells in response to U0126 as accurately as the other models. In this case, *M_aw_* deferred to the predictions made by Model *K*. Because of this ability to adapt the weights based on the current conditions, *M_aw_* was able to control pErk much more tightly over the entire experiment than any of the models considered in isolation (Figure 6C, magenta dot).

Overall controller performances, defined as the squared error between the target output profiles and the corresponding observed plant dynamics, for the aforementioned experiment as well as the two other performed corroborating experiments are summarized in Figure 6D (descriptions of all experiments are provided in Section 5 of Text S1). Unsurprisingly, the largest deviations from the target trajectory were the uncontrolled responses. The single-model controllers improved tracking performance, but varied quite widely due primarily to the degree of how accurately the mathematical model predicted the signaling response (extent of plant-model mismatch). On the other hand, the intracellular signaling dynamics were much more tightly controlled by our multiple-model controller with adaptive Akaike weights, corroborating our *in silico* findings. The adaptive weighting strategy reduced the squared error by at least 63% over the best performing single-model controller. This indicates that our proposed control strategy is able to successfully filter out the prediction models in situations where they are known to be inaccurate and include them otherwise.

## Discussion

Recent studies have advanced and evaluated engineered control strategies to direct cellular processes in a desired manner. A few notable works that used feedback control approaches paired *in silico* control with experimental corroboration include controlling cell differentiation via nonlinear MPC [13], [14] and long-term feedback control of gene expression in yeast [15], [16]. This study augments this literature base by providing a novel and experimentally-corroborated methodology to enable the successful design and implementation of open-loop control strategies. Feedback control has many advantages over open-loop control since it uses measurements during the event to adjust the control inputs based upon the system response. This closed-loop approach helps to overcome inaccuracies of the supporting mathematical model. However, feedback control is not always possible due to experimental limitations. Open-loop control pre-specifies the control inputs: like a recipe. The feasibility of our open-loop control approach to overcome the inaccuracies of a single mathematical model has been demonstrated by experimental signal tracking by an intracellular signaling pathway.

While the concept of multiple-model-based control is not new, it is employed nearly exclusively in the closed-loop framework [14], [22]–[25], where system data are collected to inform future controller inputs. For the TCR-mediated Erk activation pathway and similar systems, the biological reality and practical experimental constraints necessitated the use of multiple mathematical models in an open-loop framework to achieve sufficient control. Because of these constraints, we formulated a new technique for control input selection that did not require real-time feedback data. Our novel technique uses what limited quantitative data are already available to generate a series of weight maps that indicate the likelihood of each model for any feasible control input scenario. When selecting the appropriate control input, these maps serve as a sort of filter that emphasizes data most relevant to the given scenario and excludes data that would only confound the selection process. Thus our approach effectively utilized multiple models with an adaptive weighting scheme to plan the control inputs.

Selecting such control inputs from multiple models with competing predictions generally does not have a unique solution because the criterion by which a solution is chosen depends entirely upon the decision maker. In this multiobjective optimization framework, each objective can be defined as controller performance based on one model. Attempting to satisfy multiple conflicting objectives will lead to a family of solutions (i.e. Pareto solutions) for which any improvement in one objective comes at the cost of at least one other objective. While the Pareto front topography is method-independent, choosing a particular solution along the front depends entirely upon the preferences of the decision maker. Typically a scalarization technique is used to compact the multidimensional problem into a single objective. Zarei *et al.* formulated a fuzzy rule set comprised of linear membership functions to aggregate the objectives based on the authors' preferences to control HIV dynamics in a CD4+ T cell population [33]. Another group employed a trade-off method in which the chosen Pareto point is closest in some sense to the decision maker's aspiration level, or desired value of each objective [34]. A trade-off operator changes the aspiration level based on which criterion the decision maker wants to improve if the suggested point is not satisfactory. Promethee uses a pair-wise outranking relation among all Pareto points so that a satisfactory solution is identified as soon as the points are found [35]. The relation is typically fixed once established and cannot update itself automatically. Bemporad *et al.* proposed choosing control inputs based on a time-varying, state-dependent decision criterion that is informed by real-time observational feedback [36]. Unfortunately, most available methods share a common challenge in that they are not fully automated, require observational feedback or are application-specific. Because our goal is to control uncertain intracellular signaling dynamics, we define our preference between objectives to be the relative likelihoods of the models as quantified by the Akaike weights. This allows us to automatically determine an optimal “blend” of the various models based on existing data and predicted input conditions to maximize prediction confidence while controlling for target tracking error.

### Remarks on General Use and Limitations

We have presented a method that was shown to improve target-tracking performance in a biochemical system with relatively high modeling uncertainty and measurement noise. Naturally, we employed our own knowledge of the system as well as that taken from literature to ease the implementation of the control method in the laboratory setting. Even so, the proposed framework is general enough to be employed in a wide variety of engineering applications. The method is suitable for any physical system with possible dynamics that can be characterized by a set of mathematical models. The models should include feasible control inputs that are capable of manipulating the output dynamics and can be structurally unique. Furthermore, the ensemble of models should adequately recapitulate the relevant behaviors of the system. The method does not require real-time observability in its current open-loop configuration, although observations should not be ignored when available. While we have tested and corroborated the method only with nonlinear ODE models, we believe the modeling format is an application-related issue and not restricted by the method.

Although our proposed framework is widely applicable, its efficacy for a given system depends on a variety of modeling and experimental constraints (i.e. problem-specific information used to inform the controller), and computational constraints (i.e. control problem dimensionality).

From a modeling and experimental perspective, efficacy depends on how well the system is characterized by the prediction model bank, the accuracy of the model weight maps and the availability of quantitative measurement tools or assays and control reagents or actuators. First, the prediction model bank should be formulated in such a manner that all desired system behaviors are within the reachable dynamics of the ensemble of models. Any characteristic behaviors or operating points not included in the model bank would not be able to be recapitulated, regardless of the adaptive model weighting scheme. However, these behaviors or operating points may be captured by including models with alternate parameter values or equation structures. In the case studied in this manuscript, each model had a different equation structure, but represented alternate plausible mechanisms that are at least partially supported by data.

Second, the accuracy of the model weight maps depends on the quantity and quality of preliminary data. Without any prior information, the models are considered equally when selecting control inputs. As experiments are performed, the gathered data can be used to train the weight maps to the actual model-system relationship. Models can then be selected according to its capacity to accurately predict the effects of the inputs on the system. An implicit assumption here is that if a model is good at predicting the effect of some input at some time, it will be good at predicting the effect of another input at another time. While this assumption may not always be true, the known dynamics, developed and refined based on considerable experimental work, provide extensive constraints on what the possible unknown dynamics could be.

Finally, while our method does not require data in real time because of the open-loop formulation, accurate quantitative assays and specific control reagents are highly beneficial in the development of accurate models and model weight maps help decrease the effects of plant-model mismatch, a common failure mode in open-loop control. Even so, with the proposed adaptive weighting procedure, each model need only be partially data-consistent. We were able to demonstrate control using quantitative Western blots to measure Erk phosphorylation dynamics in T cells, despite the fact that Western blots are notoriously difficult to use and chemical reagents generally have some off-target effects.

Computational tractability is another important area to consider. To get the most benefit from the adaptive model weighting strategy, our control strategy involves a multiobjective optimization problem (of dimension *n_M_*×*n_y_*) at every time interval. In practice, this is equivalent to solving a series of single-objective optimization problems (of dimension *n_u_*×*H_u_*), the number of which depends on the desired Pareto front resolution. In this manuscript, we greatly reduce the complexity of our control problem by considering only three models, a single controlled output, and two discretized control inputs and a control horizon of one interval (at a time). However, it would not be far-fetched to have a control problem with potentially dozens of models, multiple inputs and outputs, and an extended control horizon. In such a case, it would be critical to reduce the control problem down to a computationally tractable size. This can be achieved a number of ways. For instance, dimensions corresponding to different model outputs can be scalarized using weighted aggregation. Also, dimensions corresponding to models that are redundant or dominated (i.e. low Akaike weights throughout the input space) should be removed.

### Uncertainty in the Model Weight Maps

As previously mentioned, the purpose of the weight map strategy is to help estimate an optimal blend of the considered models to create the best possible compromise input solution given modeling uncertainty. The accuracy of these maps depends heavily on the quantity and quality of the preliminary data used to train the maps. Without any prior information, there would be no evidence supporting one model over another and the resulting weight map topology would be flat. Inversely, if the system dynamics are known with certainty, but requires more than one model to recapitulate them all, then the resulting weight map topology would appear digital. That is, only one model would dominate in any given experimental scenario and the transition between which model dominates would be immediate.

**Figure 7:**
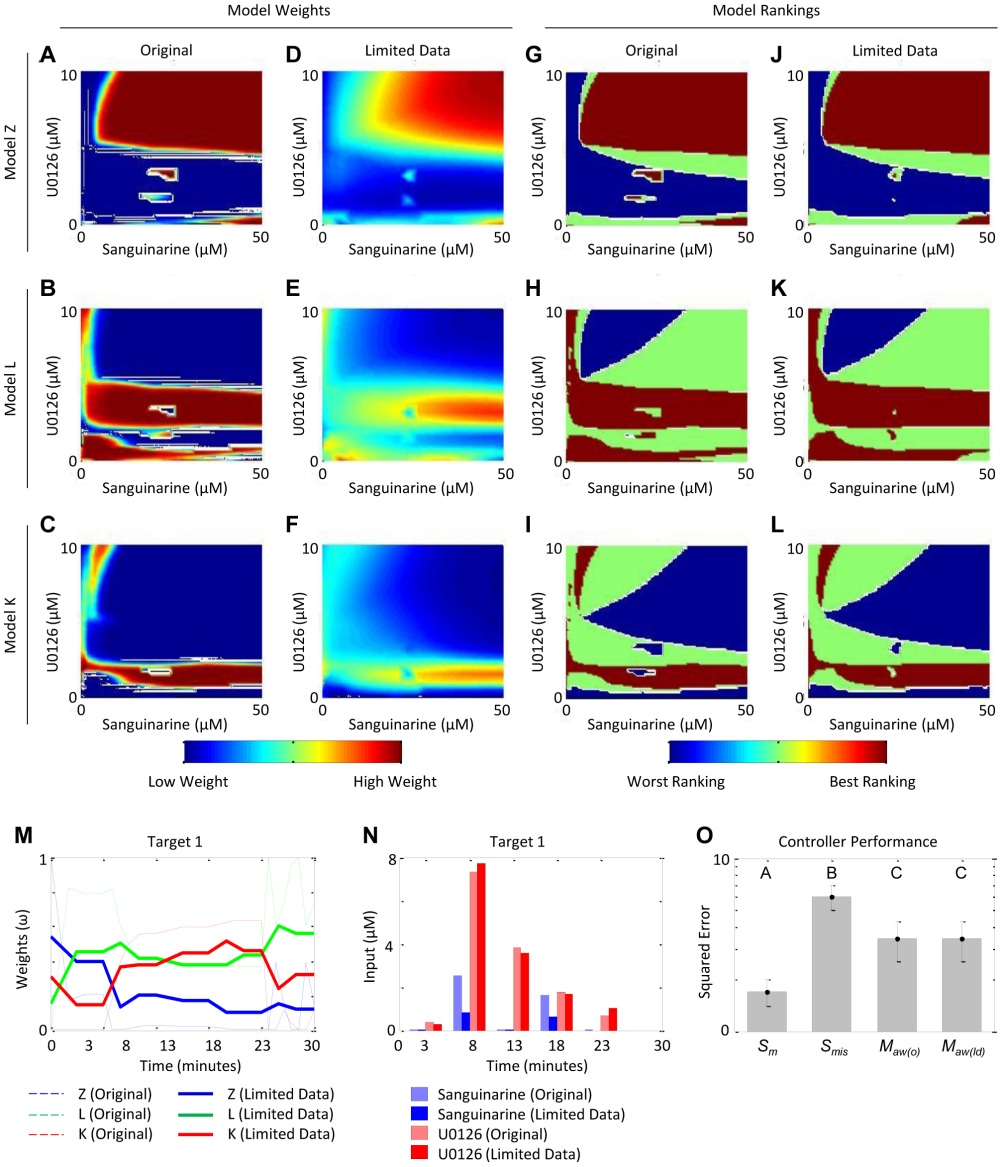
Analysis of uncertainty in the model weight maps and resulting predicted control strategies and performance. Model weights over the input space for (**A**–**C**) the original case study (full dataset) and (**D**–**F**) the case study with the limited dataset. Model rankings over the input space for (**G**–**I**) the original case study (full dataset) and (**J**–**L**) the case study with the limited dataset. (**M**) Adaptive weights and (**N**) corresponding control input regimen for the exemplar experiment characterized by the target trajectory defined by the pair (*t_off_* = 8, *p_ss_* = 0). (**O**) Controller performances as measured by squared error between target trajectories and plant dynamics controlled by single-model controllers that are matched (*S_m_*, n = 30) and mismatched (*S_mis_*, n = 60), and adaptively-weighted multiple-model controllers with the original “digital” weight maps (*M_aw(o)_*, n = 30) and smoother weight maps from the limited dataset (*M_aw(ld)_*, n = 30). Data shown are mean ± standard error. Group letters denote statistically significant differences between groups (p<0.05) as calculated by one-way ANOVA with Tukey multiple comparisons test (SigmaStat v3.5, Systat Software, Inc).

In our presented experimental study, we considered a case in which a substantial amount of time-course data (432 individual measurement points in total) was used to train the model weight maps. Due to the relatively large number of data, the AICc tends to show a strong sensitivity to any differences in model fitness values, causing the weight map topography to appear somewhat digital in nature (Figure 7A–C). However, let us consider a case in which we have fewer data (144 individual measurement points in total), or alternatively, more uncertainty in our models' ability to recapitulate the observed system behaviors. With fewer data, the AICc tends to show a weaker sensitivity to differences in model fitness values, causing the weight map topography to appear smoother (Figure 7D–F). Although the weight maps for these two scenarios are clearly different quantitatively, the order in which the models are prioritized (i.e. model rankings) are essentially identical (Figure 7G–L). To illustrate the effects of these characteristics, let us consider one exemplar experiment (Figure 7M). When utilizing the full dataset, the control strategy tends to heavily favor one model over the others at any given time. However, when the limited dataset is considered, the time course is covered by a non-trivial (i.e. non-digital) combination of the models, each representing a portion of actual system behavior. Even so, the orders in which the models are prioritized are very similar between the two cases. This means that for any given region of the input space, the choice of model providing the most reliable information is somewhat robust to the amount or quality of the training data. As a result, the predicted control regimens (Figure 7N) and their corresponding target tracking performance values (Figure 7O) also exhibit this same qualitative robustness.

### Importance of Erk Signaling in T Lymphocytes

T lymphocytes are an integral part of the human body's natural defense against the threats of invading pathogens and cancerous host cells. The function of these specialized immune cells largely depends on their phenotypic response to external stimulating signals. Propagating extracellular signals to their target substrates takes the coordinated effort of very large networks of molecular species with complex interactions ranging across vastly different spatial domains and temporal scales. While there are numerous mediators of signal transduction in lymphocytes, Erk is particularly noteworthy as it is an evolutionarily-conserved and ubiquitous group of signaling proteins critical to T cell development, proliferation and differentiation. Studies have linked Erk-mediated regulation to the differentiation of helper T cells into certain subtypes, particularly Th1 and Th2, and to allergies, asthma and serious immune disorders if improperly subtyped [1], [2]. Furthermore, it has been recognized that controlling the Ras/Raf/Mek/Erk pathway maybe beneficial towards advancing effective therapies for leukemia [37]. Constitutive activation of the Erk pathway is present in a high frequency (>50%) in patients suffering from acute myeloid leukemia (AML) and is associated with a marked reduction in survival duration [3], [4]. Conversely, blocking Erk activation has been shown to cause cell death in leukemia cell lines [3]. Treatments based on methods that work to balance these opposing forces to restore proper Erk-mediated regulation in T cells would be highly beneficial to patients suffering from such pathologies. Historically this has been the subject of experimental research [38], [39]. This study has confirmed that when used in combination the existing mathematical models of the Erk/MAPK pathway in T cells can support the engineering of control inputs to manipulate the activation and deactivation time course in a desired manner.

### Conclusions

We have developed a practical framework for controlling uncertain nonlinear systems using multiple models to generate predictable open-loop dynamical responses. The embedded model weight maps enable the controller to estimate the likelihood of each model in any feasible control scenario based on prior training data. The adaptive weighting strategy allows the controller to purposefully select subsets of the training data so that control decisions are made considering only the most relevant information at each time interval. Our open-loop controller design pairs model predictive control with an adaptive model weighting system based in information theory to create a cohesive strategy for systematically utilizing the most relevant knowledge embedded within limited training data in a computationally tractable manner. In both simulated and laboratory experiments this multiple-model control strategy and adaptive weighting scheme successfully reduced the open-loop target tracking error by more than half relative to multiple-model control with fixed weights (simulation only) and single-model control.

## Materials and Methods

### Experimental Protocol

Erk phosphorylation (pErk) data were collected from Jurkat T leukemia cell line (Jurkat clone E6.1; ATCC). Cells were grown in RPMI 1640 (Sigma) supplemented with 7.5% heat-inactivated fetal bovine serum (BioWest), 1 mM sodium pyruvate (Gibco), 12.5 mM HEPES pH 7.4 (Sigma), 12 µM sodium bicarbonate (Sigma) 50 µM 2-Mercaptoethanol (Sigma), 50 µg/ml streptomycin and 50 units/ml penicillin in an incubator at 37°C in humidified air containing 5% carbon dioxide. Cells were harvested in log-phase growth at a density of 2×10^7^ cells per treatment. Cells were stimulated using anti-human CD3 (10 µg/ml, clone: UCHT-1, eBioscience) as the stimulatory signal at 37°C in a water bath. Cells were treated with the Mek1/2 inhibitor U0126 (Calbiochem) or the MKP inhibitor sanguinarine (Sigma), depending on the protocol, dissolved in DMSO at the indicated time points with the indicated concentrations. Experimental control samples were treated with the same amount of DMSO. Samples of 2×10^6^ cells were taken at the indicated time points and lysed in 1% NP40 lysis buffer (1% NP40, 25 mM Tris, pH 7.4, 150 mM NaCl, 5 mM EDTA, 1 mM NaV, 10 mM NaF, 10 µg/ml each of aprotinin and leupeptin) for 15 min on ice. Lysates were centrifuged for 5 min at 18000 g at 4°C. The supernatant was added to the same volume of 2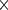 protein solubilizing mixture (PSM, 25% (w/v) sucrose, 2.5% (w/v) sodium dodecyl sulfate, 25 mM Tris, 2.5 mM EDTA, 0.05% bromophenol blue) and boiled for five minutes. Proteins were separated via SDS-PAGE, blotted for phospho-Erk1/2 (Cell Signaling), phospho-ZAP-70 (pY319, Cell Signaling) and GAPDH (Ambion). IRDye 800 and 680 secondary anti-mouse and anti-rabbit antibodies (Li-Cor) were used for signal detection using an Odyssey infrared scanner. Images of the blots were analyzed using ImageJ to produce quantitative data for model comparison. Model predictions were scaled to compensate for the fact that the data represent relative quantities only rather than absolute concentrations.

### Training Experiments

**Figure 8:**
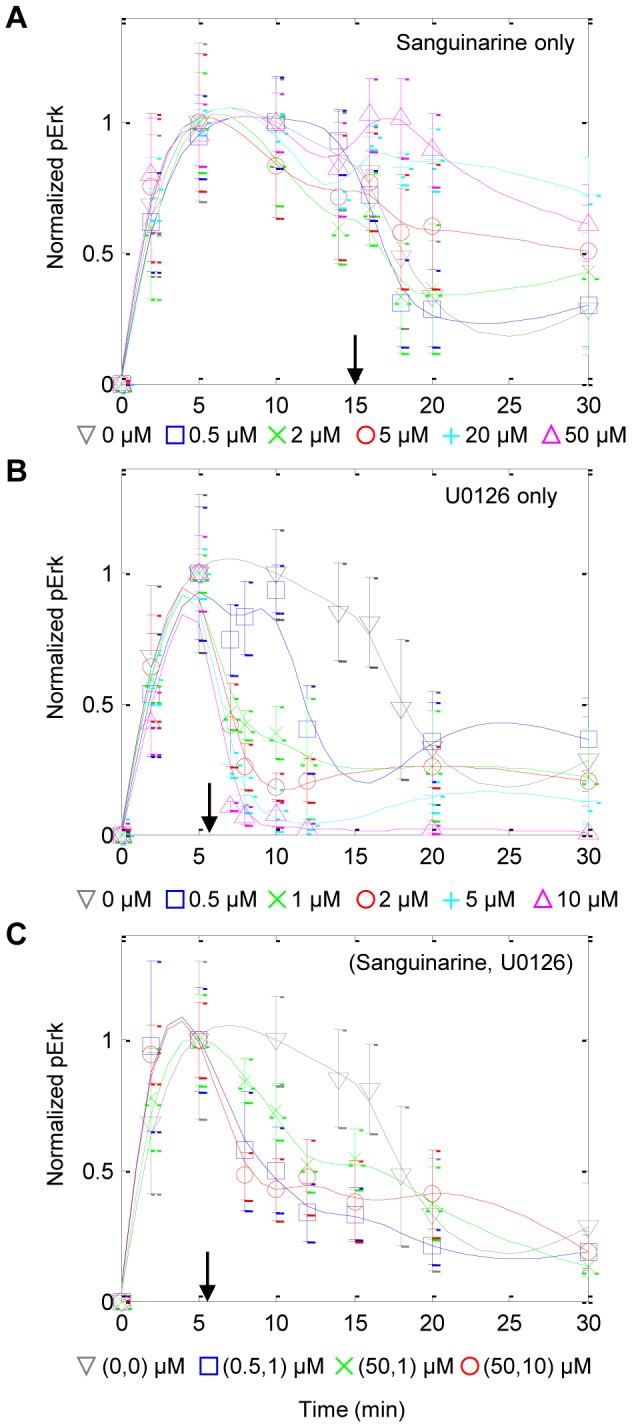
Training data used to generate the model weight maps. Rows correspond to doses of sanguinarine only, U0126 only, and combinations of the two reagents, respectively. Symbols and error bars denote means ± standard errors of the raw normalized data. Lines represent smoothed data. Arrows denote the time at which the reagents were administered.

Training experiments were designed to rapidly screen the responses of T cell populations to potential control reagent combinations. Fourteen different experiments were conducted: an experimental control (i.e. no control inputs), five individual doses of sanguinarine (0.5, 2, 5, 20 and 50 µM) at the 15 minute mark, five individual doses of U0126 (0.5, 1, 2, 5 and 10 µM) at the 6 minute mark, and three combined doses of 0.5 and 1, 50 and 1, and 50 and 10 µM for sanguinarine and U0126, respectively, at the 6 minute mark. These data were collected according to *Experimental Protocol*. The addition of low doses of sanguinarine had negligible effects on pErk concentrations. Only the moderate to high doses tested caused elevated to sustained phosphorylation of Erk (Figure 8A). On the other hand, U0126 produced an immediate reduction in the Erk phosphorylation rate even at low doses (Figure 8B) and tended to overpower the effects of sanguinarine when added together (Figure 8C). The representation of the controller input functions in the prediction models were modified to exhibit these trends.

Due to the prevalence of observation noise, experimental data 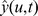 were smoothed using cubic smoothing splines to filter spurious oscillations from the time courses while retaining primary trends. This was performed in Matlab using the *csaps* function with the smoothing parameter *p* set to 0.6. The smoothing splines were sampled at 31 evenly spaced time points to increase the density of the time course data.

### Model Bank Representation

The proposed control strategy is based on a set of two or more mathematical models, 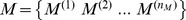, of a given system with the general form:
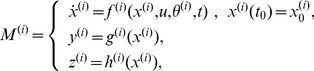
(1)where the superscript *i* denotes the model number. The state variables, control inputs, system parameters, measured outputs and controlled outputs are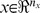, 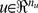, 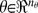, 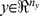 and 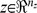, respectively, and 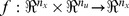, 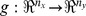 and 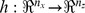 are twice continuously differentiable functions for the system dynamics, measured outputs and controlled outputs, respectively. Note that 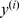 and 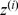 are functions of *u*, *t* and 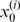. For our purposes we will use the notation 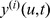 and 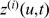.

### Approximating Model Dynamics with Sparse Grids

Sparse grids were implemented using the Sparse Grid Interpolation Toolbox for Matlab, version 5.1.1 [40], which is available at http://www.ians.uni-stuttgart.de/spinterp/. Our approach for approximating model dynamics using interval-based sparse grid interpolation is similar to that of [14]. The control inputs are assumed to lie within the bounded *n_u_*-dimensional space containing all feasible control input vectors, defined by
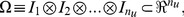
(2)where 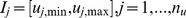 and 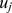 is the *j* ^th^ value of the input vector. For each model, *n_z_* output variables are selectively evaluated at points in the *n_u_*-dimensional input space and *n_t_*-dimensional time domain to form a series of 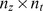 grids of dimension *n_u_* (see [14] for further details). On each grid, weighted Lagrange basis functions are combined at the support nodes to construct an input-domain interpolant with which the value of an output at a single time point can be estimated for any point in the input space. In this interval-based approach, interpolation between grids placed at various time points ensures a continuous trajectory over the prediction horizon for any point in the input space. To prevent excessive computational expense during grid construction, limits on absolute and relative error tolerances and allowable interpolation depth were specified (0.01, 1%, and 6, respectively).

### Model Weight Maps

For problem set-up, we estimate weight maps on Ω for the prediction models using Akaike weights, which are based on the Akaike Information Criterion (AIC). AIC provides a practical measure of the tradeoff between model fitness and complexity by estimating the theoretical Kullback-Leibler (KL) “distance”, or loss of information, between an approximating model and full reality [26]. Assuming normally distributed errors with constant variance and small sample sizes, the corrected-AIC (AICc) can be estimated as:
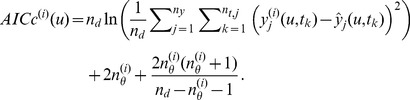
(3)where 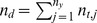 is the total number of experimental data and 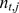 is the number of sampled time points for the *j* ^th^ measured output. The second term, where 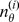 denotes the number of uncertain parameters for the *i* ^th^ model, is the bias-correction factor for the AIC (the factor 2 was introduced for historical reasons) and the third term is an additional correction factor for small sample sizes. Note that the first term includes the squared residuals between experimental data (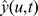) and their *i* ^th^ model counterpart (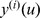). Under realistic conditions, 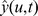 are sampled at discrete input quantities and time points and the number of data can vary between outputs. To generate a continuous approximation of 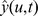 over Ω, a piecewise linear interpolant is constructed using the Matlab function *griddatan*. That is,
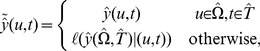
(4)where 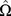 and 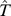 denote the discrete experimental input and observation time spaces, respectively, and 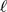 is an operator denoting (*n_u_*+1)-dimensional linear interpolation between existing points over 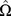 and 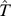 by means of Delaunay triangulation. In regions not explicitly measured, interpolated data (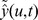) are used in place of 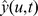 when computing AICc values.

It is important to note that only relative AICc values are meaningful due to the metric being a relative rather than absolute estimate of KL distance. It follows that the relative likelihoods of the models are given by the Akaike weights,
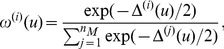
(5)where 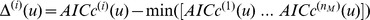. The weight 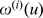 is interpreted as the strength of evidence in support, or relative probability, of the *i* ^th^ model being the KL best model from the set of models given the supporting data [26]. The weight maps for Model *Z*, Model *L* and Model *K* based on the data described in *Training Experiments* are provided in Figure 3 in Text S1.

### Open-Loop Model Predictive Control with Multiple Models

The multiple-model control strategy is built around the conventional MPC framework to reduce the computational complexity of the open-loop control problem. At each time interval, the controller surveys the possible trajectories stemming from the current state and selects the control input sequence over the control horizon (*H_u_*) so that the predicted outputs track the desired trajectories over the finite prediction horizon (*H_p_*). The first control input of the selected sequence is used to update the states of the prediction models and then stored as one entry in the final control sequence *U* ^*^ so the procedure can repeat as the prediction horizon slides along for the remaining time intervals.

The objective functions used to quantify controller performance penalizes the error between the predicted outputs for the *i* ^th^ model and the desired trajectories and a measure of the control effort over the prediction horizon starting at time 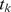 as given by


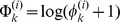
, where
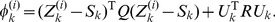
(6)The vectors 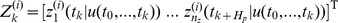 and 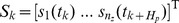 are the predicted and target outputs over *H_p_*, respectively, 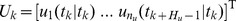 are the discrete controller inputs over *H_u_*. The proposed formulation assumes 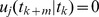, *j* = 1,…,*n_u_*, and *m* = *H_u_*,…,*H_p_* to reflect that manipulated variables are often applied as boluses in the considered biological context. The horizons *H_u_* and *H_p_* where each chosen to be one to prevent controllers from being overly conservative. *Q* and *R* are diagonal weighting matrices associated with the error and control effort, respectively, which were each chosen to be identity. Objective values are converted to log-space to compress the cost surfaces to facilitate optimization.

Each objective 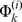 is approximated using sparse grid interpolation (similar to section *Approximating Model Dynamics with Sparse Grids*) to form an input-domain interpolant with which the value of each objective can be estimated for any point in the input space. If the approximations are sufficiently accurate, no further evaluations of the objective function or underlying state space model are required since the interpolants are generated prior to optimization during each time interval.

### Pareto Front Identification

We define the multiobjective optimization problem at the *k* ^th^ time interval as follows (with the standard Pareto interpretation of minimizers):
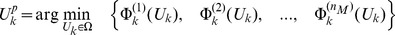
(7)


The 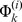 variable denotes the objective function for the *i* ^th^ model defined by (6) and the design variable *U_k_* is constrained to Ω defining biologically relevant limits. Herein we employ the normalized normal constraint method (NNC) for generating the Pareto solutions 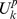 for its ability to generate a well-distributed set of global Pareto points ([41], refer to the original manuscript for further details).

NNC provides a geometrically intuitive approach to multiobjective optimization that is illustrated in Figure 4 in Text S1. It first builds a plane in the normalized objective space (called the *utopia hyperplane*) through all individual (normalized) minima 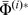, and second, generates equally distributed points in this plane 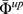 by systematically varying weights for each objective. Then for each point 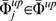, the corresponding solution 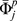 on the Pareto front 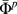 is found by minimizing the single (normalized) objective 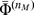 with added constraints. In addition to the original constraints, the feasible space is further restricted by adding *n_M_* −1 hyperplanes through 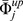 that are each normal to the *n_M_* −1 utopia plane vectors. Successive optimization runs are performed for the remaining points in 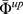. By translating the constraining normal hyperplanes between runs, we can see that the corresponding solution set along the leading edge of the objective space is generated. Since some of these points may represent non-Pareto optimal or dominated solutions, the NNC algorithm is coupled with a Pareto filter to remove such points.

### Input Selection and Model Weight Adaptation

The optimal control sequence for the *k* ^th^ time interval is selected from the set of Pareto solutions 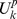 by ranking them using the objective
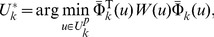
(8)where 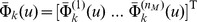 and 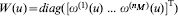 are vectors of objective function values and Akaike weights, respectively, corresponding to the control input vector 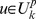.

**Figure 9:**
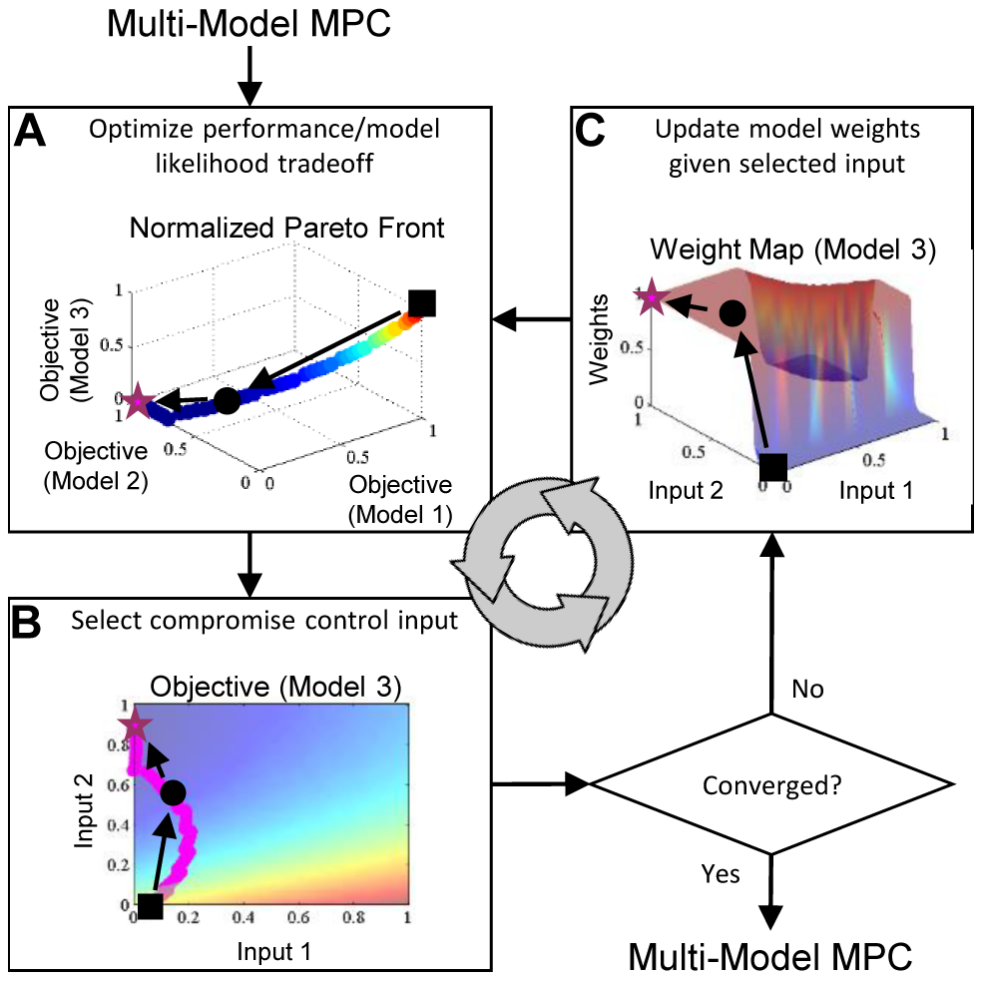
Illustration of control input selection with model weight adaptation. For a given prediction time interval in the control process, Pareto-optimal control inputs are computed by the multi-model MPC strategy by solving (7), which then enter an iterative process of control input selection and weight adaptation. (**A**) First, Pareto points are ranked with an initial weight vector ω_0_ and the optimal point (example: black square) is selected using (8). (**B**) Next, the input vector corresponding to the selected optimal point (*u*_1_, black square) is identified. If the input vector continues to change above a pre-defined threshold, the process continues to the next iteration. (**C**) Given the current input vector (*u*_1_), a new weight vector (*ω*(*u*_1_), black square) is computed. The process continues and repeats (example: black circle, then magenta star) until the aforementioned stopping criterion is met. The final input vector (*u_n_*, magenta star) is returned to the main control loop as the best compromise control strategy and used to update the prediction models in preparation for the next prediction time interval.

The models weights are adapted to accommodate the most relevant experimental data to ensure the best possible open-loop performance (Figure 9). At the first time interval, *u* is undetermined so the initial weights are computed considering the entire training data set. After the Pareto points 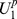 are specified by solving (7), they are ranked using (8) with the initial weights (Figure 9A) and the input vector corresponding to the best ranked point is taken as a temporary solution *u*_1_ (Figure 9B). The weights are then recalibrated at the value *u*_1_ (Figure 9C) and 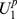 ranked again with the best ranked input vector taken as the new temporary solution *u*_2_ and so on. Updates continue until the model weights or control inputs no longer change above a prescribed threshold or the maximum allowable updates is reached. If a limit cycle is detected, (8) is recomputed by averaging the models weights in the cycle as a tie-breaker. The final input sequence is used to update the prediction models and appended to the growing open-loop control sequence *U* ^*^ as the prediction horizon slides along.

### Statistical Analysis

All statistical analysis was performed using SigmaStat v3.5 (Systat Software, Inc). Time-course data are shown as mean ± standard error at each time point. Statistical differences between groups (p≤0.05) are determined using one-way analysis of variance (ANOVA) followed by the Tukey multiple comparisons test. Target tracking performance values, as measured by squared error between target profiles and controlled plants, were log-transformed where appropriate to satisfy the normality and equal variance conditions for the ANOVA and Tukey tests.

## Supporting Information

Dataset S1 **Matlab code for proposed control algorithm and prediction models.** Contains all Matlab code necessary to implement the proposed adaptive weighted multiple-model predictive control algorithm, as well as code for the prediction models.
(ZIP)

Text S1 **Additional methods and results.** Contains details on specific model modification and additional simulated and experimental results.
(DOC)
