# Supplementary material for: Multiple Model-Informed Open-Loop Control of Uncertain Intracellular Signaling Dynamics
Source: PLoS Comput Biol. 2014 Apr 10;10(4):e1003546. doi: 10.1371/journal.pcbi.1003546 (PMC3983080; doi:10.1371/journal.pcbi.1003546)
Supplement: Dataset S1 — Matlab code for proposed control algorithm and prediction models. Contains all Matlab code necessary to implement the proposed adaptive weighted multiple-model predictive control algorithm, as well as code for the prediction models. (ZIP) [file pcbi.1003546.s001.zip › AW_MMPC/spinterp_v5.1.1/doc/spinterpdoc.pdf]

Universität
Stuttgart

Sparse Grid Interpolation Toolbox User's Guide

V5.1, February 24, 2008

Andreas Klimke

**Berichte aus dem Institut für
Angewandte Analysis und Numerische Simulation**

Documentation 2007/017

Universität Stuttgart

Sparse Grid Interpolation Toolbox User's Guide

V5.1, February 24, 2008

Andreas Klimke

Berichte aus dem Institut für Angewandte Analysis und Numerische Simulation

Documentation 2007/017

Institut für Angewandte Analysis und Numerische Simulation (IANS)
Fakultät Mathematik und Physik
Fachbereich Mathematik
Pfaffenwaldring 57
D-70 569 Stuttgart

E-Mail: ians-preprints@mathematik.uni-stuttgart.de
WWW: http://preprints.ians.uni-stuttgart.de

ISSN **1611-4176**

© Alle Rechte vorbehalten. Nachdruck nur mit Genehmigung des Autors.
IANS-Logo: Andreas Klimke. LaTeX-Style: Winfried Geis, Thomas Merkle.

Contents

1 Getting Started **7**

1.1 What is the Sparse Grid Interpolation Toolbox? 7
1.2 Initialization of the toolbox 8
1.3 A first example 8
1.4 Piecewise linear basis functions 11
1.5 Polynomial basis functions 13
1.6 Dimensional adaptivity 15

2 Advanced Topics **19**

2.1 Degree of Dimensional Adaptivity 19
2.2 Multiple output variables 22
2.3 Derivatives 24
2.4 Numerical Integration (Quadrature) 32
2.5 Optimization 36
2.6 Improving performance 41
2.7 Interfacing concepts 45
2.8 Approximating ODEs 49
2.9 External models 52

3 Functions – Alphabetical List **55**

cmpgrids 55
plotgrid 56
plotindices 57
spcgsearch 59
spcompsearch 62
spdim 64
spfminsearch 65
spget 68
spgrid 69
spinit 71
spinterp 71
spmultistart 73
spoptimget 75
spoptimset 76

sppurge 78
spquad 81
spset 82
spsurfun 88
spvals 89

License **93**

Bibliography **95**

Keyword Index **97**

1 Getting Started

1.1 What is the Sparse Grid Interpolation Toolbox?

Introduction

The interpolation problem considered with sparse grid interpolation is an optimal recovery problem (i.e. the selection of points such that a smooth multivariate function can be approximated with a suitable interpolation formula). Depending on the characteristics of the function to interpolate (degree of smoothness, periodicity), various interpolation techniques based on sparse grids exist. All of them employ Smolyak's construction, which forms the basis of all sparse grid methods. With Smolyak's famous method, well-known univariate interpolation formulas are extended to the multivariate case by using tensor products in a special way. As a result, one obtains a powerful interpolation method that requires significantly fewer support nodes than conventional interpolation on a full grid. The points comprising the multidimensional sparse grid are selected in a predefined fashion. The difference in the number of required points can be several orders of magnitude with increasing problem dimension. The most important property of the method constitutes the fact that the asymptotic error decay of full grid interpolation with increasing grid resolution is preserved up to a logarithmic factor. An additional benefit of the method is its hierarchical structure, which can be used to obtain an estimate of the current approximation error. Thus, one can easily develop an interpolation algorithm that aborts automatically when a desired accuracy is reached.

Major features

This Matlab toolbox includes hierarchical sparse grid interpolation algorithms based on both piecewise multilinear and polynomial basis functions. Special emphasis is placed on an efficient implementation that performs well even for very large dimensions $d > 10$.

There are many ways to customize the behavior of the interpolation routines. Furthermore, additional tasks involving the interpolants can be performed, such as computing derivatives or performing an optimization or integration. The following list gives an overview of the options that are available:

- Enable vectorized processing. Speed up the construction of the interpolant for functions that allow for vectorized evaluation.
- Create multiple interpolants at once for functions with multiple output arguments.
- Choose from three different grid types for piecewise linear interpolation. Depending on your objective function, a certain grid type may perform better than the others.

- If very high accuracies are required, you may use the Chebyshev-Gauss- Lobatto sparse grid, which employs polynomial basis functions.
- Compute gradients along with the interpolated values at just a small additional cost.
- Integrate the interpolant.
- Perform a search for minima and maxima using several efficient algorithms.
- Use the dimension-adaptive algorithm to automatically detect separability, and to take the importance of the dimensions into account when constructing the interpolant. This is especially useful in case of very high-dimensional problems when a regular sparse grid refinement leads to too many support nodes.
- Specify the minimum or maximum sparse grid depth to compute, or specify the minimum and maximum number of function evaluations to use (for the dimension-adaptive sparse grid).
- Last but not least, the Sparse Grid Interpolation toolbox is designed to easily integrate with your models in Matlab as well as external models.

For additional information on the theoretical and algorithmic aspects of sparse grid interpolation, please refer to the references provided at the end of this chapter.

1.2 Initialization of the toolbox

To initialize the toolbox, it must be added to the Matlab search path. You can do this by calling the function `spinit`, i.e. go to the directory containing the sparse grid interpolation toolbox and enter `spinit` at the Matlab prompt.

If you would like to use the Sparse Grid Interpolation Toolbox from within another Matlab application, you may call the `spinit` function as well. It will automatically add the correct paths to the Matlab path.

1.3 A first example

Let us interpolate a simple two-variate function

$$f(x,y) = \sin(x) + \cos(x)$$

with the default settings of the sparse grid interpolation package. Here, we interpolate the function for the domain $[0,\pi] \times [0,\pi]$.

Constructing the interpolant

First, we compute the hierarchical surpluses (i.e. the coefficients) of the interpolant.

```
f = @(x,y) sin(x) + cos(y);
z = spvals(f,2,[0 pi; 0 pi])
```

```
z =
                vals: {[65x1 double]}
            gridType: 'Clenshaw-Curtis'
                   d: 2
               range: [2x2 double]
            maxLevel: 4
         estRelError: 0.0063
         estAbsError: 0.0188
          fevalRange: [-1 2]
          minGridVal: [0 1]
          maxGridVal: [0.5000 0]
             nPoints: 65
           fevalTime: 0.0502
     surplusCompTime: 0.0024
             indices: [1x1 struct]
```

The function `spvals` returns these hierarchical surpluses, and also includes some additional information collected during the construction process of the interpolant. For instance, We obtain information on the estimated relative and absolute error. The number of sparse grid support nodes is provided, as well as the computing time for evaluating the function and computing the hierarchical surpluses. The surpluses themselves are stored under the field vals.

Computing interpolated values

To compute interpolated values, we can now use the `spinterp` function. To increase efficiency, multiple interpolated values can be computed at once. Below, we compute the interpolated values for five randomly chosen points and compare them to the exact function value by computing the maximum absolute error.

```
x1 = pi*rand(1,5); x2 = pi*rand(1,5);
y = spinterp(z,x1,x2)
```

```
error = max(abs(y - f(x1,x2)))
y =
    1.7173    0.7210    0.2675    0.7701    0.5510
error =
    0.0076
```

Visualizing the sparse grid

Let us now visualize the sparse grid. From the information returned by `spvals`, we see that the used sparse grid is of the type Clenshaw-Curtis, and the maximum level was 4. In two and three dimensions, we can easily plot the sparse grid with the `plotgrid` function. It takes the level and the dimension as input arguments. Optional is an options structure containing the sparse grid type, created with `spset`. The default grid type is the Clenshaw-Curtis grid, we thus do not have to specify the grid type here.

```
plotgrid(4,2)
```

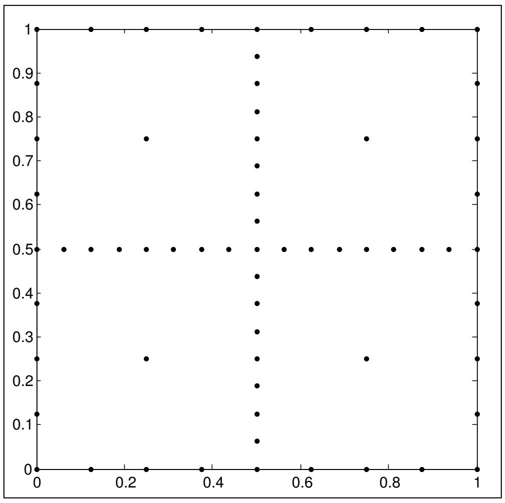

Visualizing the interpolant

To visualize the original function and compare it to the interpolant, we can plot both functions, for instance, by using `ezmesh`.

```
subplot(1,2,1);
ezmesh(f,[0 pi]);
title('f(x,y) = sin(x) + cos(y)');
subplot(1,2,2);
ezmesh(@(x,y) spinterp(z,x,y),[0 pi]);
title('Sparse grid interpolant, n = 4');
```

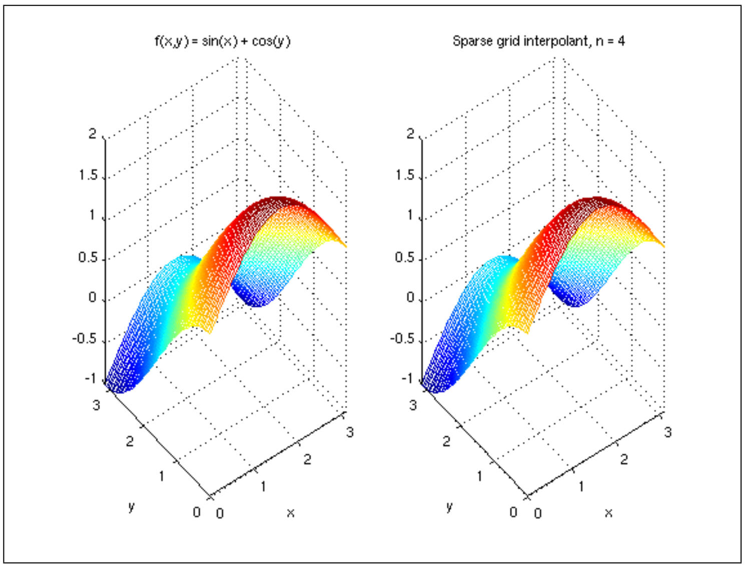

1.4 Piecewise linear basis functions

Piecewise linear basis functions provide a good compromise between accuracy and computational cost due to their bounded support. The Sparse Grid Interpolation package includes three different grid types that work with piecewise multilinear basis functions:

- The Clenshaw-Curtis grid type “ClenshawCurtis” (CC)
- the "classical" maximum-norm-based grid type “Maximum” (M), as described e.g. by Bungartz/Griebel in [1],
- The maximum-norm-based grid without points on the boundary “NoBoundary” (NB), with basis functions that extrapolate towards the boundary (it is not assumed that the objective function must be zero at the boundary).

For a detailed description of the piecewise multilinear basis functions implemented here, please see [2] or [3, ch. 3], and the references stated therein.

Accuracy of piecewise multilinear interpolation

We now take a brief look at the approximation quality. An a priori error estimate can be obtained for a d-variate function f if continuous mixed derivatives

$$D^{\beta} f = \frac{\partial^{|\beta|} f}{\partial x_1^{\beta_1} \cdots x_d^{\beta_d}},$$

with $\beta \in \mathbb{N}_0^d$, $|\beta| = \sum_{i=1}^{d} \beta_i$, and $\beta_1, \ldots, \beta_d \leq 2$, exist. According to [4] or [5], the order of the interpolation error in the maximum norm is then given by

$$\|f - A_{q,d}(f)\|_\infty = \mathcal{O}(N^{-2} \cdot |\log_2 N|^{3\cdot(d-1)}),$$

where $A_{q,d}(f)$ denotes the sparse grid interpolant of f, and N denotes the number of grid points of the sparse grids of type CC or M (the NB grid type has not yet been analyzed, but shows the same order of convergence in numerical tests). Note that the number of grid points N of $A_{q,d}(f)$ can be computed by `spdim(q-d,d)`. Piecewise multilinear approximation on a full grid with N^* grid points is much less efficient, i.e. $\mathcal{O}(N^{*-2/d})$.

Number of grid points

Table 1.1 shows the number of grid points of the non-adaptive sparse grid interpolant depending on the interpolation depth n.

Table 1.1: Comparison: Number of grid points for level $n = q - d$.

	$d=2$			$d=4$			$d=8$		
n	M	NB	CC	M	NB	CC	M	NB	CC
0	9	1	1	81	1	1	6561	1	1
1	21	5	5	297	9	9	41553	17	17
2	49	17	13	945	49	41	1.9e5	161	145
3	113	49	29	2769	209	137	7.7e5	1121	849
4	257	129	65	7681	769	401	2.8e6	6401	3937
5	577	321	145	20481	2561	1105	9.3e6	31745	15713
6	1281	769	321	52993	7937	2929	3.0e7	141569	56737
7	2817	1793	705	1.3e5	23297	7537	9.1e7	5.8e5	1.9e5

The following graph illustrates the sparse grids of level 0 and level 2 of the three respective grids in two dimensions.

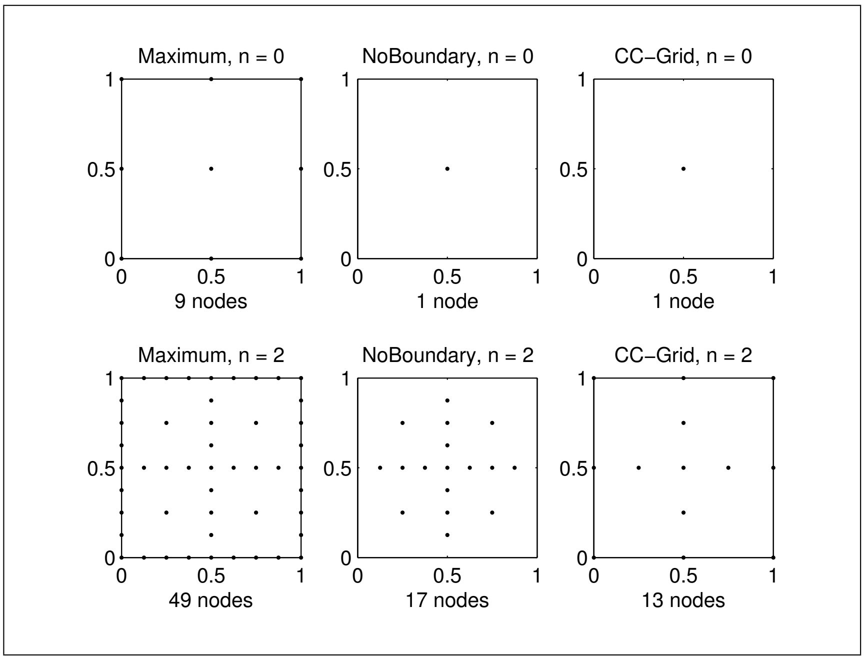

Which piecewise linear interpolation scheme works best?

Although the performance of the three grid types is rather similar for lower- dimensional problems, there are two important points to be mentioned:

- The ClenshawCurtis grid and the NoBoundary grid have just a single node at the lowest interpolation level $n = 0$ (this means that an interpolant of level 0 of these grid types is just a constant function). The Maximum grid has 3^d nodes at the lowest level. Therefore, the Maximum is not well-suited for higher-dimensional problems. For instance, for $d = 10$, already 59049 support nodes would be required to obtain an initial interpolant.

- Since the CC-grid is the most versatile grid working well in both lower and higher dimensions, at this point, the dimension-adaptive algorithms are implemented for this grid type only.

Therefore, for most practical applications, we recommend using the Clenshaw- Curtis grid. Occasionally, the other grid types may perform better by a small factor, as numerical experiments show (try running the demo `spcompare` from the command line or from the Sparse Grid Interpolation demo page).

1.5 Polynomial basis functions

The piecewise multilinear approach can be significantly improved by using higher-order basis functions, such as the Lagrangian characteristic polynomials. The approximation properties of sparse grid interpolation techniques using polynomial basis functions have been studied extensively in [4], where error bounds depending on the smoothness of the function were derived.

From the one-dimensional case, we know that one should not use equidistant nodes for higher-order polynomial interpolation. This directly suggests using Chebyshev-based node distributions. Since an additional requirement of an efficient sparse grid algorithm is the nesting of the sets of nodes, the Chebyshev Gauss-Lobatto nodes are clearly the best choice, and are therefore also suggested in [4]. In this toolbox, this grid type (CGL) is selected by the value "Chebyshev" for the GridType property configurable with the `spset` function.

Since version 5.0, an additional polynomial sparse grid is available, the Gauss-Patterson sparse grid. This grid is based on the abscissae of Gauss-Patterson integration. The Gauss-Patterson formula is a nested quadrature rule that achieves a higher degree of exactness than integration at the Chebyshev Gauss-Lobatto nodes. See [6, 7] for additional details.

For a detailed description of the polynomial basis functions implemented here, please see [3, ch. 3], and the references stated therein. Since version v3.2, the toolbox uses an improved construction algorithm employing the fast discrete cosine transform, see [8].

Accuracy of polynomial interpolation

From the error bounds of the univariate case, the following general error bounds depending on the smoothness of the objective function f are derived in [4]. For $f \in F_d^k$,

$$F_d^k = \{f : [-1,1]^d \to \mathbb{R} \mid D^\beta f \text{ continuous if } \beta_i \le k \,\forall\, i\},$$

with $\beta \in \mathbb{N}_0^d$,

$$D^\beta f = \frac{\partial^{|\beta|} f}{\partial x_1^{\beta_1} \cdots x_d^{\beta_d}},$$

the order of the interpolation error in the maximum norm is given by

$$\left\| f - A_{q,d}(f) \right\|_\infty = \mathcal{O}(N^{-k} \cdot |\log N|^{(k+2)(d+1)+1}),$$

where $A_{q,d}(f)$ denotes the sparse grid interpolant of f, and N denotes the number of grid points of the sparse grids of type CGL. Note that the number of grid points N of $A_{q,d}(f)$ can be computed by `spdim(q-d,d)`.

Number of grid points

The number of grid points of the CGL-grid is identical to the one of the Clenshaw-Curtis (CC) grid. The number of grid points of the Gauss-Patterson grid is identical to the one of the NB grid.

The following graph illustrates the sparse grids of level 0 and level 2 of the CGL-grid in two and three dimensions.

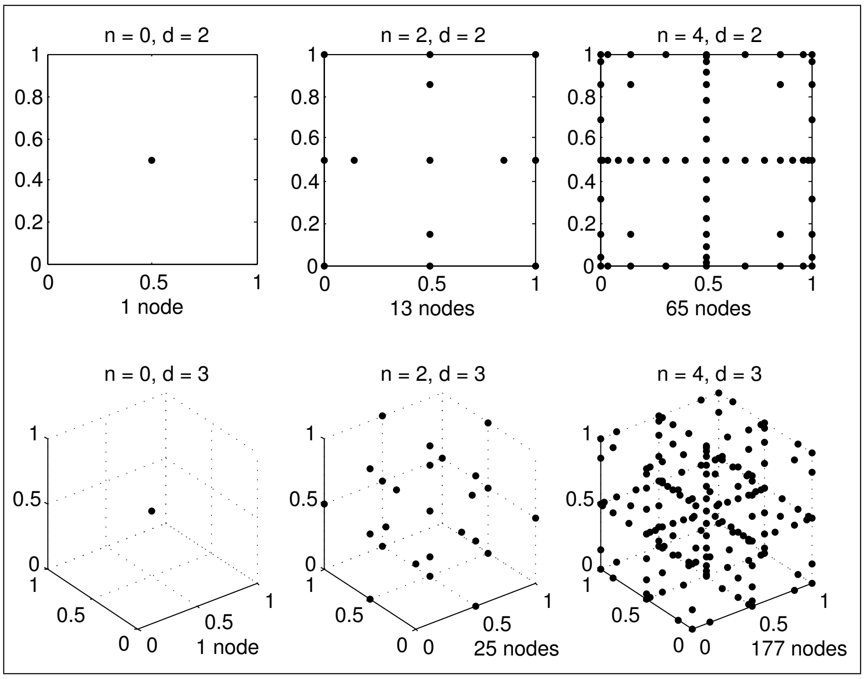

When should I use polynomial rather than linear basis functions?

There is obviously some trade-off between the accuracy gain and the computing time required to construct as well as interpolate the interpolant. Since the higher-order accuracy only becomes effective with increasing number of nodes, we recommend to use the polynomial approach only if the following two conditions are met:

- The objective function to be recovered is known to be very smooth.
- High relative accuracies smaller than 10^{-2} are required.

1.6 Dimensional adaptivity

With the standard sparse grid approach, all dimensions are treated equally, i.e. in each coordinate direction, the number of grid points is equal. The question arises as to whether one can further reduce the computational effort for objective functions where not all input variables carry equal weight. This is especially important in the case of higher-dimensional problems, where this property is often observed. Unfortunately, it is usually not known a priori which variables (or, in this context, dimensions) are important. Therefore, an efficient approach should be able to automatically detect which dimensions are the more or less important ones, without wasting any function evaluations. Hegland [9] and Gerstner and Griebel [10] show that this is indeed possible. They propose an approach to generalize sparse grids such that the number of nodes in each dimension is adaptively adjusted to the problem at hand. Here, the adaptive refinement is not performed in a spatial manner, as it is commonly done in two- and three-dimensional

problems (e.g. [5]), where more support nodes are placed in areas of increased nonlinearity or non-smoothness (this can become impractical in higher dimensions due to the required complex data structure and refinement criteria).

Besides being able to balancing the number of nodes in each coordinate direction, dimension-adaptive sparse grids are capable of automatically detecting separability (or partial separability) encountered in problems with additive (or nearly additive) structure.

The Sparse Grid Interpolation Toolbox includes a powerful dimension-adaptive algorithm based on the approach by Gerstner and Griebel [10], but also includes the significant performance enhancements described in [3, ch. 3]. Applying the dimension-adaptive algorithm is very easy - it can be switched "on" or "off" with a single parameter of the spare grid options structure that can be set with the spset function. Furthermore, the degree of dimensional adaptivity can be chosen as a scalar from the interval [0,1] where 1 stands for greedy (= purely adaptive) and 0 stands for conservative (= standard sparse grid) refinement.

Example

Consider the following quadratic test function:

$$f(x) = [\sum_{i=1}^{d} (x_i - 1)^2] - [\sum_{i=2}^{d} x_i x_{i-1}],$$

implemented in Matlab by the following code:

```
function y = trid(x)
% TRID   Quadratic function with a tridiagonal Hessian.
%    Y = TRID(X)    returns the function value Y for a D-
%    dimensional input vector X.
%
% f(x)=[sum_{i=1}^d (x_i-1)^2] - [sum_{i=2}^d x_ix_{i-1}]
%
% The test function is due to Arnold Neumaier, listed
% on the global optimization Web page at
%    http://www.mat.univie.ac.at/~neum/glopt/

d = length(x);
y = sum((x-1).^2) - sum(x(2:d).*x(1:d-1));
```

The function clearly exhibits additive structure, however, the function is not fully separable due to the second term coupling the variables. Consider the high-dimensional case $d = 100$. A traditional tensor-product approach would completely fail in interpolating a high-dimensional function of this type, since at least 2^{100} nodes are required if an interpolation formula with two nodes is extended to the multivariate case via conventional tensor products. With the dimension-adaptive sparse grid algorithm, however, the structure is automatically detected, and the function is successfully recovered using just $\mathscr{O}(d^2)$ points. For the interpolation domain, we have used $[-d^2, d^2]$ in each dimension.

Using piecewise multilinear basis functions and the Clenshaw-Curtis-Grid, f can be recovered with an estimated relative error of below 0.1 percent (the relative error is given with respect

to the estimated range of the function) with about 27000 function evaluations, as the following code shows.

```
d = 100;
range = repmat([-d^2 d^2],d,1);
options = spset('DimensionAdaptive', 'on', ...
                'DimadaptDegree', 1, ...
                'FunctionArgType', 'vector', ...
                'RelTol', 1e-3, ...
                'MaxPoints', 40000);
z1 = spvals(@trid, d, range, options)
```

```
z1 =
                 vals: {[26993x1 double]}
             gridType: 'Clenshaw-Curtis'
                    d: 100
                range: [100x2 double]
          estRelError: 3.2552e-04
          estAbsError: 9.7656e+04
           fevalRange: [100 300000100]
           minGridVal: [1x100 double]
           maxGridVal: [1x100 double]
              nPoints: 26993
            fevalTime: 5.0122
      surplusCompTime: 0.4784
              indices: [1x1 struct]
             maxLevel: [1x100 double]
        activeIndices: [5149x1 uint32]
       activeIndices2: [5749x1 uint32]
                    E: [1x5749 double]
                    G: [5749x1 double]
                   G2: [5749x1 double]
         maxSetPoints: 6
             dimAdapt: 1
```

Since the objective function is quadratic, we can even approximate the function up to floating point accuracy with the polynomial basis functions and the Chebyshev-Gauss-Lobatto grid:

```
options = spset(options, 'GridType', 'Chebyshev');
z2 = spvals(@trid, d, range, options)
```

```
z2 =
                 vals: {[20201x1 double]}
             gridType: 'Chebyshev'
                    d: 100
                range: [100x2 double]
          estRelError: 2.4835e-17
          estAbsError: 7.4506e-09
           fevalRange: [100 300000100]
```

```
          minGridVal: [1x100 double]
          maxGridVal: [1x100 double]
             nPoints: 20201
           fevalTime: 3.8847
     surplusCompTime: 3.5568
             indices: [1x1 struct]
            maxLevel: [1x100 double]
       activeIndices: [4951x1 uint32]
      activeIndices2: [5151x1 uint32]
                   E: [1x5151 double]
                   G: [5151x1 double]
                  G2: [5151x1 double]
        maxSetPoints: 2
            dimAdapt: 1
```

We can verify the quality of the interpolants by computing the maximum relative error for 100 randomly sampled points (the relative error is computed with respect to the range of the function values that occurred during the sparse grid construction). In this case, the estimated error was too optimistic in the piecewise linear case- however, the relative error for the sampled points is still below 1 percent.

```
% Compute 100 randomly sampled points
p = 100;
rand('state', 0);
x = -d^2 + 2*d^2*rand(p,d);

% Compute exact function values
y = zeros(p,1);
for k = 1:p
  y(k) = trid(x(k,:));
end

% Compute interpolated function values
xcell = num2cell(x,1);
ip1 = spinterp(z1, xcell{:});
ip2 = spinterp(z2, xcell{:});

% Compute relative errors
err_CC = max(abs(y-ip1))/(z1.fevalRange(2)-z1.fevalRange(1))
err_CGL= max(abs(y-ip2))/(z2.fevalRange(2)-z2.fevalRange(1))
```

```
err_CC =
    0.0061
err_CGL =
   1.2716e-14
```

2 Advanced Topics

2.1 Degree of Dimensional Adaptivity

When dealing with high-dimensional problems, an "exhaustive" exploration of the objective function with a standard sparse grid becomes too expensive, since too many points are generated. Therefore, dimension-adaptive sparse grids were introduced that adaptively refine the interpolant with respect to dimensions that are most important, determined by error indicators. However, there is still one problem with this approach: due to the fact that the error indicator is a measure computed from hierarchical surpluses at a few points only, it can happen that the error indicator understimates the actual error with respect to some dimensions. As a consequence, the interpolant may no longer be further refined in these dimensions. In other words, the global convergence property of sparse grid interpolants for the before-mentioned classes of functions is lost for purely greedy dimension-adaptive refinement.

The solution to this problem is to take a "middle ground" approach by introducing an additional parameter, the degree of dimensional adaptivity [10], that lets the user gradually shift emphasis between greedy (= purely adaptive) and conservative (= standard sparse grid) refinement.

Degree balancing approach

Version 5.1 of the toolbox introduces a simple yet very powerful new strategy to the degree of dimensional adaptivity called *degree balancing*, replacing the previous approach based on the interpolation depth described in [3, ch. 3]. The new approach is decribed in the following.

Definition of the degree of dimensional adaptivity

As a preliminary, we define the actual degree of dimensional adaptivity as the ratio

$$r = n_{\text{AdaptPoints}}/n_{\text{TotalPoints}},$$

where $n_{\text{AdaptPoints}}$ denotes the number of points added according to a greedy, purely dimension-adaptive refinement rule, and $n_{\text{TotalPoints}}$ denotes the total number of sparse grid points. We assume that the remaining points $n_{\text{TotalPoints}} - n_{\text{AdaptPoints}}$ are constructed by the standard sparse grid refinement rule.

Specifying the target dimensional adaptivity degree

The user may specify a target degree of dimensionsonal adaptivity using the DimadaptDegree parameter of the sparse grid options structure.

Refinement rule

During interpolant construction, index sets of both a greedy dimension-adaptive (adaptive refinement rule) and a regular sparse grid interpolant (regular refinement rule) are maintained. Then, at each step of the construction algorithm, the ratio *r* is computed, representing the current degree of dimensional adaptivity. Now, if the target rate is smaller than the current rate *r*, points corresponding to an index set maintained by the adaptive rule are added to the interpolant, otherwise, points according to the regular rule are added. Thus, the generated interpolant will have a degree of dimensional adaptivity close to the target degree (it is "balanced" around the target degree).

Benefits of the new approach

The new degree balancing approach has the following benefits:

- Transparent, easy to understand definition of the degree of dimensional adaptivity.
- Works independently of the problem dimension.
- The target degree of adaptivity can be adjusted interactively at any time (see example below).

Examples

Comparison of different degrees of dimensional adaptivity

Let us use Branin's function to illustrate how the degree of dimensional adaptivity affects the grid construction.

```
fun = inline(['(5/pi*x-5.1/(4*pi^2)*x.^2+y-6).^2␣+␣' ...
              '10*(1-1/(8*pi))*cos(x)+10']);
d = 2;
range = [-5, 10; 0, 15];
```

The code snippet below generates sparse grid interpolants for target degrees of dimensional adaptivity 0, 0.5, and 1, i.e., about 0%, 50%, or 100% of the grid points are generated by the greedy, error-indicator based refinement rule. For each interpolant, we set the minimum and maximum number of points to 65 in order to get an interpolant with close to that number of points.

```
options = spset('GridType', 'Chebyshev', 'DimensionAdaptive', 'on', ...
  'MinPoints', 65, 'MaxPoints', 65, 'Vectorized', 'on', 'KeepGrid', 'on');
z1 = spvals(fun, d, range, spset(options, 'DimadaptDegree', 0));
z2 = spvals(fun, d, range, spset(options, 'DimadaptDegree', 0.5));
z3 = spvals(fun, d, range, spset(options, 'DimadaptDegree', 1));
```

We can check how closely the actual degrees of dimensional adaptivity meet the target rates:

```
disp(sprintf(...
  'Degree␣of␣dimensional␣adaptivity:␣z1:␣%.2f,␣z2:␣%.2f,␣z3:␣%.2f', ...
  z1.dimadaptDegree, z2.dimadaptDegree, z3.dimadaptDegree));
```

```
Degree of dimensional adaptivity: z1: 0.00, z2: 0.52, z3: 0.99
```

The following plot compares the generated grids and subgrid indices.

```
z = {z1, z2, z3};
for k = 1:3
  subplot(2,3,k);
  plot(z{k}.grid{1}(:,1), z{k}.grid{1}(:,2), 'k.');
  axis([range(1,:), range(2,:)]);
  axis square;
  subplot(2,3,3+k);
  plotindices(z{k});
  title(sprintf('z%d,␣degree:␣%2.0f%%', k, 100*z{k}.dimadaptDegree))
  axis([0,8,0,6]);
end
```

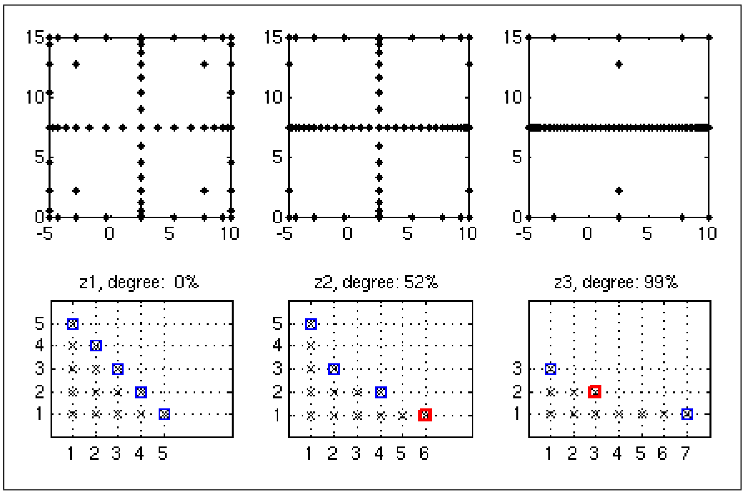

Adjusting the adaptivity degree during interpolant construction

Suppose we have created a dimension-adaptive interpolant with purely greedy refinement to achieve a target estimated error of 10^{-3}.

```
options = spset('GridType', 'Chebyshev', 'DimensionAdaptive', 'on', ...
  'RelTol', 1e-3, 'Vectorized', 'on', 'KeepGrid', 'on', 'MinPoints', 30);
z = spvals(fun, d, range, spset(options, 'DimadaptDegree', 1));
```

We find that it took 45 points to achieve this estimated accuracy:

```
z.nPoints
z.estRelError
```

```
ans =
          45
ans =
   6.3274e-07
```

We are happy with the achieved accuracy, but in order to be more comfortable with the result, we would like to add some additional points according to the standard sparse grid refinement rule. We thus change the degree of dimensional adaptivity to 0 (=conservative, non-adaptive refinement), and add another approx. 20 points to the interpolant.

```
z = spvals(fun, d, range, spset(options, 'DimadaptDegree', 0, ...
  'MinPoints', z.nPoints + 20, 'PrevResults', z));
```

We can now check if the estimated error is still satisfied:

```
z.estRelError
```

```
ans =
   6.3244e-07
```

The actual degree of dimensional adaptivity of the interpolant reflects our refinement by the two different rules. 44 points (64%) out of the total points of now 69 were added according to the dimension-adaptive refinement rule.

```
z.nPoints
z.dimadaptDegree
```

```
ans =
          69
ans =
    0.6377
```

2.2 Multiple output variables

Real-world problems usually have more than a single output argument. Sparse grids are very well-suited to deal with such kind of problems, since the regular structure allows to construct good approximations for multiple output variables at once.

The sparse grid interpolation package is designed to make dealing with multiple output arguments easy, as the following example demonstrates.

Example

Consider the following simple test function with multiple output arguments:

```
function [out1, out2, out3, out4] = multiout(x,y)
% MULTIOUT   Test function with four output arguments
out1 = (x+y).^2;
out2 = 1./exp(1+(x-0.5).^2+(y-0.3).^2);
out3 = sin(pi*(2-x))+cos(pi*(1-y));
out4 = sinh(4.*(x-0.5));
```

`spvals` will automatically compute interpolants with respect to all four output variables if the number of output variables is specified in the sparse grid `OPTIONS` structure:

```
nout = 4;
options = spset('NumberOfOutputs', nout, 'Vectorized', 'on');
z = spvals(@multiout, 2, [], options)
```

```
z =
                 vals: {4x1 cell}
             gridType: 'Clenshaw-Curtis'
                    d: 2
                range: []
             maxLevel: 5
          estRelError: 0.0034
          estAbsError: 0.0249
           fevalRange: [4x2 double]
           minGridVal: [4x2 double]
           maxGridVal: [4x2 double]
              nPoints: 145
            fevalTime: 0.0392
      surplusCompTime: 0.0129
              indices: [1x1 struct]
```

Note that the output parameters of the objective function must all be scalar. The number of outputs `nout` specified in the options structure may be smaller than the actual number of outputs. In this case, interpolants are constructed only with respect to the first `nout` arguments.

To compute interpolated values, the desired output argument must now be specified. This is done by adding an additional field `selectOutput` to the structure `z` prior to the call to the `spinterp` function. The following code plots the four computed interpolants:

```
for k = 1:nout
  z.selectOutput = k;
  subplot(2,2,k);
  ezmesh(@(x,y) spinterp(z,x,y), [0 1]);
  axis square;
  title(['out' num2str(k)]);
end
```

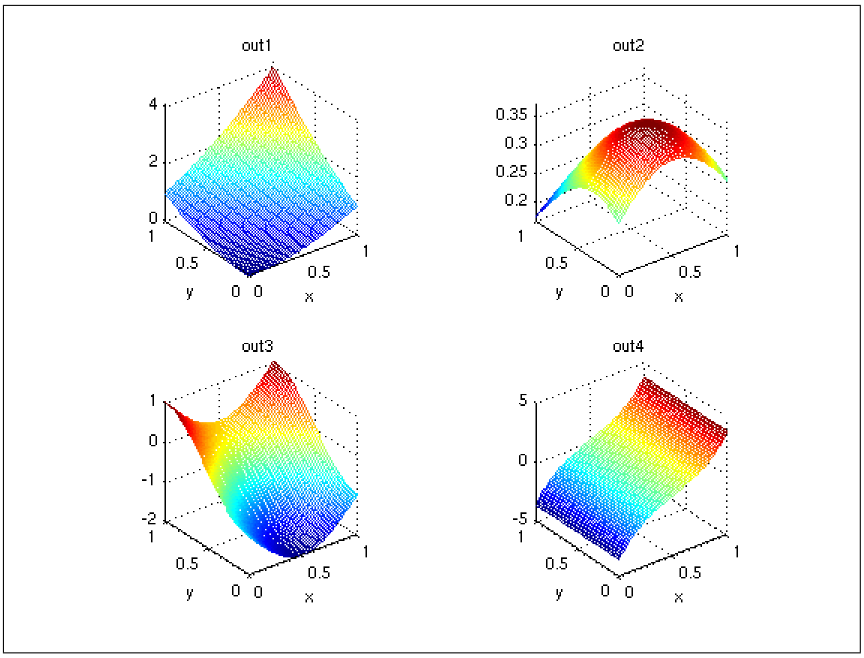

An additional example of using multiple output arguments with spvals is given by the demo `spdemovarout` available at the command line or from the demos page.

2.3 Derivatives

One of the primary purposes of sparse grid interpolation is the construction of surrogate functions for local or global optimization. While some optimization methods work well using function values only, many efficient algorithms exist that require computation of the gradient. With the Sparse Grid Interpolation Toolbox, it is possible to obtain gradients of the interpolants directly – up to floating point accuracy – as opposed to approximating them numerically, such as by finite differences. This is demonstrated in the following.

How to obtain the derivatives?

Computing derivatives is extremely simple. One just calls the method `spinterp`, but instead of a single left-hand argument, one uses the syntax `[ip,ipgrad] = spinterp(`$z, x_1, \ldots, x_n$`)`. This returns not only the function value at the point(s) $(x_1, \ldots, x_n)$, but also the complete gradient vector(s). Please see the reference page of `spinterp` for further details (or the examples provided below).

It is important to note that the entire procedure of computing the hierarchical surpluses of the sparse grid interpolant with `spvals` remains the same, regardless of whether derivatives are desired or not. Also, purging of the interpolant (see `sppurge`) can be performed in the usual manner if desired. This makes using derivative information very flexible, and it can be decided ad-hoc, well after interpolant construction (for example, when different optimization algorithms are applied), whether to use derivatives or not.

Furthermore, the derivatives computed by `spinterp` are **not** additional approximations of the derivatives of the original function, but rather, the **exact** derivatives of the interpolant (up to floating point accuracy). The advantage of this approach is that **no additional memory is required** to store derivative information. The derivatives are computed on-the-fly by efficient algorithms.

Derivatives of piecewise multilinear interpolants

We start with derivatives of piecewise multilinear Clenshaw-Curtis Sparse Grid Interpolants. Deriving a piecewise linear function leads to piecewise constant derivatives with respect to the variable that the function is differentiated for. Since the interpolant is non-differentiable at the kinks, the left-sided (or right-sided) derivative is computed at these points only. The following example in two dimensions illustrates the nature of the derivatives.

First, we define the example objective function. We also define its derivatives (this is only for comparison to give an idea of the quality of the computed derivatives).

```
% Define function and its derivatives
f = inline('1./(cos(2*x).^2 + sin(y).^2 + 1) + 0.2*y');
fdx = inline('4*cos(2*x).*sin(2*x)./(cos(2*x).^2 + sin(y).^2 + 1).^2');
fdy = inline('-2*cos(y).*sin(y)./(cos(2*x).^2 + sin(y).^2 + 1).^2 + 0.2');
```

Next, we compute the interpolant. In this case, using the regular Clenshaw- Curtis sparse grid. We limit the sparse grid depth to 4 here (i.e., $A_{q,d}(f) = A_{4+2,2}(f)$ is computed).

```
d = 2;
maxDepth = 4;
options = spset('Vectorized','on','SparseIndices','off', ...
  'MaxDepth', maxDepth);
z = spvals(f,d,[],options);
```

```
Warning: MaxDepth = 4 reached before accuracies RelTol = 0.01 or
AbsTol = 1e-06 were achieved.
The current estimated relative accuracy is 0.02877.
```

Our aim here is to plot the derivatives. Therefore, we define a suitable grid, and set up an array for the vectorized evaluation of the interpolant and its derivatives. We set up the grid such that the jumps of the derivative will be clearly visible.

```
np = 2^double(z.maxLevel)+1;
x = linspace(0,1,np);
xstep = zeros(1,(np-1)*2);
xstep(1:2:end-1) = x(1:end-1);
xstep(2:2:end) = x(2:end) - eps;
[x,y] = ndgrid(xstep);
```

Next, we evaluate the interpolant at the grid points. As result, `ipgrad` will contain an array of the shape of the input arrays `x` and `y`, with the difference that it is a cell array instead of a double array, where each cell contains the entire gradient at the point of the corresponding array entry of x and y.

```
[ip,ipgrad] = spinterp(z,x,y);
```

For plotting, it is convenient to convert the data returned as a cell array back to a double array. This is achieved by the following statements. We extract the derivatives with respect to y here.

```
% Convert returned cell array to matrix
ipgradmat = cell2mat(ipgrad);
% Get the derivatives with respect to y
ipdy = ipgradmat(2:d:end,:);
```

Similarly, we can get all derivatives with respect to x with the command

```
ipdx = ipgradmat(1:d:end,:);
```

This approach of transforming the cell array to a double array can be easily extended to other dimensions.

Finally, we plot the obtained derivatives next to the exact derivatives.

```
subplot(1,2,1,'align');
surf(x,y,fdy(x,y));
view(250,50); xlabel('x'); ylabel('y'); light;
title('Exact:␣{\partial}f␣/␣{\partial}y');
subplot(1,2,2,'align');
surf(x,y,ipdy);
view(250,50); xlabel('x'); ylabel('y'); light;
title('Approx.:␣{\partial}A^{CC}_{6,2}(f)␣/␣{\partial}y␣(piecewise␣const.
w.r.t.␣y)');
```

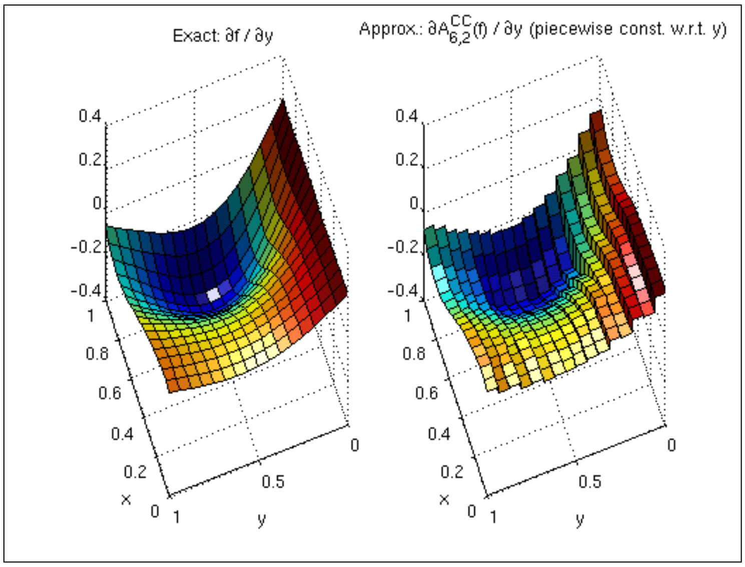

Augmented derivatives to achieve continuity

Obtaining the derivatives of the interpolant is usually not the primary goal, but rather, serves a secondary purpose. For instance, in an optimization procedure, one is not interested in the

derivatives per se. Instead, the gradient vector enters an iterative procedure to achieve the primary goal, which is to numerically compute a local optimizer. Unfortunately, the discontinuous derivatives of a piecewise multilinear interpolant have a serious drawback: the first order optimality condition grad $f = 0$ cannot be fulfilled. Instead, the sign of the gradient components will oscillate about the optimizer of the continuous interpolant, resulting in slow convergence.

To overcome this limitation, the Sparse Grid Interpolation Toolbox offers a powerful alternative to computing the exact derivatives of a piecewise multilinear interpolant. By setting a simple flag, augmented derivatives can be computed that artificially enforce continuity. This is achieved by linear interpolation with respect to the derived variable.

Let us consider an example (we use the same test function and interpolant from above).

First, we re-define the evaluation grid (there are no more jumps to emphasize).

```
np = 2^double(z.maxLevel+1)+1;
x = linspace(0,1,np);
[x,y] = ndgrid(x);
```

Prior to evaluating the interpolant, we set the flag `continuousDerivatives = 'on'`.

```
z.continuousDerivatives = 'on';
```

Computing interpolated values and gradients of the sparse grid interpolant is done as before with the command

```
[ip,ipgrad] = spinterp(z,x,y);
```

Finally, we generate the plot. Compare the plot to the previous one. Note that the derivatives are now continuous.

```
% Convert returned cell array to matrix
ipgradmat = cell2mat(ipgrad);

% Get the derivatives with respect to y
ipdy = ipgradmat(2:d:end,:);

% Plot exact derivatives and derivatives of interpolant
subplot(1,2,1,'align');
surf(x,y,fdy(x,y));
view(250,50); xlabel('x'); ylabel('y'); light;
title('Exact:␣{\partial}f␣/␣{\partial}y');
subplot(1,2,2,'align');
surf(x,y,ipdy);
view(250,50); xlabel('x'); ylabel('y'); light;
title('Approx.:␣{\partial}A^{CC}_{6,2}(f)␣/␣{\partial}y');
```

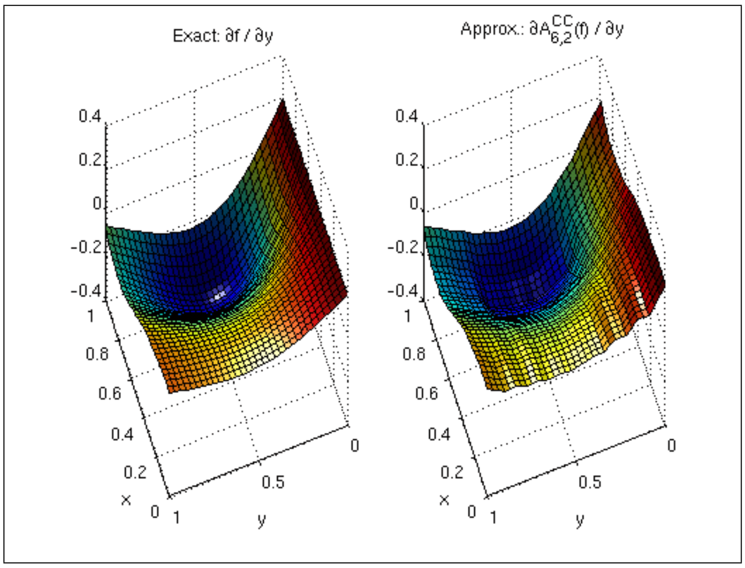

To conclude this section: If piecewise multilinear sparse grid interpolants (the Clenshaw-Curtis grid) are used, augmented derivatives can help improving efficiency when solving optimization problems with methods that require computation of the gradient.

Derivatives of polynomial interpolants

The Chebyshev-Gauss-Lobatto (CGL) sparse grid uses globally defined polynomial basis functions. These basis functions are infinitely smooth, and thus, the derivatives are infinitely smooth, too. The Sparse Grid Interpolation Toolbox offers efficient algorithms involving barycentric interpolation and the discrete cosine transform to compute gradients, with excellent numerical stability.

Consider the following example. Using the test function from above, we compute a CGL-type sparse grid interpolant (again with `maxDepth = 4`).

```
maxDepth = 4;
options = spset('Vectorized','on','SparseIndices','off', ...
  'MaxDepth', maxDepth, 'GridType', 'Chebyshev');
z = spvals(f,d,[],options);
```

```
Warning: MaxDepth = 4 reached before accuracies RelTol = 0.01 or
AbsTol = 1e-06 were achieved.
The current estimated relative accuracy is 0.020306.
```

The remaining code evaluates the interpolant and its derivatives at the full grid and creates the plot, just as above.

```
[ip,ipgrad] = spinterp(z,x,y);
ipgradmat = cell2mat(ipgrad);
ipdy = ipgradmat(2:d:end,:);
subplot(1,2,1,'align');
```

```
surf(x,y,fdy(x,y));
view(250,50); xlabel('x'); ylabel('y'); light;
title('Exact:␣{\partial}f␣/␣{\partial}y');
subplot(1,2,2,'align');
surf(x,y,ipdy);
view(250,50); xlabel('x'); ylabel('y'); light;
title('Approx.:␣{\partial}A^{CC}_{6,2}(f)␣/␣{\partial}y');
```

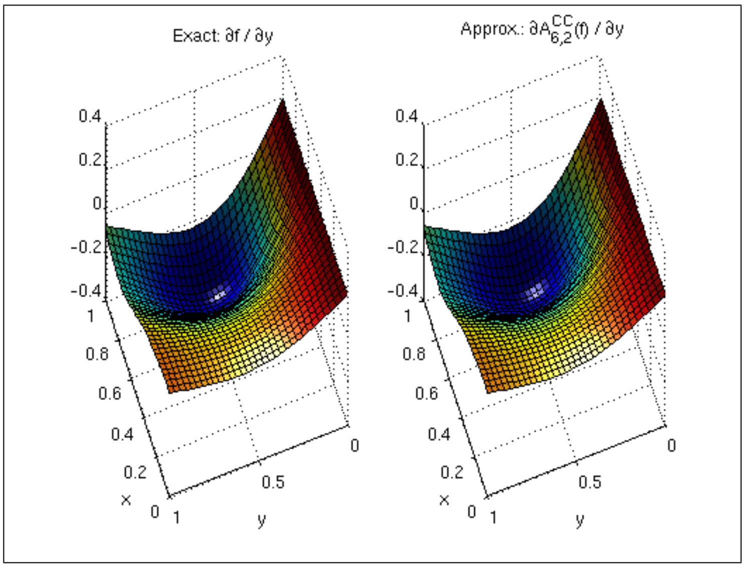

Approximation quality

Although we see the main application of computing derivatives of sparse grid interpolants in obtaining gradients during an optimization algorithm, it is interesting to investigate the approximation quality with respect to the derivatives of the original function.

This is illustrated by the example `spcomparederiv.m` that plots an approximation of the error in the maximum norm by computing the maximum absolute error of the derivatives for the six test functions of Genz (see testderivatives.m) at 100 randomly sampled points. The plot presented below is for dimension $d = 3$.

```
% Reset random number generator (to generate reproducible results)
rand('state',0);
% Run the demo.
spcomparederiv;
```

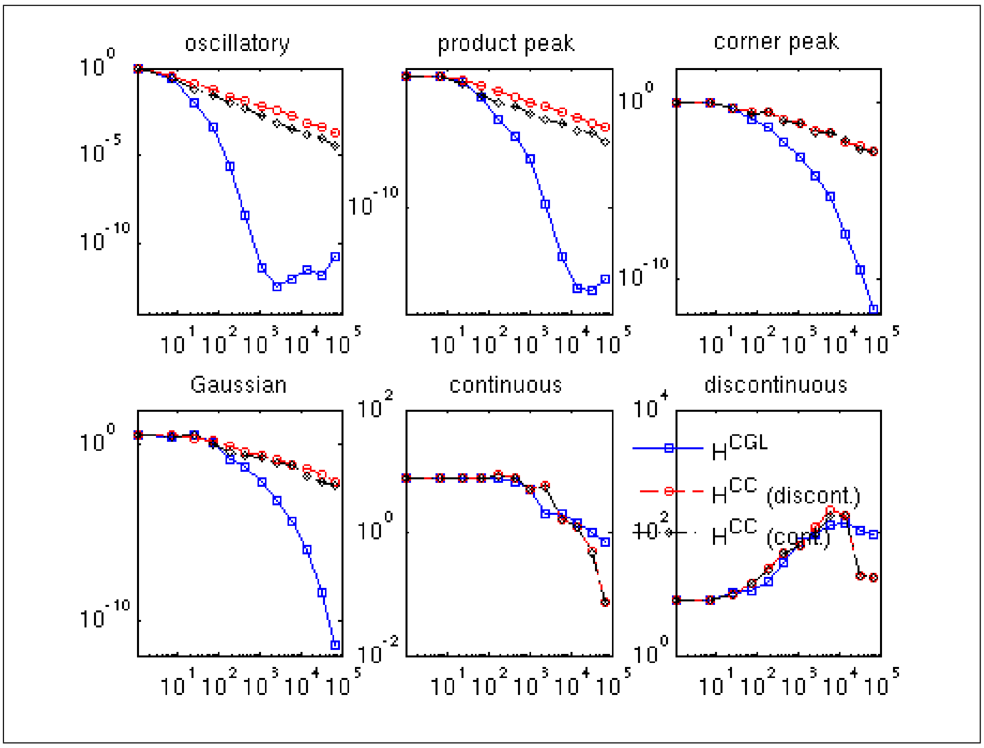

The legend indicates the three types of derivatives: discontinuous (H^{CC} grid), augmented continuous (H^{CC} grid), and smooth (H^{CGL} grid).

Remark: For the original functions labeled labelled'continuous' and 'discontinuous', single-sided derivatives are computed at the points where the function is not continuously differentiable. Note that the approximations of the derivatives of both of these two functions do not converge, for the following reasons:

- Since the **discontinuous** function itself cannot be approximated by a continuous sparse grid interpolant in the first place, the approximations of the derivatives will not converge, either.
- What is less obvious is that the derivatives of the **continuous** function **cannot** be successfully approximated for the whole considered box, either. Although the plot labeled 'continuous' appears to suggest slow convergence, **convergence in the maximum norm is not achieved** in regions close to the kink(s). However, this/these region(s) becomes smaller with increasing number of support nodes. The decreasing size of the non- converging region(s) close to the kink(s) explain(s) the decay of the absolute error in the plot: It becomes less likely that any of the 100 randomly sampled points are placed here.

Computational cost

Cost for the piecewise multilinear Clenshaw-Curtis sparse grid

The gradient vector of the piecewise multilinear Clenshaw-Curtis sparse grid interpolant can be obtained at a very small additional cost. The following two plots illustrate that this additional cost for computing either the exact derivatives or the augmented continuous derivatives amounts to just a small factor that is almost independent of the problem dimension.

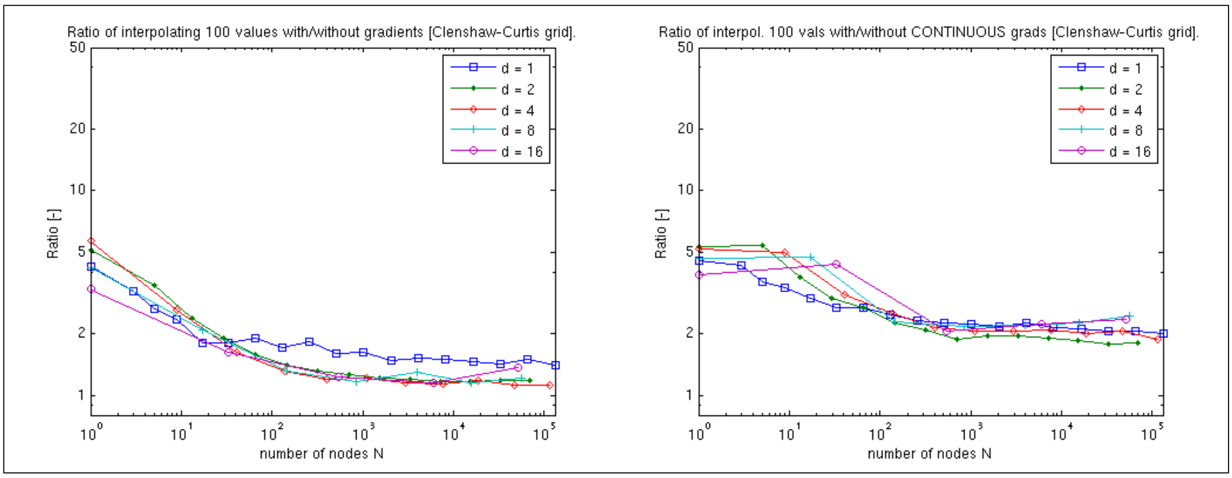

Cost for the polynomial Chebyshev-Gauss-Lobatto sparse grid

Due to the more sophisticated algorithms required in the polynomial case, the additional cost of computing the gradients is considerably higher compared to a mere interpolation of function values. However, as the dimension increases, the additional cost decreases, as fewer subgrids will require a derivative computation (many subgrids are lower-dimensional than the final interpolant, and thus, must not be differentiated with respect to the dimensions that are omitted).

Thus, the performance is very competitive especially in higher dimensions. For instance, consider applying numerical differentiation instead (which you can easily do alternatively). This would require $d+1$ interpolant evaluations to compute the gradient if single-sided differences are used, or even $2d+1$ if the more accurate centered difference formula is used.

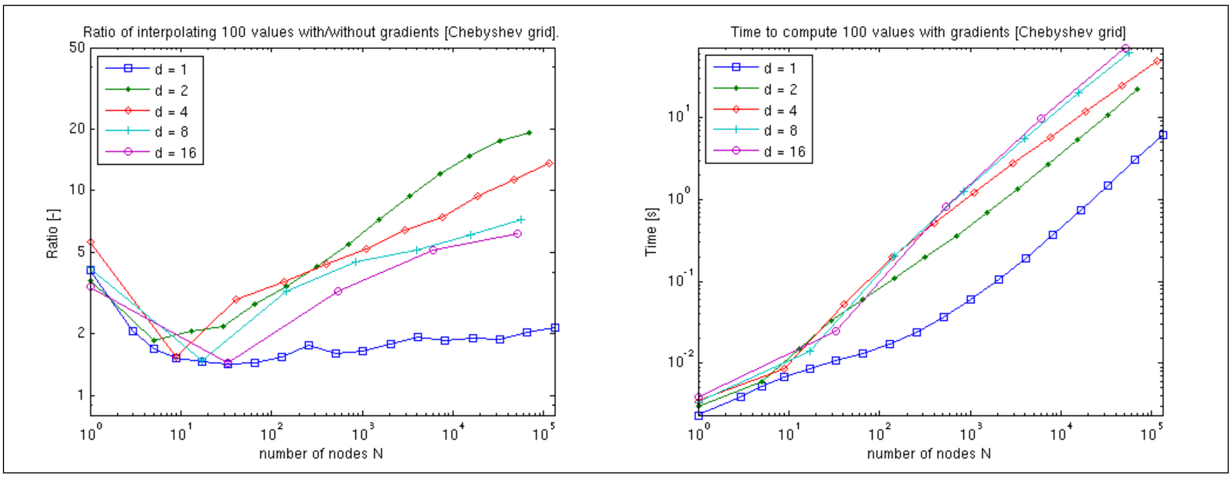

Remark: We produced the previous graphs with the example function timespderiv.m, which is included in the toolbox (see demos). The test was performed using Matlab R14 SP3 running on a Linux 2.6.15 machine equipped with one AMD Athlon 1.5 GHz CPU and 512 MB of memory.

2.4 Numerical Integration (Quadrature)

Once you have computed a sparse grid interpolant of an objective function, you can compute the integral value of it for the given range. You can do this for any grid type, and for both regular and dimension-adaptive sparse grid interpolants by simply calling the `spquad` function. A couple of examples are provided below.

Integration of regular sparse grid interpolants

Consider the task of integrating the test function

$$f(\mathbf{x}) = (1+1/d)^d \prod_{i=1}^{d} (x_i)^{1/d}$$

for the domain $[0,1]^d$, and $d = 5$. The exact value of the integral is 1. We reproduce the results of Table 1 for the columns labelled "Trapez", "Clenshaw", and "Gauss-Patterson" from [7]. Note that the grid type Clenshaw-Curtis and Chebyshev of the Sparse Grid Interpolation Toolbox correspond to the sparse grids based on the Trapez rule and the Clenshaw Curtis rule in the paper, respectively.

Define the objective function, dimension, and maximum depth:

```
d = 5;
maxDepth = 6;
f = @(x) (1+1/d)^d * prod(x)^(1/d);
```

Compute integral for increasing sparse grid depth, and generate the results table:

```
warning('off', 'MATLAB:spinterp:insufficientDepth');
z = cell(3,1);
quadr = zeros(3,1);
disp('␣Clenshaw-Curtis␣|␣␣␣␣Chebyshev␣␣␣␣␣|␣Gauss-Patterson');
disp('␣points␣␣␣␣error␣|␣points␣␣␣␣␣error␣|␣points␣␣␣␣␣error');
for k = 0:maxDepth
  options = spset('MinDepth', k, 'MaxDepth', k, ...
                  'FunctionArgType', 'vector');

        % Compute integral values with Clenshaw-Curtis grid
        options = spset(options, 'GridType', 'Clenshaw-Curtis', ...
                  'PrevResults', z{1});
  z{1} = spvals(f,d,[],options);
  quadr(1) = spquad(z{1});

        % Compute integral values with Chebyshev grid
        options = spset(options, 'GridType', 'Chebyshev', ...
                  'PrevResults', z{2});
  z{2} = spvals(f,d,[],options);
  quadr(2) = spquad(z{2});

        % Compute integral values with Gauss-Patterson grid
        options = spset(options, 'GridType', 'Gauss-Patterson', ...
                  'PrevResults', z{3});
  z{3} = spvals(f,d,[],options);
  quadr(3) = spquad(z{3});

  % Results output
  disp(sprintf('%5d␣␣%8.3e␣|␣%5d␣␣%8.3e␣|␣%5d␣␣%8.3e', ...
          z{1}.nPoints, abs(1-quadr(1)), ...
                z{2}.nPoints, abs(1-quadr(2)), ...
                z{3}.nPoints, abs(1-quadr(3))));
end
```

```
 Clenshaw-Curtis |    Chebyshev      | Gauss-Patterson
 points    error | points      error | points      error
     1  2.442e-01 |     1  2.442e-01 |      1  2.442e-01
    11  1.080e+00 |    11  6.385e-01 |     11  8.936e-03
    61  7.578e-02 |    61  1.441e-01 |     71  8.073e-04
   241  2.864e-01 |   241  1.237e-01 |    351  2.070e-04
   801  1.079e-01 |   801  6.650e-03 |   1471  2.256e-05
  2433  8.001e-02 |  2433  1.060e-02 |   5503  1.420e-06
  6993  5.030e-02 |  6993  1.743e-03 |  18943  3.437e-09
```

Integration of dimension-adaptive sparse grid interpolants

To illustrate a higher-dimensional, dimension-adpative case, we consider the absorption problem from W. Morokoff, R. Caflisch, "Quasi-monte carlo integration", J. Comp. Phys. 122, pp. 218-230, 1995, given by the integral equation

$$y(x) = \int_x^1 \gamma y(x')dx' + x.$$

The exact solution of this equation is given by

$$y(x) = \frac{1}{\gamma}(1-(1-\gamma)\exp(\gamma(1-x))).$$

Two alternate representations are given in the paper, the first being an infinite integral with an integrand with a jump, and the second one with a smooth integrand.

The sparse grid method does not work well for the first representation, since it is a non-smooth function where the discontinuities are not parallel to the coordinate directions (see S. Dirnsdorfer, "Numerical Quadrature on Sparse Grids", Diploma Thesis, TU Munich, 2000). However, in case of the second representation, very accurate results can be computed using the dimension- adaptive approach, as shown below.

We define the integrand of the absorption problem as follows. The optional parameter named SMOOTH indicates which of the two representation should be used.

```
function y = absorb(varargin)
% ABSORB(R1,...,RD,GAMMA,X,SMOOTH)   Finite sum integrand of
%   the integrral representation of the absorption problem
%   paper W. Morokoff, R. Caflisch, "Quasi-monte carlo
%   integration", J. Comp. Phys. 122, pp. 218-230, 1995.

d = length(varargin) - 3;
gamma = varargin{end-2};
x = varargin{end-1};
smooth = varargin{end};

% Compute F as in paper
if ~smooth
  % First representation with jump
  phi = @(z) (1 .* (z >= 0));  % Heaviside function
  d = d - 1;
  sumR = cell(d+1,1);
  sumR{1} = varargin{1};
  for k = 2:d+1
    sumR{k} = sumR{k-1} + varargin{k};
  end
  F = @(n) (gamma^n * phi(1-x-sumR{n}) ...
     .* phi(sumR{n+1}-(1-x)));
else
```

```
  % Second, smooth representation
  prodR1 = cell(d,1);
  prodR1{1} = ones(size(varargin{1}));
  for k = 2:d
    prodR1{k} = prodR1{k-1};
    for l = 1:k-1
      prodR1{k} = prodR1{k} .* varargin{l};
    end
  end
  prodR2 = cell(d,1);
  prodR2{1} = varargin{1};
  for k = 2:d
    prodR2{k} = prodR2{k-1} .* varargin{k};
  end
  F = @(n) (gamma^n * (1 - x)^n * ...
    prodR1{n} .* (1 - (1 - x) * prodR2{n}));
end

% Compute integrand value(s) (finite sum)
y = zeros(size(varargin{1}));
for n = 1:d
  y = y + F(n);
end
```

The following loop computes increasingly accurate approximations to the solution of the absorption problem with $d = 20$, $\gamma = 0.5$, and $x = 0$ by computing a dimension-adaptive polynomial interpolant of the smooth integrand which is then integrated using the spquad function. For comparison, we also compute the integral using crude Monte Carlo (MC) with the same number of points.

```
gamma = 0.5; x = 0; d = 20;

% Show exact solution
I_exact = 1/gamma - (1-gamma)/gamma*exp(gamma*(1-x))

options = spset('DimensionAdaptive','on', 'DimadaptDegree', 1, ...
                'GridType', 'Chebyshev', 'Vectorized', 'on');
Nmax = 50000;
N = 2*d;
z = [];

warning('off', 'MATLAB:spinterp:maxPointsReached');
while N <= Nmax
  % Compute integral via sparse grid
  spoptions = spset(options, 'MinPoints', N, ...
    'MaxPoints', N, 'PrevResults', z);
  z = spvals(@absorb, d, [], spoptions, gamma, x, true);
```

```
  e1 = abs(I_exact - spquad(z));

  % Compute integral via MC (error average for 10 runs)
  e2 = 0;
  for k = 1:10
    p = rand(z.nPoints,d);
    p = num2cell(p,1);
    I = sum(absorb(p{:}, gamma, x, true)) / double(z.nPoints);
    e2 = e2 + abs(I_exact - I);
  end
  e2 = e2 / 10;

  disp([' points: ' sprintf('%5d', z.nPoints) ...
      ' | error (CGL): ' sprintf('%9.3e',e1) ...
      ' | error (MC): ' sprintf('%9.3e',e2)]);
  N = round(z.nPoints .* 2);
end
warning('on', 'MATLAB:spinterp:maxPointsReached');
```

```
I_exact =
    0.3513

 points:    41 | error (CGL): 4.622e-04 | error (MC): 1.298e-02
 points:    87 | error (CGL): 5.606e-06 | error (MC): 6.008e-03
 points:   177 | error (CGL): 6.010e-07 | error (MC): 7.791e-03
 points:   367 | error (CGL): 1.566e-07 | error (MC): 3.442e-03
 points:   739 | error (CGL): 3.893e-08 | error (MC): 4.761e-03
 points:  1531 | error (CGL): 2.461e-08 | error (MC): 1.895e-03
 points:  3085 | error (CGL): 1.061e-09 | error (MC): 1.036e-03
 points:  6181 | error (CGL): 2.750e-09 | error (MC): 6.147e-04
 points: 12393 | error (CGL): 1.335e-09 | error (MC): 6.609e-04
 points: 24795 | error (CGL): 3.006e-10 | error (MC): 5.420e-04
 points: 49739 | error (CGL): 1.791e-10 | error (MC): 3.305e-04
```

2.5 Optimization

Once a sparse grid interpolant providing a surrogate function or meta-model of an expensive to evaluate model has been obtained, a very common task to be performed is often a search for local/global minimizers or maximizers. Since version 4.0 of the toolbox, several efficient optimization methods are available to perform this task. Furthermore, it is easy to use third-party optimization codes on sparse grid interpolants.

Available algorithms

The following optimization algorithms are included with the toolbox:

- `spcgsearch` - suitable for optimizing polynomial sparse grid interpolants.
- `spcompsearch` - suitable for optimizing piecewise linear sparse grid interpolants.
- `spfminsearch` - works for all types of interpolants, but usually less efficient than `spcompsearch` or `spcgsearch`.
- `spmultistart` - a multiple random start search method that uses any of the above methods for the local searches.

Many parameters of these algorithms can be configured with an options structure created with the `spoptimset` function.

Optimizing piecewise linear interpolants

We consider a simple algebraic test function f, the well-known six-hump camel back function. Here, we visualize f slightly shifted and in logarithmic scaling to cleary show the 6 minima. Two minima are global, indicated by the red triangle.

```
f = @(x,y) (4-2.1.*x.^2+x.^4./3).*x.^2+x.*y+(-4+4.*y.^2).*y.^2;
ezcontour(@(x,y) log(2+f(x,y)), [-3 3 -2 2], 51);
hold on;
plot([ 0.08984201  -0.08984201], [-0.71265640   0.71265640], 'r^');
```

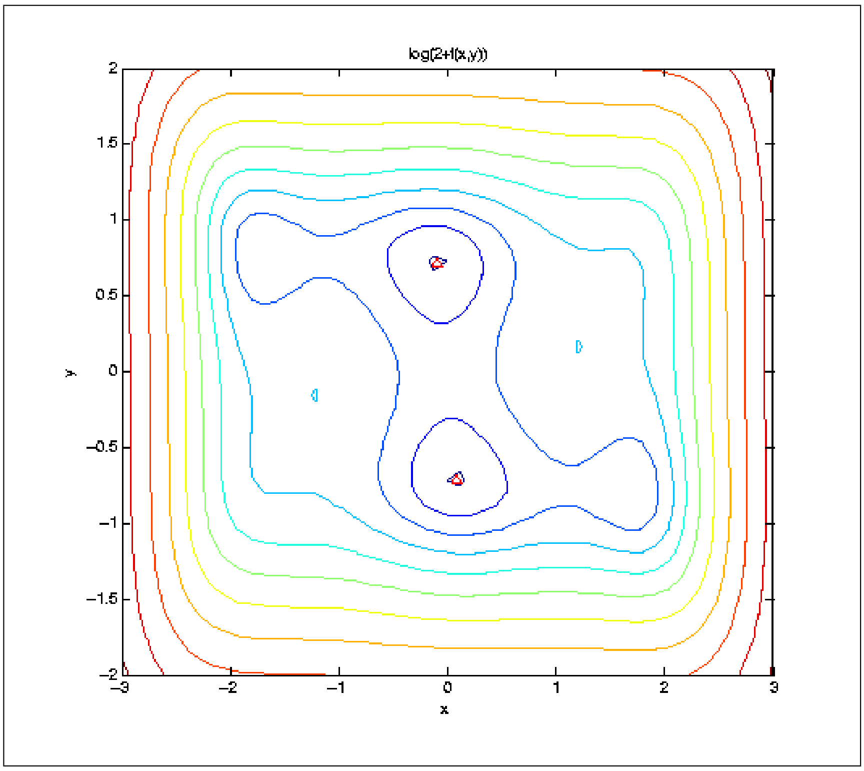

We construct a sequence of piecewise linear interpolants, and optimize them by the spcompsearch function in each step. Here, we use a maximum of $N = 705$ points, as used by the Clenshaw-Curtis grid of level $n_{\max} = 5$, to approximate the (global) minimum.

```
nmax = 7;
z = [];
range = [-3 3; -2 2];
f_exact = -1.0316284535

warning('off', 'MATLAB:spinterp:insufficientDepth');
tic;
for n = 1:nmax
  spoptions = spset('MinDepth', n, 'MaxDepth', n, 'PrevResults', z, ...
    'KeepFunctionValues', 'on');
  z = spvals(f,2,range,spoptions);
  [xopt, fval, exitflag, output] = spcompsearch(z,range);
  disp([' grid pnts: ' sprintf('%3d', z.nPoints) ...
        ' | optim fevals: ' sprintf('%3d', output.nFEvals) ...
        ' | fval: ' sprintf('%+5f', fval) ...
        ' | abs. error: ' num2str(abs(f_exact-fval))]);
end
toc;
warning('on', 'MATLAB:spinterp:insufficientDepth');
```

```
f_exact =
   -1.0316

 grid pnts:   5 | optim fevals:   8 | fval: +0.000000 | abs. error: 1.0316
 grid pnts:  13 | optim fevals:   8 | fval: +0.000000 | abs. error: 1.0316
 grid pnts:  29 | optim fevals:  12 | fval: -0.750000 | abs. error: 0.2816
 grid pnts:  65 | optim fevals:  12 | fval: -0.984375 | abs. error: 0.0472
 grid pnts: 145 | optim fevals:  20 | fval: -0.986956 | abs. error: 0.0446
 grid pnts: 321 | optim fevals:  20 | fval: -1.026468 | abs. error: 0.0051
 grid pnts: 705 | optim fevals:  24 | fval: -1.031286 | abs. error: 0.0003
Elapsed time is 0.641286 seconds.
```

Optimizing polynomial interpolants

If the objective function is smooth, polynomial interpolants are a good choice. In the example below, by using the Chebyshev-Gauss-Lobatto sparse grid, we achieve an exponential convergence rate for the considered analytic function. To further reduce the number of sparse grid points, we use a dimension-adaptive interpolant. We start with $N = 5$ nodes, and increase the number of nodes by about a factor of 1.5 in each step of the loop, up to about 100 points.

```
Nmax = 100;
N = 5;
z = [];
```

```
warning('off', 'MATLAB:spinterp:maxPointsReached');
tic;
while N <= Nmax
  spoptions = spset('MinPoints', N, 'MaxPoints', N, 'PrevResults', z, ...
    'GridType', 'Chebyshev', 'DimensionAdaptive', 'on', ...
    'KeepFunctionValues', 'on');
  z = spvals(f,2,range,spoptions);
  N = round(z.nPoints .* 1.5);
  [xopt, fval, exitflag, output] = spcgsearch(z,range);
  disp([' grid pnts: ' sprintf('%3d', z.nPoints) ...
        ' | optim fevals: ' sprintf('%3d', output.nFEvals) ...
        ' | fval: ' sprintf('%+5f', fval) ...
        ' | abs. error: ' num2str(abs(f_exact-fval))]);
end
toc;
warning('on', 'MATLAB:spinterp:maxPointsReached');
```

```
 grid pnts:   5 | optim fevals:   1 | fval: +0.000000 | abs. error: 1.0316
 grid pnts:  11 | optim fevals:   9 | fval: +0.000000 | abs. error: 1.0316
 grid pnts:  17 | optim fevals:  20 | fval: -0.537875 | abs. error: 0.49375
 grid pnts:  29 | optim fevals:  29 | fval: -1.031628 | abs. error: 1.9e-11
 grid pnts:  53 | optim fevals:  30 | fval: -1.031628 | abs. error: 1.9e-11
 grid pnts:  85 | optim fevals:  30 | fval: -1.031628 | abs. error: 1.9e-11
Elapsed time is 1.146093 seconds.
```

A high-dimensional example

Let us look at the optimization of a higher-dimensional function. We consider again the function `trid.m` that was already used to illustrate the dimension- adaptive algorithm:

```
type('trid.m');
function y = trid(x)
% TRID   Quadratic function with a tridiagonal Hessian.
%   Y = TRID(X)   returns the function value Y for a D-
%   dimensional input vector X.
%
% The test function is due to Arnold Neumaier, listed
% on the global optimization Web page at
%   http://www.mat.univie.ac.at/~neum/glopt/

d = length(x);
y = sum((x-1).^2) - sum(x(2:d).*x(1:d-1));
```

We let $d = 100$, and compute the known exact minimal value for comparison:

```
d = 100;
range = repmat([-d^2 d^2],d,1);
f_exact = -d*(d+4)*(d-1)/6
```

```
f_exact =
    -171600
```

For high-dimensional problems, it is important to use dimensional adaptivity. Note that here, as well as in the examples above, we use the `KeepFunctionValues` property to indicate that the function values obtained during the sparse grid construction should be retained in order to save time when selecting a good start point for the search.

```
options = spset('DimensionAdaptive', 'on', ...
                'DimadaptDegree', 1, ...
                'FunctionArgType', 'vector', ...
                'RelTol', 1e-3, ...
                'GridType', 'Chebyshev', ...
                'KeepFunctionValues', 'on');

Nmax = 40000;
N = 2*d;
z = [];
warning('off', 'MATLAB:spinterp:maxPointsReached');
tic;
xopt = [];
fval = [];
while N <= Nmax
  spoptions = spset(options, 'MinPoints', N, ...
    'MaxPoints', N, 'PrevResults', z);
  z = spvals(@trid,d,range,spoptions);
  z = sppurge(z);
  spoptoptions = spoptimset('TolFun',1e-6);
  [xopt,fval,exitflag,output] = spcgsearch(z,range,spoptoptions);
  N = round(z.nPoints .* 2);
  disp([' grid pnts: ' sprintf('%5d', z.nPoints) ...
        ' | optim fevals: ' sprintf('%4d', output.nFEvals) ...
        ' | fval: ' sprintf('%+9.1f', fval) ...
        ' | abs. error: ' num2str(abs(f_exact-fval))]);
end
toc;
warning('on', 'MATLAB:spinterp:maxPointsReached');
```

```
 grid pnts:   201 | optim fevals:   11 | fval:      -0 | abs. error: 171600
 grid pnts:   443 | optim fevals:   29 | fval:     -18 | abs. error: 171582
 grid pnts:   923 | optim fevals:   48 | fval:    -132 | abs. error: 171468
 grid pnts:  1883 | optim fevals:   93 | fval:    -537 | abs. error: 171063
 grid pnts:  3899 | optim fevals:  117 | fval:   -1202 | abs. error: 170398
 grid pnts:  7889 | optim fevals:  188 | fval:   -3293 | abs. error: 168307
 grid pnts: 16043 | optim fevals:  248 | fval:  -10725 | abs. error: 160874
 grid pnts: 32477 | optim fevals:  305 | fval:-171600 | abs. error: 5.6e-08
Elapsed time is 188.371965 seconds.
```

Using third-party optimization algorithms

Instead of using the optimization algorithms provided with the Sparse Grid Interpolation Toolbox, you can also use third-party optimization methods. In the following example, we use `fmincon` from The Mathwork's Optimization Toolbox on `spsurfun` to optimize the sparse grid interpolant obtained in the last step of the loop from the example above.

```
optimsetoptions = optimset('GradObj','on', ...
  'LargeScale','off');
[xopt,fval,exitflag,output] = fmincon(@(x) spsurfun(x,z), ...
  range(:,1)+range(:,2))/2,[],[],[],[],range(:,1),range(:,2), ...
  [], optimsetoptions);
disp([' grid pnts: ' sprintf('%5d', z.nPoints) ...
      ' | optim fevals: ' sprintf('%4d', output.funcCount) ...
      ' | fval: ' sprintf('%+9.1f', fval) ...
      ' | abs. error: ' num2str(abs(f_exact-fval))]);
```

```
Optimization terminated: magnitude of directional derivative in search
 direction less than 2*options.TolFun and maximum constraint violation
  is less than options.TolCon.
No active inequalities
 grid pnts: 32477 | optim fevals: 5711 | fval:-171600 | abs. error: 1.1e-07
```

2.6 Improving performance

The aim of this section is to provide an overview on how to optimize the performance of the Sparse Grid Interpolation Toolbox.

Vectorizing the objective function

Vectorizing the objective function is most beneficial if the function evaluations are very cheap, in the order of less than 1/100 s. In this case, providing a vectorized function can improve the performance of the `spvals` function. Consider the following function

$$f(x_1, x_2) = (x_1 + x_2)^2$$

and the following two m-files implementing it:

```
function y = fun(x1, x2)
y = x1 * x2;
y = y^2;
```

```
function y = fun_vec(x1, x2)
y = x1 .* x2;     % Use '.' before any '^', '*' or '/' to enable
y = y.^2;         % vectorized evaluation of expressions
```

The first m-file allows for evaluation at a single real-valued point only, the second one permits vectorized evaluation. Since in case of cheap functions, the function calls in Matlab represent a significant overhead, the function evaluation part of the `spvals` algorithm is much slower if the non-vectorized form is used. This is demonstrated by the following code.

```
tic, z1 = spvals('fun',2); toc;
tic, z2 = spvals('fun_vec',2,[],spset('Vectorized','on')); toc;
z1.fevalTime
z2.fevalTime
```

```
Elapsed time is 0.112452 seconds.
Elapsed time is 0.069006 seconds.
ans =
    0.1021
ans =
    0.0480
```

Reusing previous results

An important feature of the toolbox is that you do not have to discard previously computed results. A "best practice" is, therefore, to embed the interpolant construction in a loop. Proceeding in this way has two advantages: First, it gives the user a maximum of control in monitoring the decay of the estimated interpolation error. Second, it makes it possible to start with a low number of required points, and to increase this number slowly if the targeted accuracy is not yet achieved. There are several examples on how to implement such a loop in the provided demos. See, for instance, `spadaptanim.m` or `spcompare.m` in the `examples` directory. A small example on implementing dimension-adaptive interpolant construction in a loop is provided below.

```
np = 2;
z  = [];
options = spset('Vectorized', 'on', 'DimensionAdaptive', 'on', ...
  'RelTol', inf);
while np < 4000
  options = spset(options, 'PrevResults', z, 'MinPoints', np, ...
                           'MaxPoints', np);
  z = spvals('fun_vec',2,[],options);
  np = z.nPoints;
  disp(['np␣=␣' num2str(np) ',␣e_rel␣=␣', num2str(z.estRelError)]);
  np = np * 2;
end
```

```
np = 5, e_rel = 0.75
np = 13, e_rel = 0.5625
np = 29, e_rel = 0.046875
np = 73, e_rel = 0.011719
np = 177, e_rel = 0.0029297
```

```
np = 417, e_rel = 0.00073242
np = 897, e_rel = 6.1035e-05
np = 1921, e_rel = 4.5776e-05
np = 4097, e_rel = 3.8147e-06
```

Purging interpolant data

Since version v3.2 of the toolbox, a new function called `sppurge` is available. This function serves to "purge" or "clean up" the interpolant data from sub-grids that do not contribute significantly to the result. This is done by introducing a drop tolerance that is applied to the hierarchical surpluses. Sub-grids where the absolute value of all hierarchical surpluses fall below this drop tolerance are marked and neglected during the interpolation process. By default, very conservative purging parameters are used, guaranteeing that the accuracy of the interpolation will not be affected up to about the 12th significant digit. However, if the accuracy requirements are lower, the user may use higher drop tolerances, and thus, trade improved interpolation speed against lower accuracy. This is illustrated by the following example. We assume that an interpolant was computed for the function `fun_vec` by the code above with 4097 points, using piecewise multilinear basis functions. The following code generates a plot that shows the time required to compute 1000 randomly sampled points for different drop tolerances. The maximum absolute error is shown for comparison. This example only uses absolute drop tolerances (the relative drop tolerance is set to zero).

```
% Define drop tolerances
dropTols = [1e-5, 1e-4, 1e-3];

% Generate 1000 random points
rand('state',0);
x = rand(1000,1); y = rand(1000,1);

% Compute exact function values
f_exact = fun_vec(x,y);

e = zeros(3,1); t = zeros(3,1);
for k = 1:3
  % Purge interpolant with drop tolerance
  z = sppurge(z,spset('DropTol', [dropTols(k), 0]));
  % Interpolate and measure time
  tic, ip = spinterp(z, x, y); t(k) = toc;
  % Compute maximum error
  e(k) = max(abs(f_exact - ip));
end

% Plot results
subplot(1,2,1);
bar(t, 'b');
set(gca,'XTickLabel', {'1e-5','1e-4','1e-3'})
```

```
xlabel('Abs.␣drop␣tolerance');
ylabel('Computing␣time␣[s]');
subplot(1,2,2);
bar(log10(e), 'r');
set(gca,'XTickLabel', {'1e-5','1e-4','1e-3'})
set(gca,'YDir','reverse');
set(gca,'YLim', [-6 -2]);
set(gca,'YTick',[-5 -4 -3]);
set(gca,'YTickLabel', {'1e-5','1e-4','1e-3'})
xlabel('Abs.␣drop␣tolerance');
ylabel('Max.␣absolute␣error');
```

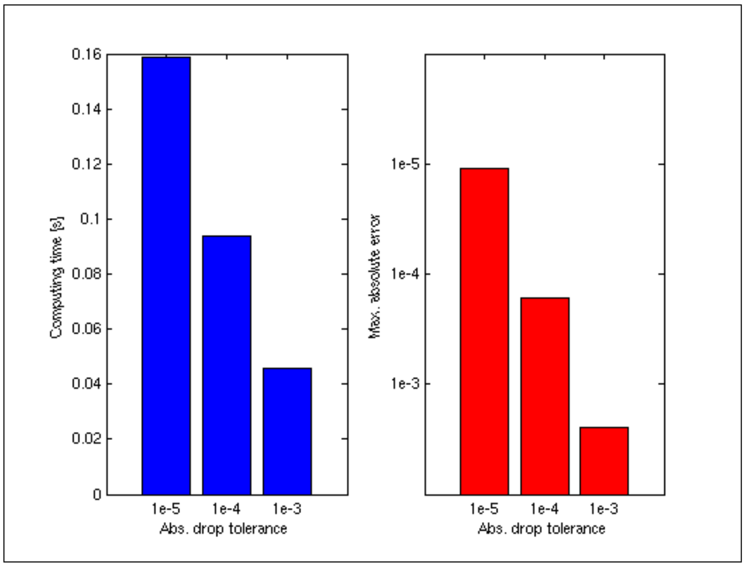

For another example using the default relative drop tolerance, see `sppurge` in the function reference.

Vectorized interpolant evaluation

The `spinterp` function is designed for vectorized evaluation. Since the sparse grid algorithm involves more computational overhead than other, simpler interpolation methods, and due to the fact that MATLAB is relatively slow if many function calls are performed (since it is an interpreted language), it is recommended to evaluate as many interpolation points at a time as possible. The following code illustrates non-vectorized vs. vectorized evaluation at 1000 points for the interpolant from above.

```
% Non-vectorized interpolation
tic
for k = 1:1000
  ip = spinterp(z,x(k),y(k));
```

```
end
toc

% Vectorized interpolation
tic, ip = spinterp(z,x,y); toc
```

```
Elapsed time is 2.526127 seconds.
Elapsed time is 0.045744 seconds.
```

2.7 Interfacing concepts

Applying the `spvals` method to construct interpolants sometimes requires a small interface function. In this section, we show the most important categories of Matlab function headers and (if necessary) how to design an appropriate interface function for them. Tables 2.1, 2.2 show the basic function header types discussed here. Combinations of those are of course also possible and can be derived from the treated cases. In the tables, the objective interpolation variables (all must be real-valued scalars) are denoted by $x_1,\dots,x_n$. Examples of the presented cases are provided below.

Table 2.1: Interface function NOT required.

#	*header*	*variable types*
1	*out* = fun$(x_1,x_2,\dots,x_n)$	$x_1,\dots,x_n$ are real scalars
2	*out* = fun$(x_1,x_2,\dots,x_n,p_1,p_2,\dots,p_m)$	$x_1,\dots,x_n$ are real scalars, $p_1,\dots,p_m$ are parameters of arbitrary type (double array, cell array, structure, etc.)
3	*out* = fun$(x_1,\dots,x_{i_1},p_1,\dots,p_{j_1},x_{i_1+1},\dots,x_{i_2},p_{j_1+1},\dots,p_{j_2},\dots)$	$x_1,\dots,x_n$ are real scalars, $p_1,\dots,p_m$ are parameters of arbitrary type (double array, cell array, structure, etc.)
4	*out* = fun(v)	v is a row or column vector with the entries $x_1,\dots,x_n$
5	*out* = fun$(v,p_1,p_2,\dots,p_m)$	v is a row or column vector with the real scalar entries $x_1,\dots,x_n$, and $p_1,\dots,p_m$ are parameters of arbitrary type (double array, cell array, structure, etc.)
6	$[out_1,out_2,\dots,out_n]$ = fun$(\dots)$	$out_1,\dots,out_n$ are real scalar output parameters, input parameters the same as one of the above
7	*varargout* = fun$(\dots)$	*varargout* is a cell array of real scalar output parameters $out_1,\dots,out_n$, input parameters the same as one of the above

Table 2.2: Interface function REQUIRED (only some exemplary cases).

#	*header*	*variable types*
8	*out* = fun$(A,p_1,p_2,\dots,p_m)$	A is a matrix where some of its entries are the objective interpolation parameters $x_1,\dots,x_n$, and $p_1,\dots,p_m$ are parameters of arbitrary type as above
9	v_{out} = fun$(x_1,x_2,\dots,x_n)$	v_{out} is a row or column vector with real scalar outputs

Examples

Type 1: *out* = $\texttt{fun}(x_1,x_2,\ldots,x_n)$

Objective function:

```
function y = fun1(x1, x2)
y = x1 .* x2;     % Use '.' before any '^', '*' or '/' to enable
y = y.^2;         % vectorized evaluation of expressions
```

Example for call to `spvals`:

```
options = spset('Vectorized', 'on');
range   = [0,2; 0,2];
z = spvals(@fun1, 2, range, options);
```

Type 2: *out* = $\texttt{fun}(x_1,x_2,\ldots,x_n,p_1,p_2,\ldots,p_m)$

Objective function:

```
function y = fun2(x1, x2, c, params)
y = c .* (params.p1 .* x1 + length(params.p2) .* x2);
```

Example for call to `spvals`:

```
options = spset('Vectorized', 'on');
range = [];    % use default range [0,1]^d
c = 2;
params = struct('p1', 3, 'p2', 'hello');
z = spvals(@fun2, 2, range, options, c, params);
```

Type 3: *out* = $\texttt{fun}(x_1,\ldots,x_{i_1},p_1,\ldots,p_{j_1},x_{i_1+1},\ldots,x_{i_2},p_{j_1+1},\ldots,p_{j_2},\ldots)$

Objective function:

```
function y = fun3(p1, x1, p2, x2)
y = p1 .* x1 + p2 .* x2;
```

Example for call to `spvals`:

```
options = spset('VariablePositions', [2 4], 'Vectorized', 'on');
range = [0,1; -1,2];
p1 = 2; p2 = 3;
z = spvals(@fun3, 2, range, options, p1, p2);
```

Type 4: *out* = $\texttt{fun}(v)$

Objective function:

```
function y = fun4(x)
y = prod(x);
```

Example for call to spvals:

```
options = spset('FunctionArgType', 'vector');
range = [0 1; 1 2; 2 3; 3 4; 4 5];
z = spvals(@fun4, 5, range, options);
```

Type 5: out = fun$(v, p_1, p_2, \ldots, p_m)$

Objective function:

```
function y = fun5(x,p);
y = x(:)'*p;    % Compute dot product
```

Example for call to spvals:

```
options = spset('FunctionArgType', 'vector');
range = []; % use default range [0,1]^d
p = rand(3,1);
z = spvals(@fun5, 3, range, options, p);
```

Type 6: $[out_1, out_2, \ldots, out_n]$ = fun$(\ldots)$

Objective function:

```
function [y1, y2] = fun6(x1, x2);
y1 = 2*x1 + 3*x2;
y2 = 4*x1 - 1*x2;
```

Example for call to spvals:

```
options = spset('NumberOfOutputs', 2);
range = []; % use default range [0,1]^d
z = spvals(@fun6, 2, range, options);
```

To compute interpolated values of functions with multiple output parameters, see Section 2.2.

Type 7: $varargout$ = fun$(\ldots)$

Objective function:

```
function varargout = fun7(x1,x2,nout);
for k = 1:nout
  varargout{k} = x1.^k + k.*x2;
end
```

Example for call to spvals:

```
nout = 4;
options = spset('NumberOfOutputs', nout, 'Vectorized', 'on');
range = []; % use default range [0,1]^d
z = spvals(@fun7, 2, range, options, nout);
```

To compute interpolated values of functions with multiple output parameters, see Section 2.2.

Type 8: *out* = fun$(A, p_1, p_2, \ldots, p_m)$

Objective function:

```
function y = fun8(A, f);
y = A\f;
```

Assume that the diagonal entries of A, i.e. $a_{11}, a_{22}, \ldots, a_{nn}$ vary in some given range. An interpolant of fun8 is sought for these varying diagonal entries of A:

```
d = 3;
A = magic(d); f = ones(d,1);
range = [diag(A)-0.5 diag(A)+0.5];
nout = d;
options = spset('NumberOfOutputs', nout, 'FunctionArgType', 'vector');
z = spvals(@interface_fun8, d, range, options, A, f);
```

The interface function `interface_fun8` looks like this:

```
function varargout = interface_fun8(a, A, f);
% Interface function to fun8

% Write the modifiable entries into A
for k = 1:length(a);
  A(k,k) = a(k);
end

% Call objective function fun8
y = fun8(A,f);

% Put the results in cell array (outputs must be cell row vector
% of scalars to be treated by spvals)
varargout = num2cell(y)';
```

Note that the original output, a column vector from the solution of the linear equation system is transformed into a cell array with a single row to match one of the admissible output variants. The original input is also modified to contain the interpolation parameters as a vector, which is permitted by `spvals`. The original Matrix as well as the right-hand side f are passed as additional parameters. To compute interpolated values of functions with multiple output parameters, see Section 2.2.

Type 9: v_{out} = fun$(x_1, x_2, \ldots, x_n)$

Objective function:

```
function y = fun9(x1, x2)
y = [x2 .* cos(x1); ...
     x2 .* sin(x1); ...
     x2];
```

Assume that the output of `fun9` is not a list of real scalars or a `varargout` cell array. In this case, a conversion of the output is required. The interface function uses Matlab's `num2cell` function to achieve this.

```
function varargout = interface_fun9(x1, x2);
y = fun9(x1, x2);
varargout = num2cell(y)';
```

Example for call to spvals:

```
nout = 3;
options = spset('NumberOfOutputs', nout);
z = spvals(@interface_fun9, 2, [], options);
```

To compute interpolated values of functions with multiple output parameters, see Section 2.2.

2.8 Approximating ODEs

As an example for a more complex function with multiple input- and output arguments, we show how to handle an ordinary differential equation. The model considered is a second order differential equation

$$Q''(t) + aQ'(t) + b = 50\cos(t)$$

from [11, pp. 145–162] simulating an electrical circuit.

The ODE model in Matlab

Rewriting this second-order equation as a system of first order equations, we can define the ODE file in Matlab format as follows:

```
function [out1, out2, out3] = circuit(t, u, flag, a, b);
% CIRCUIT   definition of the electrical circuit ODE.

switch flag
 case ''
  out1 = [u(2); 50*cos(t) - a*u(2) - b*u(1)];
 case 'init'
  out1 = [0; 5];        % tspan
  out2 = [5; 1];        % initial conditions
  out3 = odeset('RelTol', 1e-6);
end
```

We can solve this ODE for $a = 2$, $b = 4$, and the default initial conditions and time span as defined in the ODE file using the MATLAB solver `ode45`.

```
[t,Q] = ode45('circuit', [], [], [], 2, 4);
plot(t,Q)
xlabel('t');
grid on;
legend('Q(t)', 'Q''(t)');
```

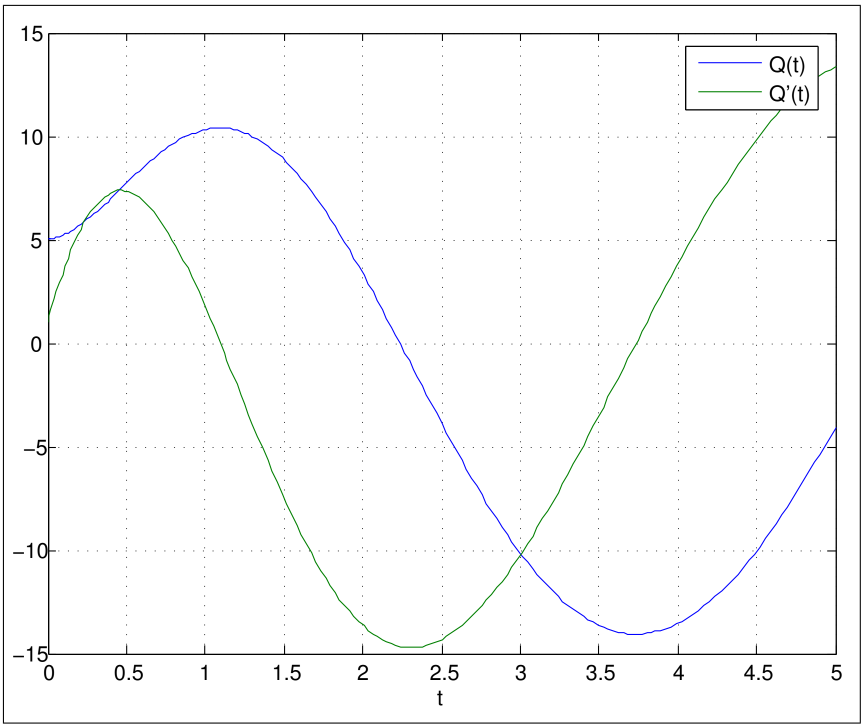

Interpolation problem statement and possible applications

We now consider the initial conditions and the parameters a, b to vary in some range, that is we assume intervals for $Q(0)$, $Q'(0)$, a, and b, and compute an error-controlled sparse grid interpolant for the ODE model at each time step. The interpolant can then be used to do several useful analyses, for instance, perform a Monte Carlo simulation with random variables, optimize the model for the given range of parameters and initial conditions, e.g. minimize or maximize the amplitude, or compute an envelope of the result using fuzzy calculus or interval analysis. In many cases, this can be done considerably faster than by using the original ODE directly, since the construction and evaluation of the interpolant is very fast.

The interface function

We proceed as follows. First of all, we write an interface function of the ODE model to enable its evaluation by the spvals function.

```
function varargout = interface_circuit(Q0, Q0prime, a, b, tspan, nsteps)
% Definition of the complete model as a function of the uncertain
% input parameters.

% The time steps must be at fixed steps such that the number of
% outputs and time steps stay the same for each parameter
% variation.
t = linspace(tspan(1), tspan(2), nsteps);
```

```
% Call the ODE solver
[t, Q] = ode45('circuit', t, [Q0 Q0prime], [], a, b);

% Convert result vector to parameter list. This conversion is
% necessary, since the output arguments of the objective function
% to SPVALS must all be scalar. In this case, we assume that only
% the first column (i.e. Q, not Q') is of interest and thus
% returned.
varargout = num2cell(Q(:,1)');
```

Interpolant construction

Next, we construct the interpolant, simultaneously for all time steps. Here, we use the intervals $[Q(0)] = [4,6]$, $[Q'(0)] = [0,2]$, $[a] = [1,3]$, and $[b] = [3,5]$.

```
% Problem dimension
d = 4;

% Define the time span considered
tspan = [0 5];

% Define the number of steps to consider
nsteps = 101;

% Define the objective range of the initial conditions and the
% parameters
range = [4 6;  % [Q(0)]
         0 2;  % [Q'(0)]
         1 3;  % [a]
         3 5]; % [b]

% Maximum number of sparse grid levels to compute
nmax = 3;

% Initialize z
z = [];

% Turn insufficient depth warning off, since it is anticipated.
warning('off', 'MATLAB:spinterp:insufficientDepth');

% Compute increasingly accurate interpolants; use previous results;
% display estimated maximum relative error over all time steps at
% each iteration.
for n = 1:nmax
  options = spset('Vectorized', 'off', 'MinDepth', n, 'MaxDepth', ...
```

```
                      n, 'NumberOfOutputs', nsteps, 'PrevResults', z);
  z = spvals('interface_circuit', d, range, options, tspan, nsteps);
  disp(['Current␣(estimated)␣maximum␣relative␣error␣over␣all␣time' ...
        'steps:␣', num2str(z.estRelError)]);
end

% Turn insufficient depth warning back on
warning('on', 'MATLAB:spinterp:insufficientDepth');
```

```
Current (estimated) maximum relative error over all timesteps: 0.64844
Current (estimated) maximum relative error over all timesteps: 0.34119
Current (estimated) maximum relative error over all timesteps: 0.057381
```

Computing interpolated values

We can now compute interpolated values at each time step, for any combination of parameters within the range that the interpolant was computed for. The structure z contains all the required information. We only need to select the desired output parameter (i.e. the time step in this example). To compute 10 randomly distributed values at time $t = 5$ (which is step #101 with the chosen discretization) within the box $[Q(0)] \times [Q'(0)] \times [a] \times [b]$, we would simply use the following commands:

```
% Compute 10 randomly distributed points in [0,1] and re-scale them to
% the objective range
x = cell(1,4);
for k = 1:d
  x{k} = range(k,1) + rand(1,10) .* (range(k,2) - range(k,1));
end

% Select output parameter #101
z.selectOutput = 101;
% Compute and display interpolated values
y = spinterp(z, x{:})
```

```
y =
  Columns 1 through 7
   -2.7824   -1.3367   -1.8990   -3.4497   -1.9133   -4.1978   -0.1284
  Columns 8 through 10
   -4.9939   -8.1073   -3.3928
```

2.9 External models

Through system calls available in Matlab, one can easily execute external programs computing external models. The results from the external program can either be passed as an output stream

(requires subsequent parsing of the stream to retrieve the results in usable format), or by saving the results to a file and reading the results from Matlab.

By embedding the system calls, reading/parsing of the result, etc., in Matlab functions, one can obtain wrapper functions that are treatable like regular Matlab functions, and thus, easily accessible to the spvals algorithm. In the following, we present Matlab pseudo-code for a possible approach.

```
function [varargout] = external_model(external_config, x1, ..., xd)

try
  store permutation (x1,...xd) to external_config.inputfile

  % Start external program, pass input file name to program, pass
  % output file name to program.
  system([external_config.program '␣-i␣' external_config.inputfile ...
         '␣-o␣' external_config.outputfile]);

  read result from external_config.outputfile into varargout
catch
  Do some error handling
end
```

In the presented case, the call to spvals would look like this:

```
external_config.program = 'myprog.exe';
external_config.inputfile = 'in.txt';
external_config.outputfile = 'out.txt';
options = spset('VariablePositions', [1 + 1:d], 'NumberOfOutputs', nout);
z = spvals(@external_model, d, range, options, external_config);
```

3 Functions – Alphabetical List

cmpgrids

Compare the available sparse grid types.

Syntax

```
cmpgrids
cmpgrids(N)
cmpgrids(N,D)
```

Description

`cmpgrids` Compares the maximum-norm-based grid, the no-boundary-nodes grid, the Clenshaw-Curtis grid, the Chebyshev-Gauss-Lobatto grid, and the Gauss-Patterson grid in dimension $D = 2$ and level $N = 3$.

`cmpgrids(N)` Compares the grids for level N.

`cmpgrids(N,D)` Compares the grids in dimension D. Permitted are only the values $D = 2$ or $D = 3$.

Examples

The following statement plots the four available sparse grids with level N = 3 in two dimensions, producing the following graph.

```
cmpgrids(3,2);
```

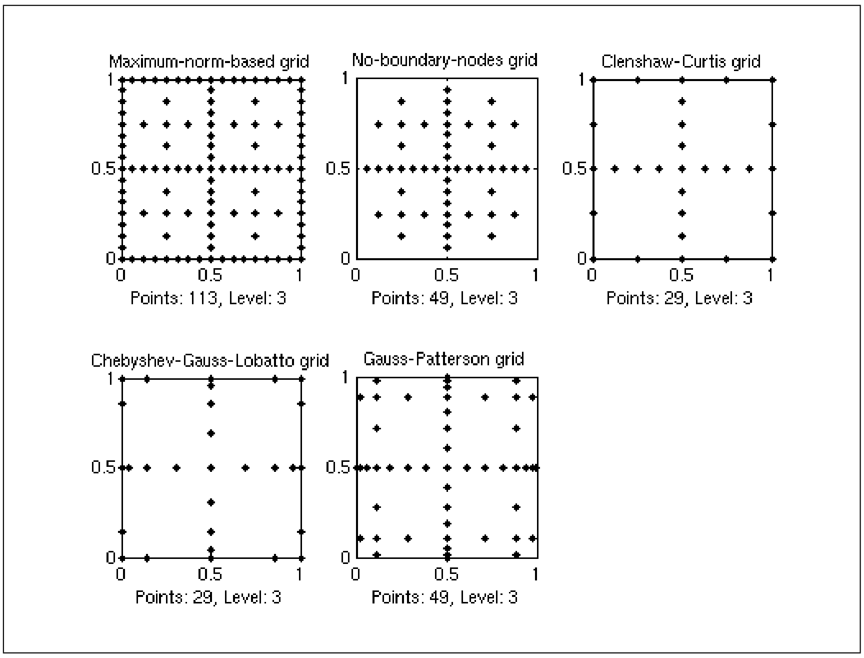

See Also `plotgrid`, `plotindices`, `spgrid`.

---

plotgrid

Plots a sparse grid.

Syntax

```
plotgrid(N,D)
plotgrid(N,D,OPTIONS)
H = plotgrid(...)
```

Description

`plotgrid(N,D)` Plots the sparse grid of level *N* and dimension *D*. By default, the Clenshaw-Curtis sparse grid type is selected.

`plotgrid(N,D,OPTIONS)` Plots the sparse grid, but with the grid type as specified in `OPTIONS`. `OPTIONS` must be a structure created with the `spset` function. `H = PLOTGRID(...)` Returns a vector of handles to the grid points (useful for changing the look of the plotted grid).

Examples

The following statements can be used to plot the Chebyshev-Gauss-Lobatto sparse grid of level $N = 4$ in three dimensions, highlighting the grid points of the levels in different colors:

```
options = spset('GridType', 'Chebyshev');
n = 4;
h = plotgrid(n,3,options);
cols = brighten(jet(n+1),-1);
legendstr = cell(1,n+1);
for k = 0:n
  set(h(k+1), 'Color', cols(k+1,:), 'MarkerSize', 20);
  legendstr{k+1} = ['n␣=␣' num2str(k)];
end
grid on;
legend(legendstr);
```

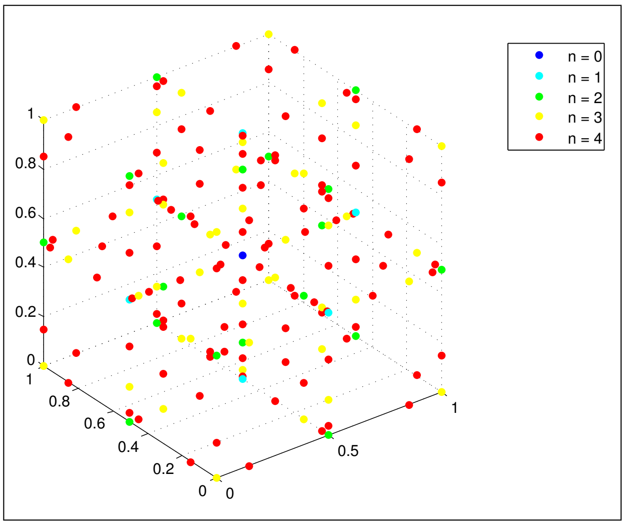

See Also `cmpgrids`, `plotindices`, `spgrid`.

plotindices

Visualizes the index sets of a two-dimensional dimension-adaptive sparse grid.

Syntax

```
plotindices(Z)
```

Description

`plotindices(Z)` Plots the set of multi-indices S_k of a two-dimensional dimension-adaptive sparse grid interpolant $A_{S_k}(f)$. `Z` must be the sparse grid data as returned by `spvals`. `spvals` must be called with the option `'DimensionAdaptive'` switched `'on'` (this can be done using `spset`).

Examples

The following code constructs a dimension-adaptive sparse grid interpolant of the function

$$f(x,y) = \sin(10x^2) + y^2$$

using greedy grid refinement (the degree of dimensional adaptivity is set to 1). The default interpolation box is `range`$= [0,1]^2$.

```
f = inline('sin(10.*x)+y.^2');
options = spset('DimensionAdaptive', 'on', 'DimAdaptDegree', 1);
z = spvals(f, 2, [], options)
```

```
z =

              vals: {[149x1 double]}
          gridType: 'Clenshaw-Curtis'
                 d: 2
             range: []
       estRelError: 0.0018
       estAbsError: 0.0039
        fevalRange: [-0.9589 1.2500]
        minGridVal: [0.5000 0]
        maxGridVal: [0.1562 0.5000]
           nPoints: 149
         fevalTime: 0.3286
   surplusCompTime: 0.0089
           indices: [1x1 struct]
          maxLevel: [7 4]
     activeIndices: [3x1 uint32]
    activeIndices2: [13x1 uint32]
                 E: [1x13 double]
                 G: [13x1 double]
                G2: [13x1 double]
      maxSetPoints: 7
          dimAdapt: 1
```

The resulting interpolant is plotted using Matlab's `ezmesh` command and an anonymous function containing the call to `spinterp`. Plotting the multi-index sets used by the interpolant reveals that the refinement is more dense in the x-direction, since more points are required to resolve the oscillation of the sine curve. Due to the greedy refinement, only a single index (2,2)

is computed in joint dimensions, since the error indicator of the multi-index (2,2) is equal to zero (f is a separable function).

```
subplot(1,2,1);
ezmesh(@(x,y) spinterp(z,x,y), [0 1]);
axis square;
subplot(1,2,2);
plotindices(z);
```

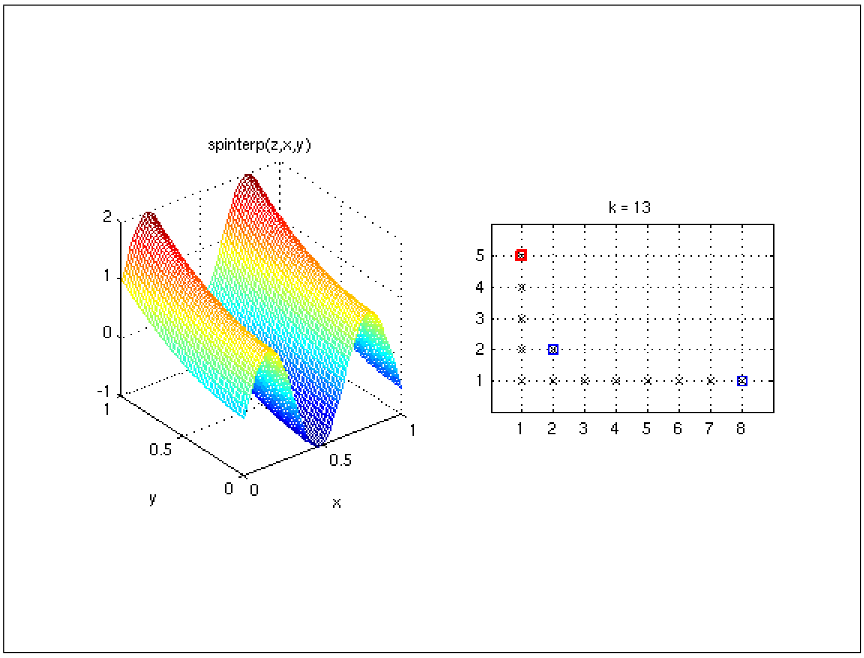

See Also `plotgrid`, `plotindices`, `spgrid`.

spcgsearch

Optimizes the sparse grid interpolant using the CG method. Recommended for optimizing polynomial sparse grids (Chebyshev grid). It is discouraged to apply this method to piecewise linear sparse grids since they are not smooth enough for the algorithm to perform well (use `spcompsearch` instead for these grid types).

Syntax

```
X = spcgsearch(Z)
X = spcgsearch(Z,XBOX)
X = spcgsearch(Z,XBOX,OPTIONS)
```

```
[X,FVAL] = spcgsearch(...)
[X,FVAL,EXITFLAG] = spcgsearch(...)
[X,FVAL,EXITFLAG,OUTPUT] = spcgsearch(...)
```

Description

`X = spcgsearch(Z)` Starts the search at the best available sparse grid point and attempts to find a local minimizer of the sparse grid interpolant `Z`. The entire range of the sparse grid interpolant is searched.

`X = spcgsearch(Z,XBOX)` Uses the search box `XBOX = [a1, b1; a2, b2; ...]`. The size of search box `XBOX` must be smaller than or equal to the range of the interpolant.

`X = spcgsearch(Z,XBOX,OPTIONS)` Minimizes with the default optimization parameters replaced by values in the structure `OPTIONS`, created with the `spoptimset` function. See `spoptimset` for details.

`[X,FVAL] = spcgsearch(...)` Returns the value of the sparse grid interpolant at `X`.

`[X,FVAL,EXITFLAG] = spcgsearch(...)` Returns an `EXITFLAG` that describes the exit condition of spcgsearch. Possible values of `EXITFLAG` and the corresponding exit conditions are

- 1 – `spcgsearch` converged to a solution `X`.
- 0 – Maximum number of function evaluations or iterations reached.

`[X,FVAL,EXITFLAG,OUTPUT] = spcgsearch(...)` Returns a structure `OUTPUT` with the number of function evaluations in `OUTPUT.nFEvals`, the number of gradients in `.nGradEvals`, and the computing time in `.time`.

Examples

Usually, the objective function will be expensive to evaluate. Here, we just consider the well-known the six-hump camel-back for function simplicity.

```
f = @(x,y) (4-2.1.*x.^2+x.^4./3).*x.^2+x.*y+(-4+4.*y.^2).*y.^2;
```

Before applying the `spcgsearch` algorithm, we need to create a sparse grid interpolant of the objective function. This is done as usual using the `spvals` algorithm.
In preparation to calling `spvals`, we first set up the interpolant construction with adequate parameters. A conjugate gradient (CG) line search algorithm uses derivatives to determine the search direction, it best to use the smooth Chebyshev grid in order to obtain an interpolant with accurate, smooth derivatives. Furthermore, it is useful to keep the function values as they can be used by the optimization algorithm to select good starting values for the optimization.

```
options = spset('keepFunctionValues','on', 'GridType', 'Chebyshev', ...
  'DimensionAdaptive', 'on', 'DimAdaptDegree', 1, 'MinPoints', 10);
```

We construct the interpolant for the range that we are interested in optimizing the objective function for.

```
range = [-3 3; -2 2];
```

Now, we are ready to construct the sparse grid interpolant.

```
z = spvals(f, 2, range, options)
```

```
z =
               vals: {[37x1 double]}
           gridType: 'Chebyshev'
                  d: 2
              range: [2x2 double]
        estRelError: 6.7208e-16
        estAbsError: 1.1013e-13
         fevalRange: [-0.9706 162.9000]
         minGridVal: [0.5000 0.6913]
         maxGridVal: [0 0]
            nPoints: 37
          fevalTime: 0.0690
    surplusCompTime: 0.3137
            indices: [1x1 struct]
           maxLevel: [4 3]
      activeIndices: [4x1 uint32]
     activeIndices2: [11x1 uint32]
                  E: [Inf 108.9000 48 48.6000 10.7392 6 16.0000 7.1054e-15
1.1013e-13 7.1054e-15 1.4211e-14]
                  G: [11x1 double]
                 G2: [11x1 double]
       maxSetPoints: 4
           dimAdapt: 1
              fvals: {[37x1 double]}
```

Having obtained the interpolant, we can now search for the minimizer using `spcgsearch`. This is achieved by simply calling

```
[xopt, fval] = spcgsearch(z)
```

```
xopt =
   -0.0898
    0.7127
fval =
   -1.0316
```

There are multiple ways of configuring the search using an options structure defined with `spoptimset`. For instance, you can display information at each iteration. Additional information on the optimization can be obtained by specifying optional left-hand parameters:

```
optoptions = spoptimset('Display', 'iter');
[xopt, fval, exitflag, output] = spcgsearch(z, [], optoptions)
```

```
 Iteration   Func-count Grad-count     f(x)           Procedure
     0           1          1       -0.970563         start point
     1          10          1          -1.024         line search
     2          17          2         -1.03161        line search
     3          24          3         -1.03163        line search
     4          29          4         -1.03163        line search
xopt =
   -0.0898
    0.7127
fval =
   -1.0316
exitflag =
     1
output =
       nFEvals: 29
    nGradEvals: 4
          time: 0.2737
```

See Also spoptimset.

spcompsearch

Optimizes the sparse grid interpolant using the compass (coordinate) search method. Best-suited for piecewise multilinear sparse grids.

Syntax

```
X = spcompsearch(Z)
X = spcompsearch(Z,XBOX)
X = spcompsearch(Z,XBOX,OPTIONS)
[X,FVAL] = spcompsearch(...)
[X,FVAL,EXITFLAG] = spcompsearch(...)
[X,FVAL,EXITFLAG,OUTPUT] = spcompsearch(...)
```

Description

X = spcompsearch(Z) Starts the search at the best available sparse grid point and attempts to find a local minimizer of the sparse grid interpolant Z. The entire range of the sparse grid interpolant is searched.

X = spcompsearch(Z,XBOX) Uses the search box XBOX = [a1, b1; a2, b2; ...]. The size of search box XBOX must be smaller than or equal to the range of the interpolant.

X = spcompsearch(Z,XBOX,OPTIONS) Minimizes with the default optimization parameters

replaced by values in the structure OPTIONS, created with the spoptimset function. See spoptimset for details.

[X,FVAL] = spcompsearch(...) Returns the value of the sparse grid interpolant at X.

[X,FVAL,EXITFLAG] = spcompsearch(...) Returns an EXITFLAG that describes the exit condition of spcompsearch. Possible values of EXITFLAG and the corresponding exit conditions are

- 1 – spcompsearch converged to a solution X.
- 0 – Maximum number of function evaluations or iterations reached.

[X,FVAL,EXITFLAG,OUTPUT] = spcompsearch(...) Returns a structure OUTPUT with the number of function evaluations in OUTPUT.nFEvals and the computing time in .time.

Examples

A compass search algorithm is a direct search method that does not need derivatives, so it is well-suited to optimize a piecewise multilinear sparse grid interpolant computed for the grid types Maximum, NoBoundary, or Clenshaw- Curtis.
As with the example presented for spcgsearch, we consider the six-hump camel- back function.

```
f = @(x,y) (4-2.1.*x.^2+x.^4./3).*x.^2+x.*y+(-4+4.*y.^2).*y.^2;
```

We create the sparse grid interpolant using spvals as follows. Note that it is useful to keep the function values as they can be used by the optimization algorithm to select good starting values for the optimization without having to evaluate the interpolant.

```
options = spset('keepFunctionValues','on', 'GridType', 'Clenshaw-Curtis', ...
  'DimensionAdaptive', 'on', 'DimAdaptDegree', 1, 'MinPoints', 10);
```

The next steps are setting the interpolation range (the optimization range will be the same by default), constructing the interpolant, providing additional (optional) optimization parameters, and finally, the call to the spcompsearch algorithm.

```
range = [-3 3; -2 2];
z = spvals(f, 2, range, options)
optoptions = spoptimset('Display', 'iter');
[xopt, fval] = spcompsearch(z, [], optoptions)
```

```
z =

              vals: {[205x1 double]}
          gridType: 'Clenshaw-Curtis'
                 d: 2
             range: [2x2 double]
       estRelError: 0.0037
       estAbsError: 0.6031
        fevalRange: [-0.9970 162.9000]
```

```
          minGridVal: [0.5000 0.3281]
          maxGridVal: [0 0]
             nPoints: 205
           fevalTime: 0.1031
     surplusCompTime: 0.0077
             indices: [1x1 struct]
            maxLevel: [7 6]
       activeIndices: [4x1 uint32]
      activeIndices2: [17x1 uint32]
                   E: [Inf 108.9000 48 52.2844 45.8547 6 24 12.7500 22.3788
4.3594 7.6817 0 0 1.2568 2.2379 0.3364 0.6031]
                   G: [17x1 double]
                  G2: [17x1 double]
        maxSetPoints: 7
            dimAdapt: 1
               fvals: {[205x1 double]}
 Iteration   Func-count Grad-count     f(x)           Procedure
     0            0         0       -0.997009         start point
     1            4         0       -0.997009         contract step
     2            8         0       -0.997009         contract step
     3           12         0       -0.997009         contract step
     4           16         0        -1.02647         coordinate step
     5           20         0        -1.02647         contract step
     6           24         0        -1.02647         contract step
xopt =
    0.0938
   -0.6875
fval =
   -1.0265
```

See Also spoptimset.

spdim

Computes the number of sparse grid points.

Syntax

```
P = spdim(N,D)
P = spdim(N,D,OPTIONS)
```

Description

`P = spdim(N,D)` Computes the number of points of the sparse grid of dimension D and level N.

`P = spdim(N,D,OPTIONS)` Computes the number of points as above, but with the default grid type replaced by the grid type specified in `OPTIONS`, an argument created with `spset`. See `spset` for details.

Examples

Compute the number of support nodes of the 10-dimensional sparse grid of level 7 for the Clenshaw-Curtis (default) grid with the following command:

```
spdim(7,10)
```

```
ans =
      652065
```

For comparison, compute the number of nodes of the maximum-norm-based sparse grid:

```
options = spset('GridType','Maximum');
spdim(7,10,options)
```

```
ans =
   1.8317e+09
```

spfminsearch

Optimizes the sparse grid interpolant using MATLAB's `fminsearch` method.

Syntax

```
X = spfminsearch(Z)
X = spfminsearch(Z,XBOX)
X = spfminsearch(Z,XBOX,OPTIONS)
[X,FVAL] = spfminsearch(...)
[X,FVAL,EXITFLAG] = spfminsearch(...)
[X,FVAL,EXITFLAG,OUTPUT] = spfminsearch(...)
```

Description

`X = spfminsearch(Z)` Starts the search at the best available sparse grid point and attempts to find a local minimizer of the sparse grid interpolant `Z`. The entire range of the sparse grid interpolant is searched.

`X = spfminsearch(Z,XBOX)` Uses the search box `XBOX = [a1, b1; a2, b2; ...]`. The size of search box `XBOX` must be smaller than or equal to the range of the interpolant.

`X = spfminsearch(Z,XBOX,OPTIONS)` Minimizes with the default optimization parameters replaced by values in the structure `OPTIONS`, created with the `spoptimset` function. See `spoptimset` for details.

`[X,FVAL] = spfminsearch(...)` Returns the value of the sparse grid interpolant at `X`.

`[X,FVAL,EXITFLAG] = spfminsearch(...)` Returns an `EXITFLAG` that describes the exit condition of `spfminsearch`. Possible values of `EXITFLAG` and the corresponding exit conditions are

- 1 – `spfminsearch` converged to a solution `X`.
- 0 – Maximum number of function evaluations or iterations reached.

`[X,FVAL,EXITFLAG,OUTPUT] = spfminsearch(...)` Returns a structure `OUTPUT` with the number of function evaluations in `OUTPUT.nFEvals` and the computing time in `.time`. The `OUTPUT` result from the `fminsearch` call is returned as `OUTPUT.fminsearchOutput`.

Examples

`spfminsearch` internally calls MATLAB's `fminsearch` function to perform the search. The sparse grid interpolant is modified by a penalty function such that the search is restricted to the provided search box.
`spfminsearch` is a derivative-free method that is suitable for all sparse grid types. However, it is usually outperformed by `spcompsearch` for the grid types `Maximum`, `NoBoundary`, or `Clenshaw-Curtis`, and by `spcgsearch` for the grid type `Chebyshev`.
As with the example presented for `spcgsearch`, we consider the six-hump camel- back function (see that example for further details).

```
f = @(x,y) (4-2.1.*x.^2+x.^4./3).*x.^2+x.*y+(-4+4.*y.^2).*y.^2;
```

Interpolant creation and setting optional parameters:

```
options = spset('keepFunctionValues','on', 'GridType', 'Chebyshev', ...
  'DimensionAdaptive', 'on', 'DimAdaptDegree', 1, 'MinPoints', 10);
range = [-3 3; -2 2];
z = spvals(f, 2, range, options);
optoptions = spoptimset('Display', 'iter');
```

Performing the optimization:

```
[xopt, fval] = spfminsearch(z, [], optoptions)
```

```
 Iteration   Func-count     min f(x)         Procedure
     0            1        -0.970563
     1            3        -0.970563         initial simplex
     2            5        -0.997137         expand
     3            7         -0.99731         reflect
```

```
     4            9          -0.99731         contract inside
     5           11          -0.999861        contract inside
     6           13          -1.00004         reflect
     7           15          -1.00004         contract inside
     8           17          -1.00004         contract inside
     9           19          -1.00004         contract inside
    10           21           -1.0002         expand
    11           23          -1.00055         expand
    12           25          -1.00087         expand
    13           27          -1.00192         expand
    14           29          -1.00227         expand
    15           31          -1.00483         expand
    16           32          -1.00483         reflect
    17           34          -1.00771         expand
    18           36          -1.01172         expand
    19           38          -1.01615         expand
    20           40          -1.02567         expand
    21           41          -1.02567         reflect
    22           43          -1.03063         reflect
    23           44          -1.03063         reflect
    24           46          -1.03083         reflect
    25           48          -1.03119         contract inside
    26           50          -1.03155         contract inside
    27           52          -1.03155         contract inside
    28           54          -1.03155         contract inside
    29           56          -1.03162         contract inside
    30           58          -1.03162         contract inside
    31           60          -1.03162         contract inside
    32           62          -1.03162         reflect
    33           64          -1.03163         contract inside
    34           66          -1.03163         contract inside
    35           68          -1.03163         contract inside
    36           70          -1.03163         contract inside
    37           72          -1.03163         contract inside
    38           74          -1.03163         contract inside
    39           76          -1.03163         contract inside
    40           78          -1.03163         contract inside
    41           80          -1.03163         contract inside
    42           82          -1.03163         reflect
    43           84          -1.03163         contract inside

Optimization terminated:
 the current x satisfies the termination criteria using OPTIONS.TolX of
 1.000000e-04
 and F(X) satisfies the convergence criteria using OPTIONS.TolFun of
 1.000000e-04
```

```
xopt =
   -0.0899
    0.7127
fval =
   -1.0316
```

See Also spoptimset.

spget

Get sparse grid interpolation OPTIONS parameters.

Syntax

```
VAL = spget(OPTIONS, 'NAME')
VAL = spget(OPTIONS, 'NAME', DEFAULT)
```

Description

VAL = spget(OPTIONS, 'NAME') Extracts the value of the property NAME from the sparse grid options structure OPTIONS, returning an empty matrix if the property value is not specified in OPTIONS. It is sufficient to type only the leading characters that uniquely identify the property. Case is ignored for property names. [] is a valid OPTIONS argument.

VAL = spget(OPTIONS, 'NAME', DEFAULT) Extracts the named property as above, but returns VAL = DEFAULT if the named property is not specified in OPTIONS.

Examples

Assume that an options structure has been created using the spset command:

```
options = spset('GridType', 'Maximum', 'MaxDepth', 4)
```

```
options =
              GridType: 'Maximum'
                RelTol: []
                AbsTol: []
            Vectorized: []
              MinDepth: []
              MaxDepth: 4
     VariablePositions: []
         NumberOfOutputs: []
           PrevResults: []
```

```
       FunctionArgType: []
    KeepFunctionValues: []
              KeepGrid: []
     DimensionAdaptive: []
             MinPoints: []
             MaxPoints: []
        DimadaptDegree: []
         SparseIndices: []
```

Using spget, we can extract the contents of the structure:

```
gridType = spget(options, 'GridType')
```

```
gridType =
Maximum
```

By using the third argument, we can set a default in case the according property of the structure is empty:

```
minDepth = spget(options, 'MinDepth')
minDepth = spget(options, 'MinDepth', 2)
```

```
minDepth =
     []

minDepth =
     2
```

Note that the default argument has no effect if the accessed property contains a value.

```
gridType = spget(options, 'GridType', 'Clenshaw-Curtis')
```

```
gridType =
Maximum
```

See Also spset.

spgrid

Compute the sparse grid point coordinates.

Syntax

```
X = spgrid(N,D)
X = spgrid(N,D,OPTIONS)
```

Description

`X = spgrid(N,D)` Computes the sparse grid points of level N and problem dimension D. The coordinate value of dimension $i = 1 \dots d$ is stored in column i of the matrix X. One row of the matrix X represents one grid point.

`X = spgrid(N,D,OPTIONS)` computes the sparse grid points as above, but with default grid type replaced by the grid type specified in `OPTIONS`, an argument created with the `spset` function.

Remark Note that spgrid only computes the grid points that are added to the interpolant at level N.

Examples

Compute the grid points of the Clenshaw-Curtis (default) grid for the first 3 levels ($n = 0 \dots 2$), dimension $d = 2$, and display them:

```
d = 2;
for n = 0:2
  x = spgrid(n,d)
end
```

```
x =
     0.5000    0.5000

x =
          0    0.5000
     1.0000    0.5000
     0.5000         0
     0.5000    1.0000

x =
     0.2500    0.5000
     0.7500    0.5000
          0         0
     1.0000         0
          0    1.0000
     1.0000    1.0000
     0.5000    0.2500
     0.5000    0.7500
```

See Also `cmpgrids`, `plotgrid`, `spset`.

spinit

Initialize the Sparse Grid Interpolation toolbox.

Syntax

```
spinit
```

Description

`spinit` Simply adds the sparse grid toolbox directories to the Matlab path. This ensures that the sparse grid routines are found when working in other directories.

Remark To run the sparse grid interpolation demos from the help browser, `spinit` must be started first.

spinterp

Evaluation of the sparse grid interpolant.

Syntax

```
IP = spinterp(Z,Y1,...,YD)
[IP,IPGRAD] = spinterp(Z,Y1,...,YD)
```

Description

`IP = spinterp(Z,Y1,...,YD)` Computes the interpolated values `IP` at the point(s) `(Y1,..., YD)` over the sparse grid. The input parameters `Yi` may be double arrays of equal size for vectorized processing. The sparse grid data must be given as a structure `Z` containing the hierarchical surpluses (computed with `spvals`).

`[IP,IPGRAD] = spinterp(Z,Y1,...,YD)` Computes interpolated values `IP` and derivatives `IPGRAD` at the specified points. The derivatives are returned as `D`$\times$`1` gradient vectors inside of a cell array that has equal size as the double array `IP`. See Section 2.3 for additional information.

Examples

Assume a sparse grid interpolant of the Matlab peaks function has been computed for the domain $[0,2]^2$ using the following command:

```
z = spvals(@(x,y) peaks(x,y), 2, [0,2; 0,2]);
```

Then, we can evaluate z at a single point, e.g. the point (0.5, 0.5), simply like this:

```
ip = spinterp(z, 0.5, 0.5);
```

```
  ip =
      0.3754
```

If multiple evaluations of the interpolant are required, it is best to use a vectorized call to spinterp for fast processing. For example, to evaluate the interpolant at the full grid $[0,2] \times [0,2]$ at 50×50 points (equidistant spacing), we can proceed as follows:

```
x = linspace(0,2,50); y = linspace(0,2,50);
[xmat,ymat] = meshgrid(x,y);
tic; ip = spinterp(z, xmat, ymat); toc
```

```
  Elapsed time is 0.202514 seconds.
```

Note that the output size of ip matches the size of the input matrices:

```
size(xmat)
size(ip)
```

```
  ans =
      50    50
  ans =
      50    50
```

We could visualize the result using the surf command:

```
surf(xmat, ymat, ip);
axis tight;
```

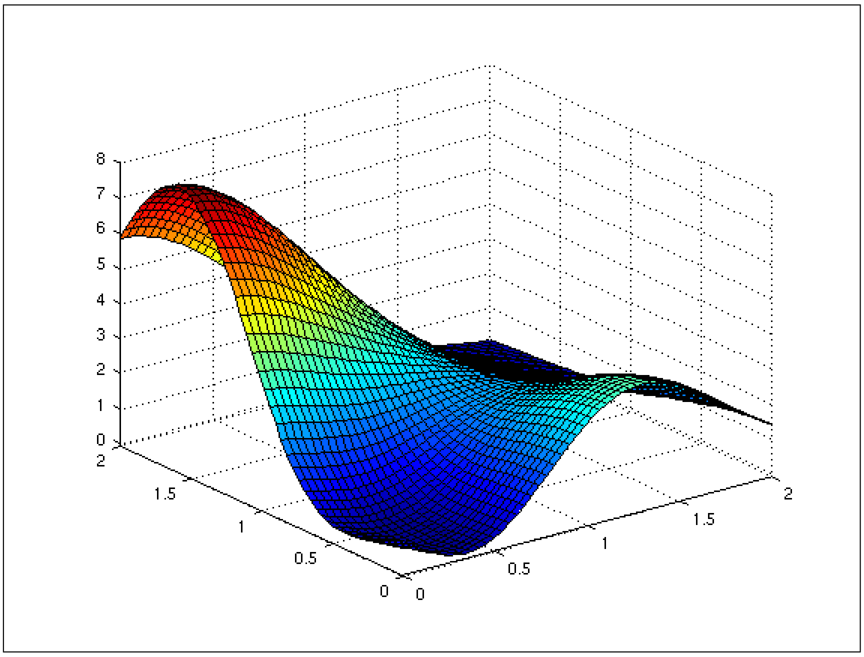

See Also `spvals`.

spmultistart

Attemps to find a global optimizer of the sparse grid interpolant by performing multiple local searches starting from random start points.

Syntax

```
X = spmultistart(Z)
X = spmultistart(Z,XBOX)
X = spmultistart(Z,XBOX,OPTIONS)
[X,FVAL] = spmultistart(...)
[X,FVAL,EXITFLAG] = spmultistart(...)
[X,FVAL,EXITFLAG,OUTPUT] = spmultistart(...)
```

Description

`X = spmultistart(Z)` Attemps to find a global optimizer `X` of the sparse grid interpolant `Z` by performing multiple local searches starting from random start points. The entire range of the sparse grid interpolant is searched. Using the default settings, the first start point is not random but the best available sparse grid point. By default, `spcompsearch` is used for the local searches if the grid type is not of type `Chebyshev`. If it is, `spcgsearch` is used.

`X = spmultistart(Z,XBOX)` Uses the search box `XBOX = [a1, b1; a2, b2; ...]`. The size of search box `XBOX` must be smaller than or equal to the range of the interpolant.

`X = spmultistart(Z,XBOX,OPTIONS)` Minimizes with the default optimization parameters replaced by values in the structure `OPTIONS`, created with the `spoptimset` function. For instance, the local optimization method can be selected. See `spoptimset` for details.

`[X,FVAL] = spmultistart(...)` Returns the value of the sparse grid interpolant at `X`.

`[X,FVAL,EXITFLAG] = spmultistart(...)` Returns an `EXITFLAG` that describes the exit condition of `spmultistart`. Possible values of `EXITFLAG` and the corresponding exit conditions are

- 1 – `spmultistart` converged to at least one solution `X`.
- 0 – Maximum number of function evaluations or iterations reached for all local searches.

`[X,FVAL,EXITFLAG,OUTPUT] = spmultistart(...)` Returns a structure `OUTPUT` with the total computing time in `.time`, and a cell array of all local search results `.allResults`.

Examples

The following example demonstrates the usage of `spmultistart` with its default options. Many parameters can be modified using `spoptimset`. Here, we optimize Branin's function, a function with three global optimizers.

```
f = inline(['(5/pi*x-5.1/(4*pi^2)*x.^2+y-6).^2␣+' ...
            '10*(1-1/(8*pi))*cos(x)+10']);
```

Interpolant creation:

```
range = [-5 10; 0 15];
options = spset('keepFunctionValues','on', 'GridType', 'Chebyshev', ...
                'DimensionAdaptive', 'on', 'DimAdaptDegree', 1, ...
                'MinPoints', 10);
z = spvals(f, 2, range, options);
```

Performing the optimization:

```
[xopt, fval, exitflag, output] = spmultistart(z)
```

```
  xopt =
     -3.1416
     12.2751
  fval =
      0.3978
  exitflag =
       1
  output =
           time: 4.0003
      allResults: [1x1 struct]
```

All local optimization results are available from the output structure:

```
output.allResults.x
output.allResults.fval
```

```
  ans =
      3.1416    9.4248    3.1416    9.4248    3.1416    9.4248   -3.1416
  9.4248   -3.1416    9.4248
      2.2750    2.4750    2.2750    2.4750    2.2750    2.4750   12.2751
  2.4750   12.2751    2.4750
  ans =
      0.3980    0.3979    0.3980    0.3979    0.3980    0.3979    0.3978
  0.3979    0.3978    0.3979
```

See Also `spoptimset`.

spoptimget

Get sparse grid optimization OPTIONS parameters.

Syntax

```
VAL = spoptimget(OPTIONS, 'NAME')
VAL = spoptimget(OPTIONS, 'NAME', DEFAULT)
```

Description

`VAL = spoptimget(OPTIONS, 'NAME')` Extracts the value of the property `NAME` from the sparse grid optimization options structure `OPTIONS`, returning an empty matrix if the property value is not specified in `OPTIONS`. It is sufficient to type only the leading characters that uniquely identify the property. Case is ignored for property names. `[]` is a valid `OPTIONS` argument.

`VAL = spoptimget(OPTIONS, 'NAME', DEFAULT)` Extracts the named property as above, but returns `VAL = DEFAULT` if the named property is not specified in `OPTIONS`.

Examples

Assume that an options structure has been created using the `spoptimset` command:

```
options = spoptimset('TolFun', 1e-4, 'MaxIter', 200)
```

```
options =
           Minimize: []
           Maximize: []
             TolFun: 1.0000e-04
               TolX: []
            MaxIter: 200
         StartPoint: []
        TestCorners: []
         PrevResult: []
             Method: []
          NumStarts: []
     OptimsetOptions: []
            Display: []
```

Using `spoptimget`, we can extract the contents of the structure:

```
tolFun = spoptimget(options, 'TolFun')
```

```
tolFun =
   1.0000e-04
```

By using the third argument, we can set a default in case the according property of the structure is empty:

```
startPoint = spoptimget(options, 'StartPoint')
startPoint = spoptimget(options, 'StartPoint', 'best')
```

```
startPoint =
     []
startPoint =
best
```

Note that the default argument has no effect if the accessed property contains a value.

```
maxIter = spoptimget(options, 'MaxIter', 500)
```

```
maxIter =
   200
```

See Also `spoptimset`.

---

spoptimset

Create/alter a sparse grid optimization OPTIONS structure.

Syntax

```
OPTIONS = spoptimset('NAME1',VALUE1,'NAME2',VALUE2,...)
OPTIONS = spoptimset(OLDOPTS,'NAME1',VALUE1,...)
OPTIONS = spoptimset(OLDOPTS,NEWOPTS)
```

Description

`spoptimset` with no input arguments displays all property names and their possible values.

`OPTIONS = spoptimset('NAME1',VALUE1,'NAME2',VALUE2,...)` creates options structure `OPTIONS` in which the named properties have the specified values. Any unspecified properties have default values. It is sufficient to type only the leading characters that uniquely identify the property. Case is ignored for property names.

`OPTIONS = spoptimset(OLDOPTS,'NAME1',VALUE1,...)` alters an existing options structure `OLDOPTS`.

`OPTIONS = spoptimset(OLDOPTS,NEWOPTS)` combines an existing options structure `OLDOPTS` with a new options structure `NEWOPTS`. Any new properties overwrite corresponding old properties.

Properties

The properties configurable with `spoptimset` are listed in Table 3.1.

Table 3.1: Properties configurable with `spoptimset`.

Property	*Value {default}*	*Description*
`Minimize`	{on} | off	If set to on, the optimization algorithm will search for a minimizer.
`Maximize`	on | {off}	If set to on, the optimization algorithm will search for a maximizer (searching for the minimizer and the maximizer at the same time is allowed). Note that searching for a maximizer is currently not supported by `spcgsearch`.
`TolX`	positive scalar {1e-4}	Termination tolerance on `X`. Note that the tolerance on `X` is taken with respect to the problem being re-scaled to the unit interval in each coordinate direction. That is, for instance, a sparse grid interpolant defined for the box `[0,1e6]x[0,1e-6]` with `TolX = 0.1` would mean a break tolerance of `1e5` in x_1 and a tolerance of `1e-7` in x_2-direction. This parameter does not apply to `spcgsearch`.
`TolFun`	positive scalar {1e-6}	The search is terminated when the change of the function value from one iteration to the next is smaller than `TolFun`.
`MaxIter`	integer {100}	Maximum number of allowed iterations.
`StartPoint`	{best} | random | `Dx1` vector	Start search from best available, random, or specified start point.
`TestCorners`	on | {off}	Specifically includes the 2^D corner points of the search box as potential start points of the search.
`PrevResult`	`(d+1)x{1|2}` double array	Specifies a possible best start point, such as from a previous search over a subdomain of the current search box. Format: `[xoptmin;ymin xoptmax;ymax]`, where `xoptmin` and `xoptmax` are column vectors. Depending on the contents of the `Minimize` and `Maximize` fields, minima and/or maxima information should be provided. `PrevResult` is only considered as a start point if `StartPoint` is set to `best`.
`Method`	{spcgsearch} | {spcompsearch} | spfminsearch	Specifies the method used by the multiple random start search `spmultistart`. `spcgsearch` is the default for the Chebyshev grid, otherwise, it is `spcompsearch`.
`NumStarts`	integer {10}	Number of local searches to perform for the multiple random start method `spmultistart`. The following points are considered: (best) + (NumStarts-1 random points).
`Optimset` `Options`	struct {[]}	This feature is useful if additional configuration of the `fminsearch` algorithm used by `spfminsearch` is required beyond the parameters available through `spoptimset`. Example: `opions = spoptimset('Optimset', optimset('FunValCheck','on'));`
`Display`	{off} | iter	Optionally, displays information at each iteration.

Examples

As a preliminary to the following example, we construct a sparse grid interpolant of a test function (Branin's function) as follows.

```
f = inline(['(5/pi*x-5.1/(4*pi^2)*x.^2+y-6).^2␣+' ...
            '10*(1-1/(8*pi))*cos(x)+10']);
range = [-5 10; 0 15];
options = spset('keepFunctionValues','on', 'GridType', 'Chebyshev', ...
  'DimensionAdaptive', 'on', 'DimAdaptDegree', 1, 'MinPoints', 10, ...
        'RelTol', 1e-6);
z = spvals(f, 2, range, options);
```

A typical case of a modification of the sparse grid optimization options structure is given by the need to specify a more stringent error tolerance on the function value to be used by the spcgsearch algorithm.

```
optoptions = spoptimset('TolFun', 1e-10);
format long e;
[xopt, fval] = spcgsearch(z, [], optoptions)
format short e;
```

```
xopt =
     3.141592655097273e+00
     2.274999997132531e+00
fval =
     3.978873577297303e-01
```

See Also spoptimget.

sppurge

Purge sparse grid data.

Syntax

```
Z = sppurge(Z)
Z = sppurge(Z,OPTIONS)
```

Description

Z = sppurge(Z) Marks indices that have corresponding hierarchical surplus values larger than the default drop tolerance [0, 100*eps]. The sppurge function returns the same sparse grid interpolant data Z, but enhanced by a field purgeData that is used by spinterp to only consider the marked indices in the interpolation process, thus saving computing time.

`Z = sppurge(Z,OPTIONS)` The parameter `OPTIONS` must be an options structure generated with `spset`. Only the value of the `DropTol` property is used, which enables the user to set any absolute and relative drop tolerance to be used by the purging algorithm.

Examples

We consider the quadratic test function

$$f(x) = [\sum_{i=1}^{d}(x_i - 1)^2] - [\sum_{i=2}^{d} x_i x_{i-1}],$$

implemented in Matlab by the following code:

```
function y = trid(x)
% TRID   Quadratic function with a tridiagonal Hessian.
%   Y = TRID(X)   returns the function value Y for a D-
%   dimensional input vector X.
%
% The test function is due to Arnold Neumaier, listed
% on the global optimization Web page at
%   http://www.mat.univie.ac.at/~neum/glopt/

d = length(x);
y = sum((x-1).^2) - sum(x(2:d).*x(1:d-1));
```

During the construction of the interpolant, many sub-grids are encountered that do no contribute to the interpolant, i.e., they have hierarchical surpluses that are all zero (up to floating point accuracy). An adaptive algorithm cannot know these non-contributing sub-grids in advance. However, using the `DropTol` feature, we can tell the interpolation function `spinterp` to neglect the sub-grids that do not contribute, and thus, save a significant amount of computing time. We consider the high-dimensional case $d = 100$. With the dimension-adaptive algorithm, the problem structure is automatically detected, and the function is successfully recovered using just $\mathscr{O}(d^2)$ function evaluations. For the interpolation domain, we use $[-d^2, d^2]$ in each dimension.

```
d = 100;
range = repmat([-d^2 d^2],d,1);
options = spset('DimensionAdaptive', 'on', ...
                'DimadaptDegree', 1, ...
                'FunctionArgType', 'vector', ...
                'RelTol', 1e-3, ...
                'MaxPoints', 40000);
z = spvals('trid',d,range,options);
```

We now evaluate the obtained interpolant, first without, and thereafter, with the `DropTol` feature set to the default value of $[0, 100 * \texttt{eps}]$ (absolute drop tolerance is zero, relative drop tolerance is $100 * \texttt{eps}$). We evaluate the interpolant at 100 random points, measure the time, the absolute error, and compare the timing results in a plot.

```
% Compute 100 randomly sampled points
p = 100;
rand('state', 0);
x = -d^2 + 2*d^2*rand(p,d);

% Compute exact function values
y = zeros(p,1);
for k = 1:p
  y(k) = trid(x(k,:));
end

xcell = num2cell(x,1);
tic;
% Compute interpolated function values, no dropped indices
ip1 = spinterp(z, xcell{:});
t1 = toc

% Perform purging of interpolant data
tic;
z = sppurge(z);
t2 = toc
tic;
% Compute interpolated function values
% Some indices dropped according to drop tolerance
ip2 = spinterp(z, xcell{:});
t3 = toc

% Compute relative errors
err_ndt = max(abs(y-ip1))/(z.fevalRange(2)-z.fevalRange(1))
err_wdt = max(abs(y-ip2))/(z.fevalRange(2)-z.fevalRange(1))
```

```
t1 =
    2.1393

t2 =
    0.0128

t3 =
    0.2933

err_ndt =
    0.0061

err_wdt =
    0.0061
```

The result is quite impressing: Without losing accuracy (which is no surprise considering the very low drop tolerance of $100 * \texttt{eps}$ compared to the relative error tolerance $1e-3$), for the 100 sampled points, a speedup by a factor of about 7 is achieved (including the cost of the sppurge function).

```
bar([NaN t1; t2 t3],'stacked');
legend('spurge', 'spinterp');
set(gca,'XTickLabel',{'without','with␣purging'});
ylabel('time␣[s]');
```

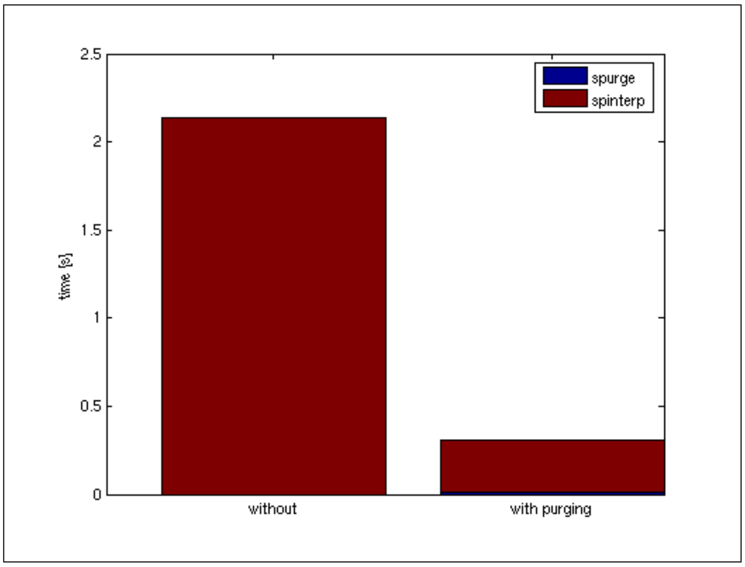

See Also spset.

spquad

Compute integral value of sparse grid interpolant.

Syntax

```
Q = spquad(Z)
```

Description

`Q = spquad(Z)` Computes the integral over the sparse grid domain. The sparse grid data must be given as a structure `Z` containing the hierarchical surpluses (computed with with `spvals`).

The following additional option is available with spquad that is set by adding a field to the structure `Z`:

- `selectOutput [ integer {1} ]` Set the output variable number if an interpolant with multiple output variables was constructed with `spvals`. This determines which ouput variable the integral is computed for.

Examples

Assume a sparse grid interpolant of the Matlab peaks function has been computed for the domain $[0,2]^2$ using the following commands:

```
options = spset('DimensionAdaptive', 'on', 'RelTol', 1e-4, ...
  'GridType', 'Chebyshev');
z = spvals(@(x,y) peaks(x,y), 2, [0,2; 0,2], options);
```

Then, we can compute the integral for this domain simply like this:

```
spquad(z)
```

```
  ans =
      9.9553
```

For comparison, let us compute the integral value with Matlab's `dblquad`:

```
dblquad(@(x,y) peaks(x,y), 0, 2, 0, 2)
```

```
  ans =
      9.9553
```

See Also `spvals`.

spset

Create/alter a sparse grid interpolation `OPTIONS` structure.

Syntax

```
OPTIONS = spset('NAME1',VALUE1,'NAME2',VALUE2,...)
OPTIONS = spset(OLDOPTS,'NAME1',VALUE1,...)
OPTIONS = spset(OLDOPTS,NEWOPTS)
```

Description

`spset` with no input arguments displays all property names and their possible values.

`OPTIONS = spset('NAME1',VALUE1,'NAME2',VALUE2,...)` creates an options structure `OPTIONS` in which the named properties have the specified values. Any unspecified properties have default values. It is sufficient to type only the leading characters that uniquely identify the

property. Case is ignored for property names.

`OPTIONS = spset(OLDOPTS,'NAME1',VALUE1,...)` alters an existing structure `OLDOPTS`.

`OPTIONS = spset(OLDOPTS,NEWOPTS)` combines an existing options structure `OLDOPTS` with a new options structure `NEWOPTS`. Any new properties overwrite corresponding old properties.

The properties configurable with `spset` are listed in Table 3.2 and Table 3.3.

Examples

Since `spset` offers many possibilities to alter the behavior of the sparse grid interpolant construction, we provide several typical examples in the following.

Example 1: Basic usage of spset

As an example for a typical task requiring the modification of the sparse grid options structure. we construct an interpolant with a specified number of function evaluations. The dimension-adaptive approach permits to do this in an elegant manner. The following code constructs an interpolant with about 100 nodes, since both `MinPoints` as well as `MaxPoints` are set to 100.

```
f = @(x,y) exp(x+y);
z = spvals(f, 2, [], spset('DimensionAdaptive', 'on', ...
                     'MinPoints', 100, 'MaxPoints', 100))
```

```
  z =
                 vals: {[129x1 double]}
             gridType: 'Clenshaw-Curtis'
                    d: 2
                range: []
          estRelError: 8.3520e-04
          estAbsError: 0.0053
           fevalRange: [1 7.3891]
           minGridVal: [0 0]
           maxGridVal: [1 1]
              nPoints: 129
            fevalTime: 0.0546
      surplusCompTime: 0.0058
              indices: [1x1 struct]
             maxLevel: [5 5]
        activeIndices: [5x1 uint32]
       activeIndices2: [13x1 uint32]
                    E: [1x19 double]
                    G: [19x1 double]
                   G2: [19x1 double]
         maxSetPoints: 5
             dimAdapt: 1
```

Table 3.2: Properties configurable with spset (part I).

Property	*Value {default}*	*Description*
GridType	{Clenshaw-Curtis} | Maximum | NoBoundary | Chebyshev | Gauss-Patterson	Sparse grid type and basis functions to use by spvals. For an illustration of the grid types, run cmpgrids.
RelTol	positive scalar {1e-2}	A relative error tolerance that applies to all hierarchical surpluses w^n of the current deepest level n of the sparse grid interpolation formula. The grid is further refined until all hierarchical surpluses are less than $\max(\texttt{RelTol}\cdot(\max(\texttt{fevalRange})-\min(\texttt{fevalRange})),\texttt{AbsTol})$. fevalRange contains all results evaluating FUN up to that point.
AbsTol	positive scalar {1e-6}	Absolute error tolerance, used by the error criterion stated under the property RelTol.
Vectorized	on | {off}	Indicates if FUN is available for vectorized evaluation. Vectorized coding of FUN can significantly reduce the computation time used by spvals. For an example using a vectorized function, see spdemo.
MinDepth	integer {2}	Minimum interpolation depth, specifies the minimum number of hierarchical interpolation levels N to compute. **Remark:** MinDepth has no effect if the dimension-adaptive grid refinement is switched on. An example is provided below.
MaxDepth	integer {8}	Maximum interpolation depth, specifies the maximum number of hierarchical interpolation levels N to compute. **Remark:** Since version 5.0, MaxDepth also applies to the dimension-adaptive algorithm. If MaxDepth is reached with respect to a coordinate direction, this direction is no longer refined further.
Variable Positions	1xD vector {[]}	Position of the ranges in the argument list when FUN is evaluated. By setting VariablePositions, spvals will evaluate FUN with respect to some of its input parameters, but not necessarily the first D ones. The actual position is assigned by providing the number in the input argument list of the function FUN. This number must be provided for each interpolation dimension. Therefore, the value of VariablePositions must be a 1xD array. Also see example below.
NumberOf Outputs	integer {1}	If FUN produces multiple outputs (where all must be scalar), indicate this here to perform the sparse grid computation for many output variables at once. Also see the example spdemovarout.m.
PrevResults	struct {[]}	Previous sparse grid data. An existing result structure obtained from spvals may be provided to further refine an existing sparse grid. Also see example below.
Function ArgType	{list} | vector	Indicates whether the objective function takes the input parameters as a comma-separated list (default) or as a vector.
KeepFunction Values	{off} | on	If this parameter is set, a structure field fvals is returned, containing a cell array with the function values at the sparse grid points.
KeepGrid	{off} | on	If this parameter is set, a structure field grid is returned, containing a cell array with the the sparse grid points.

Table 3.3: Properties configurable with spset (part II).

Property	*Value {default}*	*Description*
DimensionAdaptive	{off} | on	Dimension-adaptive grids try to adaptively find important dimensions and adjust the sparse grid structure accordingly. Especially in case of higher-dimensional problems, a dimension-adaptive strategy can significantly reduce the number of nodes required to get a good interpolant.
DimadaptDegree	positive scalar {0.9}	Fine-tuning parameter to alter the degree of dimensional adaptivity. A value of 1 places strong emphasis on the error estimates, and thus leads to strong dimensional adaptivity. A value of 0 disregards the error estimates, and constructs a conventional sparse grid based on the amount of work involved.
DegreeStrategy	{balancing} | depth	The **balancing** strategy balances the number of grid points generated according to the greedy, error estimate-based refinement rule compared to the number of points generated by the regular sparse grid refinement rule. E.g., a DimadaptDegree value of 0.9 results in around 90% of the grid points generated by the error estimate-based rule.<br>The **depth** strategy ensures that the maximum depth reached by the error estimate-based refinement in one dimension does not get too deep compared to the depth reached in other dimensions. This strategy was used by default prior to v5.1 of the toolbox, and is described [3, ch. 3].
MinPoints	integer {100}	This parameter only applies to dimension-adaptive sparse grids, and indicates the minimum number of support nodes (i.e., function evaluations to perform). An example is provided below.
MaxPoints	integer {10000}	This parameter only applies to dimension-adaptive sparse grids. The dimension-adaptive algorithm is aborted once the function evaluation count exceeds this number.
SparseIndices	{auto} | off | on	Manually turn the efficient sparse storage scheme (new feature since version 3.0) of the multi-index arrays on or off. The default switch auto uses the new scheme for the ClenshawCurtis, the Chebyshev, and the Gauss-Patterson grid, and the old (full) storage scheme from spinterp version 2.x for the Maximum and the NoBoundary grid (the sparse grid storage scheme is not supported for these two grid types).
DropTol	{auto} | off | 1x2 vector	During the sparse grid construction progress, the spvals algorithm may add sub-grids with hierarchical surpluses that are all close to 0 or of negligible magnitude compared to the surpluses of other sub-grids. In particular, this occurs when additive structure is present in the objective function. To increase performance of the spinterp algorithm, run the sppurge algorithm to mark sub-grids to be neglected where all (absolute) hierarchical surpluses are less than $\max(\texttt{relDropTol} * (\max(\texttt{fevalRange}) - \min(\texttt{fevalRange}), \texttt{absDropTol}))$. You may specify the absolute and the relative drop tolerance as a vector [absDropTol, relDropTol], or turn it off completely (= behavior of version 3.0 and earlier). The switch auto uses the values $\texttt{absDropTol} = 0$, $\texttt{relDropTol} = 100 * \texttt{eps}$, that is, by default, only a relative drop tolerance is used.
EnableDCT	{on} | off	Enables/disables the DCT-based algorithm when constructing the Chebyshev-Gauss-Lobatto type sparse grid.

Note that the MinPoints and MaxPoints properties only work for dimension-adaptive grids. If we want to construct a non-adaptive grid of a certain depth, the MinDepth and MaxDepth options can be used. Recall that the number of points of a regular sparse grid can be determined a priori with the spdim function.

```
n = 4; d=2;
z = spvals(f, 2, [], spset('MinDepth', n, 'MaxDepth', n))
```

```
z =
              vals: {[65x1 double]}
          gridType: 'Clenshaw-Curtis'
                 d: 2
             range: []
          maxLevel: 4
       estRelError: 0.0031
       estAbsError: 0.0201
        fevalRange: [1 7.3891]
        minGridVal: [0 0]
        maxGridVal: [1 1]
           nPoints: 65
         fevalTime: 0.1066
   surplusCompTime: 0.0029
           indices: [1x1 struct]
```

Example 2: Providing previous results

After computing an interpolant with a certain accuracy, it is often required to improve it further later on. Due to the hierarchical construction scheme, the previous results are not lost but can be passed to spvals for further refinement, as the following code illustrates.

```
f = @(x,y) exp(x+y);
z = [];
for n = 1:4
  z = spvals(f, 2, [], spset('MinDepth', n, 'MaxDepth', n, ...
                             'PrevResults', z));
  disp(['n␣=␣' num2str(z.maxLevel) ',␣estimated␣rel.␣error:␣', ...
        num2str(z.estRelError)]);
end
```

```
Warning: MaxDepth = 1 reached before accuracies
    RelTol = 0.01 or AbsTol = 1e-06 were achieved.
The current estimated relative accuracy is 0.62246.
n = 1, estimated rel. error: 0.62246
Warning: MaxDepth = 2 reached before accuracies
   RelTol = 0.01 or AbsTol = 1e-06 were achieved.
The current estimated relative accuracy is 0.17905.
n = 2, estimated rel. error: 0.17905
```

```
Warning: MaxDepth = 3 reached before accuracies
   RelTol = 0.01 or AbsTol = 1e-06 were achieved.
The current estimated relative accuracy is 0.011133.
n = 3, estimated rel. error: 0.011133
n = 4, estimated rel. error: 0.0031415
```

Example 3: Using the VariablePositions property

Consider the case of a function of four parameters, e.g.

$$f(a,b,x,y) = a(x^2+y^2)+b\exp(x+y).$$

Suppose that the parameters a and b are fixed to $a = 0.5$ and $b = 0.2$, and we wish to compute an approximation of f for $x,y \in [0,1]^2$. The default syntax of spvals would require the interpolated parameters to appear at the start of the argument list, i.e. would require an argument list `(x,y,a,b)` to enable the call `spvals(f,2,[],[],a,b)`.
By using `VariablePositions`, we can use the function as it is defined above, as the following code shows.

```
f = inline('a.*(x.^2+y.^2) + b.*exp(x+y)','a','b','x','y')
a = 0.5; b = 0.2;
options = spset('VariablePositions', [3 4]);
z = spvals(f,2,[],options,a,b)
```

```
f =
     Inline function:
     f(a,b,x,y) = a.*(x.^2+y.^2) + b.*exp(x+y)
z =
              vals: {[29x1 double]}
          gridType: 'Clenshaw-Curtis'
                 d: 2
             range: []
          maxLevel: 3
       estRelError: 0.0062
       estAbsError: 0.0142
        fevalRange: [0.2000 2.4778]
        minGridVal: [0 0]
        maxGridVal: [1 1]
           nPoints: 29
         fevalTime: 0.0508
   surplusCompTime: 0.0015
           indices: [1x1 struct]
```

Since the interpolation problem is two-dimensional, we assign a 1×2 vector to the `VariablePositions` property, indicating that the first parameter an interpolation of which is required is located at position 3 of the argument list of f, and the second one at position 4. Note that since the function f takes four input parameters, the remaining parameters are appended to the argument list of `spvals` after the options argument.

See Also spget, spvals.

---

spsurfun

Evaluate the sparse grid interpolant at a single point.
spsurfun is provided for conveniece to be used as an alternative to spinterp, where the point Y to be evaluated is given as a row or column vector. This functional form is often adopted by multivariate optimization algorithms in Matlab.
Note that this form allows the evaluation of the sparse grid interpolant at a single point only. Therefore, It is recommended to use spinterp instead if multiple evaluations of the interpolant can be performed simultaneously.

Syntax

```
IP = spsurfun(Y,Z)
[IP,IPGRAD] = spsurfun(Y,Z)
```

Description

IP = spsurfun(Y,Z) Computes the interpolated value IP at the single point [Y1, ..., YD] for the sparse grid interpolant Z.

[IP,IPGRAD] = spsurfun(...) Computes the interpolated value IP and the gradient vector IPGRAD.

Two additional options are available with spsurfun that are set by adding a field to the structure Z:

- selectOutput [integer 1] Set the output variable number if an interpolant with multiple output variables was constructed with spvals.
- continuousDerivatives ['on' | 'off'] Enable augmented continuous derivatives for the Clenshaw-Curtis grid.

Examples

The following code shows how to use spsurfun. Note that as opposed to the spinterp syntax, the second argument is the sparse grid interpolant, not the first one.

```
f = inline('x.^2 + y.^2 - 2.*z');
z = spvals(f,3,[],spset('GridType','Chebyshev'));
[ip,ipgrad] = spinterp(z, 0.5, 0.2, 0.2)
[ip,ipgrad] = spsurfun([0.5, 0.2, 0.2], z)
```

```
in =
   -0.1100
ipgrad =
    [3x1 double]
ip =
   -0.1100
ipgrad =
    1.0000
    0.4000
   -2.0000
```

See the help page on sparse grid optimization for an example where `spsurfun` is used with an optimization method from Mathwork's Optimization Toolbox.

See Also `spinterp`.

spvals

Construct a sparse grid interpolant.

Syntax

```
Z = spvals(FUN,D)
Z = spvals(FUN,D,RANGE)
Z = spvals(FUN,D,RANGE,OPTIONS)
Z = spvals(FUN,D,RANGE,OPTIONS,P1,P2, ...)
```

Description

`Z = spvals(FUN,D)` Compute the sparse grid representation `Z` for multi-linear sparse grid interpolation of the function `FUN`. The grid is computed over the d-dimensional unit cube $[0,1]^D$.

`Z = spvals(FUN,D,RANGE)` In addition to the syntax above, the interpolation box dimensions may be specified. `RANGE` is a `2xD` array, e.g. to compute the sparse grid representation over the domain $[0,1] \times [2,4] \times [1,5]$ of `FUN`, `RANGE` must be `[0,1; 2,4; 1,5]`. If `RANGE` is empty (=`[]`), it is assumed to be $[0,1]^D$.

`Z = spvals(FUN,D,RANGE,OPTIONS)` Computes the sparse grid representation as above, but with default interpolation properties replaced by values in `OPTIONS`, an argument created with `spset`.

`Z = spvals(FUN,D,RANGE,OPTIONS,P1,P2, ...)` Passes the parameters `P1,P2,...` to the objective function `FUN`.

Examples

The following examples demonstrate the generation of sparse grid interpolants under a variety of different parameters. The extensive configurability of spvals is achieved via the `spset` function. Additional examples of constructing interpolants of external functions, models with several output parameters, ODEs, etc. are provided in the Advanced Topics section of this document.
We first define the test function. In the examples below, we use Branin's test function:

$$f_{\mathrm{BR}}(x_1,x_2) = \left(\frac{5}{\pi}x_1 - \frac{5.1}{4\pi^2}x_1^2 + x_2 - 6\right)^2 + 10\left(1 - \frac{1}{8\pi}\right)\cos x_1 + 10.$$

We set the dimension to $d = 2$, and the interpolation domain to `range = [-5,10; 0,15]`.

```
fun = inline(['(5/pi*x-5.1/(4*pi^2)*x.^2+y-6).^2␣+␣' ...
             '10*(1-1/(8*pi))*cos(x)+10']);
d = 2;
range = [-5, 10; 0, 15];
```

Now, we compute a regular (i.e. non-adaptive) sparse grid interpolant of fun using the default settings of the Sparse Grid Interpolation toolbox. This will compute a piecewise linear interpolant at the Clenshaw-Curtis sparse grid.

```
z1 = spvals(fun, d, range)
```

```
z1 =

               vals: {[145x1 double]}
           gridType: 'Clenshaw-Curtis'
                  d: 2
              range: [2x2 double]
           maxLevel: 5
        estRelError: 0.0087
        estAbsError: 2.6622
         fevalRange: [1.3697 308.1291]
         minGridVal: [0.1250 0.7500]
         maxGridVal: [0 0]
            nPoints: 145
          fevalTime: 0.2437
    surplusCompTime: 0.0040
            indices: [1x1 struct]
```

For comparison, we now compute two additional interpolants, one being a regular Chebyshev-Gauss-Lobatto grid, the other one being a dimension-adaptive sparse grid interpolant of the same grid type. To do this, we must pass an according options structure to the `spvals` routine. We do not have to store this options structure- it is possible to pass a structure generated on-the-fly to the function.

```
z2 = spvals(fun, d, range, spset('GridType', 'Chebyshev'))
z3 = spvals(fun, d, range, spset('GridType', 'Chebyshev', ...
                                 'DimensionAdaptive', 'on', ...
```

```
                                   'DimAdaptDegree', 1, ...
                                   'MinPoints', 20))
```

```
z2 =
                 vals: {[65x1 double]}
             gridType: 'Chebyshev'
                    d: 2
                range: [2x2 double]
             maxLevel: 4
          estRelError: 0.0095
          estAbsError: 2.9017
           fevalRange: [2.5620 308.1291]
           minGridVal: [0.5000 0.2222]
           maxGridVal: [0 0]
              nPoints: 65
            fevalTime: 0.1211
      surplusCompTime: 0.0225
              indices: [1x1 struct]

z3 =
                 vals: {[29x1 double]}
             gridType: 'Chebyshev'
                    d: 2
                range: [2x2 double]
          estRelError: 0.0095
          estAbsError: 2.9017
           fevalRange: [2.7065 308.1291]
           minGridVal: [0.5000 0.1464]
           maxGridVal: [0 0]
              nPoints: 29
            fevalTime: 0.0468
      surplusCompTime: 0.0094
              indices: [1x1 struct]
             maxLevel: [4 2]
        activeIndices: [3x1 uint32]
       activeIndices2: [9x1 uint32]
                    E: [1x9 double]
                    G: [9x1 double]
                   G2: [9x1 double]
         maxSetPoints: 4
             dimAdapt: 1
```

The following code generates a plot comparing the three interpolants. Furthermore, the error is plotted compared to the original function.

```
z = {z1, z2, z3};
for k = 1:3
  f_z = @(x,y) spinterp(z{k}, x, y);
```

```
  error_z = @(x,y) fun(x,y) - spinterp(z{k}, x, y);
  subplot(2,3,k);
  ezmesh(f_z, [range(1,:),range(2,:)]);
  title(['interpolant␣z_' num2str(k)]);
  view(20,30);
  subplot(2,3,k+3);
  ezmesh(error_z, [range(1,:),range(2,:)]);
  title(['error␣of␣z_' num2str(k)]);
  view(20,30);
end
```

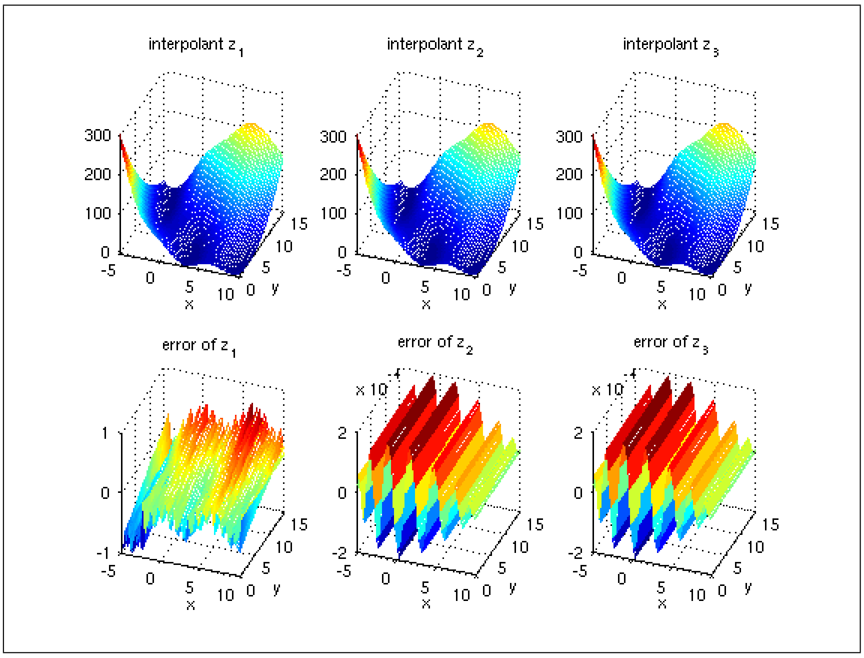

See Also spinterp, spset.

License

SPARSE GRID INTERPOLATION TOOLBOX - LICENSE

Copyright (c) 2006 W. Andreas Klimke, Universitaet Stuttgart. Copyright (c) 2007-2008 W. A. Klimke. All Rights Reserved. All Rights Reserved.

Permission is hereby granted, free of charge, to any person obtaining a copy of this software and associated documentation files (the "Software"), to deal in the Software without restriction, including without limitation the rights to use, copy, modify, merge, publish, distribute, sublicense, and/or sell copies of the Software, and to permit persons to whom the Software is furnished to do so, subject to the following conditions:

The above copyright notice and this permission notice shall be included in all copies or substantial portions of the Software.

THE SOFTWARE IS PROVIDED "AS IS", WITHOUT WARRANTY OF ANY KIND, EXPRESS OR IMPLIED, INCLUDING BUT NOT LIMITED TO THE WARRANTIES OF MERCHANTABILITY, FITNESS FOR A PARTICULAR PURPOSE AND NONINFRINGEMENT. IN NO EVENT SHALL THE AUTHORS OR COPYRIGHT HOLDERS BE LIABLE FOR ANY CLAIM, DAMAGES OR OTHER LIABILITY, WHETHER IN AN ACTION OF CONTRACT, TORT OR OTHERWISE, ARISING FROM, OUT OF OR IN CONNECTION WITH THE SOFTWARE OR THE USE OR OTHER DEALINGS IN THE SOFTWARE.

Bibliography

[1] H.-J. Bungartz and M. Griebel. Sparse grids. *Acta Numerica*, 13:147–269, 2004. 11

[2] Andreas Klimke and Barbara Wohlmuth. Algorithm 847: spinterp: Piecewise multilinear hierarchical sparse grid interpolation in matlab. *ACM Transactions on Mathematical Software*, 31(4), 2005. 11

[3] Andreas Klimke. *Uncertainty modeling using fuzzy arithmetic and sparse grids*. PhD thesis, Universität Stuttgart, Shaker Verlag, Aachen, 2006. 11, 14, 16, 19, 85

[4] Volker Barthelmann, Erich Novak, and Klaus Ritter. High dimensional polynomial interpolation on sparse grids. *Adv. Comput. Math.*, 12(4):273–288, 2000. 12, 13, 14

[5] Hans-Joachim Bungartz. *Finite Elements of Higher Order on Sparse Grids*. Shaker Verlag, Aachen, 1998. 12, 16

[6] T.N.L. Patterson. The optimum addition of points to quadrature formulae. *Mathematics of Computation*, 22(104):847–856+s21–s31, 1968. 14

[7] T. Gerstner and M. Griebel. Numerical integration using sparse grids. *Numerical Algorithms*, 18(3–4):209–232, 1998. 14, 32

[8] Andreas Klimke. Efficient construction of hierarchical polynomial sparse grid interpolants using the fast discrete cosine transform. Technical Report IANS Preprint 2006/007, Universität Stuttgart, 2006. 14

[9] Markus Hegland. Adaptive sparse grids. In K. Burrage and Roger B. Sidje, editors, *Proceedings of the 2001 International conference on Computational Techniques and Applications, University of Queensland*, volume 44 of *ANZIAM Journal*, pages C335–C353, 2003. 15

[10] T. Gerstner and M. Griebel. Dimension-adaptive tensor-product quadrature. *Computing*, 71(1):65–87, 2003. 15, 16, 19

[11] James J. Buckley, Esfandiar Eslami, and Thomas Feuring. *Fuzzy Mathematics in Economics and Engineering*. Physica-Verlag, Heidelberg, Germany, 2002. 49

Keyword Index

AbsTol, **84**

cmpgrids, **55**, 55
continuousDerivatives, 27

DegreeStrategy, **85**
DimadaptDegree, **85**
DimensionAdaptive, **85**
Display, **77**
DropTol, **85**

EnableDCT, **85**

FunctionArgType, **84**

GridType, **84**

KeepFunctionValues, **84**
KeepGrid, **84**

MaxDepth, **84**
Maximize, **77**
MaxIter, **77**
MaxPoints, **85**
Method, **77**
MinDepth, **84**
Minimize, **77**
MinPoints, **85**

NumberOfOutputs, **84**
NumStarts, **77**

OptimsetOptions, **77**

plotgrid, 10, **56**, 57
plotindices, 21, **57**, 59
PrevResult, **77**
PrevResults, **84**

RelTol, **84**

selectOutput, 23, 52
SparseIndices, **85**
spcgsearch, 39, 40, **59, 60**, 61, 78
spcompsearch, 38, **62**, 63
spdim, **64**, 65
spfminsearch, **65**, 66
spget, **68**, 69
spgrid, **69**, 70
spinit, **71**
spinterp, 9, 10, 18, 23, 26–28, 43–45, 52, 59, **71**, 72, 80, 88, 91, 92
spmultistart, **73**, 74
spoptimget, **75**, 75, 76
spoptimset, 40, 61, 63, 66, 75, **76**, 78
sppurge, 40, 43, **78**, 80
spquad, 33, 36, **81**, 82
spset, 17, 20–23, 25, 28, 33, 35, 38–40, 42, 43, 46–49, 51, 53, 57, 58, 60, 63, 65, 66, 68, 74, 78, 79, **82**, 82, 83, 86–88, 90
spsurfun, 41, **88**, 88
spvals, 9, 17, 20–23, 25, 28, 33, 35, 38–40, 42, 46–49, 52, 53, 58, 61, 63, 66, 71, 74, 78, 79, 82, 83, 86–88, **89**, 90
StartPoint, **77**

TestCorners, **77**
TolFun, **77**

`TolX`, **77**

`VariablePositions`, **84**
`Vectorized`, **84**
