# Supplementary material for: Multiple Model-Informed Open-Loop Control of Uncertain Intracellular Signaling Dynamics
Source: PLoS Comput Biol. 2014 Apr 10;10(4):e1003546. doi: 10.1371/journal.pcbi.1003546 (PMC3983080; doi:10.1371/journal.pcbi.1003546)
Supplement: Dataset S1 — Matlab code for proposed control algorithm and prediction models. Contains all Matlab code necessary to implement the proposed adaptive weighted multiple-model predictive control algorithm, as well as code for the prediction models. (ZIP) [file pcbi.1003546.s001.zip › AW_MMPC/spinterp_v5.1.1/help/advanced_topics.html]

Advanced topics (Sparse Grid Interpolation Toolbox)


|  |  |
| --- | --- |
| **Sparse Grid Interpolation Toolbox** |  |

# Advanced topics

The following chapter introduces advanced features and describes how to use the toolbox for solving real-world problems.

|  |  |
| --- | --- |
| Degree of dimensional adaptivity | Balancing greedy vs. conservative refinement for dimension-adaptive interpolants |
| Multiple output variables | Interpolating objective models with multiple output variables |
| Derivatives | Computing the gradient vector of the sparse grid interpolant at arbitrary points |
| Integration | Numerical integration using sparse grids |
| Optimization | Discusses the search methods available to efficiently perform an optimization of the sparse grid interpolant |
| Improving performance | Provides an overview on how to achieve maximum performance of the Sparse Grid Interpolation Toolbox |
| Interfacing concepts | Concepts of how to best interface existing Matlab models with `spvals` for the interpolant construction |
| Approximating ODEs | Recovering ODEs with one or several time-dependent outputs for a range of initial conditions or parameters |
| External models | Interfacing `spvals` with models not implemented in Matlab |

|  |  |  |  |  |
| --- | --- | --- | --- | --- |
|  | Sparse Grid Interpolation product page |  | Degree of Dimensional Adaptivity |  |
