# Supplementary material for: Multiple Model-Informed Open-Loop Control of Uncertain Intracellular Signaling Dynamics
Source: PLoS Comput Biol. 2014 Apr 10;10(4):e1003546. doi: 10.1371/journal.pcbi.1003546 (PMC3983080; doi:10.1371/journal.pcbi.1003546)
Supplement: Dataset S1 — Matlab code for proposed control algorithm and prediction models. Contains all Matlab code necessary to implement the proposed adaptive weighted multiple-model predictive control algorithm, as well as code for the prediction models. (ZIP) [file pcbi.1003546.s001.zip › AW_MMPC/spinterp_v5.1.1/help/bibliography.html]

Bibliography (Sparse Grid Interpolation Toolbox)


|  |  |
| --- | --- |
| **Sparse Grid Interpolation Toolbox** |  |

# Selected references

|  |  |
| --- | --- |
| [1] | H.-J. Bungartz and M. Griebel. Sparse grids. *Acta Numerica* 13:147-269, 2004. |
| [2] | A. Klimke and B. Wohlmuth. Algorithm 847: spinterp: Piecewise multilinear hierarchical sparse grid interpolation in MATLAB. *ACM Transactions on Mathematical Software*, 31(4), 2005. |
| [3] | A. Klimke. *Uncertainty Modeling using Fuzzy Arithmetic and Sparse Grids*, PhD Thesis, Universität Stuttgart, Shaker Verlag, Aachen, 2006. |
| [4] | V. Barthelmann, E. Novak, and K. Ritter. High dimensional polynomial interpolation on sparse grids. *Adv. Comput. Math.*, 12(4):273-288, 2000. |
| [5] | H.-J. Bungartz. *Finite Elements of Higher Order on Sparse Grids.* Shaker Verlag, Aachen, 1998. |
| [6] | A. Klimke. Efficient construction of hierarchical polynomial sparse grid interpolants using the fast discrete cosine transform. Technical Report IANS Preprint 2006/007, Universität Stuttgart, 2006. |
| [7] | M. Hegland. Adaptive sparse grids. In K. Burrage and R. B. Sidje, editors, *Proceedings of the 2001 International conference on Computational Techniques and Applications*, University of Queensland, volume 44 of ANZIAM Journal, pages C335-C353, 2003. 59 |
| [8] | T. Gerstner and M. Griebel. Dimension-adaptive tensor-product quadrature. *Computing*, 71(1):65-87, 2003. |
| [9] | T.N.L. Patterson. The Optimum Addition of Points to Quadrature Formulae. *Mathematics of Computation*, 22(104):847-856+s21-s31, 1968. |
| [10] | T. Gerstner and M. Griebel. Numerical Integration using Sparse Grids *Numerical Algorithms*, 18(3-4):209-232, 1998. |

|  |  |  |  |  |
| --- | --- | --- | --- | --- |
|  | Dimensional adaptivity |  | Sparse Grid Interpolation product page |  |
