# Supplementary material for: Multiple Model-Informed Open-Loop Control of Uncertain Intracellular Signaling Dynamics
Source: PLoS Comput Biol. 2014 Apr 10;10(4):e1003546. doi: 10.1371/journal.pcbi.1003546 (PMC3983080; doi:10.1371/journal.pcbi.1003546)
Supplement: Dataset S1 — Matlab code for proposed control algorithm and prediction models. Contains all Matlab code necessary to implement the proposed adaptive weighted multiple-model predictive control algorithm, as well as code for the prediction models. (ZIP) [file pcbi.1003546.s001.zip › AW_MMPC/spinterp_v5.1.1/help/cmpgrids.html]

cmpgrids :: (Sparse Grid Interpolation Toolbox)


|  |  |
| --- | --- |
| **Sparse Grid Interpolation Toolbox** |  |

# cmpgrids

Compare the available sparse grid types.

## Syntax

`cmpgrids`  
`cmpgrids(N)`  
`cmpgrids(N,D)`

## Description

`cmpgrids` Compares the maximum-norm-based grid, the no-boundary-nodes grid, the Clenshaw-Curtis grid, the Chebyshev-Gauss-Lobatto grid, and the Gauss-Patterson grid in dimension `D = 2` and level `N = 3`.

`cmpgrids(N)` Compares the grids for level `N`.

`cmpgrids(N,D)` Compares the grids in dimension `D`. Permitted are only the values `D = 2` or `D = 3`.

## Examples

The following statement plots the four available sparse grids with level `N = 3` in two dimensions, producing the following graph.

```
cmpgrids(3,2);
```

## See Also

`plotgrid`,
`plotindices`,
`spgrid`.

|  |
| --- |
|  |
