# Supplementary material for: Multiple Model-Informed Open-Loop Control of Uncertain Intracellular Signaling Dynamics
Source: PLoS Comput Biol. 2014 Apr 10;10(4):e1003546. doi: 10.1371/journal.pcbi.1003546 (PMC3983080; doi:10.1371/journal.pcbi.1003546)
Supplement: Dataset S1 — Matlab code for proposed control algorithm and prediction models. Contains all Matlab code necessary to implement the proposed adaptive weighted multiple-model predictive control algorithm, as well as code for the prediction models. (ZIP) [file pcbi.1003546.s001.zip › AW_MMPC/spinterp_v5.1.1/help/demos.html]

Demos (Sparse Grid Interpolation Toolbox)


|  |  |
| --- | --- |
| **Sparse Grid Interpolation Toolbox** |  |

# Demos

Several demos are avaible that illustrate the features of the toolbox. Click here to open the Demos tab in the help navigator.

|  |
| --- |
|  |
