# Supplementary material for: Multiple Model-Informed Open-Loop Control of Uncertain Intracellular Signaling Dynamics
Source: PLoS Comput Biol. 2014 Apr 10;10(4):e1003546. doi: 10.1371/journal.pcbi.1003546 (PMC3983080; doi:10.1371/journal.pcbi.1003546)
Supplement: Dataset S1 — Matlab code for proposed control algorithm and prediction models. Contains all Matlab code necessary to implement the proposed adaptive weighted multiple-model predictive control algorithm, as well as code for the prediction models. (ZIP) [file pcbi.1003546.s001.zip › AW_MMPC/spinterp_v5.1.1/help/derivatives.html]

Derivatives (Sparse Grid Interpolation Toolbox)


|  |  |
| --- | --- |
| **Sparse Grid Interpolation Toolbox** |  |

# Derivatives

One of the primary purposes of sparse grid interpolation is the construction of surrogate functions for local or global optimization. While some optimization methods work well using function values only, many efficient algorithms exist that require computation of the gradient. With the Sparse Grid Interpolation Toolbox, it is possible to obtain gradients of the interpolants directly - up to floating point accuracy - as opposed to approximating them numerically, such as by finite differences. This is demonstrated in the following.

## Contents

- How to obtain the derivatives?
- Derivatives of piecewise multilinear interpolants
- Augmented derivatives to achieve continuity
- Derivatives of polynomial interpolants
- Approximation quality
- Computational cost

## How to obtain the derivatives?

Computing derivatives is extremely simple. One just calls the method spinterp, but instead of a single left-hand argument, one uses the syntax [ip,ipgrad] = spinterp(z,x1,...,xn). This returns not only the function value at the point(s) (x1,...,xn), but also the complete gradient vector(s). Please see the help page of spinterp for further details (or the examples provided below).

It is important to note that the entire procedure of computing the hierarchical surpluses of the sparse grid interpolant with
spvals remains the same, regardless of whether derivatives are desired or not. Also, purging of the interpolant (see sppurge) can be performed in the usual manner if desired. This makes using derivative information very flexible, and it can be decided
ad-hoc, well after interpolant construction (for example, when different optimization algorithms are applied), whether to
use derivatives or not.

Furthermore, the derivatives computed by spinterp are **not** additional approximations of the derivatives of the original function, but rather, the **exact** derivatives of the interpolant (up to floating point accuracy). The advantage of this approach is that **no additional memory is required** to store derivative information. The derivatives are computed on-the-fly by efficient algorithms.

## Derivatives of piecewise multilinear interpolants

We start with derivatives of piecewise multilinear Clenshaw-Curtis Sparse Grid Interpolants. Deriving a piecewise linear function
leads to piecewise constant derivatives with respect to the variable that the function is differentiated for. Since the interpolant
is non-differentiable at the kinks, the left-sided (or right-sided) derivative is computed at these points only. The following
example in two dimensions illustrates the nature of the derivatives.

First, we define the example objective function. We also define its derivatives (this is only for comparison to give an idea
of the quality of the computed derivatives).

```
% Define function and its derivatives
f = inline('1./(cos(2*x).^2 + sin(y).^2 + 1) + 0.2*y');
fdx = inline('4*cos(2*x).*sin(2*x) ./ (cos(2*x).^2 + sin(y).^2 + 1).^2');
fdy = inline('-2*cos(y).*sin(y) ./ (cos(2*x).^2 + sin(y).^2 + 1).^2 + 0.2');
```

Next, we compute the interpolant. In this case, using the regular Clenshaw-Curtis sparse grid. We limit the sparse grid depth
to 4 here (i.e., A\_{q,d}(f) = A\_{4+2,2}(f) is computed).

```
d = 2;
maxDepth = 4;
options = spset('Vectorized','on','SparseIndices','off', ...
  'MaxDepth', maxDepth);
z = spvals(f,d,[],options);
```

```
Warning: MaxDepth = 4 reached before accuracies RelTol = 0.01 or AbsTol = 1e-06 were achieved.
The current estimated relative accuracy is 0.02877.
```

Our aim here is to plot the derivatives. Therefore, we define a suitable grid, and set up an array for the vectorized evaluation
of the interpolant and its derivatives. We set up the grid such that the jumps of the derivative will be clearly visible.

```
np = 2^double(z.maxLevel)+1;
x = linspace(0,1,np);
xstep = zeros(1,(np-1)*2);
xstep(1:2:end-1) = x(1:end-1);
xstep(2:2:end) = x(2:end) - eps;
[x,y] = ndgrid(xstep);
```

Next, we evaluate the interpolant at the grid points. As result, ipgrad will contain an array of the shape of the input arrays x and y, with the difference that it is a cell array instead of a double array, where each cell contains the entire gradient at the
point of the corresponding array entry of x and y.

```
[ip,ipgrad] = spinterp(z,x,y);
```

For plotting, it is convenient to convert the data returned as a cell array back to a double array. This is achieved by the
following statements. We extract the derivatives with respect to y here.

```
% Convert returned cell array to matrix
ipgradmat = cell2mat(ipgrad);
% Get the derivatives with respect to y
ipdy = ipgradmat(2:d:end,:);
```

Similarly, we can get all derivatives with respect to x with the command

```
ipdx = ipgradmat(1:d:end,:);
```

This approach of transforming the cell array to a double array can be easily extended to other dimensions.

Finally, we plot the obtained derivatives next to the exact derivatives.

```
subplot(1,2,1,'align');
surf(x,y,fdy(x,y));
view(250,50); xlabel('x'); ylabel('y'); light;
title('Exact: {\partial}f / {\partial}y');
subplot(1,2,2,'align');
surf(x,y,ipdy);
view(250,50); xlabel('x'); ylabel('y'); light;
title('Approx.: {\partial}A^{CC}_{6,2}(f) / {\partial}y (piecewise const. w.r.t. y)');
```

## Augmented derivatives to achieve continuity

Obtaining the derivatives of the interpolant is usually not the primary goal, but rather, serves a secondary purpose. For
instance, in an optimization procedure, one is not interested in the derivatives per se. Instead, the gradient vector enters
an iterative procedure to achieve the primary goal, which is to numerically compute a local optimizer. Unfortunately, the
discontinuous derivatives of a piecewise multilinear interpolant have a serious drawback: the first order optimality condition
grad(f)=0 cannot be fulfilled. Instead, the sign of the gradient components will oscillate about the optimizer of the continuous
interpolant, resulting in slow convergence.

To overcome this limitation, the Sparse Grid Interpolation Toolbox offers a powerful alternative to computing the exact derivatives
of a piecewise multilinear interpolant. By setting a simple flag, augmented derivatives can be computed that artificially
enforce continuity. This is achieved by linear interpolation with respect to the derived variable.

Let us consider an example (we use the same test function and interpolant from above).

First, we re-define the evaluation grid (there are no more jumps to emphasize).

```
np = 2^double(z.maxLevel+1)+1;
x = linspace(0,1,np);
[x,y] = ndgrid(x);
```

Prior to evaluating the interpolant, we set the flag continuousDerivatives = 'on'.

```
z.continuousDerivatives = 'on';
```

Computing interpolated values and gradients of the sparse grid interpolant is done as before with the command

```
[ip,ipgrad] = spinterp(z,x,y);
```

Finally, we generate the plot. Compare the plot to the previous one. Note that the derivatives are now continuous.

```
% Convert returned cell array to matrix
ipgradmat = cell2mat(ipgrad);

% Get the derivatives with respect to y
ipdy = ipgradmat(2:d:end,:);

% Plot exact derivatives and derivatives of interpolant
subplot(1,2,1,'align');
surf(x,y,fdy(x,y));
view(250,50); xlabel('x'); ylabel('y'); light;
title('Exact: {\partial}f / {\partial}y');
subplot(1,2,2,'align');
surf(x,y,ipdy);
view(250,50); xlabel('x'); ylabel('y'); light;
title('Approx.: {\partial}A^{CC}_{6,2}(f) / {\partial}y');
```

To conclude this section: If piecewise multilinear sparse grid interpolants (the Clenshaw-Curtis grid) are used, augmented
derivatives can help improving efficiency when solving optimization problems with methods that require computation of the
gradient.

## Derivatives of polynomial interpolants

The Chebyshev-Gauss-Lobatto (CGL) sparse grid uses globally defined polynomial basis functions. These basis functions are
infinitely smooth, and thus, the derivatives are infinitely smooth, too. The Sparse Grid Interpolation Toolbox offers efficient
algorithms involving barycentric interpolation and the discrete cosine transform to compute gradients, with excellent numerical
stability.

Consider the following example. Using the test function from above, we compute a CGL-type sparse grid interpolant (again with
maxDepth = 4).

```
maxDepth = 4;
options = spset('Vectorized','on','SparseIndices','off', ...
  'MaxDepth', maxDepth, 'GridType', 'Chebyshev');
z = spvals(f,d,[],options);
```

```
Warning: MaxDepth = 4 reached before accuracies RelTol = 0.01 or AbsTol = 1e-06 were achieved.
The current estimated relative accuracy is 0.020306.
```

The remaining code evaluates the interpolant and its derivatives at the full grid and creates the plot, just as above.

```
[ip,ipgrad] = spinterp(z,x,y);
ipgradmat = cell2mat(ipgrad);
ipdy = ipgradmat(2:d:end,:);
subplot(1,2,1,'align');
surf(x,y,fdy(x,y));
view(250,50); xlabel('x'); ylabel('y'); light;
title('Exact: {\partial}f / {\partial}y');
subplot(1,2,2,'align');
surf(x,y,ipdy);
view(250,50); xlabel('x'); ylabel('y'); light;
title('Approx.: {\partial}A^{CC}_{6,2}(f) / {\partial}y');
```

## Approximation quality

Although we see the main application of computing derivatives of sparse grid interpolants in obtaining gradients during an
optimization algorithm, it is interesting to investigate the approximation quality with respect to the derivatives of the
original function.

This is illustrated by the example spcomparederiv.m that plots an approximation of the error in the maximum norm by computing the maximum absolute error of the derivatives for
the six test functions of Genz (see testderivatives.m) at 100 randomly sampled points. The plot presented below is for dimension d = 3.

```
% Reset random number generator (to generate reproducible results)
rand('state',0);
% Run the demo.
spcomparederiv;
```

The legend indicates the three types of derivatives: discontinuous (HCC grid), augmented continuous (HCC grid), and smooth (HCGL grid).

**Remark:** For the original functions labeled 'continuous' and 'discontinuous', single-sided derivatives are computed at the points
where the function is not continuously differentiable. Note that the approximations of the derivatives of both of these two functions do not converge, for the following reasons:

- Since the **discontinuous** function itself cannot be approximated by a continuous sparse grid interpolant in the first place, the approximations of the derivatives will not converge, either.- What is less obvious is that the derivatives of the **continuous** function **cannot** be successfully approximated for the whole considered box, either. Although the plot labelled 'continuous' appears to suggest slow convergence, **convergence in the maximum norm is not achieved** in regions close to the kink(s). However, this/these region(s) becomes smaller with increasing number of support nodes. The decreasing size of the non-converging region(s) close to the kink(s) explain(s) the decay of the absolute error in the plot: It becomes less likely that any of the 100 randomly sampled points are placed here.

## Computational cost

### Cost for the piecewise multilinear Clenshaw-Curtis sparse grid

The gradient vector of the piecewise multilinear Clenshaw-Curtis sparse grid interpolant can be obtained at a very small additional cost. The following two plots illustrate that this additional cost for computing either the exact derivatives or the augmented continuous derivatives amounts to just a small factor that is almost independent of the problem dimension.

 

### Cost for the polynomial Chebyshev-Gauss-Lobatto sparse grid

Due to the more sophisticated algorithms required in the polynomial case, the additional cost of computing the gradients is considerably higher compared to a mere interpolation of function values. However, as the dimension increases, the additional cost decreases, as fewer subgrids will require a derivative computation (many subgrids are lower-dimensional than the final interpolant, and thus, must not be differentiated with respect to the dimensions that are omitted).

Thus, the performance is very competitive especially in higher dimensions. For instance, consider applying numerical differentiation instead (which you can easily do alternatively). This would require `d + 1` interpolant evaluations to compute the gradient if single-sided differences are used, or even `2d + 1` if the more accurate centered difference formula is used.

 

**Remark:** We produced the previous graphs with the example function `timespderiv.m`, which is included in the toolbox (see demos). The test was performed using Matlab R14 SP3 running on a Linux 2.6.15 machine equipped with one AMD Athlon 1.5 GHz CPU and 512 MB of memory.

|  |  |  |  |  |
| --- | --- | --- | --- | --- |
|  | Multiple outputs |  | Integration |  |
