# Supplementary material for: Multiple Model-Informed Open-Loop Control of Uncertain Intracellular Signaling Dynamics
Source: PLoS Comput Biol. 2014 Apr 10;10(4):e1003546. doi: 10.1371/journal.pcbi.1003546 (PMC3983080; doi:10.1371/journal.pcbi.1003546)
Supplement: Dataset S1 — Matlab code for proposed control algorithm and prediction models. Contains all Matlab code necessary to implement the proposed adaptive weighted multiple-model predictive control algorithm, as well as code for the prediction models. (ZIP) [file pcbi.1003546.s001.zip › AW_MMPC/spinterp_v5.1.1/help/dimadaptdegree.html]

Degree of Dimensional Adaptivity (Sparse Grid Interpolation Toolbox)


|  |  |
| --- | --- |
| **Sparse Grid Interpolation Toolbox** |  |

# Degree of Dimensional Adaptivity

When dealing with high-dimensional problems, an "exhaustive" exploration of the objective function with a standard sparse grid becomes too expensive, since too many points are generated. Therefore, dimension-adaptive sparse grids were introduced that adaptively refine the interpolant with respect to dimensions that are most important, determined by error indicators. However, there is still one problem with this approach: due to the fact that the error indicator is a measure computed from hierarchical surpluses at a few points only, it can happen that the error indicator understimates the actual error with respect to some dimensions. As a consequence, the interpolant may no longer be further refined in these dimensions. In other words, the global convergence property of sparse grid interpolants for the before-mentioned classes of functions is lost for purely greedy dimension-adaptive refinement.

The solution to this problem is to take a "middle ground" approach by introducing an additional parameter, the degree of dimensional adaptivity [8], that lets the user gradually shift emphasis between greedy (= purely adaptive) and conservative (= standard sparse grid) refinement.

## *Degree balancing* approach

Version 5.1 of the toolbox introduces a simple yet very powerful new strategy to the degree of dimensional adaptivity called *degree balancing*, replacing the previous approach based on the interpolation depth described in [3, ch. 3]. The new approach is decribed in the following.

### Definition of the degree of dimensional adaptivity

As a preliminary, we define the actual degree of dimensional adaptivity as the ratio `r` = `nAdaptPoints` / `nTotalPoints`, where `nAdaptPoints` denotes the number of points added according to a greedy, purely dimension-adaptive refinement rule, and `nTotalPoints` denotes the total number of sparse grid points. We assume that the remaining points `(nTotalPoints - nAdaptPoints)` are constructed by the standard sparse grid refinement rule.

### Specifying the target dimensional adaptivity degree

The user may specify a target degree of dimensionsonal adaptivity using the `DimadaptDegree` parameter of the sparse grid options structure.

### Refinement rule

During interpolant construction, index sets of both a greedy dimension-adaptive (*adaptive* refinement rule) and a regular sparse grid interpolant (*regular* refinement rule) are maintained. Then, at each step of the construction algorithm, the ratio `r` = `nAdaptPoints` / `nTotalPoints` is computed. The ratio `r` represents the current degree of dimensional adaptivity. Now, if the target rate is smaller than the current rate `r`, points corresponding to an index set maintained by the adaptive rule are added to the interpolant, otherwise, points according to the regular rule are added. Thus, the generated interpolant will have a degree of dimensional adaptivity close to the target degree (it is "balanced" around the target degree).

### Benefits of the new approach

The new degree balancing approach has the following benefits:

- Transparent, easy to understand definition of the degree of dimensional adaptivity.
- Works independently of the problem dimension.
- The target degree of adaptivity can be adjusted interactively at any time (see example below).

## Examples

- Comparison of different degrees of dimensional adaptivity
- Adjusting the adaptivity degree during interpolant construction

## Comparison of different degrees of dimensional adaptivity

Let us use Branin's function to illustrate how the degree of dimensional adaptivity affects the grid construction.

```
fun = inline(['(5/pi*x-5.1/(4*pi^2)*x.^2+y-6).^2 + ' ...
              '10*(1-1/(8*pi))*cos(x)+10']);
d = 2;
range = [-5, 10; 0, 15];
```

The code snippet below generates sparse grid interpolants for target degrees of dimensional adaptivity 0, 0.5, and 1, i.e.,
about 0%, 50%, or 100% of the grid points are generated by the greedy, error-indicator based refinement rule. For each interpolant,
we set the minimum and maximum number of points to 65 in order to get an interpolant with close to that number of points.

```
options = spset('GridType', 'Chebyshev', 'DimensionAdaptive', 'on', ...
  'MinPoints', 65, 'MaxPoints', 65, 'Vectorized', 'on', 'KeepGrid', 'on');
z1 = spvals(fun, d, range, spset(options, 'DimadaptDegree', 0));
z2 = spvals(fun, d, range, spset(options, 'DimadaptDegree', 0.5));
z3 = spvals(fun, d, range, spset(options, 'DimadaptDegree', 1));
```

We can check how closely the actual degrees of dimensional adaptivity meet the target rates:

```
disp(sprintf('Degree of dimensional adaptivity: z1: %.2f, z2: %.2f, z3: %.2f', ...
  z1.dimadaptDegree, z2.dimadaptDegree, z3.dimadaptDegree));
```

```
Degree of dimensional adaptivity: z1: 0.00, z2: 0.52, z3: 0.99
```

The following plot compares the generated grids and subgrid indices.

```
z = {z1, z2, z3};
for k = 1:3
  subplot(2,3,k);
  plot(z{k}.grid{1}(:,1), z{k}.grid{1}(:,2), 'k.');
  axis([range(1,:), range(2,:)]);
  axis square;
  subplot(2,3,3+k);
  plotindices(z{k});
  title(sprintf('z%d, degree: %2.0f%%', k, 100*z{k}.dimadaptDegree))
  axis([0,8,0,6]);
end
```

## Adjusting the adaptivity degree during interpolant construction

Suppose we have created a dimension-adaptive interpolant with purely greedy refinement to achieve a target estimated error
of `1e-3`.

```
options = spset('GridType', 'Chebyshev', 'DimensionAdaptive', 'on', ...
  'RelTol', 1e-3, 'Vectorized', 'on', 'KeepGrid', 'on', 'MinPoints', 30);
z = spvals(fun, d, range, spset(options, 'DimadaptDegree', 1));
```

We find that it took 45 points to achieve this estimated accuracy:

```
z.nPoints
z.estRelError
```

```
ans =
          45
ans =
   6.3274e-07
```

We are happy with the achieved accuracy, but in order to be more comfortable with the result, we would like to add some additional
points according to the standard sparse grid refinement rule. We thus change the degree of dimensional adaptivity to 0 (=conservative,
non-adaptive refinement), and add another approx. 20 points to the interpolant.

```
z = spvals(fun, d, range, spset(options, 'DimadaptDegree', 0, ...
  'MinPoints', z.nPoints + 20, 'PrevResults', z));
```

We can now check if the estimated error is still satisfied:

```
z.estRelError
```

```
ans =
   6.3244e-07
```

The actual degree of dimensional adaptivity of the interpolant reflects our refinement by the two different rules. 44 points
(64%) out of the total points of now 69 were added according to the dimension-adaptive refinement rule.

```
z.nPoints
z.dimadaptDegree
```

```
ans =
          69
ans =
    0.6377
```

|  |  |  |  |  |
| --- | --- | --- | --- | --- |
|  | Advanced Topics |  | Multiple Output Variables |  |
