# Supplementary material for: Multiple Model-Informed Open-Loop Control of Uncertain Intracellular Signaling Dynamics
Source: PLoS Comput Biol. 2014 Apr 10;10(4):e1003546. doi: 10.1371/journal.pcbi.1003546 (PMC3983080; doi:10.1371/journal.pcbi.1003546)
Supplement: Dataset S1 — Matlab code for proposed control algorithm and prediction models. Contains all Matlab code necessary to implement the proposed adaptive weighted multiple-model predictive control algorithm, as well as code for the prediction models. (ZIP) [file pcbi.1003546.s001.zip › AW_MMPC/spinterp_v5.1.1/help/dimension-adaptive.html]

Bibliography (Sparse Grid Interpolation Toolbox)


|  |  |
| --- | --- |
| **Sparse Grid Interpolation Toolbox** |  |

# Dimensional adaptivity

With the standard sparse grid approach, all dimensions are treated equally, i.e. in each coordinate direction, the number of grid points is equal. The question arises as to whether one can further reduce the computational effort for objective functions where not all input variables carry equal weight. This is especially important in the case of higher-dimensional problems, where this property is often observed. Unfortunately, it is usually not known a priori which variables (or, in this context, dimensions) are important. Therefore, an efficient approach should be able to automatically detect which dimensions are the more or less important ones, without wasting any function evaluations.

Hegland [7] and Gerstner and Griebel [8] show that this is indeed possible. They propose an approach to generalize sparse grids such that the number of nodes in each dimension is adaptively adjusted to the problem at hand. Here, the adaptive refinement is not performed in a spatial manner, as it is commonly done in two- and three-dimensional problems (e.g. [5]), where more support nodes are placed in areas of increased nonlinearity or non-smoothness (this can become impractical in higher dimensions due to the required complex data structure and refinement criteria).

Besides being able to balancing the number of nodes in each coordinate direction, dimension-adaptive sparse grids are capable of automatically detecting separability (or partial separability) encountered in problems with additive (or nearly additive) structure.

The Sparse Grid Interpolation Toolbox includes a powerful dimension-adaptive algorithm based on the approach by Gerstner and Griebel [8], but also includes the significant performance enhancements described in [3, ch. 3]. Applying the dimension-adaptive algorithm is very easy - it can be switched "on" or "off" with a single parameter of the spare grid `options` structure that can be set with the `spset` function. Furthermore, the degree of dimensional adaptivity can be chosen as a scalar from the interval [0,1] where 1 stands for greedy (= purely adaptive) and 0 stands for conservative (= standard sparse grid) refinement.

## Example

Consider the following quadratic test function:

Implemented in Matlab by the following code:

```
type('trid.m')
```

```
function y = trid(x)
% TRID   Quadratic function with a tridiagonal Hessian.
%   Y = TRID(X)   returns the function value Y for a D-
%   dimensional input vector X.
%
% f(x)=[sum_{i=1}^d (x_i-1)^2] - [sum_{i=2}^d x_ix_{i-1}]
%
% The test function is due to Arnold Neumaier, listed
% on the global optimization Web page at 
%   http://www.mat.univie.ac.at/~neum/glopt/

d = length(x);
y = sum((x-1).^2) - sum(x(2:d).*x(1:d-1));
```

The function clearly exhibits additive structure, however, the function is not fully separable due to the second term coupling the
variables. Consider the high-dimensional case d = 100. A traditional tensor-product approach would completely fail in interpolating a high-dimensional function of this type, since
at least 2100 nodes are required if an interpolation formula with two nodes is extended to the multivariate case via conventional
tensor products. With the dimension-adaptive sparse grid algorithm, however, the structure is automatically detected, and
the function is successfully recovered using just O(d2) points. For the interpolation domain, we have used [-d2, d2] in each dimension.

Using piecewise multilinear basis functions and the Clenshaw-Curtis-Grid, f can be recovered with an estimated relative error
of below 0.1 percent (the relative error is given with respect to the estimated range of the function) with about 27000 function
evaluations, as the following code shows.

```
d = 100;
range = repmat([-d^2 d^2],d,1);
options = spset('DimensionAdaptive', 'on', ...
                'DimadaptDegree', 1, ...
                'FunctionArgType', 'vector', ...
                'RelTol', 1e-3, ...
                'MaxPoints', 40000);

z1 = spvals(@trid, d, range, options)
```

```
z1 = 
               vals: {[26993x1 double]}
           gridType: 'Clenshaw-Curtis'
                  d: 100
              range: [100x2 double]
        estRelError: 3.2552e-04
        estAbsError: 9.7656e+04
         fevalRange: [100 300000100]
         minGridVal: [1x100 double]
         maxGridVal: [1x100 double]
            nPoints: 26993
          fevalTime: 5.0122
    surplusCompTime: 0.4784
            indices: [1x1 struct]
           maxLevel: [1x100 double]
      activeIndices: [5149x1 uint32]
     activeIndices2: [5749x1 uint32]
                  E: [1x5749 double]
                  G: [5749x1 double]
                 G2: [5749x1 double]
       maxSetPoints: 6
           dimAdapt: 1
```

Since the objective function is quadratic, we can even approximate the function up to floating point accuracy with the polynomial
basis functions and the Chebyshev-Gauss-Lobatto grid:

```
options = spset(options, 'GridType', 'Chebyshev');
z2 = spvals(@trid, d, range, options)
```

```
z2 = 
               vals: {[20201x1 double]}
           gridType: 'Chebyshev'
                  d: 100
              range: [100x2 double]
        estRelError: 2.4835e-17
        estAbsError: 7.4506e-09
         fevalRange: [100 300000100]
         minGridVal: [1x100 double]
         maxGridVal: [1x100 double]
            nPoints: 20201
          fevalTime: 3.8847
    surplusCompTime: 3.5568
            indices: [1x1 struct]
           maxLevel: [1x100 double]
      activeIndices: [4951x1 uint32]
     activeIndices2: [5151x1 uint32]
                  E: [1x5151 double]
                  G: [5151x1 double]
                 G2: [5151x1 double]
       maxSetPoints: 2
           dimAdapt: 1
```

We can verify the quality of the interpolants by computing the maximum relative error for 100 randomly sampled points (the
relative error is computed with respect to the range of the function values that occurred during the sparse grid construction).
In this case, the estimated error was too optimistic in the piecewise linear case- however, the relative error for the sampled points is still below 1 percent.

```
% Compute 100 randomly sampled points
p = 100;
rand('state', 0);
x = -d^2 + 2*d^2*rand(p,d);

% Compute exact function values
y = zeros(p,1);
for k = 1:p
  y(k) = trid(x(k,:));
end

% Compute interpolated function values
xcell = num2cell(x,1);
ip1 = spinterp(z1, xcell{:});
ip2 = spinterp(z2, xcell{:});

% Compute relative errors
err_CC = max(abs(y-ip1))/(z1.fevalRange(2)-z1.fevalRange(1))
err_CGL= max(abs(y-ip2))/(z2.fevalRange(2)-z2.fevalRange(1))
```

```
err_CC =
    0.0061
err_CGL =
   1.2716e-14
```

|  |  |  |  |  |
| --- | --- | --- | --- | --- |
|  | Polynomial basis functions |  | Bibliography |  |
