# Supplementary material for: Multiple Model-Informed Open-Loop Control of Uncertain Intracellular Signaling Dynamics
Source: PLoS Comput Biol. 2014 Apr 10;10(4):e1003546. doi: 10.1371/journal.pcbi.1003546 (PMC3983080; doi:10.1371/journal.pcbi.1003546)
Supplement: Dataset S1 — Matlab code for proposed control algorithm and prediction models. Contains all Matlab code necessary to implement the proposed adaptive weighted multiple-model predictive control algorithm, as well as code for the prediction models. (ZIP) [file pcbi.1003546.s001.zip › AW_MMPC/spinterp_v5.1.1/help/examples.html]

Major examples (Sparse Grid Interpolation Toolbox)


|  |  |
| --- | --- |
| **Sparse Grid Interpolation Toolbox** |  |

# Major examples

Use this example index to jump to code examples in the documentation. The examples marked [demo] are available at the Matlab command line or from the Sparse Grid Interpolation demo page within the Matlab help browser.

## Grid visualization

- Plot available sparse grid types for level `N = 3`, `D = 2` [example]- Plot a 3D sparse grid with points colored according to level [example]- Plot the set of multi-indices `S_k` of a two-dimensional dimension-adaptive sparse grid interpolant [example]- `cmpgrids`: Plot available sparse grid types for level `N = 4`, `D = 2` [demo]

## Piecewise linear basis functions

- Interpolate a simple two-dimensional function [example]- Construct an interpolant of Branin's function [example]- Perform multiple evaluations at once: vectorized call to `spinterp` [example], [example]- `spdemo`: Interpolate a simple two-dimensional function [demo]- `spcompare`: Compare multilinear interpolation schemes for the test functions of Gerz [demo]

## Polynomial basis functions

- Construct a polynomial interpolant of Branin's function (dimension-adaptive and non-adaptive) [example]- `spcomparepoly`: Error plots for multilinear vs. polynomial basis functions [demo]

## Dimension-adaptive sparse grids

- Recovery of a quadratic function with a tridiagonal Hessian (`d = 100`, piecewise linear and polynomial basis functions) [example]- `spadaptdemo`: Dimension-adaptive interpolation of a simple two-dimensional function [demo]- `spadaptanim`: Illustrates the dimension-adaptive construction[demo]- `spadapterror`: Compares the error: adaptive vs. non-adaptive [demo]- Comparison of different degrees of dimensional adaptivity [example]- Adjusting the adaptivity degree during interpolant construction [example]

## Computing Derivatives

- Computing the derivatives of a bivariate piecewise multilinear interpolant [example]- Augmented derivatives to achieve continuity [example]- Derivatives of polynomial interpolants [example]- `spcomparederiv`: Error plots for the derivative computation schemes [demo]

## Numerical Integration

- Comparison of regular sparse grids for a 5d test problem [example]- Integrating a high-dimensional dimension-adaptive interpolant [example]

## Optimization

- Using the optimization algorithms: `spcgsearch` [example], `spcompsearch` [example], `spfminsearch` [example], `spmultistart` [example]- Optimizing a high-dimensional interpolant [example]- Using third-party optimization methods [example]

## Providing additional options

- Setting the minimum/maximum number of support nodes [example]- Re-using previous results [example]- Using the VariablePositions property [example]

## Functions with multiple outputs

- Call to `spvals`: Function header type examples 6 [example], 7 [example], 8 [example], and 9 [example]- Construct interpolant and compute interpolated values [example]- Approximate ODE output simultaneously at multiple time steps [example]- `spdemovarout`: Interpolate a function with multiple output arguments [demo]

## Performance related examples and demos

- Vectorizing the objective function [example]- Re-using previous results [example], [example]- Using `sppurge` to increase the performance when evaluating the sparse grid interpolants [example], [example]- `timespvals`: Measure the performance of the hierarchical construction of the interpolant [demo]- `timespvalsdct`: Measure the performance of the hierarchical construction of Chebyshev-polynomial-based interpolants, with and without using a fast DCT [demo]- `timespinterp`: Measure the performance of computing 1000 interpolated values [demo]- `timespderiv`: Measure the performance of computing both interpolated values and gradients [demo]

|  |
| --- |
|  |
