# Supplementary material for: Multiple Model-Informed Open-Loop Control of Uncertain Intracellular Signaling Dynamics
Source: PLoS Comput Biol. 2014 Apr 10;10(4):e1003546. doi: 10.1371/journal.pcbi.1003546 (PMC3983080; doi:10.1371/journal.pcbi.1003546)
Supplement: Dataset S1 — Matlab code for proposed control algorithm and prediction models. Contains all Matlab code necessary to implement the proposed adaptive weighted multiple-model predictive control algorithm, as well as code for the prediction models. (ZIP) [file pcbi.1003546.s001.zip › AW_MMPC/spinterp_v5.1.1/help/external.html]

External models (Sparse Grid Interpolation Toolbox)


|  |  |
| --- | --- |
| **Sparse Grid Interpolation Toolbox** |  |

# External models

Through system calls available in Matlab, one can easily execute external programs computing external models. The results from the external program can either be passed as an output stream (requires subsequent parsing of the stream to retrieve the results in usable format), or by saving the results to a file and reading the results from Matlab.

By embedding the system calls, reading/parsing of the result, etc., in Matlab functions, one can obtain wrapper functions that are treatable like regular Matlab functions, and thus, easily accessible to the `spvals` algorithm.

In the following, we present Matlab pseudo-code for a possible approach.

```
function [varargout] = external_model(external_config, x1, ..., xd)

try 
  store permutation (x1,...xd) to external_config.inputfile

  % Start external program, pass input file name to program, pass
  % output file name to program.
  system([external_config.program ' -i ' external_config.inputfile ...
	       ' -o ' external_config.outputfile]);
	       
  read result from external_config.outputfile into varargout
catch
  Do some error handling
end
```

In the presented case, the call to `spvals` would look like this:

```
external_config.program = 'myprog.exe';
external_config.inputfile = 'in.txt';
external_config.outputfile = 'out.txt';
options = spset('VariablePositions', [1 + 1:d], 'NumberOfOutputs', nout);
z = spvals(@external_model, d, range, options, external_config);
```

|  |  |  |  |  |
| --- | --- | --- | --- | --- |
|  | Approximating ODEs |  | Sparse Grid Interpolation product page |  |
