# Supplementary material for: Multiple Model-Informed Open-Loop Control of Uncertain Intracellular Signaling Dynamics
Source: PLoS Comput Biol. 2014 Apr 10;10(4):e1003546. doi: 10.1371/journal.pcbi.1003546 (PMC3983080; doi:10.1371/journal.pcbi.1003546)
Supplement: Dataset S1 — Matlab code for proposed control algorithm and prediction models. Contains all Matlab code necessary to implement the proposed adaptive weighted multiple-model predictive control algorithm, as well as code for the prediction models. (ZIP) [file pcbi.1003546.s001.zip › AW_MMPC/spinterp_v5.1.1/help/firstexample.html]

A first example (Sparse Grid Interpolation Toolbox)


|  |  |
| --- | --- |
| **Sparse Grid Interpolation Toolbox** |  |

# A first example

Let us interpolate a simple two-variate function

with the default settings of the sparse grid interpolation package. Here, we interpolate the function for the domain [0,pi]x[0,pi].

## Constructing the interpolant

First, we compute the hierarchical surpluses (i.e. the coefficients) of the interpolant.

```
f = @(x,y) sin(x) + cos(y);
z = spvals(f,2,[0 pi; 0 pi])
```

```
z = 
               vals: {[65x1 double]}
           gridType: 'Clenshaw-Curtis'
                  d: 2
              range: [2x2 double]
           maxLevel: 4
        estRelError: 0.0063
        estAbsError: 0.0188
         fevalRange: [-1 2]
         minGridVal: [0 1]
         maxGridVal: [0.5000 0]
            nPoints: 65
          fevalTime: 0.0502
    surplusCompTime: 0.0024
            indices: [1x1 struct]
```

The function spvals returns these hierarchical surpluses, and also includes some additional information collected during the construction process
of the interpolant. For instance, We obtain information on the estimated relative and absolute error. The number of sparse
grid support nodes is provided, as well as the computing time for evaluating the function and computing the hierarchical surpluses.
The surpluses themselves are stored under the field vals.

## Computing interpolated values

To compute interpolated values, we can now use the spinterp function. To increase efficiency, multiple interpolated values can be computed at once. Below, we compute the interpolated
values for five randomly chosen points and compare them to the exact function value by computing the maximum absolute error.

```
x1 = pi*rand(1,5); x2 = pi*rand(1,5);
y = spinterp(z,x1,x2)
error = max(abs(y - f(x1,x2)))
```

```
y =
    1.7173    0.7210    0.2675    0.7701    0.5510
error =
    0.0076
```

## Visualizing the sparse grid

Let us now visualize the sparse grid. From the information returned by spvals, we see that the used sparse grid is of the type Clenshaw-Curtis, and the maximum level was 4. In two and three dimensions,
we can easily plot the sparse grid with the plotgrid function. It takes the level and the dimension as input arguments. Optional is an options structure containing the sparse
grid type, created with spset. The default grid type is the Clenshaw-Curtis grid, we thus do not have to specify the grid type here.

```
plotgrid(4,2)
```

## Visualizing the interpolant

To visualize the original function and compare it to the interpolant, we can plot both functions, for instance, by using ezmesh.

```
subplot(1,2,1);
ezmesh(f,[0 pi]);
title('f(x,y) = sin(x) + cos(y)');
subplot(1,2,2);
ezmesh(@(x,y) spinterp(z,x,y),[0 pi]);
title('Sparse grid interpolant, n = 4');
```

|  |  |  |  |  |
| --- | --- | --- | --- | --- |
|  | Initialization of the toolbox |  | Linear basis functions |  |
