# Supplementary material for: Multiple Model-Informed Open-Loop Control of Uncertain Intracellular Signaling Dynamics
Source: PLoS Comput Biol. 2014 Apr 10;10(4):e1003546. doi: 10.1371/journal.pcbi.1003546 (PMC3983080; doi:10.1371/journal.pcbi.1003546)
Supplement: Dataset S1 — Matlab code for proposed control algorithm and prediction models. Contains all Matlab code necessary to implement the proposed adaptive weighted multiple-model predictive control algorithm, as well as code for the prediction models. (ZIP) [file pcbi.1003546.s001.zip › AW_MMPC/spinterp_v5.1.1/help/functions_list.html]

Functions -- Alphabetical List :: (Sparse Grid Interpolation Toolbox)


|  |  |
| --- | --- |
| **Sparse Grid Interpolation Toolbox** |  |

# Functions -- Alphabetical List

cmpgrids  
plotgrid  
plotindices  
spcgsearch  
spcompsearch  
spdim  
spfminsearch  
spget  
spgrid  
spinit  
spinterp  
spmultistart  
spoptimget  
spoptimset  
sppurge  
spquad  
spset  
spsurfun  
spvals

|  |
| --- |
|  |
