# Supplementary material for: Multiple Model-Informed Open-Loop Control of Uncertain Intracellular Signaling Dynamics
Source: PLoS Comput Biol. 2014 Apr 10;10(4):e1003546. doi: 10.1371/journal.pcbi.1003546 (PMC3983080; doi:10.1371/journal.pcbi.1003546)
Supplement: Dataset S1 — Matlab code for proposed control algorithm and prediction models. Contains all Matlab code necessary to implement the proposed adaptive weighted multiple-model predictive control algorithm, as well as code for the prediction models. (ZIP) [file pcbi.1003546.s001.zip › AW_MMPC/spinterp_v5.1.1/help/getting_started.html]

Sparse Grid Interpolation basics (Sparse Grid Interpolation Toolbox)


|  |  |
| --- | --- |
| **Sparse Grid Interpolation Toolbox** |  |

# Sparse Grid Interpolation basics

The following chapter describes how to begin using the Sparse Grid Interpolation Toolbox for your approximation and function recovery problems.

|  |  |
| --- | --- |
| What is the Sparse Grid Interpolation Toolbox? | Major features and key areas of the toolbox |
| Initialization of the toolbox | Required prior to using the toolbox |
| A first example | Interpolating a simple two-dimensional function |
| Linear basis functions | Sparse grids with piecewise linear basis functions || Polynomial basis functions | Sparse grids with polynomial basis functions || Dimensional adaptivity | Dimension-adaptive sparse grids |
| Bibliography | Selected references on sparse grids and sparse grid interpolation |

|  |  |  |  |  |
| --- | --- | --- | --- | --- |
|  | Sparse Grid Interpolation product page |  | What is the Sparse Grid Interpolation Toolbox? |  |
