# Supplementary material for: Multiple Model-Informed Open-Loop Control of Uncertain Intracellular Signaling Dynamics
Source: PLoS Comput Biol. 2014 Apr 10;10(4):e1003546. doi: 10.1371/journal.pcbi.1003546 (PMC3983080; doi:10.1371/journal.pcbi.1003546)
Supplement: Dataset S1 — Matlab code for proposed control algorithm and prediction models. Contains all Matlab code necessary to implement the proposed adaptive weighted multiple-model predictive control algorithm, as well as code for the prediction models. (ZIP) [file pcbi.1003546.s001.zip › AW_MMPC/spinterp_v5.1.1/help/initialization.html]

Initialization of the toolbox (Sparse Grid Interpolation Toolbox)


|  |  |
| --- | --- |
| **Sparse Grid Interpolation Toolbox** |  |

# Initialization of the toolbox

To initialize the toolbox, it must be added to the Matlab search path. You can do this by calling the function `spinit`, i.e. go to the directory containing the sparse grid interpolation toolbox and enter `spinit` at the Matlab prompt.

If you would like to use the Sparse Grid Interpolation Toolbox from within another Matlab application, you may call the `spinit` function as well. It will automatically add the correct paths to the Matlab path.

|  |  |  |  |  |
| --- | --- | --- | --- | --- |
|  | What is the Sparse Grid Interpolation Toolbox? |  | A first example |  |
