# Supplementary material for: Multiple Model-Informed Open-Loop Control of Uncertain Intracellular Signaling Dynamics
Source: PLoS Comput Biol. 2014 Apr 10;10(4):e1003546. doi: 10.1371/journal.pcbi.1003546 (PMC3983080; doi:10.1371/journal.pcbi.1003546)
Supplement: Dataset S1 — Matlab code for proposed control algorithm and prediction models. Contains all Matlab code necessary to implement the proposed adaptive weighted multiple-model predictive control algorithm, as well as code for the prediction models. (ZIP) [file pcbi.1003546.s001.zip › AW_MMPC/spinterp_v5.1.1/help/integration.html]

Numerical Integration (Quadrature) (Sparse Grid Interpolation Toolbox)


|  |  |
| --- | --- |
| **Sparse Grid Interpolation Toolbox** |  |

# Numerical Integration (Quadrature)

Once you have computed a sparse grid interpolant of an objective function, you can compute the integral value of it for the given range. You can do this for any grid type, and for both regular and dimension-adaptive sparse grid interpolants by simply calling the spquad function. A couple of examples are provided below.

## Contents

- Integration of regular sparse grid interpolants
- Integration of dimension-adaptive sparse grid interpolants

## Integration of regular sparse grid interpolants

Consider the task of integrating the test function

for the domain [0,1]^d, and d = 5. The exact value of the integral is 1. We reproduce the results of Table 1 for the columns labelled "Trapez", "Clenshaw",
and "Gauss-Patterson" from the paper "Numerical integration using sparse grids" by T. Gerstner and M. Griebel, Numerical
Algorithms 18(3-4), 1998, pp. 209-232.

Note that the grid type Clenshaw-Curtis and Chebyshev of the Sparse Grid Interpolation Toolbox correspond to the sparse grids
based on the Trapez rule and the Clenshaw Curtis rule in the paper, respectively.

Define the objective function, dimension, and maximum depth:

```
d = 5;
maxDepth = 6;
f = @(x) (1+1/d)^d * prod(x)^(1/d);
```

Compute integral for increasing sparse grid depth, and generate the results table:

```
warning('off', 'MATLAB:spinterp:insufficientDepth');
z = cell(3,1);
quadr = zeros(3,1);
disp(' Clenshaw-Curtis |    Chebyshev     | Gauss-Patterson');
disp(' points    error | points     error | points     error');
for k = 0:maxDepth
  options = spset('MinDepth', k, 'MaxDepth', k, ...
                  'FunctionArgType', 'vector');

        % Compute integral values with Clenshaw-Curtis grid
        options = spset(options, 'GridType', 'Clenshaw-Curtis', ...
                  'PrevResults', z{1});
  z{1} = spvals(f,d,[],options);
  quadr(1) = spquad(z{1});

        % Compute integral values with Chebyshev grid
        options = spset(options, 'GridType', 'Chebyshev', ...
                  'PrevResults', z{2});
  z{2} = spvals(f,d,[],options);
  quadr(2) = spquad(z{2});

        % Compute integral values with Gauss-Patterson grid
        options = spset(options, 'GridType', 'Gauss-Patterson', ...
                  'PrevResults', z{3});
  z{3} = spvals(f,d,[],options);
  quadr(3) = spquad(z{3});

  % Results output
  disp(sprintf('%5d  %8.3e | %5d  %8.3e | %5d  %8.3e', ...
          z{1}.nPoints, abs(1-quadr(1)), ...
                z{2}.nPoints, abs(1-quadr(2)), ...
                z{3}.nPoints, abs(1-quadr(3))));
end
```

```
 Clenshaw-Curtis |    Chebyshev     | Gauss-Patterson
 points    error | points     error | points     error
    1  2.442e-01 |     1  2.442e-01 |     1  2.442e-01
   11  1.080e+00 |    11  6.385e-01 |    11  8.936e-03
   61  7.578e-02 |    61  1.441e-01 |    71  8.073e-04
  241  2.864e-01 |   241  1.237e-01 |   351  2.070e-04
  801  1.079e-01 |   801  6.650e-03 |  1471  2.256e-05
 2433  8.001e-02 |  2433  1.060e-02 |  5503  1.420e-06
 6993  5.030e-02 |  6993  1.743e-03 | 18943  3.437e-09
```

## Integration of dimension-adaptive sparse grid interpolants

To illustrate a higher-dimensional, dimension-adpative case, we consider the absorption problem from W. Morokoff, R. Caflisch,
"Quasi-monte carlo integration", J. Comp. Phys. 122, pp. 218-230, 1995, given by the integral equation

The exact solution of this equation is given by

Two alternate representations are given in the paper, the first being an infinite integral with an integrand with a jump,
and the second one with a smooth integrand.

The sparse grid method does not work well for the first representation, since it is a non-smooth function where the discontinuities
are not parallel to the coordinate directions (see S. Dirnsdorfer, "Numerical Quadrature on Sparse Grids", Diploma Thesis,
TU Munich, 2000). However, in case of the second representation, very accurate results can be computed using the dimension-adaptive
approach, as shown below.

We define the integrand of the absorption problem as follows. The optional parameter named SMOOTH indicates which of the two representation should be used.

```
type('absorb.m');
```

```
function y = absorb(varargin)
% ABSORB(R1,...,RD,GAMMA,X,SMOOTH)   Finite sum integrand of 
%   the integrral representation of the absorption problem 
%   paper W. Morokoff, R. Caflisch, "Quasi-monte carlo 
%   integration", J. Comp. Phys. 122, pp. 218-230, 1995.

d = length(varargin) - 3;
gamma = varargin{end-2};
x = varargin{end-1};
smooth = varargin{end};

% Compute F as in paper 
if ~smooth
  % First representation with jump

  phi = @(z) (1 .* (z >= 0));  % Heaviside function
  d = d - 1;
  sumR = cell(d+1,1);
  sumR{1} = varargin{1};
  for k = 2:d+1
    sumR{k} = sumR{k-1} + varargin{k};
  end
  F = @(n) (gamma^n * phi(1-x-sumR{n}) ...
    .* phi(sumR{n+1}-(1-x)));
else
  % Second, smooth representation
  
  prodR1 = cell(d,1);
  prodR1{1} = ones(size(varargin{1}));
  for k = 2:d
    prodR1{k} = prodR1{k-1};
    for l = 1:k-1
      prodR1{k} = prodR1{k} .* varargin{l};
    end
  end
  prodR2 = cell(d,1);
  prodR2{1} = varargin{1};
  for k = 2:d
    prodR2{k} = prodR2{k-1} .* varargin{k};
  end
  F = @(n) (gamma^n * (1 - x)^n * ...
    prodR1{n} .* (1 - (1 - x) * prodR2{n}));
end

% Compute integrand value(s) (finite sum)
y = zeros(size(varargin{1}));
for n = 1:d
  y = y + F(n);
end
```

The following loop computes increasingly accurate approximations to the solution of the absorption problem with d = 20, gamma = 0.5, and x = 0 by computing a dimension-adaptive polynomial interpolant of the smooth integrand which is then integrated using the spquad function. For comparison, we also compute the integral using crude Monte Carlo (MC) with the same number of points.

```
gamma = 0.5; x = 0; d = 20;

% Show exact solution
I_exact = 1/gamma - (1-gamma)/gamma*exp(gamma*(1-x))

options = spset('DimensionAdaptive','on', 'DimadaptDegree', 1, ...
                'GridType', 'Chebyshev', 'Vectorized', 'on');
Nmax = 50000;
N = 2*d;
z = [];

warning('off', 'MATLAB:spinterp:maxPointsReached');
while N <= Nmax
  % Compute integral via sparse grid
  spoptions = spset(options, 'MinPoints', N, ...
    'MaxPoints', N, 'PrevResults', z);
  z = spvals(@absorb, d, [], spoptions, gamma, x, true);
  e1 = abs(I_exact - spquad(z));

  % Compute integral via MC (error average for 10 runs)
  e2 = 0;
  for k = 1:10
    p = rand(z.nPoints,d);
    p = num2cell(p,1);
    I = sum(absorb(p{:}, gamma, x, true)) / double(z.nPoints);
    e2 = e2 + abs(I_exact - I);
  end
  e2 = e2 / 10;

  disp([' points: ' sprintf('%5d', z.nPoints) ...
      ' | error (CGL): ' sprintf('%9.3e',e1) ...
      ' | error (MC): ' sprintf('%9.3e',e2)]);
  N = round(z.nPoints .* 2);
end
warning('on', 'MATLAB:spinterp:maxPointsReached');
```

```
I_exact =
    0.3513

 points:    41 | error (CGL): 4.622e-04 | error (MC): 1.298e-02
 points:    87 | error (CGL): 5.606e-06 | error (MC): 6.008e-03
 points:   177 | error (CGL): 6.010e-07 | error (MC): 7.791e-03
 points:   367 | error (CGL): 1.566e-07 | error (MC): 3.442e-03
 points:   739 | error (CGL): 3.893e-08 | error (MC): 4.761e-03
 points:  1531 | error (CGL): 2.461e-08 | error (MC): 1.895e-03
 points:  3085 | error (CGL): 1.061e-09 | error (MC): 1.036e-03
 points:  6181 | error (CGL): 2.750e-09 | error (MC): 6.147e-04
 points: 12393 | error (CGL): 1.335e-09 | error (MC): 6.609e-04
 points: 24795 | error (CGL): 3.006e-10 | error (MC): 5.420e-04
 points: 49739 | error (CGL): 1.791e-10 | error (MC): 3.305e-04
```

|  |  |  |  |  |
| --- | --- | --- | --- | --- |
|  | Derivatives |  | Optimization |  |
