# Supplementary material for: Multiple Model-Informed Open-Loop Control of Uncertain Intracellular Signaling Dynamics
Source: PLoS Comput Biol. 2014 Apr 10;10(4):e1003546. doi: 10.1371/journal.pcbi.1003546 (PMC3983080; doi:10.1371/journal.pcbi.1003546)
Supplement: Dataset S1 — Matlab code for proposed control algorithm and prediction models. Contains all Matlab code necessary to implement the proposed adaptive weighted multiple-model predictive control algorithm, as well as code for the prediction models. (ZIP) [file pcbi.1003546.s001.zip › AW_MMPC/spinterp_v5.1.1/help/interfacing.html]

Interfacing concepts (Sparse Grid Interpolation Toolbox)


|  |  |
| --- | --- |
| **Sparse Grid Interpolation Toolbox** |  |

# Interfacing concepts

Applying the `spvals` method to construct interpolants sometimes requires a small interface function. In this section, we show the most important categories of Matlab function headers and (if necessary) how to design an appropriate interface function for them. The following tables shows the basic function header types discussed here. Combinations of those are of course also possible and can be derived from the treated cases. In the tables, the objective interpolation variables (all must be real-valued scalars) are denoted by `x1,...,xn`. Examples of the presented cases are provided below.

## Interface function NOT required

| **header** | **variable types** |
| --- | --- |
| `out = fun(x1, x2, ..., xn)` | `x1, ..., xn` are real scalars |
| `out = fun(x1, x2, ..., xn, p1, p2, ..., pm)` | `x1, ..., xn` are real scalars, `p1, ..., pm` are parameters of arbitrary type (double array, cell array, structure, etc.) |
| `out = fun(x1, ..., xi1, p1, ..., pj1, xi1+1, ..., xi2, pi1+1, ..., pj2, ...)` | `x1, ..., xn` are real scalars, `p1, ..., pm` are parameters of arbitrary type (double array, cell array, structure, etc.) |
| `out = fun(v)` | `v` is a row or column vector with the entries `x1, ..., xn` |
| `out = fun(v, p1, p2, ..., pm)` | `v` is a row or column vector with the real scalar entries `x1, ..., xn`, and `p1, ..., pm` are parameters of arbitrary type (double array, cell array, structure, etc.) |
| `[out1, out2, ..., outn] = fun(...)` | `out1, ..., outn` are real scalar output parameters, input parameters the same as one of the above |
| `varargout = fun(...)` | `varargout` is a cell array of real scalar output parameters `out1, ..., outn`, input parameters the same as one of the above |

## Interface function REQUIRED (only some exemplary cases)

| **header** | **variable types** |
| --- | --- |
| `out = fun(A, p1, p2, ..., pm)` | `A` is a matrix where some of its entries are the objective interpolation parameters `x1, ..., xn`, and `p1, ..., pm` are parameters of arbitrary type as above |
| `vout = fun(x1, x2, ..., xn)` | `vout` is a row or column vector with real scalar outputs |

## Examples

### Type 1: `out = fun(x1, x2, ..., xn)`

Objective function:

```
type('fun1.m')
```

```
function y = fun1(x1, x2)
y = x1 .* x2;    % Use '.' before any '^', '*' or '/' to enable
y = y.^2;        % vectorized evaluation of expressions
```

Example for call to spvals:

```
options = spset('Vectorized', 'on');
range   = [0,2; 0,2];
z = spvals(@fun1, 2, range, options);
```

  

### Type 2: `out = fun(x1, x2, ..., xn, p1, p2, ..., pm)`

Objective function:

```
type('fun2.m');
```

```
function y = fun2(x1, x2, c, params)
y = c .* (params.p1 .* x1 + length(params.p2) .* x2);
```

Example for call to spvals:

```
options = spset('Vectorized', 'on');
range = [];   % use default range [0,1]^d
c = 2;
params = struct('p1', 3, 'p2', 'hello');
z = spvals(@fun2, 2, range, options, c, params);
```

  

### Type 3: `out = fun(x1, ..., xi1, p1, ..., pj1, xi1+1, ..., xi2, pi1+1, ..., pj2, ...)`

Objective function:

```
type('fun3.m');
```

```
function y = fun3(p1, x1, p2, x2)
y = p1 .* x1 + p2 .* x2;
```

Example for call to spvals:

```
options = spset('VariablePositions', [2 4], 'Vectorized', 'on');
range = [0,1; -1,2];
p1 = 2; p2 = 3;
z = spvals(@fun3, 2, range, options, p1, p2);
```

  

### Type 4: `out = fun(v)`

Objective function:

```
type('fun4.m');
```

```
function y = fun4(x)
y = prod(x);
```

Example for call to spvals:

```
options = spset('FunctionArgType', 'vector');
range = [0 1; 1 2; 2 3; 3 4; 4 5];
z = spvals(@fun4, 5, range, options);
```

  

### Type 5: `out = fun(v, p1, p2, ..., pm)`

Objective function:

```
type('fun5.m');
```

```
function y = fun5(x,p);
y = x(:)'*p;   % Compute dot product
```

Example for call to spvals:

```
options = spset('FunctionArgType', 'vector');
range = []; % use default range [0,1]^d
p = rand(3,1);
z = spvals(@fun5, 3, range, options, p);
```

  

### Type 6: `[out1, out2, ..., outn] = fun(...)`

Objective function:

```
type('fun6.m');
```

```
function [y1, y2] = fun6(x1, x2);
y1 = 2*x1 + 3*x2;
y2 = 4*x1 - 1*x2;
```

Example for call to spvals:

```
options = spset('NumberOfOutputs', 2);
range = []; % use default range [0,1]^d
z = spvals(@fun6, 2, range, options);
```

To compute interpolated values of functions with multiple output parameters, see the help page multiple output arguments.

  

### Type 7: `varargout = fun(...)`

Objective function:

```
type('fun7.m');
```

```
function varargout = fun7(x1,x2,nout);
for k = 1:nout
  varargout{k} = x1.^k + k.*x2;
end
```

Example for call to spvals:

```
nout = 4;
options = spset('NumberOfOutputs', nout, 'Vectorized', 'on');
range = []; % use default range [0,1]^d
z = spvals(@fun7, 2, range, options, nout);
```

To compute interpolated values of functions with multiple output parameters, see the help page multiple output arguments.

  

### Type 8: `out = fun(A, p1, p2, ..., pm)`

Objective function:

```
type('fun8.m');
```

```
function y = fun8(A, f);
y = A\f;
```

Assume that the diagonal entries of A a\_11, a\_22, ..., a\_nn vary in some given range. An interpolant of fun8 is sought for
these varying diagonal entries of A:

```
d = 3;
A = magic(d); f = ones(d,1);
range = [diag(A)-0.5 diag(A)+0.5];
nout = d;
options = spset('NumberOfOutputs', nout, 'FunctionArgType', 'vector');
z = spvals(@interface_fun8, d, range, options, A, f);
```

The interface function interface\_fun8 looks like this:

```
type('interface_fun8.m');
```

```
function varargout = interface_fun8(a, A, f);
% Interface function to fun8

% Write the modifiable entries into A
for k = 1:length(a);
  A(k,k) = a(k);
end

% Call objective function fun8
y = fun8(A,f);

% Put the results in cell array (outputs must be cell row vector 
% of scalars to be treated by spvals)
varargout = num2cell(y)';
```

Note that the original output, a column vector from the solution of the linear equation system is transformed into a cell array
with a single row to match one of the admissible output variants. The original input is also modified to contain the interpolation
parameters as a vector, which is permitted by `spvals`. The original Matrix as well as the right-hand side f are passed as additional
parameters.

To compute interpolated values of functions with multiple output parameters, see the help page multiple output arguments.

  

### Type 9: `vout = fun(x1, x2, ..., xn`)

Objective function:

```
type('fun9.m');
```

```
function y = fun9(x1, x2)
y = [x2 .* cos(x1); ...
     x2 .* sin(x1); ...
     x2];
```

Assume that the output of fun9 is not a list of real scalars or a varargout cell array. In this case, a conversion of the output is required. The interface
function uses Matlab's num2cell function to achieve this.

```
type('interface_fun9.m');
```

```
function varargout = interface_fun9(x1, x2);
y = fun9(x1, x2);
varargout = num2cell(y)';
```

Example for call to spvals:

```
nout = 3;
options = spset('NumberOfOutputs', nout);
z = spvals(@interface_fun9, 2, [], options);
```

To compute interpolated values of functions with multiple output parameters, see the help page multiple output arguments.

|  |  |  |  |  |
| --- | --- | --- | --- | --- |
|  | Optimizing performance |  | Approximating ODEs |  |
