# Supplementary material for: Multiple Model-Informed Open-Loop Control of Uncertain Intracellular Signaling Dynamics
Source: PLoS Comput Biol. 2014 Apr 10;10(4):e1003546. doi: 10.1371/journal.pcbi.1003546 (PMC3983080; doi:10.1371/journal.pcbi.1003546)
Supplement: Dataset S1 — Matlab code for proposed control algorithm and prediction models. Contains all Matlab code necessary to implement the proposed adaptive weighted multiple-model predictive control algorithm, as well as code for the prediction models. (ZIP) [file pcbi.1003546.s001.zip › AW_MMPC/spinterp_v5.1.1/help/linear.html]

Linear basis functions (Sparse Grid Interpolation Toolbox)


|  |  |
| --- | --- |
| **Sparse Grid Interpolation Toolbox** |  |

# Piecewise linear basis functions

Piecewise linear basis functions provide a good compromise between accuracy and computational cost due to their bounded support. The Sparse Grid Interpolation package includes three different grid types that work with piecewise multilinear basis functions:

- The Clenshaw-Curtis grid type "`ClenshawCurtis`" (CC)- the "classical" maximum-norm-based grid type "`Maximum`" (M), as described e.g. by Bungartz/Griebel in [1],- The maximum-norm-based grid without points on the boundary "`NoBoundary`" (NB), with basis functions that extrapolate towards the boundary (it is not assumed that the objective function must be zero at the boundary).

For a detailed description of the piecewise multilinear basis functions implemented here, please see [2] or [3, ch. 3], and the references stated therein.

## Accuracy of piecewise multilinear interpolation

We now take a brief look at the approximation quality. An a priori error estimate can be obtained for a `d`-variate function f if continuous mixed derivatives

with

exist. According to [4] or [5], the order of the interpolation error in the maximum norm is then given by

where Aq,d(f) denotes the sparse grid interpolant of f, and N denotes the number of grid points of the sparse grids of type CC or M (the NB grid type has not yet been analyzed, but shows the same order of convergence in numerical tests). Note that the number of grid points N of Aq,d(f) can be computed by `spdim(q-d,d)`. Piecewise multilinear approximation on a full grid with N\* grid points is much less efficient, i.e. *O*(N\*-2/d).

## Number of grid points

The following table shows the number of grid points of the non-adaptive sparse grid interpolant depending on the interpolation depth `n`.

| n | **d=2** | | | **d=4** | | | **d=8** | | |
| --- | --- | --- | --- | --- | --- | --- | --- | --- | --- |
|  | M | NB | CC | M | NB | CC | M | NB | CC |
| 0 | 9 | 1 | 1 | 81 | 1 | 1 | 6561 | 1 | 1 |
| 1 | 21 | 5 | 5 | 297 | 9 | 9 | 41553 | 17 | 17 |
| 2 | 49 | 17 | 13 | 945 | 49 | 41 | 1.9e5 | 161 | 145 |
| 3 | 113 | 49 | 29 | 2769 | 209 | 137 | 7.7e5 | 1121 | 849 |
| 4 | 257 | 129 | 65 | 7681 | 769 | 401 | 2.8e6 | 6401 | 3937 |
| 5 | 577 | 321 | 145 | 20481 | 2561 | 1105 | 9.3e6 | 31745 | 15713 |
| 6 | 1281 | 769 | 321 | 52993 | 7937 | 2929 | 3.0e7 | 141569 | 56737 |
| 7 | 2817 | 1793 | 705 | 1.3e5 | 23297 | 7537 | 9.1e7 | 5.8e5 | 1.9e5 |

The following graph illustrates the sparse grids of level 0 and level 2 of the three respective grids in two dimensions.

## Which piecewise linear interpolation scheme works best?

Although the performance of the three grid types is rather similar for lower-dimensional problems, there are two important points to be mentioned:

- The `ClenshawCurtis` grid and the `NoBoundary` grid have just a single node at the lowest interpolation level `n = 0` (this means that an interpolant of level 0 of these grid types is just a constant function). The `Maximum` grid has `3^d` nodes at the lowest level. Therefore, the `Maximum` is not well-suited for higher-dimensional problems. For instance, for `d = 10`, already 59049 support nodes would be required to obtain an initial interpolant.- Since the CC-grid is the most versatile grid working well in both lower and higher dimensions, at this point, the dimension-adaptive algorithms are implemented for this grid type only.

Therefore, for most practical applications, we recommend using the Clenshaw-Curtis grid. Occasionally, the other grid types may perform better by a small factor, as numerical experiments show (try running the demo `spcompare` from the command line or from the Sparse Grid Interpolation demo page).

|  |  |  |  |  |
| --- | --- | --- | --- | --- |
|  | A first example |  | Polynomial basis functions |  |
