# Supplementary material for: Multiple Model-Informed Open-Loop Control of Uncertain Intracellular Signaling Dynamics
Source: PLoS Comput Biol. 2014 Apr 10;10(4):e1003546. doi: 10.1371/journal.pcbi.1003546 (PMC3983080; doi:10.1371/journal.pcbi.1003546)
Supplement: Dataset S1 — Matlab code for proposed control algorithm and prediction models. Contains all Matlab code necessary to implement the proposed adaptive weighted multiple-model predictive control algorithm, as well as code for the prediction models. (ZIP) [file pcbi.1003546.s001.zip › AW_MMPC/spinterp_v5.1.1/help/multiple_outputs.html]

Multiple output variables (Sparse Grid Interpolation Toolbox)


|  |  |
| --- | --- |
| **Sparse Grid Interpolation Toolbox** |  |

# Multiple output variables

Real-world problems usually have more than a single output argument. Sparse grids are very well-suited to deal with such kind of problems, since the regular structure allows to construct good approximations for multiple output variables at once.

The sparse grid interpolation package is designed to make dealing with multiple output arguments easy, as the following example demonstrates.

## Example

Consider the following simple test function with multiple output arguments:

```
type multiout.m
```

```
function [out1, out2, out3, out4] = multiout(x,y)
% MULTIOUT   Test function with four output arguments
out1 = (x+y).^2;
out2 = 1./exp(1+(x-0.5).^2+(y-0.3).^2);
out3 = sin(pi*(2-x))+cos(pi*(1-y));
out4 = sinh(4.*(x-0.5));
```

spvals will automatically compute interpolants with respect to all four output variables if the number of output variables is specified
in the sparse grid OPTIONS structure:

```
nout = 4;
options = spset('NumberOfOutputs', nout, 'Vectorized', 'on');
z = spvals(@multiout, 2, [], options)
```

```
z = 
               vals: {4x1 cell}
           gridType: 'Clenshaw-Curtis'
                  d: 2
              range: []
           maxLevel: 5
        estRelError: 0.0034
        estAbsError: 0.0249
         fevalRange: [4x2 double]
         minGridVal: [4x2 double]
         maxGridVal: [4x2 double]
            nPoints: 145
          fevalTime: 0.0392
    surplusCompTime: 0.0129
            indices: [1x1 struct]
```

Note that the output parameters of the objective function must **all be scalar**. The number of outputs nout specified in the options structure may be smaller than the actual number of outputs. In this case, interpolants are constructed only with respect to the first nout arguments.

To compute interpolated values, the desired output argument must now be specified. This is done by adding an additional field **selectOutput** to the structure z prior to the call to the spinterp function. The following code plots the four computed interpolants:

```
for k = 1:nout
  z.selectOutput = k;
  subplot(2,2,k);
  ezmesh(@(x,y) spinterp(z,x,y), [0 1]);
  axis square;
  title(['out' num2str(k)]);
end
```

An additional example of using multiple output arguments with spvals is given by the demo spdemovarout available at the command line or from the demos page.

|  |  |  |  |  |
| --- | --- | --- | --- | --- |
|  | Degree of Dimensional Adaptivity |  | Derivatives |  |
