# Supplementary material for: Multiple Model-Informed Open-Loop Control of Uncertain Intracellular Signaling Dynamics
Source: PLoS Comput Biol. 2014 Apr 10;10(4):e1003546. doi: 10.1371/journal.pcbi.1003546 (PMC3983080; doi:10.1371/journal.pcbi.1003546)
Supplement: Dataset S1 — Matlab code for proposed control algorithm and prediction models. Contains all Matlab code necessary to implement the proposed adaptive weighted multiple-model predictive control algorithm, as well as code for the prediction models. (ZIP) [file pcbi.1003546.s001.zip › AW_MMPC/spinterp_v5.1.1/help/ode.html]

Approximating ODEs (Sparse Grid Interpolation Toolbox)


|  |  |
| --- | --- |
| **Sparse Grid Interpolation Toolbox** |  |

# Approximating ODEs

As an example for a more complex function with multiple input- and output arguments, we show how to handle an ordinary differential equation. The model considered is a second order differential equation

Q''(t) + a Q'(t) + b = 50 cos(t)

from [1, pp. 145-162] simulating an electrical circuit.

## Contents

- The ODE model in Matlab
- Interpolation problem statement and possible applications
- The interface function
- Interpolant construction
- Computing interpolated values

## The ODE model in Matlab

Rewriting this second-order equation as a system of first order equations, we can define the ODE file in MATLAB format as follows:

```
type('circuit.m')
```

```
function [out1, out2, out3] = circuit(t, u, flag, a, b);
% CIRCUIT   definition of the electrical circuit ODE.
	
switch flag
 case ''
	out1 = [u(2); 50*cos(t) - a*u(2) - b*u(1)];
 case 'init'
	out1 = [0; 5];       % tspan
	out2 = [5; 1];       % initial conditions
	out3 = odeset('RelTol', 1e-6);
end
```

We can solve this ODE for a = 2, b = 4, and the default initial conditions and time span as defined in the ODE file using
the MATLAB solver ODE45.

```
[t,Q] = ode45('circuit', [], [], [], 2, 4);
plot(t,Q)
xlabel('t');
grid on;
legend('Q(t)', 'Q''(t)');
```

## Interpolation problem statement and possible applications

We now consider the initial conditions and the parameters a,b to vary in some range, that is we assume intervals for Q(0),
Q'(0), a, and b, and compute an error-controlled sparse grid interpolant for the ODE model at each time step. The interpolant
can then be used to do several useful analyses, for instance, perform a Monte Carlo simulation with random variables, optimize
the model for the given range of parameters and initial conditions, e.g. minimize or maximize the amplitude, or compute an
envelope of the result using fuzzy calculus or interval analysis. In many cases, this can be done considerably faster than
by using the original ODE directly, since the construction and evaluation of the interpolant is very fast.

## The interface function

We proceed as follows. First of all, we write an interface function of the ODE model to enable its evaluation by the spvals function.

```
type('interface_circuit.m')
```

```
function varargout = interface_circuit(Q0, Q0prime, a, b, tspan, nsteps)
% Definition of the complete model as a function of the uncertain
% input parameters. 

% The time steps must be at fixed steps such that the number of
% outputs and time steps stay the same for each parameter
% variation. 
t = linspace(tspan(1), tspan(2), nsteps);

% Call the ODE solver
[t, Q] = ode45('circuit', t, [Q0 Q0prime], [], a, b);

% Convert result vector to parameter list. This conversion is
% necessary, since the output arguments of the objective function
% to SPVALS must all be scalar. In this case, we assume that only
% the first column (i.e. Q, not Q') is of interest and thus
% returned.
varargout = num2cell(Q(:,1)');
```

## Interpolant construction

Next, we construct the interpolant, simultaneously for all time steps. Here, we use the intervals [Q(0)] = [4,6], [Q'(0)]
= [0,2], [a] = [1,3], and [b] = [3,5].

```
% Problem dimension
d = 4;

% Define the time span considered
tspan = [0 5];

% Define the number of steps to consider
nsteps = 101;

% Define the objective range of the initial conditions and the
% parameters
range = [4 6;  % [Q(0)]
         0 2;  % [Q'(0)]
         1 3;  % [a]
         3 5]; % [b]

% Maximum number of sparse grid levels to compute
nmax = 3;

% Initialize z
z = [];

% Turn insufficient depth warning off, since it is anticipated.
warning('off', 'MATLAB:spinterp:insufficientDepth');

% Compute increasingly accurate interpolants; use previous results;
% display estimated maximum relative error over all time steps at
% each iteration.
for n = 1:nmax
  options = spset('Vectorized', 'off', 'MinDepth', n, 'MaxDepth', ...
                  n, 'NumberOfOutputs', nsteps, 'PrevResults', z);
  z = spvals('interface_circuit', d, range, options, tspan, nsteps);
  disp(['Current (estimated) maximum relative error over all time' ...
        'steps: ', num2str(z.estRelError)]);
end

% Turn insufficient depth warning back on
warning('on', 'MATLAB:spinterp:insufficientDepth');
```

```
Current (estimated) maximum relative error over all timesteps: 0.64844
Current (estimated) maximum relative error over all timesteps: 0.34119
Current (estimated) maximum relative error over all timesteps: 0.057381
```

## Computing interpolated values

We can now compute interpolated values at each time step, for any combination of parameters within the range that the interpolant
was computed for. The structure z contains all the required information. We only need to select the desired output parameter
(i.e. the time step in this example). To compute 10 randomly distributed values at time t=5 (which is step #101 with the chosen
discretization) within the box [Q(0)] x [Q'(0)] x [a] x [b], we would simply use the following commands:

```
% Compute 10 randomly distributed points in [0,1] and re-scale them to
% the objective range
x = cell(1,4);
for k = 1:d
  x{k} = range(k,1) + rand(1,10) .* (range(k,2) - range(k,1));
end

% Select output parameter #101
z.selectOutput = 101;
% Compute and display interpolated values
y = spinterp(z, x{:})
```

```
y =
  Columns 1 through 7 
   -2.7824   -1.3367   -1.8990   -3.4497   -1.9133   -4.1978   -0.1284
  Columns 8 through 10 
   -4.9939   -8.1073   -3.3928
```

## References

|  |  |
| --- | --- |
| [1] | J. J. Buckley, E. Eslami, and T. Feuring. *Fuzzy Mathematics in Economics and Engineering*. Physica-Verlag, Heidelberg, Germany, 2002. |

|  |  |  |  |  |
| --- | --- | --- | --- | --- |
|  | Interfacing concepts |  | External models |  |
