# Supplementary material for: Multiple Model-Informed Open-Loop Control of Uncertain Intracellular Signaling Dynamics
Source: PLoS Comput Biol. 2014 Apr 10;10(4):e1003546. doi: 10.1371/journal.pcbi.1003546 (PMC3983080; doi:10.1371/journal.pcbi.1003546)
Supplement: Dataset S1 — Matlab code for proposed control algorithm and prediction models. Contains all Matlab code necessary to implement the proposed adaptive weighted multiple-model predictive control algorithm, as well as code for the prediction models. (ZIP) [file pcbi.1003546.s001.zip › AW_MMPC/spinterp_v5.1.1/help/optimization.html]

Optimization (Sparse Grid Interpolation Toolbox)


|  |  |
| --- | --- |
| **Sparse Grid Interpolation Toolbox** |  |

# Optimization

Once a sparse grid interpolant providing a surrogate function or meta-model of an expensive to evaluate model has been obtained, a very common task to be performed is often a search for local/global minimizers or maximizers. Since version 4.0 of the toolbox, several efficient optimization methods are available to perform this task. Furthermore, it is easy to use third-party optimization codes on sparse grid interpolants.

## Contents

- Available algorithms
- Optimizing piecewise linear interpolants
- Optimizing polynomial interpolants
- A high-dimensional example
- Using third-party optimization algorithms

## Available algorithms

The following optimization algorithms are included with the toolbox:

- spcgsearch - suitable for optimizing polynomial sparse grid interpolants.
- spcompsearch - suitable for optimizing piecewise linear sparse grid interpolants.
- spfminsearch - works for all types of interpolants, but usually less efficient than spcompsearch or spcgsearch.
- spmultistart - a multiple random start search method that uses any of the above methods for the local searches.

Many parameters of these algorithms can be configured with an options structure created with the spoptimset function.

## Optimizing piecewise linear interpolants

We consider a simple algebraic test function f, the well-known six-hump camel back function. Here, we visualize f slightly shifted and in logarithmic scaling to cleary show the 6 minima. Two minima are global, indicated by the red triangle.

```
f = @(x,y) (4-2.1.*x.^2+x.^4./3).*x.^2+x.*y+(-4+4.*y.^2).*y.^2;
ezcontour(@(x,y) log(2+f(x,y)), [-3 3 -2 2], 51);
hold on;
plot([ 0.08984201  -0.08984201], [-0.71265640   0.71265640], 'r^');
```

We construct a sequence of piecewise linear interpolants, and optimize them by the spcompsearch function in each step. Here, we use a maximum of N = 705 points, as used by the Clenshaw-Curtis grid of level nmax = 5, to approximate the (global) minimum.

```
nmax = 7;
z = [];
range = [-3 3; -2 2];
f_exact = -1.0316284535

warning('off', 'MATLAB:spinterp:insufficientDepth');
tic;
for n = 1:nmax
  spoptions = spset('MinDepth', n, 'MaxDepth', n, 'PrevResults', z, ...
          'KeepFunctionValues', 'on');
  z = spvals(f,2,range,spoptions);
  [xopt, fval, exitflag, output] = spcompsearch(z,range);
  disp([' grid pnts: ' sprintf('%3d', z.nPoints) ...
        ' | optim fevals: ' sprintf('%3d', output.nFEvals) ...
        ' | fval: ' sprintf('%+5f', fval) ...
        ' | abs. error: ' num2str(abs(f_exact-fval))]);
end
toc;
warning('on', 'MATLAB:spinterp:insufficientDepth');
```

```
f_exact =

   -1.0316

 grid pnts:   5 | optim fevals:   8 | fval: +0.000000 | abs. error: 1.0316
 grid pnts:  13 | optim fevals:   8 | fval: +0.000000 | abs. error: 1.0316
 grid pnts:  29 | optim fevals:  12 | fval: -0.750000 | abs. error: 0.28163
 grid pnts:  65 | optim fevals:  12 | fval: -0.984375 | abs. error: 0.047253
 grid pnts: 145 | optim fevals:  20 | fval: -0.986956 | abs. error: 0.044672
 grid pnts: 321 | optim fevals:  20 | fval: -1.026468 | abs. error: 0.0051603
 grid pnts: 705 | optim fevals:  24 | fval: -1.031286 | abs. error: 0.00034234
Elapsed time is 0.641286 seconds.
```

## Optimizing polynomial interpolants

If the objective function is smooth, polynomial interpolants are a good choice. In the example below, by using the Chebyshev-Gauss-Lobatto
sparse grid, we achieve an exponential convergence rate for the considered analytic function. To further reduce the number
of sparse grid points, we use a dimension-adaptive interpolant. We start with N = 5 nodes, and increase the number of nodes by about a factor of 1.5 in each step of the loop, up to about 100 points.

```
Nmax = 100;
N = 5;
z = [];
warning('off', 'MATLAB:spinterp:maxPointsReached');
tic;
while N <= Nmax
  spoptions = spset('MinPoints', N, 'MaxPoints', N, 'PrevResults', z, ...
    'GridType', 'Chebyshev', 'DimensionAdaptive', 'on', ...
    'KeepFunctionValues', 'on');
  z = spvals(f,2,range,spoptions);
  N = round(z.nPoints .* 1.5);
  [xopt, fval, exitflag, output] = spcgsearch(z,range);
  disp([' grid pnts: ' sprintf('%3d', z.nPoints) ...
        ' | optim fevals: ' sprintf('%3d', output.nFEvals) ...
        ' | fval: ' sprintf('%+5f', fval) ...
        ' | abs. error: ' num2str(abs(f_exact-fval))]);
end
toc;
warning('on', 'MATLAB:spinterp:maxPointsReached');
```

```
 grid pnts:   5 | optim fevals:   1 | fval: +0.000000 | abs. error: 1.0316
 grid pnts:  11 | optim fevals:   9 | fval: +0.000000 | abs. error: 1.0316
 grid pnts:  17 | optim fevals:  20 | fval: -0.537875 | abs. error: 0.49375
 grid pnts:  29 | optim fevals:  29 | fval: -1.031628 | abs. error: 1.9134e-11
 grid pnts:  53 | optim fevals:  30 | fval: -1.031628 | abs. error: 1.924e-11
 grid pnts:  85 | optim fevals:  30 | fval: -1.031628 | abs. error: 1.9262e-11
Elapsed time is 1.146093 seconds.
```

## A high-dimensional example

Let us look at the optimization of a higher-dimensional function. We consider again the function trid.m that was already used to illustrate the dimension-adaptive algorithm:

```
type('trid.m');
```

```
function y = trid(x)
% TRID   Quadratic function with a tridiagonal Hessian.
%   Y = TRID(X)   returns the function value Y for a D-
%   dimensional input vector X.
%
% The test function is due to Arnold Neumaier, listed
% on the global optimization Web page at 
%   http://www.mat.univie.ac.at/~neum/glopt/

d = length(x);
y = sum((x-1).^2) - sum(x(2:d).*x(1:d-1));
```

We let d=100, and compute the known exact minimal value for comparison:

```
d = 100;
range = repmat([-d^2 d^2],d,1);
f_exact = -d*(d+4)*(d-1)/6
```

```
f_exact =
     -171600
```

For high-dimensional problems, it is important to use dimensional adaptivity. Note that here, as well as in the examples above,
we use the KeepFunctionValues property to indicate that the function values obtained during the sparse grid construction should be retained in order to
save time when selecting a good start point for the search.

```
options = spset('DimensionAdaptive', 'on', ...
                'DimadaptDegree', 1, ...
                'FunctionArgType', 'vector', ...
                'RelTol', 1e-3, ...
                'GridType', 'Chebyshev', ...
                'KeepFunctionValues', 'on');

Nmax = 40000;
N = 2*d;
z = [];
warning('off', 'MATLAB:spinterp:maxPointsReached');
tic;
xopt = [];
fval = [];
while N <= Nmax
  spoptions = spset(options, 'MinPoints', N, ...
    'MaxPoints', N, 'PrevResults', z);
  z = spvals(@trid,d,range,spoptions);
  z = sppurge(z);
  spoptoptions = spoptimset('TolFun',1e-6);
  [xopt,fval,exitflag,output] = spcgsearch(z,range,spoptoptions);
  N = round(z.nPoints .* 2);
  disp([' grid pnts: ' sprintf('%5d', z.nPoints) ...
        ' | optim fevals: ' sprintf('%4d', output.nFEvals) ...
        ' | fval: ' sprintf('%+9.1f', fval) ...
        ' | abs. error: ' num2str(abs(f_exact-fval))]);
end
toc;
warning('on', 'MATLAB:spinterp:maxPointsReached');
```

```
 grid pnts:   201 | optim fevals:   11 | fval:      -0.0 | abs. error: 171600
 grid pnts:   443 | optim fevals:   29 | fval:     -18.0 | abs. error: 171582
 grid pnts:   923 | optim fevals:   48 | fval:    -132.0 | abs. error: 171468
 grid pnts:  1883 | optim fevals:   93 | fval:    -537.0 | abs. error: 171063
 grid pnts:  3899 | optim fevals:  117 | fval:   -1202.0 | abs. error: 170398
 grid pnts:  7889 | optim fevals:  188 | fval:   -3293.0 | abs. error: 168307
 grid pnts: 16043 | optim fevals:  248 | fval:  -10725.9 | abs. error: 160874.1483
 grid pnts: 32477 | optim fevals:  305 | fval: -171600.0 | abs. error: 5.6811e-08
Elapsed time is 188.371965 seconds.
```

## Using third-party optimization algorithms

Instead of using the optimization algorithms provided with the Sparse Grid Interpolation Toolbox, you can also use third-party
optimization methods. In the following example, we use fmincon from The Mathwork's Optimization Toolbox on spsurfun to optimize the sparse grid interpolant obtained in the last step of the loop from the example above.

```
optimsetoptions = optimset('GradObj','on', ...
  'LargeScale','off');
[xopt,fval,exitflag,output] = fmincon(@(x) spsurfun(x,z), ...
  range(:,1)+range(:,2))/2,[],[],[],[],range(:,1),range(:,2), ...
  [], optimsetoptions);
disp([' grid pnts: ' sprintf('%5d', z.nPoints) ...
      ' | optim fevals: ' sprintf('%4d', output.funcCount) ...
      ' | fval: ' sprintf('%+9.1f', fval) ...
      ' | abs. error: ' num2str(abs(f_exact-fval))]);
```

```
Optimization terminated: magnitude of directional derivative in search
 direction less than 2*options.TolFun and maximum constraint violation
  is less than options.TolCon.
No active inequalities
 grid pnts: 32477 | optim fevals: 5711 | fval: -171600.0 | abs. error: 1.1176e-07
```

|  |  |  |  |  |
| --- | --- | --- | --- | --- |
|  | Integration |  | Improving performance |  |
