# Supplementary material for: Multiple Model-Informed Open-Loop Control of Uncertain Intracellular Signaling Dynamics
Source: PLoS Comput Biol. 2014 Apr 10;10(4):e1003546. doi: 10.1371/journal.pcbi.1003546 (PMC3983080; doi:10.1371/journal.pcbi.1003546)
Supplement: Dataset S1 — Matlab code for proposed control algorithm and prediction models. Contains all Matlab code necessary to implement the proposed adaptive weighted multiple-model predictive control algorithm, as well as code for the prediction models. (ZIP) [file pcbi.1003546.s001.zip › AW_MMPC/spinterp_v5.1.1/help/performance.html]

Improving Performance (Sparse Grid Interpolation Toolbox)


|  |  |
| --- | --- |
| **Sparse Grid Interpolation Toolbox** |  |

# Improving Performance

The aim of this section is to provide an overview on how to optimize the performance of the Sparse Grid Interpolation Toolbox.

## Contents

- Vectorizing the objective function
- Reusing previous results
- Purging interpolant data
- Vectorized interpolant evaluation

## Vectorizing the objective function

Vectorizing the objective function is most beneficial if the function evaluations are very cheap, in the order of less than
1/100 s. In this case, providing a vectorized function can improve the performance of the `spvals` function. Consider the following
function

and the following two m-files implementing it:

```
type fun.m
```

```
function y = fun(x1, x2)
y = x1 * x2;
y = y^2;
```

```
type fun_vec.m
```

```
function y = fun_vec(x1, x2)
y = x1 .* x2;    % Use '.' before any '^', '*' or '/' to enable
y = y.^2;        % vectorized evaluation of expressions
```

The first m-file allows for evaluation at a single real-valued point only, the second one permits vectorized evaluation. Since
in case of cheap functions, the function calls in Matlab represent a significant overhead, the function evaluation part of
the `spvals` algorithm is much slower if the non-vectorized form is used. This is demonstrated by the following code.

```
tic, z1 = spvals('fun',2); toc;
tic, z2 = spvals('fun_vec',2,[],spset('Vectorized','on')); toc;
z1.fevalTime
z2.fevalTime
```

```
Elapsed time is 0.112452 seconds.
Elapsed time is 0.069006 seconds.
ans =
    0.1021
ans =
    0.0480
```

## Reusing previous results

An important feature of the toolbox is that you do not have to discard previously computed results. A "best practice" is,
therefore, to embed the interpolant construction in a loop. Proceeding in this way has two advantages: First, it gives the
user a maximum of control in monitoring the decay of the estimated interpolation error. Second, it makes it possible to start
with a low number of required points, and to increase this number slowly if the targeted accuracy is not yet achieved. There
are several examples on how to implement such a loop in the provided demos. See, for instance, spadaptanim.m or spcompare.m in the examples directory.

A small example on implementing dimension-adaptive interpolant construction in a loop is provided below.

```
np = 2;
z  = [];
options = spset('Vectorized', 'on', 'DimensionAdaptive', 'on', ...
  'RelTol', inf);
while np < 4000
  options = spset(options, 'PrevResults', z, 'MinPoints', np, 'MaxPoints', np);
  z = spvals('fun_vec',2,[],options);
  np = z.nPoints;
  disp(['np = ' num2str(np) ', e_rel = ', num2str(z.estRelError)]);
  np = np * 2;
end
```

```
np = 5, e_rel = 0.75
np = 13, e_rel = 0.5625
np = 29, e_rel = 0.046875
np = 73, e_rel = 0.011719
np = 177, e_rel = 0.0029297
np = 417, e_rel = 0.00073242
np = 897, e_rel = 6.1035e-05
np = 1921, e_rel = 4.5776e-05
np = 4097, e_rel = 3.8147e-06
```

## Purging interpolant data

Since version v3.2 of the toolbox, a new function called sppurge is available. This function serves to "purge" or "clean up" the interpolant data from subgrids that do not contribute significantly
to the result. This is done by introducing a drop tolerance that is applied to the hierarchical surpluses. Subgrids where
the absolute value of all hierarchical surpluses fall below this drop tolerance are marked and neglected during the interpolation
process. By default, very conservative purging parameters are used, guaranteeing that the accuracy of the interpolation will
not be affected up to about the 12th significant digit. However, if the accuracy requirements are lower, the user may use
higher drop tolerances, and thus, trade improved interpolation speed against lower accuracy. This is illustrated by the following
example.

We assume that an interpolant was computed for the function fun\_vec by the code above with 4097 points, using piecewise multilinear basis functions. The following code generates a plot that
shows the time required to compute 1000 randomly sampled points for different drop tolerances. The maximum absolute error
is shown for comparison. This example only uses absolute drop tolerances (the relative drop tolerance is set to zero).

```
% Define drop tolerances
dropTols = [1e-5, 1e-4, 1e-3];

% Generate 1000 random points
rand('state',0);
x = rand(1000,1); y = rand(1000,1);

% Compute exact function values
f_exact = fun_vec(x,y);

e = zeros(3,1); t = zeros(3,1);
for k = 1:3
  % Purge interpolant with drop tolerance
  z = sppurge(z,spset('DropTol', [dropTols(k), 0]));
  % Interpolate and measure time
  tic, ip = spinterp(z, x, y); t(k) = toc;
  % Compute maximum error
  e(k) = max(abs(f_exact - ip));
end

% Plot results
subplot(1,2,1);
bar(t, 'b');
set(gca,'XTickLabel', {'1e-5','1e-4','1e-3'})
xlabel('Abs. drop tolerance');
ylabel('Computing time [s]');
subplot(1,2,2);
bar(log10(e), 'r');
set(gca,'XTickLabel', {'1e-5','1e-4','1e-3'})
set(gca,'YDir','reverse');
set(gca,'YLim', [-6 -2]);
set(gca,'YTick',[-5 -4 -3]);
set(gca,'YTickLabel', {'1e-5','1e-4','1e-3'})
xlabel('Abs. drop tolerance');
ylabel('Max. absolute error');
```

For another example using the default relative drop tolerance, see sppurge.

## Vectorized interpolant evaluation

The spinterp function is designed for vectorized evaluation. Since the sparse grid algorithm involves more computational overhead than
other, simpler interpolation methods, and due to the fact that Matlab is relatively slow if many function calls are performed
(since it is an interpreted language), it is recommended to evaluate as many interpolation points at a time as possible. The
following code illustrates non-vectorized vs. vectorized evaluation at 1000 points for the interpolant from above.

```
% Non-vectorized interpolation
tic
for k = 1:1000
  ip = spinterp(z,x(k),y(k));
end
toc

% Vectorized interpolation
tic, ip = spinterp(z,x,y); toc
```

```
Elapsed time is 2.526127 seconds.
Elapsed time is 0.045744 seconds.
```

|  |  |  |  |  |
| --- | --- | --- | --- | --- |
|  | Optimization |  | Interfacing concepts |  |
