# Supplementary material for: Multiple Model-Informed Open-Loop Control of Uncertain Intracellular Signaling Dynamics
Source: PLoS Comput Biol. 2014 Apr 10;10(4):e1003546. doi: 10.1371/journal.pcbi.1003546 (PMC3983080; doi:10.1371/journal.pcbi.1003546)
Supplement: Dataset S1 — Matlab code for proposed control algorithm and prediction models. Contains all Matlab code necessary to implement the proposed adaptive weighted multiple-model predictive control algorithm, as well as code for the prediction models. (ZIP) [file pcbi.1003546.s001.zip › AW_MMPC/spinterp_v5.1.1/help/plotgrid.html]

plotgrid :: (Sparse Grid Interpolation Toolbox)


|  |  |
| --- | --- |
| **Sparse Grid Interpolation Toolbox** |  |

# plotgrid

Plots a sparse grid.

## Syntax

`plotgrid(N,D)`  
`plotgrid(N,D,OPTIONS)`  
`H = plotgrid(...)`

## Description

`plotgrid(N,D)` Plots the sparse grid of level `N` and dimension `D`. By default, the Clenshaw-Curtis sparse grid type is selected.

`plotgrid(N,D,OPTIONS)` Plots the sparse grid, but with the grid type as specified in `OPTIONS`. `OPTIONS` must be a structure created with the `spset` function.

`H = PLOTGRID(...)` Returns a vector of handles to the grid points (useful for changing the look of the plotted grid).

## Examples

The following statements can be used to plot the Chebyshev-Gauss-Lobatto sparse grid of level `N = 4` in three dimensions, highlighting the grid points of the levels in different colors:

```
options = spset('GridType', 'Chebyshev');
n = 4;
h = plotgrid(n,3,options);
cols = brighten(jet(n+1),-1);
legendstr = cell(1,n+1);
for k = 0:n
  set(h(k+1), 'Color', cols(k+1,:), 'MarkerSize', 20);
  legendstr{k+1} = ['n = ' num2str(k)];
end
grid on;
legend(legendstr);
```

## See Also

`cmpgrids`,
`plotindices`,
`spgrid`.

|  |
| --- |
|  |
