# Supplementary material for: Multiple Model-Informed Open-Loop Control of Uncertain Intracellular Signaling Dynamics
Source: PLoS Comput Biol. 2014 Apr 10;10(4):e1003546. doi: 10.1371/journal.pcbi.1003546 (PMC3983080; doi:10.1371/journal.pcbi.1003546)
Supplement: Dataset S1 — Matlab code for proposed control algorithm and prediction models. Contains all Matlab code necessary to implement the proposed adaptive weighted multiple-model predictive control algorithm, as well as code for the prediction models. (ZIP) [file pcbi.1003546.s001.zip › AW_MMPC/spinterp_v5.1.1/help/plotindices.html]

plotindices :: (Sparse Grid Interpolation Toolbox)


|  |  |
| --- | --- |
| **Sparse Grid Interpolation Toolbox** |  |

# plotindices

Visualizes the index sets of a two-dimensional dimension-adaptive sparse grid.

## Syntax

`plotindices(Z)`

## Description

`plotindices(Z)` Plots the set of multi-indices `S_k` of a two-dimensional dimension-adaptive sparse grid interpolant `A_{S_k}(f)`. `Z` must be the sparse grid data as returned by `spvals`. `spvals` must be called with the option `'DimensionAdaptive'` switched `'on'` (this can be done using `spset`).

## Examples

The following code constructs a dimension-adaptive sparse grid interpolant of the function

using greedy grid refinement (the degree of dimensional adaptivity is set to 1). The default interpolation box is range = [0,1]^2.

```
f = inline('sin(10.*x)+y.^2');
options = spset('DimensionAdaptive', 'on', 'DimAdaptDegree', 1);
z = spvals(f, 2, [], options)
```

```
z = 

               vals: {[149x1 double]}
           gridType: 'Clenshaw-Curtis'
                  d: 2
              range: []
        estRelError: 0.0018
        estAbsError: 0.0039
         fevalRange: [-0.9589 1.2500]
         minGridVal: [0.5000 0]
         maxGridVal: [0.1562 0.5000]
            nPoints: 149
          fevalTime: 0.3286
    surplusCompTime: 0.0089
            indices: [1x1 struct]
           maxLevel: [7 4]
      activeIndices: [3x1 uint32]
     activeIndices2: [13x1 uint32]
                  E: [1x13 double]
                  G: [13x1 double]
                 G2: [13x1 double]
       maxSetPoints: 7
           dimAdapt: 1
```

The resulting interpolant is plotted using Matlab's ezmesh command and an anonymous function containing the call to spinterp. Plotting the multi-index sets used by the interpolant reveals that the refinement is more dense in the x-direction, since
more points are required to resolve the oscillation of the sine curve. Due to the greedy refinement, only a single index (2,2)
is computed in joint dimensions, since the error indicator of the multi-index (2,2) is equal to zero (f is a separable function).

```
subplot(1,2,1);
ezmesh(@(x,y) spinterp(z,x,y), [0 1]);
axis square;
subplot(1,2,2);
plotindices(z);
```

## See Also

`plotgrid`,
`plotindices`,
`spgrid`.

|  |
| --- |
|  |
