# Supplementary material for: Multiple Model-Informed Open-Loop Control of Uncertain Intracellular Signaling Dynamics
Source: PLoS Comput Biol. 2014 Apr 10;10(4):e1003546. doi: 10.1371/journal.pcbi.1003546 (PMC3983080; doi:10.1371/journal.pcbi.1003546)
Supplement: Dataset S1 — Matlab code for proposed control algorithm and prediction models. Contains all Matlab code necessary to implement the proposed adaptive weighted multiple-model predictive control algorithm, as well as code for the prediction models. (ZIP) [file pcbi.1003546.s001.zip › AW_MMPC/spinterp_v5.1.1/help/polynomial.html]

Polynomial basis functions (Sparse Grid Interpolation Toolbox)


|  |  |
| --- | --- |
| **Sparse Grid Interpolation Toolbox** |  |

# Polynomial basis functions

The piecewise multilinear approach can be significantly improved by using higher-order basis functions, such as the Lagrangian characteristic polynomials. The approximation properties of sparse grid interpolation techniques using polynomial basis functions have been studied extensively in [4], where error bounds depending on the smoothness of the function were derived.

From the one-dimensional case, we know that one should not use equidistant nodes for higher-order polynomial interpolation. This directly suggests using Chebyshev-based node distributions. Since an additional requirement of an efficient sparse grid algorithm is the nesting of the sets of nodes, the Chebyshev Gauss-Lobatto nodes are clearly the best choice, and are therefore also suggested in [4]. In this toolbox, this grid type (CGL) is selected by the value "**`Chebyshev`**" for the `GridType` property configurable with the `spset` function.

Since version 5.0.0, an additional polynomial sparse grid is available, the Gauss-Patterson sparse grid. This grid is based on the abscissae of Gauss-Patterson integration. The Gauss-Patterson formula is a nested quadrature rule that achieves a higher degree of exactness than integration at the Chebyshev Gauss-Lobatto nodes. See [9,10] for additional details.

For a detailed description of the polynomial basis functions implemented here, please see [3, ch. 3], and the references stated therein. Since version
v3.2, the toolbox uses an improved construction algorithm employing the fast
discrete cosine transform, see [6].

## Accuracy of polynomial interpolation

From the error bounds of the univariate case, the following general error bounds depending on the smoothness of the objective function f are derived in [4]. For f in Fdk,

|  |  |
| --- | --- |
|  |  |

the order of the interpolation error in the maximum norm is given by

where Aq,d(f) denotes the sparse grid interpolant of f, and N denotes the number of grid points of the sparse grids of type CGL. Note that the number of grid points N of Aq,d(f) can be computed by `spdim(q-d,d)`.

## Number of grid points

The number of grid points of the CGL-grid is identical to the one of the Clenshaw-Curtis (CC) grid. The number of grid points of the Gauss-Patterson grid is identical to the one of the NB grid.

The following graph illustrates the sparse grids of level 0 and level 2 of the CGL-grid in two and three dimensions.

## When should I use polynomial rather than linear basis functions?

There is obviously some trade-off between the accuracy gain and the computing time required to construct as well as interpolate the interpolant. Since the higher-order accuracy only becomes effective with increasing number of nodes, we recommend to use the polynomial approach only if the following two conditions are met:

- The objective function to be recovered is known to be very smooth.- High relative accuracies smaller than 10-2 are required.

|  |  |  |  |  |
| --- | --- | --- | --- | --- |
|  | Linear basis functions |  | Dimensional adaptivity |  |
