# Supplementary material for: Multiple Model-Informed Open-Loop Control of Uncertain Intracellular Signaling Dynamics
Source: PLoS Comput Biol. 2014 Apr 10;10(4):e1003546. doi: 10.1371/journal.pcbi.1003546 (PMC3983080; doi:10.1371/journal.pcbi.1003546)
Supplement: Dataset S1 — Matlab code for proposed control algorithm and prediction models. Contains all Matlab code necessary to implement the proposed adaptive weighted multiple-model predictive control algorithm, as well as code for the prediction models. (ZIP) [file pcbi.1003546.s001.zip › AW_MMPC/spinterp_v5.1.1/help/release_notes.html]

Release notes (Sparse Grid Interpolation Toolbox)


|  |  |
| --- | --- |
| **Sparse Grid Interpolation Toolbox** |  |

# Release notes

## V5.1.1

### Bug fixes

- Fixed bug in grid point cropping in `spfindopt` used to find best optimization start point (added tolerance, fixed indices range).- Fixed non-critical "Out of range value or NaN computed in integer arithmetic" warning occurring occasionally in the dimension-adaptive algorithm (seen in Matlab v7.4.0.287, R2007a).

## V5.1.0

### New features

- Added new degree balancing strategy for improved handling of greedy, dimension-adaptive vs. conservative grid refinement. See Advanced Topics, Degree of Dimensional Adaptivity for more information.

### Bug fixes

- Fixed bug in dimension-adaptive spvals algorithm: in rare cases, an incorrect warning message was issued (reported 'no more active indices available' instead of 'maximum number of points reached prior to convergence').- Added warning message when maximum supported depth for Chebyshev grid (level 10) is exceeded.

## V5.0.0

### New features

- Added full numerical integration capability for both regular and dimension-adaptive sparse grid interpolants. See Advanced Topics, Integration for more information.- Added Gauss-Patterson grid type.- `MaxDepth` now also applies to the dimension-adaptive algorithm. If `MaxDepth` is reached with respect to a coordinate direction, this direction is no longer refined further.

### Bug fixes

- Fixed bug in dimension-adaptive algorithm: level vector was not reset.

## V4.0.0

### New features

- Added efficient optimization algorithms specifically implemented to optimize sparse grid interpolants. See Advanced Topics, Optimization for more information.

### Bug fixes

- Modified augmented gradient computation for piecewise linear sparse grids to ensure that the gradient vector is `0` at an extremum.- Improved allocation of grids points in the dimension-adaptive algorithm when handling very large dimensions `d > 100`. The maximum problem dimension that can be handled is now `d = 65534`.- Fixed display of menus on Web page that occurred with Internet Explorer 7.

## V3.5.1

### New features

- Added support of calling `spinterp` with the points to evaluate as a single matrix (special thanks to Daniel Harenberg for the suggestion and the provided code).

### Bug fixes

- Fixed bug in gradient computation that caused a wrong scaling of derivatives when range intervals were not equal to one (problem found and solved by Katrin Grau).

## V3.5.0

### New features

- Added capability of computing accurate gradient vectors. This can be done when evaluating the interpolant, at a small additional cost. This major enhancement is available for the Clenshaw-Curtis and the Chebyshev sparse grid, for both the regular and the dimension-adaptive algorithms. See Advanced Topics, Derivatives for more information.

### Bug fixes

- It is now possible to evaluate the Chebyshev sparse grid interpolant **outside** of the box range defined during the call to `spvals`. This previously caused an error in the `barypdstep` algorithm. However, doing this is usually not recommended, since, in most cases, the quality of the approximation decreases rapidly outside of the specified box.- Fixed figure showing piecewise multilinear sparse grid types in the printed documentation (Chebyshev grid figure was shown twice instead).- Fixed dimension-adaptive example in documentation (value for `DimadaptDegree` argument in call to `spset` was missing).

## V3.2.0

### New features

- Construction of hierarchical polynomial sparse grid interpolants using the fast discrete cosine transform. This new algorithm greatly improves the efficiency of the Chebyshev-type sparse grid in lower dimensions and the dimension-adaptive Chebyshev-type sparse grid when just few dimensions are important. See demo `timespvalsdct.m`.- Purging algorithm. Optionally cleans up sparse grid interpolants from less important sub-grids to increase evaluation speed. See `sppurge`.- Added additional documentation on optimizing the performance of the Sparse Grid Interpolation Toolbox.

### Bug fixes

- Changed warning message that was displayed if the maximum number of points was reached prior to reaching the requested error tolerance when performing a dimension-adaptive interpolant construction with `spvals`. It now displays the current error estimate and the target error tolerances.- The private function `spcmpvalsccsp.m` was missing the pre-allocation of `backvec`, causing performance losses.

## V3.0.1 beta

### New features

Compared to the previous major release V2.1.1, the following features have been added:

- Polynomial basis functions at the Chebyshev-Gauss-Lobatto sparse grid. Efficient implementation using Barycentric interpolation.- Dimension-adaptive algorithm for the treatment of higher-dimensional problems (based on piecewise linear basis functions at the Clenshaw-Curtis grid and based on polynomial basis functions at the Chebyshev-Gauss-Lobatto grid).- New data structure to store the interpolant's hierarchical surpluses that is especially efficient in case of higher-dimensional problems.- Much improved documentation and extensive demo suite. The documentation now includes Matlab help browser support, HTML documentation of all functions, additional examples, and a help index.

### Upgrade issues/backward compatibility

Please note the following minor changes affecting the user interface compared to the previous release V2.1.1:

- The toolbox now **requires at least Matlab Version 7.0 (R14)**, since the toolbox makes heavy use of the integer data formats and integer arithmetic available since this Matlab release.- To avoid naming ambiguity/conflicts, the initialization command was renamed from `init` to `spinit`.- The structure generated by `spvals` (usually denoted by `z`) now stores the hierarchical surpluses `z.vals` in the new, improved sparse data storage format. Therefore, manual accessing of the hierarchical surpluses of the sub-grids has to be done differently. However, the previous behavior of `spvals` (i.e. in case of d = 2, n = 2, the hierarchical surpluses of the indices sub-grids with multi-index (0,2), (1,1), (0,2) were stored as the (n+1)th element of the cell array `z.vals`) can be achieved by generating a sparse grid `OPTIONS` structure with the switch `SparseIndices` set to 'off' using the `spset` command.

### Bug fixes

- Version V3.0 beta used a default degree of dimensional adaptivity of 0.5 instead of the value 0.9 mentioned in the documentation. The private subroutine barypdstep.m used a non-initialized double array that caused a severe performance loss. (Fixed)- Version V2.1.1 of the toolbox produced a non-critical "structure assignment warning" in Matlab Version 7 R14 SP2 during the call to `spvals`. (Fixed)

|  |
| --- |
|  |
