# Supplementary material for: Multiple Model-Informed Open-Loop Control of Uncertain Intracellular Signaling Dynamics
Source: PLoS Comput Biol. 2014 Apr 10;10(4):e1003546. doi: 10.1371/journal.pcbi.1003546 (PMC3983080; doi:10.1371/journal.pcbi.1003546)
Supplement: Dataset S1 — Matlab code for proposed control algorithm and prediction models. Contains all Matlab code necessary to implement the proposed adaptive weighted multiple-model predictive control algorithm, as well as code for the prediction models. (ZIP) [file pcbi.1003546.s001.zip › AW_MMPC/spinterp_v5.1.1/help/run_demos.html]

Demos: Reference Runs (Sparse Grid Interpolation Toolbox)


|  |  |
| --- | --- |
| **Sparse Grid Interpolation Toolbox** |  |

# Demos: Reference Runs

In the following, we provide the output of the provided demo programs of the software as a reference. The results shown here
were computed using MATLAB Version 7.0 (R14SP2) running on a Linux version 2.4.27 workstation equipped with one Intel Pentium
4 CPU 3.2 GHz Processor and 2 GB of memory.

## Contents

- cmpgrids
- spdemo
- spdemovarout
- spcompare
- spcomparepoly
- spcomparederiv
- spadaptdemo
- spadaptanim
- spadapterror
- timespvals
- timespinterp
- timespvalsdct
- timespderiv

## cmpgrids

```
cmpgrids
```

## spdemo

```
spdemo
```

```
Sparse grid representation of the function:

z = 

               vals: {1x9 cell}
           gridType: 'Clenshaw-Curtis'
                  d: 2
              range: [2x2 double]
           maxLevel: 8
        estRelError: 0.0090
        estAbsError: 0.0089
         fevalRange: [0.0049 0.9975]
         minGridVal: [1 0]
         maxGridVal: [0.0625 0.6250]
            nPoints: 1537
          fevalTime: 0.0326
    surplusCompTime: 0.0870
```

## spdemovarout

```
spdemovarout
```

## spcompare

```
rand('seed',0); spcompare
```

```
Problem dimension: d = 3
Current test function: 1
  Grid: Maximum
    n:  0, spvals:  0.01 [s], spinterp:  0.04 [s], e_max: 2.082e-02.
    n:  1, spvals:  0.02 [s], spinterp:  0.10 [s], e_max: 5.472e-03.
    n:  2, spvals:  0.03 [s], spinterp:  0.19 [s], e_max: 1.404e-03.
    n:  3, spvals:  0.05 [s], spinterp:  0.32 [s], e_max: 3.406e-04.
    n:  4, spvals:  0.12 [s], spinterp:  0.50 [s], e_max: 9.049e-05.
    n:  5, spvals:  0.33 [s], spinterp:  0.73 [s], e_max: 2.251e-05.
    n:  6, spvals:  0.91 [s], spinterp:  1.03 [s], e_max: 5.544e-06.
    n:  7, spvals:  2.63 [s], spinterp:  1.38 [s], e_max: 1.367e-06.
 
  Grid: NoBoundary
    n:  0, spvals:  0.01 [s], spinterp:  0.00 [s], e_max: 6.291e-01.
    n:  1, spvals:  0.01 [s], spinterp:  0.01 [s], e_max: 1.144e-01.
    n:  2, spvals:  0.02 [s], spinterp:  0.02 [s], e_max: 1.957e-02.
    n:  3, spvals:  0.02 [s], spinterp:  0.04 [s], e_max: 2.888e-03.
    n:  4, spvals:  0.03 [s], spinterp:  0.08 [s], e_max: 4.944e-04.
    n:  5, spvals:  0.04 [s], spinterp:  0.14 [s], e_max: 1.022e-04.
    n:  6, spvals:  0.10 [s], spinterp:  0.22 [s], e_max: 2.628e-05.
    n:  7, spvals:  0.27 [s], spinterp:  0.32 [s], e_max: 6.685e-06.
    n:  8, spvals:  0.82 [s], spinterp:  0.46 [s], e_max: 1.695e-06.
    n:  9, spvals:  2.57 [s], spinterp:  0.62 [s], e_max: 4.465e-07.
 
  Grid: Clenshaw-Curtis
    n:  0, spvals:  0.01 [s], spinterp:  0.00 [s], e_max: 6.291e-01.
    n:  1, spvals:  0.01 [s], spinterp:  0.01 [s], e_max: 8.631e-02.
    n:  2, spvals:  0.02 [s], spinterp:  0.02 [s], e_max: 1.162e-02.
    n:  3, spvals:  0.02 [s], spinterp:  0.05 [s], e_max: 1.853e-03.
    n:  4, spvals:  0.02 [s], spinterp:  0.10 [s], e_max: 4.773e-04.
    n:  5, spvals:  0.03 [s], spinterp:  0.17 [s], e_max: 1.365e-04.
    n:  6, spvals:  0.04 [s], spinterp:  0.28 [s], e_max: 3.399e-05.
    n:  7, spvals:  0.06 [s], spinterp:  0.43 [s], e_max: 8.348e-06.
    n:  8, spvals:  0.15 [s], spinterp:  0.62 [s], e_max: 2.114e-06.
    n:  9, spvals:  0.39 [s], spinterp:  0.87 [s], e_max: 5.395e-07.
    n: 10, spvals:  1.08 [s], spinterp:  1.17 [s], e_max: 1.505e-07.
    n: 11, spvals:  3.05 [s], spinterp:  1.52 [s], e_max: 3.774e-08.
 
Current test function: 2
  Grid: Maximum
    n:  0, spvals:  0.01 [s], spinterp:  0.04 [s], e_max: 2.590e-02.
    n:  1, spvals:  0.02 [s], spinterp:  0.10 [s], e_max: 1.026e-02.
    n:  2, spvals:  0.02 [s], spinterp:  0.19 [s], e_max: 3.023e-03.
    n:  3, spvals:  0.05 [s], spinterp:  0.33 [s], e_max: 8.083e-04.
    n:  4, spvals:  0.11 [s], spinterp:  0.50 [s], e_max: 2.226e-04.
    n:  5, spvals:  0.31 [s], spinterp:  0.74 [s], e_max: 5.946e-05.
    n:  6, spvals:  0.90 [s], spinterp:  1.03 [s], e_max: 1.701e-05.
    n:  7, spvals:  2.62 [s], spinterp:  1.40 [s], e_max: 3.998e-06.
 
  Grid: NoBoundary
    n:  0, spvals:  0.01 [s], spinterp:  0.00 [s], e_max: 1.042e-01.
    n:  1, spvals:  0.01 [s], spinterp:  0.01 [s], e_max: 4.942e-02.
    n:  2, spvals:  0.02 [s], spinterp:  0.02 [s], e_max: 9.395e-03.
    n:  3, spvals:  0.02 [s], spinterp:  0.04 [s], e_max: 3.584e-03.
    n:  4, spvals:  0.03 [s], spinterp:  0.08 [s], e_max: 9.649e-04.
    n:  5, spvals:  0.04 [s], spinterp:  0.14 [s], e_max: 1.551e-04.
    n:  6, spvals:  0.10 [s], spinterp:  0.22 [s], e_max: 3.802e-05.
    n:  7, spvals:  0.27 [s], spinterp:  0.33 [s], e_max: 1.145e-05.
    n:  8, spvals:  0.82 [s], spinterp:  0.46 [s], e_max: 3.255e-06.
    n:  9, spvals:  2.58 [s], spinterp:  0.62 [s], e_max: 9.271e-07.
 
  Grid: Clenshaw-Curtis
    n:  0, spvals:  0.01 [s], spinterp:  0.00 [s], e_max: 1.042e-01.
    n:  1, spvals:  0.02 [s], spinterp:  0.01 [s], e_max: 2.576e-02.
    n:  2, spvals:  0.02 [s], spinterp:  0.02 [s], e_max: 9.905e-03.
    n:  3, spvals:  0.02 [s], spinterp:  0.05 [s], e_max: 3.291e-03.
    n:  4, spvals:  0.02 [s], spinterp:  0.10 [s], e_max: 1.082e-03.
    n:  5, spvals:  0.03 [s], spinterp:  0.17 [s], e_max: 3.555e-04.
    n:  6, spvals:  0.04 [s], spinterp:  0.28 [s], e_max: 1.038e-04.
    n:  7, spvals:  0.07 [s], spinterp:  0.43 [s], e_max: 3.321e-05.
    n:  8, spvals:  0.14 [s], spinterp:  0.62 [s], e_max: 8.394e-06.
    n:  9, spvals:  0.38 [s], spinterp:  0.87 [s], e_max: 2.580e-06.
    n: 10, spvals:  1.08 [s], spinterp:  1.17 [s], e_max: 7.148e-07.
    n: 11, spvals:  3.05 [s], spinterp:  1.53 [s], e_max: 1.843e-07.
 
Current test function: 3
  Grid: Maximum
    n:  0, spvals:  0.01 [s], spinterp:  0.04 [s], e_max: 1.485e-01.
    n:  1, spvals:  0.02 [s], spinterp:  0.10 [s], e_max: 5.552e-02.
    n:  2, spvals:  0.02 [s], spinterp:  0.19 [s], e_max: 1.177e-02.
    n:  3, spvals:  0.05 [s], spinterp:  0.32 [s], e_max: 4.404e-03.
    n:  4, spvals:  0.11 [s], spinterp:  0.50 [s], e_max: 1.253e-03.
    n:  5, spvals:  0.31 [s], spinterp:  0.73 [s], e_max: 3.538e-04.
    n:  6, spvals:  0.90 [s], spinterp:  1.02 [s], e_max: 8.091e-05.
    n:  7, spvals:  2.63 [s], spinterp:  1.38 [s], e_max: 2.556e-05.
 
  Grid: NoBoundary
    n:  0, spvals:  0.01 [s], spinterp:  0.00 [s], e_max: 5.684e-01.
    n:  1, spvals:  0.01 [s], spinterp:  0.01 [s], e_max: 4.165e-01.
    n:  2, spvals:  0.02 [s], spinterp:  0.02 [s], e_max: 2.490e-01.
    n:  3, spvals:  0.02 [s], spinterp:  0.04 [s], e_max: 1.245e-01.
    n:  4, spvals:  0.03 [s], spinterp:  0.08 [s], e_max: 5.412e-02.
    n:  5, spvals:  0.04 [s], spinterp:  0.14 [s], e_max: 1.997e-02.
    n:  6, spvals:  0.10 [s], spinterp:  0.21 [s], e_max: 5.983e-03.
    n:  7, spvals:  0.27 [s], spinterp:  0.32 [s], e_max: 1.325e-03.
    n:  8, spvals:  0.83 [s], spinterp:  0.46 [s], e_max: 1.718e-04.
    n:  9, spvals:  2.60 [s], spinterp:  0.63 [s], e_max: 6.234e-05.
 
  Grid: Clenshaw-Curtis
    n:  0, spvals:  0.01 [s], spinterp:  0.00 [s], e_max: 5.684e-01.
    n:  1, spvals:  0.01 [s], spinterp:  0.01 [s], e_max: 3.733e-01.
    n:  2, spvals:  0.02 [s], spinterp:  0.02 [s], e_max: 1.034e-01.
    n:  3, spvals:  0.02 [s], spinterp:  0.05 [s], e_max: 9.989e-02.
    n:  4, spvals:  0.02 [s], spinterp:  0.10 [s], e_max: 3.256e-02.
    n:  5, spvals:  0.03 [s], spinterp:  0.17 [s], e_max: 6.465e-03.
    n:  6, spvals:  0.04 [s], spinterp:  0.28 [s], e_max: 4.184e-03.
    n:  7, spvals:  0.06 [s], spinterp:  0.43 [s], e_max: 1.172e-03.
    n:  8, spvals:  0.15 [s], spinterp:  0.63 [s], e_max: 3.437e-04.
    n:  9, spvals:  0.39 [s], spinterp:  0.86 [s], e_max: 8.140e-05.
    n: 10, spvals:  1.08 [s], spinterp:  1.16 [s], e_max: 2.276e-05.
    n: 11, spvals:  3.06 [s], spinterp:  1.53 [s], e_max: 5.808e-06.
 
Current test function: 4
  Grid: Maximum
    n:  0, spvals:  0.01 [s], spinterp:  0.04 [s], e_max: 3.339e-01.
    n:  1, spvals:  0.02 [s], spinterp:  0.10 [s], e_max: 2.097e-01.
    n:  2, spvals:  0.02 [s], spinterp:  0.19 [s], e_max: 1.000e-01.
    n:  3, spvals:  0.05 [s], spinterp:  0.32 [s], e_max: 2.992e-02.
    n:  4, spvals:  0.11 [s], spinterp:  0.50 [s], e_max: 9.766e-03.
    n:  5, spvals:  0.31 [s], spinterp:  0.73 [s], e_max: 3.026e-03.
    n:  6, spvals:  0.91 [s], spinterp:  1.03 [s], e_max: 8.032e-04.
    n:  7, spvals:  2.62 [s], spinterp:  1.38 [s], e_max: 2.657e-04.
 
  Grid: NoBoundary
    n:  0, spvals:  0.01 [s], spinterp:  0.00 [s], e_max: 5.057e-01.
    n:  1, spvals:  0.01 [s], spinterp:  0.01 [s], e_max: 9.416e-01.
    n:  2, spvals:  0.02 [s], spinterp:  0.02 [s], e_max: 5.588e-01.
    n:  3, spvals:  0.02 [s], spinterp:  0.04 [s], e_max: 1.215e-01.
    n:  4, spvals:  0.03 [s], spinterp:  0.08 [s], e_max: 2.914e-02.
    n:  5, spvals:  0.04 [s], spinterp:  0.14 [s], e_max: 1.089e-02.
    n:  6, spvals:  0.09 [s], spinterp:  0.22 [s], e_max: 3.201e-03.
    n:  7, spvals:  0.27 [s], spinterp:  0.32 [s], e_max: 9.707e-04.
    n:  8, spvals:  0.82 [s], spinterp:  0.45 [s], e_max: 2.458e-04.
    n:  9, spvals:  2.59 [s], spinterp:  0.62 [s], e_max: 5.818e-05.
 
  Grid: Clenshaw-Curtis
    n:  0, spvals:  0.01 [s], spinterp:  0.00 [s], e_max: 5.057e-01.
    n:  1, spvals:  0.02 [s], spinterp:  0.01 [s], e_max: 5.859e-01.
    n:  2, spvals:  0.02 [s], spinterp:  0.02 [s], e_max: 2.055e-01.
    n:  3, spvals:  0.02 [s], spinterp:  0.05 [s], e_max: 1.346e-01.
    n:  4, spvals:  0.02 [s], spinterp:  0.10 [s], e_max: 5.354e-02.
    n:  5, spvals:  0.03 [s], spinterp:  0.17 [s], e_max: 2.046e-02.
    n:  6, spvals:  0.04 [s], spinterp:  0.28 [s], e_max: 7.167e-03.
    n:  7, spvals:  0.07 [s], spinterp:  0.43 [s], e_max: 2.352e-03.
    n:  8, spvals:  0.15 [s], spinterp:  0.62 [s], e_max: 7.053e-04.
    n:  9, spvals:  0.38 [s], spinterp:  0.87 [s], e_max: 2.334e-04.
    n: 10, spvals:  1.09 [s], spinterp:  1.17 [s], e_max: 6.823e-05.
    n: 11, spvals:  3.05 [s], spinterp:  1.53 [s], e_max: 1.959e-05.
 
Current test function: 5
  Grid: Maximum
    n:  0, spvals:  0.01 [s], spinterp:  0.04 [s], e_max: 6.180e-01.
    n:  1, spvals:  0.02 [s], spinterp:  0.10 [s], e_max: 5.439e-01.
    n:  2, spvals:  0.02 [s], spinterp:  0.19 [s], e_max: 4.147e-01.
    n:  3, spvals:  0.05 [s], spinterp:  0.32 [s], e_max: 2.716e-01.
    n:  4, spvals:  0.11 [s], spinterp:  0.50 [s], e_max: 1.924e-01.
    n:  5, spvals:  0.31 [s], spinterp:  0.73 [s], e_max: 1.037e-01.
    n:  6, spvals:  0.91 [s], spinterp:  1.03 [s], e_max: 6.731e-02.
    n:  7, spvals:  2.63 [s], spinterp:  1.38 [s], e_max: 3.346e-02.
 
  Grid: NoBoundary
    n:  0, spvals:  0.01 [s], spinterp:  0.00 [s], e_max: 6.545e-01.
    n:  1, spvals:  0.01 [s], spinterp:  0.01 [s], e_max: 6.437e-01.
    n:  2, spvals:  0.02 [s], spinterp:  0.02 [s], e_max: 5.727e-01.
    n:  3, spvals:  0.02 [s], spinterp:  0.04 [s], e_max: 4.115e-01.
    n:  4, spvals:  0.03 [s], spinterp:  0.08 [s], e_max: 4.338e-01.
    n:  5, spvals:  0.04 [s], spinterp:  0.14 [s], e_max: 1.901e-01.
    n:  6, spvals:  0.10 [s], spinterp:  0.22 [s], e_max: 1.648e-01.
    n:  7, spvals:  0.27 [s], spinterp:  0.32 [s], e_max: 6.769e-02.
    n:  8, spvals:  0.83 [s], spinterp:  0.45 [s], e_max: 3.101e-02.
    n:  9, spvals:  2.58 [s], spinterp:  0.62 [s], e_max: 1.499e-02.
 
  Grid: Clenshaw-Curtis
    n:  0, spvals:  0.01 [s], spinterp:  0.00 [s], e_max: 6.545e-01.
    n:  1, spvals:  0.02 [s], spinterp:  0.01 [s], e_max: 6.493e-01.
    n:  2, spvals:  0.02 [s], spinterp:  0.02 [s], e_max: 6.296e-01.
    n:  3, spvals:  0.02 [s], spinterp:  0.05 [s], e_max: 5.834e-01.
    n:  4, spvals:  0.02 [s], spinterp:  0.09 [s], e_max: 4.927e-01.
    n:  5, spvals:  0.03 [s], spinterp:  0.17 [s], e_max: 3.837e-01.
    n:  6, spvals:  0.04 [s], spinterp:  0.28 [s], e_max: 2.509e-01.
    n:  7, spvals:  0.07 [s], spinterp:  0.43 [s], e_max: 1.723e-01.
    n:  8, spvals:  0.15 [s], spinterp:  0.62 [s], e_max: 9.562e-02.
    n:  9, spvals:  0.38 [s], spinterp:  0.87 [s], e_max: 6.174e-02.
    n: 10, spvals:  1.08 [s], spinterp:  1.17 [s], e_max: 3.088e-02.
    n: 11, spvals:  3.05 [s], spinterp:  1.53 [s], e_max: 1.385e-02.
 
Current test function: 6
  Grid: Maximum
    n:  0, spvals:  0.01 [s], spinterp:  0.04 [s], e_max: 4.241e+00.
    n:  1, spvals:  0.02 [s], spinterp:  0.11 [s], e_max: 4.004e+00.
    n:  2, spvals:  0.02 [s], spinterp:  0.19 [s], e_max: 4.190e+00.
    n:  3, spvals:  0.05 [s], spinterp:  0.32 [s], e_max: 3.731e+00.
    n:  4, spvals:  0.11 [s], spinterp:  0.50 [s], e_max: 2.973e+00.
    n:  5, spvals:  0.31 [s], spinterp:  0.73 [s], e_max: 2.000e+00.
    n:  6, spvals:  0.91 [s], spinterp:  1.02 [s], e_max: 2.135e+00.
    n:  7, spvals:  2.63 [s], spinterp:  1.38 [s], e_max: 1.618e+00.
 
  Grid: NoBoundary
    n:  0, spvals:  0.01 [s], spinterp:  0.00 [s], e_max: 4.517e+00.
    n:  1, spvals:  0.01 [s], spinterp:  0.01 [s], e_max: 4.517e+00.
    n:  2, spvals:  0.01 [s], spinterp:  0.02 [s], e_max: 4.517e+00.
    n:  3, spvals:  0.02 [s], spinterp:  0.04 [s], e_max: 7.205e+00.
    n:  4, spvals:  0.03 [s], spinterp:  0.08 [s], e_max: 4.317e+00.
    n:  5, spvals:  0.04 [s], spinterp:  0.14 [s], e_max: 2.433e+00.
    n:  6, spvals:  0.10 [s], spinterp:  0.22 [s], e_max: 2.562e+00.
    n:  7, spvals:  0.27 [s], spinterp:  0.32 [s], e_max: 2.209e+00.
    n:  8, spvals:  0.83 [s], spinterp:  0.46 [s], e_max: 2.407e+00.
    n:  9, spvals:  2.58 [s], spinterp:  0.63 [s], e_max: 1.457e+00.
 
  Grid: Clenshaw-Curtis
    n:  0, spvals:  0.01 [s], spinterp:  0.00 [s], e_max: 4.517e+00.
    n:  1, spvals:  0.01 [s], spinterp:  0.01 [s], e_max: 4.517e+00.
    n:  2, spvals:  0.02 [s], spinterp:  0.02 [s], e_max: 4.307e+00.
    n:  3, spvals:  0.02 [s], spinterp:  0.05 [s], e_max: 4.060e+00.
    n:  4, spvals:  0.02 [s], spinterp:  0.10 [s], e_max: 4.041e+00.
    n:  5, spvals:  0.03 [s], spinterp:  0.17 [s], e_max: 3.857e+00.
    n:  6, spvals:  0.04 [s], spinterp:  0.29 [s], e_max: 3.041e+00.
    n:  7, spvals:  0.07 [s], spinterp:  0.43 [s], e_max: 2.046e+00.
    n:  8, spvals:  0.15 [s], spinterp:  0.62 [s], e_max: 2.175e+00.
    n:  9, spvals:  0.38 [s], spinterp:  0.87 [s], e_max: 1.643e+00.
    n: 10, spvals:  1.08 [s], spinterp:  1.17 [s], e_max: 2.324e+00.
    n: 11, spvals:  3.04 [s], spinterp:  1.54 [s], e_max: 1.471e+00.
```

## spcomparepoly

```
rand('seed',0); spcomparepoly
```

```
Problem dimension: d = 3, max. nodes: N = 6017
Current test function: 1
  Grid: Chebyshev
    n:  0, spvals:  0.01 [s], spinterp:  0.00 [s], e_max: 5.018e-01.
    n:  1, spvals:  0.02 [s], spinterp:  0.00 [s], e_max: 6.306e-02.
    n:  2, spvals:  0.02 [s], spinterp:  0.01 [s], e_max: 6.553e-03.
    n:  3, spvals:  0.03 [s], spinterp:  0.03 [s], e_max: 7.842e-05.
    n:  4, spvals:  0.05 [s], spinterp:  0.05 [s], e_max: 3.848e-07.
    n:  5, spvals:  0.09 [s], spinterp:  0.10 [s], e_max: 1.071e-09.
    n:  6, spvals:  0.18 [s], spinterp:  0.16 [s], e_max: 1.447e-12.
    n:  7, spvals:  0.32 [s], spinterp:  0.25 [s], e_max: 1.665e-15.
    n:  8, spvals:  0.56 [s], spinterp:  0.38 [s], e_max: 2.554e-15.
 
  Grid: Gauss-Patterson
    n:  0, spvals:  0.01 [s], spinterp:  0.00 [s], e_max: 5.018e-01.
    n:  1, spvals:  0.02 [s], spinterp:  0.00 [s], e_max: 6.478e-02.
    n:  2, spvals:  0.02 [s], spinterp:  0.01 [s], e_max: 6.630e-03.
    n:  3, spvals:  0.03 [s], spinterp:  0.03 [s], e_max: 4.256e-05.
    n:  4, spvals:  0.07 [s], spinterp:  0.07 [s], e_max: 8.490e-08.
    n:  5, spvals:  0.17 [s], spinterp:  0.13 [s], e_max: 1.658e-10.
 
  Grid: Clenshaw-Curtis
    n:  0, spvals:  0.01 [s], spinterp:  0.00 [s], e_max: 5.018e-01.
    n:  1, spvals:  0.01 [s], spinterp:  0.00 [s], e_max: 5.379e-02.
    n:  2, spvals:  0.02 [s], spinterp:  0.00 [s], e_max: 1.068e-02.
    n:  3, spvals:  0.02 [s], spinterp:  0.01 [s], e_max: 1.692e-03.
    n:  4, spvals:  0.02 [s], spinterp:  0.01 [s], e_max: 4.618e-04.
    n:  5, spvals:  0.03 [s], spinterp:  0.02 [s], e_max: 1.258e-04.
    n:  6, spvals:  0.04 [s], spinterp:  0.03 [s], e_max: 3.032e-05.
    n:  7, spvals:  0.07 [s], spinterp:  0.05 [s], e_max: 8.001e-06.
    n:  8, spvals:  0.15 [s], spinterp:  0.07 [s], e_max: 2.362e-06.
 
Current test function: 2
  Grid: Chebyshev
    n:  0, spvals:  0.01 [s], spinterp:  0.00 [s], e_max: 1.001e-01.
    n:  1, spvals:  0.02 [s], spinterp:  0.00 [s], e_max: 1.481e-02.
    n:  2, spvals:  0.02 [s], spinterp:  0.01 [s], e_max: 4.769e-03.
    n:  3, spvals:  0.03 [s], spinterp:  0.03 [s], e_max: 6.415e-04.
    n:  4, spvals:  0.05 [s], spinterp:  0.05 [s], e_max: 8.104e-05.
    n:  5, spvals:  0.09 [s], spinterp:  0.10 [s], e_max: 4.739e-06.
    n:  6, spvals:  0.18 [s], spinterp:  0.16 [s], e_max: 1.056e-07.
    n:  7, spvals:  0.32 [s], spinterp:  0.25 [s], e_max: 1.663e-09.
    n:  8, spvals:  0.57 [s], spinterp:  0.39 [s], e_max: 1.197e-11.
 
  Grid: Gauss-Patterson
    n:  0, spvals:  0.01 [s], spinterp:  0.00 [s], e_max: 1.001e-01.
    n:  1, spvals:  0.02 [s], spinterp:  0.00 [s], e_max: 3.321e-02.
    n:  2, spvals:  0.02 [s], spinterp:  0.01 [s], e_max: 4.254e-03.
    n:  3, spvals:  0.03 [s], spinterp:  0.03 [s], e_max: 2.876e-04.
    n:  4, spvals:  0.07 [s], spinterp:  0.07 [s], e_max: 5.701e-06.
    n:  5, spvals:  0.18 [s], spinterp:  0.13 [s], e_max: 4.869e-08.
 
  Grid: Clenshaw-Curtis
    n:  0, spvals:  0.01 [s], spinterp:  0.00 [s], e_max: 1.001e-01.
    n:  1, spvals:  0.02 [s], spinterp:  0.00 [s], e_max: 2.462e-02.
    n:  2, spvals:  0.02 [s], spinterp:  0.00 [s], e_max: 9.673e-03.
    n:  3, spvals:  0.02 [s], spinterp:  0.01 [s], e_max: 3.259e-03.
    n:  4, spvals:  0.02 [s], spinterp:  0.01 [s], e_max: 1.118e-03.
    n:  5, spvals:  0.03 [s], spinterp:  0.02 [s], e_max: 3.170e-04.
    n:  6, spvals:  0.04 [s], spinterp:  0.03 [s], e_max: 9.598e-05.
    n:  7, spvals:  0.07 [s], spinterp:  0.05 [s], e_max: 3.164e-05.
    n:  8, spvals:  0.15 [s], spinterp:  0.07 [s], e_max: 8.116e-06.
 
Current test function: 3
  Grid: Chebyshev
    n:  0, spvals:  0.01 [s], spinterp:  0.00 [s], e_max: 3.030e-01.
    n:  1, spvals:  0.02 [s], spinterp:  0.00 [s], e_max: 1.598e-01.
    n:  2, spvals:  0.02 [s], spinterp:  0.01 [s], e_max: 3.826e-02.
    n:  3, spvals:  0.03 [s], spinterp:  0.03 [s], e_max: 2.763e-02.
    n:  4, spvals:  0.05 [s], spinterp:  0.06 [s], e_max: 6.861e-03.
    n:  5, spvals:  0.10 [s], spinterp:  0.10 [s], e_max: 6.918e-04.
    n:  6, spvals:  0.18 [s], spinterp:  0.16 [s], e_max: 7.857e-05.
    n:  7, spvals:  0.32 [s], spinterp:  0.25 [s], e_max: 8.324e-06.
    n:  8, spvals:  0.56 [s], spinterp:  0.39 [s], e_max: 3.878e-07.
 
  Grid: Gauss-Patterson
    n:  0, spvals:  0.01 [s], spinterp:  0.00 [s], e_max: 3.030e-01.
    n:  1, spvals:  0.02 [s], spinterp:  0.00 [s], e_max: 1.669e-01.
    n:  2, spvals:  0.02 [s], spinterp:  0.01 [s], e_max: 4.473e-02.
    n:  3, spvals:  0.03 [s], spinterp:  0.03 [s], e_max: 4.740e-03.
    n:  4, spvals:  0.07 [s], spinterp:  0.07 [s], e_max: 3.462e-04.
    n:  5, spvals:  0.17 [s], spinterp:  0.13 [s], e_max: 4.559e-05.
 
  Grid: Clenshaw-Curtis
    n:  0, spvals:  0.01 [s], spinterp:  0.00 [s], e_max: 3.030e-01.
    n:  1, spvals:  0.02 [s], spinterp:  0.00 [s], e_max: 1.472e-01.
    n:  2, spvals:  0.02 [s], spinterp:  0.00 [s], e_max: 6.178e-02.
    n:  3, spvals:  0.02 [s], spinterp:  0.01 [s], e_max: 7.891e-02.
    n:  4, spvals:  0.02 [s], spinterp:  0.01 [s], e_max: 6.245e-03.
    n:  5, spvals:  0.03 [s], spinterp:  0.02 [s], e_max: 5.753e-03.
    n:  6, spvals:  0.04 [s], spinterp:  0.03 [s], e_max: 1.423e-03.
    n:  7, spvals:  0.07 [s], spinterp:  0.05 [s], e_max: 4.932e-04.
    n:  8, spvals:  0.15 [s], spinterp:  0.07 [s], e_max: 5.652e-05.
 
Current test function: 4
  Grid: Chebyshev
    n:  0, spvals:  0.01 [s], spinterp:  0.00 [s], e_max: 5.056e-01.
    n:  1, spvals:  0.02 [s], spinterp:  0.00 [s], e_max: 6.951e-01.
    n:  2, spvals:  0.02 [s], spinterp:  0.01 [s], e_max: 4.160e-01.
    n:  3, spvals:  0.03 [s], spinterp:  0.03 [s], e_max: 1.551e-01.
    n:  4, spvals:  0.05 [s], spinterp:  0.05 [s], e_max: 3.637e-02.
    n:  5, spvals:  0.10 [s], spinterp:  0.10 [s], e_max: 4.841e-03.
    n:  6, spvals:  0.18 [s], spinterp:  0.16 [s], e_max: 2.100e-04.
    n:  7, spvals:  0.32 [s], spinterp:  0.25 [s], e_max: 6.429e-06.
    n:  8, spvals:  0.56 [s], spinterp:  0.38 [s], e_max: 3.622e-08.
 
  Grid: Gauss-Patterson
    n:  0, spvals:  0.01 [s], spinterp:  0.00 [s], e_max: 5.056e-01.
    n:  1, spvals:  0.02 [s], spinterp:  0.00 [s], e_max: 6.951e-01.
    n:  2, spvals:  0.02 [s], spinterp:  0.01 [s], e_max: 2.533e-01.
    n:  3, spvals:  0.04 [s], spinterp:  0.03 [s], e_max: 7.480e-02.
    n:  4, spvals:  0.07 [s], spinterp:  0.07 [s], e_max: 8.683e-03.
    n:  5, spvals:  0.17 [s], spinterp:  0.13 [s], e_max: 1.059e-04.
 
  Grid: Clenshaw-Curtis
    n:  0, spvals:  0.01 [s], spinterp:  0.00 [s], e_max: 5.056e-01.
    n:  1, spvals:  0.02 [s], spinterp:  0.00 [s], e_max: 5.730e-01.
    n:  2, spvals:  0.02 [s], spinterp:  0.00 [s], e_max: 1.980e-01.
    n:  3, spvals:  0.02 [s], spinterp:  0.01 [s], e_max: 1.000e-01.
    n:  4, spvals:  0.02 [s], spinterp:  0.01 [s], e_max: 4.882e-02.
    n:  5, spvals:  0.03 [s], spinterp:  0.02 [s], e_max: 1.528e-02.
    n:  6, spvals:  0.04 [s], spinterp:  0.03 [s], e_max: 5.724e-03.
    n:  7, spvals:  0.06 [s], spinterp:  0.05 [s], e_max: 1.887e-03.
    n:  8, spvals:  0.15 [s], spinterp:  0.07 [s], e_max: 6.093e-04.
 
Current test function: 5
  Grid: Chebyshev
    n:  0, spvals:  0.01 [s], spinterp:  0.00 [s], e_max: 5.329e-01.
    n:  1, spvals:  0.01 [s], spinterp:  0.01 [s], e_max: 5.283e-01.
    n:  2, spvals:  0.02 [s], spinterp:  0.01 [s], e_max: 5.014e-01.
    n:  3, spvals:  0.03 [s], spinterp:  0.03 [s], e_max: 3.957e-01.
    n:  4, spvals:  0.05 [s], spinterp:  0.05 [s], e_max: 1.900e-01.
    n:  5, spvals:  0.10 [s], spinterp:  0.10 [s], e_max: 1.921e-01.
    n:  6, spvals:  0.18 [s], spinterp:  0.16 [s], e_max: 9.081e-02.
    n:  7, spvals:  0.32 [s], spinterp:  0.25 [s], e_max: 2.433e-02.
    n:  8, spvals:  0.56 [s], spinterp:  0.38 [s], e_max: 1.296e-02.
 
  Grid: Gauss-Patterson
    n:  0, spvals:  0.01 [s], spinterp:  0.00 [s], e_max: 5.329e-01.
    n:  1, spvals:  0.02 [s], spinterp:  0.00 [s], e_max: 5.217e-01.
    n:  2, spvals:  0.02 [s], spinterp:  0.01 [s], e_max: 4.437e-01.
    n:  3, spvals:  0.03 [s], spinterp:  0.03 [s], e_max: 2.577e-01.
    n:  4, spvals:  0.07 [s], spinterp:  0.07 [s], e_max: 1.548e-01.
    n:  5, spvals:  0.17 [s], spinterp:  0.13 [s], e_max: 6.078e-02.
 
  Grid: Clenshaw-Curtis
    n:  0, spvals:  0.01 [s], spinterp:  0.00 [s], e_max: 5.329e-01.
    n:  1, spvals:  0.02 [s], spinterp:  0.00 [s], e_max: 5.279e-01.
    n:  2, spvals:  0.02 [s], spinterp:  0.00 [s], e_max: 5.087e-01.
    n:  3, spvals:  0.02 [s], spinterp:  0.01 [s], e_max: 4.627e-01.
    n:  4, spvals:  0.02 [s], spinterp:  0.01 [s], e_max: 3.814e-01.
    n:  5, spvals:  0.03 [s], spinterp:  0.02 [s], e_max: 2.771e-01.
    n:  6, spvals:  0.04 [s], spinterp:  0.03 [s], e_max: 1.698e-01.
    n:  7, spvals:  0.07 [s], spinterp:  0.05 [s], e_max: 9.262e-02.
    n:  8, spvals:  0.15 [s], spinterp:  0.07 [s], e_max: 4.581e-02.
 
Current test function: 6
  Grid: Chebyshev
    n:  0, spvals:  0.01 [s], spinterp:  0.00 [s], e_max: 3.553e+00.
    n:  1, spvals:  0.01 [s], spinterp:  0.00 [s], e_max: 3.553e+00.
    n:  2, spvals:  0.02 [s], spinterp:  0.01 [s], e_max: 3.189e+00.
    n:  3, spvals:  0.03 [s], spinterp:  0.03 [s], e_max: 2.090e+00.
    n:  4, spvals:  0.05 [s], spinterp:  0.05 [s], e_max: 1.852e+00.
    n:  5, spvals:  0.10 [s], spinterp:  0.10 [s], e_max: 2.008e+00.
    n:  6, spvals:  0.18 [s], spinterp:  0.16 [s], e_max: 2.434e+00.
    n:  7, spvals:  0.32 [s], spinterp:  0.25 [s], e_max: 1.907e+00.
    n:  8, spvals:  0.56 [s], spinterp:  0.39 [s], e_max: 1.060e+00.
 
  Grid: Gauss-Patterson
    n:  0, spvals:  0.01 [s], spinterp:  0.00 [s], e_max: 3.553e+00.
    n:  1, spvals:  0.01 [s], spinterp:  0.00 [s], e_max: 3.553e+00.
    n:  2, spvals:  0.02 [s], spinterp:  0.01 [s], e_max: 2.422e+00.
    n:  3, spvals:  0.03 [s], spinterp:  0.03 [s], e_max: 2.798e+00.
    n:  4, spvals:  0.07 [s], spinterp:  0.07 [s], e_max: 2.469e+00.
    n:  5, spvals:  0.18 [s], spinterp:  0.13 [s], e_max: 1.475e+00.
 
  Grid: Clenshaw-Curtis
    n:  0, spvals:  0.01 [s], spinterp:  0.00 [s], e_max: 3.553e+00.
    n:  1, spvals:  0.02 [s], spinterp:  0.00 [s], e_max: 3.553e+00.
    n:  2, spvals:  0.02 [s], spinterp:  0.00 [s], e_max: 3.007e+00.
    n:  3, spvals:  0.02 [s], spinterp:  0.01 [s], e_max: 3.098e+00.
    n:  4, spvals:  0.02 [s], spinterp:  0.01 [s], e_max: 3.326e+00.
    n:  5, spvals:  0.03 [s], spinterp:  0.02 [s], e_max: 3.114e+00.
    n:  6, spvals:  0.04 [s], spinterp:  0.03 [s], e_max: 2.565e+00.
    n:  7, spvals:  0.07 [s], spinterp:  0.05 [s], e_max: 1.468e+00.
    n:  8, spvals:  0.15 [s], spinterp:  0.07 [s], e_max: 4.985e-01.
```

## spcomparederiv

```
rand('seed',0); spcomparederiv
```

```
Problem dimension: d = 3, max. nodes: N = 6017
Current test function: 1
 Grid: Chebyshev
    n:  0, spvals:  0.01 [s], spinterp:  0.00 [s], e_max: 6.033e-01.
    n:  1, spvals:  0.02 [s], spinterp:  0.01 [s], e_max: 1.937e-01.
    n:  2, spvals:  0.02 [s], spinterp:  0.03 [s], e_max: 1.975e-02.
    n:  3, spvals:  0.03 [s], spinterp:  0.08 [s], e_max: 3.528e-04.
    n:  4, spvals:  0.05 [s], spinterp:  0.20 [s], e_max: 2.143e-06.
    n:  5, spvals:  0.10 [s], spinterp:  0.42 [s], e_max: 7.110e-09.
    n:  6, spvals:  0.18 [s], spinterp:  0.87 [s], e_max: 1.277e-11.
    n:  7, spvals:  0.32 [s], spinterp:  1.73 [s], e_max: 7.425e-13.
    n:  8, spvals:  0.56 [s], spinterp:  3.44 [s], e_max: 2.887e-12.
 
 Grid: Clenshaw-Curtis
    n:  0, spvals:  0.01 [s], spinterp:  0.00 [s], e_max: 6.033e-01.
    n:  1, spvals:  0.02 [s], spinterp:  0.00 [s], e_max: 1.831e-01.
    n:  2, spvals:  0.02 [s], spinterp:  0.01 [s], e_max: 4.766e-02.
    n:  3, spvals:  0.02 [s], spinterp:  0.01 [s], e_max: 2.084e-02.
    n:  4, spvals:  0.02 [s], spinterp:  0.02 [s], e_max: 1.066e-02.
    n:  5, spvals:  0.03 [s], spinterp:  0.03 [s], e_max: 6.509e-03.
    n:  6, spvals:  0.04 [s], spinterp:  0.04 [s], e_max: 2.766e-03.
    n:  7, spvals:  0.07 [s], spinterp:  0.06 [s], e_max: 1.317e-03.
    n:  8, spvals:  0.15 [s], spinterp:  0.08 [s], e_max: 6.536e-04.
 
 Grid: Clenshaw-Curtis
   Enabling augmented continuous derivatives.
    n:  0, spvals:  0.01 [s], spinterp:  0.00 [s], e_max: 6.033e-01.
    n:  1, spvals:  0.01 [s], spinterp:  0.01 [s], e_max: 1.937e-01.
    n:  2, spvals:  0.02 [s], spinterp:  0.01 [s], e_max: 2.320e-02.
    n:  3, spvals:  0.02 [s], spinterp:  0.02 [s], e_max: 8.242e-03.
    n:  4, spvals:  0.02 [s], spinterp:  0.03 [s], e_max: 3.464e-03.
    n:  5, spvals:  0.03 [s], spinterp:  0.05 [s], e_max: 1.449e-03.
    n:  6, spvals:  0.04 [s], spinterp:  0.08 [s], e_max: 7.253e-04.
    n:  7, spvals:  0.07 [s], spinterp:  0.11 [s], e_max: 3.888e-04.
    n:  8, spvals:  0.15 [s], spinterp:  0.16 [s], e_max: 1.781e-04.
 
Current test function: 2
 Grid: Chebyshev
    n:  0, spvals:  0.01 [s], spinterp:  0.00 [s], e_max: 2.023e-01.
    n:  1, spvals:  0.02 [s], spinterp:  0.01 [s], e_max: 1.398e-01.
    n:  2, spvals:  0.02 [s], spinterp:  0.03 [s], e_max: 3.281e-02.
    n:  3, spvals:  0.03 [s], spinterp:  0.09 [s], e_max: 6.150e-03.
    n:  4, spvals:  0.05 [s], spinterp:  0.20 [s], e_max: 5.827e-04.
    n:  5, spvals:  0.10 [s], spinterp:  0.43 [s], e_max: 8.254e-05.
    n:  6, spvals:  0.18 [s], spinterp:  0.87 [s], e_max: 2.459e-06.
    n:  7, spvals:  0.32 [s], spinterp:  1.73 [s], e_max: 3.644e-08.
    n:  8, spvals:  0.58 [s], spinterp:  3.43 [s], e_max: 2.265e-10.
 
 Grid: Clenshaw-Curtis
    n:  0, spvals:  0.01 [s], spinterp:  0.00 [s], e_max: 2.023e-01.
    n:  1, spvals:  0.02 [s], spinterp:  0.00 [s], e_max: 1.468e-01.
    n:  2, spvals:  0.02 [s], spinterp:  0.01 [s], e_max: 9.324e-02.
    n:  3, spvals:  0.02 [s], spinterp:  0.01 [s], e_max: 5.634e-02.
    n:  4, spvals:  0.02 [s], spinterp:  0.02 [s], e_max: 3.036e-02.
    n:  5, spvals:  0.03 [s], spinterp:  0.03 [s], e_max: 1.366e-02.
    n:  6, spvals:  0.04 [s], spinterp:  0.04 [s], e_max: 6.607e-03.
    n:  7, spvals:  0.07 [s], spinterp:  0.06 [s], e_max: 3.579e-03.
    n:  8, spvals:  0.15 [s], spinterp:  0.08 [s], e_max: 1.884e-03.
 
 Grid: Clenshaw-Curtis
   Enabling augmented continuous derivatives.
    n:  0, spvals:  0.01 [s], spinterp:  0.00 [s], e_max: 2.023e-01.
    n:  1, spvals:  0.01 [s], spinterp:  0.01 [s], e_max: 1.497e-01.
    n:  2, spvals:  0.02 [s], spinterp:  0.01 [s], e_max: 5.882e-02.
    n:  3, spvals:  0.02 [s], spinterp:  0.02 [s], e_max: 3.761e-02.
    n:  4, spvals:  0.02 [s], spinterp:  0.03 [s], e_max: 2.644e-02.
    n:  5, spvals:  0.03 [s], spinterp:  0.05 [s], e_max: 4.679e-03.
    n:  6, spvals:  0.04 [s], spinterp:  0.08 [s], e_max: 2.191e-03.
    n:  7, spvals:  0.06 [s], spinterp:  0.11 [s], e_max: 1.074e-03.
    n:  8, spvals:  0.15 [s], spinterp:  0.16 [s], e_max: 4.386e-04.
 
Current test function: 3
 Grid: Chebyshev
    n:  0, spvals:  0.01 [s], spinterp:  0.00 [s], e_max: 8.350e-01.
    n:  1, spvals:  0.02 [s], spinterp:  0.01 [s], e_max: 6.276e-01.
    n:  2, spvals:  0.02 [s], spinterp:  0.03 [s], e_max: 2.228e-01.
    n:  3, spvals:  0.03 [s], spinterp:  0.08 [s], e_max: 1.175e-01.
    n:  4, spvals:  0.05 [s], spinterp:  0.20 [s], e_max: 3.137e-02.
    n:  5, spvals:  0.10 [s], spinterp:  0.43 [s], e_max: 5.188e-03.
    n:  6, spvals:  0.18 [s], spinterp:  0.87 [s], e_max: 7.634e-04.
    n:  7, spvals:  0.32 [s], spinterp:  1.73 [s], e_max: 5.840e-05.
    n:  8, spvals:  0.58 [s], spinterp:  3.44 [s], e_max: 2.573e-06.
 
 Grid: Clenshaw-Curtis
    n:  0, spvals:  0.01 [s], spinterp:  0.00 [s], e_max: 8.350e-01.
    n:  1, spvals:  0.02 [s], spinterp:  0.00 [s], e_max: 6.517e-01.
    n:  2, spvals:  0.02 [s], spinterp:  0.01 [s], e_max: 2.296e-01.
    n:  3, spvals:  0.02 [s], spinterp:  0.01 [s], e_max: 3.827e-01.
    n:  4, spvals:  0.02 [s], spinterp:  0.02 [s], e_max: 2.122e-01.
    n:  5, spvals:  0.03 [s], spinterp:  0.03 [s], e_max: 7.974e-02.
    n:  6, spvals:  0.04 [s], spinterp:  0.04 [s], e_max: 3.369e-02.
    n:  7, spvals:  0.07 [s], spinterp:  0.06 [s], e_max: 1.317e-02.
    n:  8, spvals:  0.15 [s], spinterp:  0.08 [s], e_max: 1.128e-02.
 
 Grid: Clenshaw-Curtis
   Enabling augmented continuous derivatives.
    n:  0, spvals:  0.01 [s], spinterp:  0.00 [s], e_max: 8.350e-01.
    n:  1, spvals:  0.01 [s], spinterp:  0.01 [s], e_max: 6.276e-01.
    n:  2, spvals:  0.02 [s], spinterp:  0.01 [s], e_max: 2.358e-01.
    n:  3, spvals:  0.02 [s], spinterp:  0.02 [s], e_max: 3.168e-01.
    n:  4, spvals:  0.02 [s], spinterp:  0.03 [s], e_max: 2.136e-01.
    n:  5, spvals:  0.03 [s], spinterp:  0.05 [s], e_max: 8.036e-02.
    n:  6, spvals:  0.04 [s], spinterp:  0.08 [s], e_max: 3.867e-02.
    n:  7, spvals:  0.07 [s], spinterp:  0.11 [s], e_max: 1.284e-02.
    n:  8, spvals:  0.16 [s], spinterp:  0.16 [s], e_max: 6.540e-03.
 
Current test function: 4
 Grid: Chebyshev
    n:  0, spvals:  0.01 [s], spinterp:  0.00 [s], e_max: 4.228e+00.
    n:  1, spvals:  0.02 [s], spinterp:  0.01 [s], e_max: 3.778e+00.
    n:  2, spvals:  0.02 [s], spinterp:  0.03 [s], e_max: 2.721e+00.
    n:  3, spvals:  0.03 [s], spinterp:  0.08 [s], e_max: 1.244e+00.
    n:  4, spvals:  0.05 [s], spinterp:  0.20 [s], e_max: 4.510e-01.
    n:  5, spvals:  0.10 [s], spinterp:  0.43 [s], e_max: 7.297e-02.
    n:  6, spvals:  0.18 [s], spinterp:  0.87 [s], e_max: 4.426e-03.
    n:  7, spvals:  0.32 [s], spinterp:  1.74 [s], e_max: 6.474e-05.
    n:  8, spvals:  0.56 [s], spinterp:  3.44 [s], e_max: 4.425e-07.
 
 Grid: Clenshaw-Curtis
    n:  0, spvals:  0.01 [s], spinterp:  0.00 [s], e_max: 4.228e+00.
    n:  1, spvals:  0.02 [s], spinterp:  0.00 [s], e_max: 3.218e+00.
    n:  2, spvals:  0.02 [s], spinterp:  0.01 [s], e_max: 2.696e+00.
    n:  3, spvals:  0.02 [s], spinterp:  0.01 [s], e_max: 2.328e+00.
    n:  4, spvals:  0.02 [s], spinterp:  0.02 [s], e_max: 1.301e+00.
    n:  5, spvals:  0.03 [s], spinterp:  0.03 [s], e_max: 4.936e-01.
    n:  6, spvals:  0.04 [s], spinterp:  0.04 [s], e_max: 2.811e-01.
    n:  7, spvals:  0.07 [s], spinterp:  0.06 [s], e_max: 1.428e-01.
    n:  8, spvals:  0.15 [s], spinterp:  0.08 [s], e_max: 5.465e-02.
 
 Grid: Clenshaw-Curtis
   Enabling augmented continuous derivatives.
    n:  0, spvals:  0.01 [s], spinterp:  0.00 [s], e_max: 4.228e+00.
    n:  1, spvals:  0.01 [s], spinterp:  0.01 [s], e_max: 3.779e+00.
    n:  2, spvals:  0.02 [s], spinterp:  0.01 [s], e_max: 2.352e+00.
    n:  3, spvals:  0.02 [s], spinterp:  0.02 [s], e_max: 1.224e+00.
    n:  4, spvals:  0.02 [s], spinterp:  0.03 [s], e_max: 1.079e+00.
    n:  5, spvals:  0.03 [s], spinterp:  0.05 [s], e_max: 4.725e-01.
    n:  6, spvals:  0.04 [s], spinterp:  0.08 [s], e_max: 1.477e-01.
    n:  7, spvals:  0.07 [s], spinterp:  0.11 [s], e_max: 9.491e-02.
    n:  8, spvals:  0.15 [s], spinterp:  0.16 [s], e_max: 4.890e-02.
 
Current test function: 5
 Grid: Chebyshev
    n:  0, spvals:  0.01 [s], spinterp:  0.00 [s], e_max: 4.793e+00.
    n:  1, spvals:  0.02 [s], spinterp:  0.01 [s], e_max: 4.787e+00.
    n:  2, spvals:  0.02 [s], spinterp:  0.03 [s], e_max: 4.741e+00.
    n:  3, spvals:  0.03 [s], spinterp:  0.08 [s], e_max: 4.559e+00.
    n:  4, spvals:  0.05 [s], spinterp:  0.20 [s], e_max: 3.894e+00.
    n:  5, spvals:  0.10 [s], spinterp:  0.42 [s], e_max: 3.690e+00.
    n:  6, spvals:  0.18 [s], spinterp:  0.87 [s], e_max: 3.379e+00.
    n:  7, spvals:  0.32 [s], spinterp:  1.74 [s], e_max: 2.974e+00.
    n:  8, spvals:  0.56 [s], spinterp:  3.47 [s], e_max: 2.135e+00.
 
 Grid: Clenshaw-Curtis
    n:  0, spvals:  0.01 [s], spinterp:  0.00 [s], e_max: 4.793e+00.
    n:  1, spvals:  0.02 [s], spinterp:  0.00 [s], e_max: 4.788e+00.
    n:  2, spvals:  0.02 [s], spinterp:  0.01 [s], e_max: 4.763e+00.
    n:  3, spvals:  0.02 [s], spinterp:  0.01 [s], e_max: 4.728e+00.
    n:  4, spvals:  0.02 [s], spinterp:  0.02 [s], e_max: 4.426e+00.
    n:  5, spvals:  0.03 [s], spinterp:  0.03 [s], e_max: 4.232e+00.
    n:  6, spvals:  0.04 [s], spinterp:  0.04 [s], e_max: 4.424e+00.
    n:  7, spvals:  0.07 [s], spinterp:  0.06 [s], e_max: 4.212e+00.
    n:  8, spvals:  0.15 [s], spinterp:  0.08 [s], e_max: 3.783e+00.
 
 Grid: Clenshaw-Curtis
   Enabling augmented continuous derivatives.
    n:  0, spvals:  0.02 [s], spinterp:  0.00 [s], e_max: 4.793e+00.
    n:  1, spvals:  0.02 [s], spinterp:  0.01 [s], e_max: 4.787e+00.
    n:  2, spvals:  0.02 [s], spinterp:  0.01 [s], e_max: 4.756e+00.
    n:  3, spvals:  0.02 [s], spinterp:  0.02 [s], e_max: 4.723e+00.
    n:  4, spvals:  0.02 [s], spinterp:  0.03 [s], e_max: 4.439e+00.
    n:  5, spvals:  0.03 [s], spinterp:  0.05 [s], e_max: 4.241e+00.
    n:  6, spvals:  0.04 [s], spinterp:  0.07 [s], e_max: 4.379e+00.
    n:  7, spvals:  0.07 [s], spinterp:  0.11 [s], e_max: 4.059e+00.
    n:  8, spvals:  0.15 [s], spinterp:  0.16 [s], e_max: 3.790e+00.
 
Current test function: 6
 Grid: Chebyshev
    n:  0, spvals:  0.01 [s], spinterp:  0.00 [s], e_max: 9.443e+00.
    n:  1, spvals:  0.02 [s], spinterp:  0.01 [s], e_max: 9.443e+00.
    n:  2, spvals:  0.02 [s], spinterp:  0.03 [s], e_max: 1.131e+01.
    n:  3, spvals:  0.03 [s], spinterp:  0.08 [s], e_max: 1.522e+01.
    n:  4, spvals:  0.05 [s], spinterp:  0.20 [s], e_max: 2.324e+01.
    n:  5, spvals:  0.10 [s], spinterp:  0.43 [s], e_max: 3.695e+01.
    n:  6, spvals:  0.18 [s], spinterp:  0.88 [s], e_max: 7.164e+01.
    n:  7, spvals:  0.32 [s], spinterp:  1.74 [s], e_max: 9.425e+01.
    n:  8, spvals:  0.56 [s], spinterp:  3.45 [s], e_max: 2.005e+02.
 
 Grid: Clenshaw-Curtis
    n:  0, spvals:  0.01 [s], spinterp:  0.00 [s], e_max: 9.443e+00.
    n:  1, spvals:  0.01 [s], spinterp:  0.00 [s], e_max: 9.443e+00.
    n:  2, spvals:  0.02 [s], spinterp:  0.01 [s], e_max: 1.153e+01.
    n:  3, spvals:  0.02 [s], spinterp:  0.01 [s], e_max: 1.515e+01.
    n:  4, spvals:  0.02 [s], spinterp:  0.02 [s], e_max: 2.432e+01.
    n:  5, spvals:  0.03 [s], spinterp:  0.03 [s], e_max: 4.580e+01.
    n:  6, spvals:  0.04 [s], spinterp:  0.04 [s], e_max: 9.028e+01.
    n:  7, spvals:  0.07 [s], spinterp:  0.06 [s], e_max: 1.800e+02.
    n:  8, spvals:  0.15 [s], spinterp:  0.08 [s], e_max: 1.009e+02.
 
 Grid: Clenshaw-Curtis
   Enabling augmented continuous derivatives.
    n:  0, spvals:  0.01 [s], spinterp:  0.00 [s], e_max: 9.443e+00.
    n:  1, spvals:  0.01 [s], spinterp:  0.01 [s], e_max: 9.443e+00.
    n:  2, spvals:  0.02 [s], spinterp:  0.01 [s], e_max: 1.153e+01.
    n:  3, spvals:  0.02 [s], spinterp:  0.02 [s], e_max: 1.347e+01.
    n:  4, spvals:  0.02 [s], spinterp:  0.03 [s], e_max: 2.022e+01.
    n:  5, spvals:  0.03 [s], spinterp:  0.05 [s], e_max: 3.789e+01.
    n:  6, spvals:  0.04 [s], spinterp:  0.07 [s], e_max: 9.028e+01.
    n:  7, spvals:  0.07 [s], spinterp:  0.11 [s], e_max: 1.716e+02.
    n:  8, spvals:  0.15 [s], spinterp:  0.16 [s], e_max: 1.009e+02.
```

## spadaptdemo

```
spadaptdemo
```

```
ans = 

               vals: {[29x1 double]}
           gridType: 'Chebyshev'
                  d: 2
              range: [2x2 double]
        estRelError: 0.0095
        estAbsError: 2.9017
         fevalRange: [2.7065 308.1291]
         minGridVal: [0.5000 0.1464]
         maxGridVal: [0 0]
            nPoints: 29
          fevalTime: 0.0116
    surplusCompTime: 0.0062
            indices: [1x1 struct]
           maxLevel: [4 2]
      activeIndices: [3x1 uint32]
     activeIndices2: [9x1 uint32]
                  E: [1x9 double]
                  G: [9x1 double]
                 G2: [9x1 double]
       maxSetPoints: 4
           dimAdapt: 1
```

## spadaptanim

```
spadaptanim
```

```
Warning: Current number of support nodes nPoints = 5: MaxPoints = 2 reached before accuracies RelTol = 1e-08 or AbsTol = 1e-06 were achieved.
The current estimated relative accuracy is 1. Increase maximum number of allowable points MaxPoints to further refine the interpolant. 

z = 

               vals: {[5x1 double]}
           gridType: 'Clenshaw-Curtis'
                  d: 2
              range: [2x2 double]
        estRelError: 1
        estAbsError: 0.9933
         fevalRange: [1.0067 2]
         minGridVal: [0 0.5000]
         maxGridVal: [0.5000 0.5000]
            nPoints: 5
          fevalTime: 0.0029
    surplusCompTime: 3.0900e-04
            indices: [1x1 struct]
           maxLevel: [1 1]
      activeIndices: [2x1 uint32]
     activeIndices2: [3x1 uint32]
                  E: [Inf 0.9933 0.9933]
                  G: [3x1 double]
                 G2: [3x1 double]
       maxSetPoints: 1
           dimAdapt: 1

Warning: Current number of support nodes nPoints = 7: MaxPoints = 6 reached before accuracies RelTol = 1e-08 or AbsTol = 1e-06 were achieved.
The current estimated relative accuracy is 1. Increase maximum number of allowable points MaxPoints to further refine the interpolant. 

z = 

               vals: {[7x1 double]}
           gridType: 'Clenshaw-Curtis'
                  d: 2
              range: [2x2 double]
        estRelError: 1
        estAbsError: 0.9933
         fevalRange: [1.0067 2]
         minGridVal: [0 0.5000]
         maxGridVal: [0.5000 0.5000]
            nPoints: 7
          fevalTime: 0.0015
    surplusCompTime: 4.4900e-04
            indices: [1x1 struct]
           maxLevel: [2 1]
      activeIndices: [2x1 uint32]
     activeIndices2: [4x1 uint32]
                  E: [Inf 0.9933 0.9933 0.2169]
                  G: [4x1 double]
                 G2: [4x1 double]
       maxSetPoints: 2
           dimAdapt: 1

Warning: Current number of support nodes nPoints = 13: MaxPoints = 8 reached before accuracies RelTol = 1e-08 or AbsTol = 1e-06 were achieved.
The current estimated relative accuracy is 0.10917. Increase maximum number of allowable points MaxPoints to further refine the interpolant. 

z = 

               vals: {[13x1 double]}
           gridType: 'Clenshaw-Curtis'
                  d: 2
              range: [2x2 double]
        estRelError: 0.1092
        estAbsError: 0.2169
         fevalRange: [0.0135 2]
         minGridVal: [0 0]
         maxGridVal: [0.5000 0.5000]
            nPoints: 13
          fevalTime: 0.0019
    surplusCompTime: 6.2600e-04
            indices: [1x1 struct]
           maxLevel: [2 2]
      activeIndices: [3x1 uint32]
     activeIndices2: [6x1 uint32]
                  E: [Inf 0.9933 0.9933 0.2169 2.0470e-16 0.2169]
                  G: [6x1 double]
                 G2: [6x1 double]
       maxSetPoints: 2
           dimAdapt: 1

Warning: Current number of support nodes nPoints = 17: MaxPoints = 14 reached before accuracies RelTol = 1e-08 or AbsTol = 1e-06 were achieved.
The current estimated relative accuracy is 0.10917. Increase maximum number of allowable points MaxPoints to further refine the interpolant. 

z = 

               vals: {[17x1 double]}
           gridType: 'Clenshaw-Curtis'
                  d: 2
              range: [2x2 double]
        estRelError: 0.1092
        estAbsError: 0.2169
         fevalRange: [0.0135 2]
         minGridVal: [0 0]
         maxGridVal: [0.5000 0.5000]
            nPoints: 17
          fevalTime: 0.0015
    surplusCompTime: 5.0200e-04
            indices: [1x1 struct]
           maxLevel: [3 2]
      activeIndices: [3x1 uint32]
     activeIndices2: [7x1 uint32]
                  E: [Inf 0.9933 0.9933 0.2169 2.0470e-16 0.2169 0.0884]
                  G: [7x1 double]
                 G2: [7x1 double]
       maxSetPoints: 3
           dimAdapt: 1

Warning: Current number of support nodes nPoints = 21: MaxPoints = 18 reached before accuracies RelTol = 1e-08 or AbsTol = 1e-06 were achieved.
The current estimated relative accuracy is 0.044481. Increase maximum number of allowable points MaxPoints to further refine the interpolant. 

z = 

               vals: {[21x1 double]}
           gridType: 'Clenshaw-Curtis'
                  d: 2
              range: [2x2 double]
        estRelError: 0.0445
        estAbsError: 0.0884
         fevalRange: [0.0135 2]
         minGridVal: [0 0]
         maxGridVal: [0.5000 0.5000]
            nPoints: 21
          fevalTime: 0.0015
    surplusCompTime: 5.0400e-04
            indices: [1x1 struct]
           maxLevel: [3 3]
      activeIndices: [3x1 uint32]
     activeIndices2: [8x1 uint32]
                  E: [1x8 double]
                  G: [8x1 double]
                 G2: [8x1 double]
       maxSetPoints: 3
           dimAdapt: 1

Warning: Current number of support nodes nPoints = 29: MaxPoints = 22 reached before accuracies RelTol = 1e-08 or AbsTol = 1e-06 were achieved.
The current estimated relative accuracy is 0.044481. Increase maximum number of allowable points MaxPoints to further refine the interpolant. 

z = 

               vals: {[29x1 double]}
           gridType: 'Clenshaw-Curtis'
                  d: 2
              range: [2x2 double]
        estRelError: 0.0445
        estAbsError: 0.0884
         fevalRange: [0.0135 2]
         minGridVal: [0 0]
         maxGridVal: [0.5000 0.5000]
            nPoints: 29
          fevalTime: 0.0015
    surplusCompTime: 5.5200e-04
            indices: [1x1 struct]
           maxLevel: [4 3]
      activeIndices: [3x1 uint32]
     activeIndices2: [9x1 uint32]
                  E: [1x9 double]
                  G: [9x1 double]
                 G2: [9x1 double]
       maxSetPoints: 4
           dimAdapt: 1

Warning: Current number of support nodes nPoints = 37: MaxPoints = 30 reached before accuracies RelTol = 1e-08 or AbsTol = 1e-06 were achieved.
The current estimated relative accuracy is 0.029721. Increase maximum number of allowable points MaxPoints to further refine the interpolant. 

z = 

               vals: {[37x1 double]}
           gridType: 'Clenshaw-Curtis'
                  d: 2
              range: [2x2 double]
        estRelError: 0.0297
        estAbsError: 0.0590
         fevalRange: [0.0135 2]
         minGridVal: [0 0]
         maxGridVal: [0.5000 0.5000]
            nPoints: 37
          fevalTime: 0.0015
    surplusCompTime: 5.5500e-04
            indices: [1x1 struct]
           maxLevel: [4 4]
      activeIndices: [3x1 uint32]
     activeIndices2: [10x1 uint32]
                  E: [1x10 double]
                  G: [10x1 double]
                 G2: [10x1 double]
       maxSetPoints: 4
           dimAdapt: 1

Warning: Current number of support nodes nPoints = 53: MaxPoints = 38 reached before accuracies RelTol = 1e-08 or AbsTol = 1e-06 were achieved.
The current estimated relative accuracy is 0.029721. Increase maximum number of allowable points MaxPoints to further refine the interpolant. 

z = 

               vals: {[53x1 double]}
           gridType: 'Clenshaw-Curtis'
                  d: 2
              range: [2x2 double]
        estRelError: 0.0297
        estAbsError: 0.0590
         fevalRange: [0.0135 2]
         minGridVal: [0 0]
         maxGridVal: [0.5000 0.5000]
            nPoints: 53
          fevalTime: 0.0015
    surplusCompTime: 6.3800e-04
            indices: [1x1 struct]
           maxLevel: [5 4]
      activeIndices: [3x1 uint32]
     activeIndices2: [11x1 uint32]
                  E: [1x11 double]
                  G: [11x1 double]
                 G2: [11x1 double]
       maxSetPoints: 5
           dimAdapt: 1

Warning: Current number of support nodes nPoints = 69: MaxPoints = 54 reached before accuracies RelTol = 1e-08 or AbsTol = 1e-06 were achieved.
The current estimated relative accuracy is 0.0091788. Increase maximum number of allowable points MaxPoints to further refine the interpolant. 

z = 

               vals: {[69x1 double]}
           gridType: 'Clenshaw-Curtis'
                  d: 2
              range: [2x2 double]
        estRelError: 0.0092
        estAbsError: 0.0182
         fevalRange: [0.0135 2]
         minGridVal: [0 0]
         maxGridVal: [0.5000 0.5000]
            nPoints: 69
          fevalTime: 0.0015
    surplusCompTime: 6.3400e-04
            indices: [1x1 struct]
           maxLevel: [5 5]
      activeIndices: [3x1 uint32]
     activeIndices2: [12x1 uint32]
                  E: [1x12 double]
                  G: [12x1 double]
                 G2: [12x1 double]
       maxSetPoints: 5
           dimAdapt: 1
```

## spadapterror

```
spadapterror
```

## timespvals

```
rand('seed',0); clf; timespvals
```

```
Current dim: 1
Current level n = 0...
Warning: MaxDepth = 0 reached before accuracies RelTol = 0.01 or AbsTol = 1e-06 were achieved.
The current estimated relative accuracy is Inf.
Computing sparse grid points and evaluating function took 0.004246 [s].
Computing hierarchical surpluses took 0 [s].
 
Current level n = 1...
Warning: MaxDepth = 1 reached before accuracies RelTol = 0.01 or AbsTol = 1e-06 were achieved.
The current estimated relative accuracy is 0.55111.
Computing sparse grid points and evaluating function took 0.008609 [s].
Computing hierarchical surpluses took 0.000284 [s].
 
Current level n = 2...
Warning: MaxDepth = 2 reached before accuracies RelTol = 0.01 or AbsTol = 1e-06 were achieved.
The current estimated relative accuracy is 0.012876.
Computing sparse grid points and evaluating function took 0.012964 [s].
Computing hierarchical surpluses took 0.000587 [s].
 
Current level n = 3...
Computing sparse grid points and evaluating function took 0.017078 [s].
Computing hierarchical surpluses took 0.000817 [s].
 
Current level n = 4...
Computing sparse grid points and evaluating function took 0.021465 [s].
Computing hierarchical surpluses took 0.001066 [s].
 
Current level n = 5...
Computing sparse grid points and evaluating function took 0.025588 [s].
Computing hierarchical surpluses took 0.001353 [s].
 
Current level n = 6...
Computing sparse grid points and evaluating function took 0.029809 [s].
Computing hierarchical surpluses took 0.001737 [s].
 
Current level n = 7...
Computing sparse grid points and evaluating function took 0.033955 [s].
Computing hierarchical surpluses took 0.002348 [s].
 
Current level n = 8...
Computing sparse grid points and evaluating function took 0.03829 [s].
Computing hierarchical surpluses took 0.003489 [s].
 
Current level n = 9...
Computing sparse grid points and evaluating function took 0.042454 [s].
Computing hierarchical surpluses took 0.007144 [s].
 
Current level n = 10...
Computing sparse grid points and evaluating function took 0.046701 [s].
Computing hierarchical surpluses took 0.012579 [s].
 
Current level n = 11...
Computing sparse grid points and evaluating function took 0.051018 [s].
Computing hierarchical surpluses took 0.024573 [s].
 
Current level n = 12...
Computing sparse grid points and evaluating function took 0.05602 [s].
Computing hierarchical surpluses took 0.04929 [s].
 
Current level n = 13...
Computing sparse grid points and evaluating function took 0.060891 [s].
Computing hierarchical surpluses took 0.10274 [s].
 
Current level n = 14...
Computing sparse grid points and evaluating function took 0.067331 [s].
Computing hierarchical surpluses took 0.22245 [s].
 
Current level n = 15...
Computing sparse grid points and evaluating function took 0.075115 [s].
Computing hierarchical surpluses took 0.47911 [s].
 
Current level n = 16...
Computing sparse grid points and evaluating function took 0.088169 [s].
Computing hierarchical surpluses took 1.0343 [s].
 
Current level n = 17...
Computing sparse grid points and evaluating function took 0.11048 [s].
Computing hierarchical surpluses took 2.2157 [s].
 
Current dim: 2
Current level n = 0...
Warning: MaxDepth = 0 reached before accuracies RelTol = 0.01 or AbsTol = 1e-06 were achieved.
The current estimated relative accuracy is Inf.
Computing sparse grid points and evaluating function took 0.003936 [s].
Computing hierarchical surpluses took 0 [s].
 
Current level n = 1...
Warning: MaxDepth = 1 reached before accuracies RelTol = 0.01 or AbsTol = 1e-06 were achieved.
The current estimated relative accuracy is 0.5429.
Computing sparse grid points and evaluating function took 0.00826 [s].
Computing hierarchical surpluses took 0.000242 [s].
 
Current level n = 2...
Warning: MaxDepth = 2 reached before accuracies RelTol = 0.01 or AbsTol = 1e-06 were achieved.
The current estimated relative accuracy is 0.040298.
Computing sparse grid points and evaluating function took 0.013298 [s].
Computing hierarchical surpluses took 0.000527 [s].
 
Current level n = 3...
Computing sparse grid points and evaluating function took 0.018399 [s].
Computing hierarchical surpluses took 0.000844 [s].
 
Current level n = 4...
Computing sparse grid points and evaluating function took 0.024076 [s].
Computing hierarchical surpluses took 0.001269 [s].
 
Current level n = 5...
Computing sparse grid points and evaluating function took 0.030687 [s].
Computing hierarchical surpluses took 0.002126 [s].
 
Current level n = 6...
Computing sparse grid points and evaluating function took 0.037456 [s].
Computing hierarchical surpluses took 0.004191 [s].
 
Current level n = 7...
Computing sparse grid points and evaluating function took 0.04433 [s].
Computing hierarchical surpluses took 0.00971 [s].
 
Current level n = 8...
Computing sparse grid points and evaluating function took 0.051922 [s].
Computing hierarchical surpluses took 0.025741 [s].
 
Current level n = 9...
Computing sparse grid points and evaluating function took 0.060129 [s].
Computing hierarchical surpluses took 0.068388 [s].
 
Current level n = 10...
Computing sparse grid points and evaluating function took 0.06934 [s].
Computing hierarchical surpluses took 0.18094 [s].
 
Current level n = 11...
Computing sparse grid points and evaluating function took 0.080314 [s].
Computing hierarchical surpluses took 0.47192 [s].
 
Current level n = 12...
Computing sparse grid points and evaluating function took 0.095747 [s].
Computing hierarchical surpluses took 1.2145 [s].
 
Current level n = 13...
Computing sparse grid points and evaluating function took 0.11999 [s].
Computing hierarchical surpluses took 3.0892 [s].
 
Current dim: 4
Current level n = 0...
Warning: MaxDepth = 0 reached before accuracies RelTol = 0.01 or AbsTol = 1e-06 were achieved.
The current estimated relative accuracy is Inf.
Computing sparse grid points and evaluating function took 0.003948 [s].
Computing hierarchical surpluses took 0 [s].
 
Current level n = 1...
Warning: MaxDepth = 1 reached before accuracies RelTol = 0.01 or AbsTol = 1e-06 were achieved.
The current estimated relative accuracy is 0.53823.
Computing sparse grid points and evaluating function took 0.008771 [s].
Computing hierarchical surpluses took 0.000254 [s].
 
Current level n = 2...
Warning: MaxDepth = 2 reached before accuracies RelTol = 0.01 or AbsTol = 1e-06 were achieved.
The current estimated relative accuracy is 0.037751.
Computing sparse grid points and evaluating function took 0.015695 [s].
Computing hierarchical surpluses took 0.000573 [s].
 
Current level n = 3...
Computing sparse grid points and evaluating function took 0.027401 [s].
Computing hierarchical surpluses took 0.001317 [s].
 
Current level n = 4...
Computing sparse grid points and evaluating function took 0.046848 [s].
Computing hierarchical surpluses took 0.003853 [s].
 
Current level n = 5...
Computing sparse grid points and evaluating function took 0.077303 [s].
Computing hierarchical surpluses took 0.014127 [s].
 
Current level n = 6...
Computing sparse grid points and evaluating function took 0.12329 [s].
Computing hierarchical surpluses took 0.055509 [s].
 
Current level n = 7...
Computing sparse grid points and evaluating function took 0.19194 [s].
Computing hierarchical surpluses took 0.20568 [s].
 
Current level n = 8...
Computing sparse grid points and evaluating function took 0.29023 [s].
Computing hierarchical surpluses took 0.74409 [s].
 
Current level n = 9...
Computing sparse grid points and evaluating function took 0.443 [s].
Computing hierarchical surpluses took 2.5837 [s].
 
Current level n = 10...
Computing sparse grid points and evaluating function took 0.66088 [s].
Computing hierarchical surpluses took 8.6651 [s].
 
Current dim: 8
Current level n = 0...
Warning: MaxDepth = 0 reached before accuracies RelTol = 0.01 or AbsTol = 1e-06 were achieved.
The current estimated relative accuracy is Inf.
Computing sparse grid points and evaluating function took 0.004104 [s].
Computing hierarchical surpluses took 0 [s].
 
Current level n = 1...
Warning: MaxDepth = 1 reached before accuracies RelTol = 0.01 or AbsTol = 1e-06 were achieved.
The current estimated relative accuracy is 0.5278.
Computing sparse grid points and evaluating function took 0.009298 [s].
Computing hierarchical surpluses took 0.000251 [s].
 
Current level n = 2...
Warning: MaxDepth = 2 reached before accuracies RelTol = 0.01 or AbsTol = 1e-06 were achieved.
The current estimated relative accuracy is 0.027811.
Computing sparse grid points and evaluating function took 0.02562 [s].
Computing hierarchical surpluses took 0.000811 [s].
 
Current level n = 3...
Computing sparse grid points and evaluating function took 0.083636 [s].
Computing hierarchical surpluses took 0.004903 [s].
 
Current level n = 4...
Computing sparse grid points and evaluating function took 0.26137 [s].
Computing hierarchical surpluses took 0.041604 [s].
 
Current level n = 5...
Computing sparse grid points and evaluating function took 0.73304 [s].
Computing hierarchical surpluses took 0.307 [s].
 
Current level n = 6...
Computing sparse grid points and evaluating function took 1.8743 [s].
Computing hierarchical surpluses took 1.9925 [s].
 
Current dim: 16
Current level n = 0...
Warning: MaxDepth = 0 reached before accuracies RelTol = 0.01 or AbsTol = 1e-06 were achieved.
The current estimated relative accuracy is Inf.
Computing sparse grid points and evaluating function took 0.004287 [s].
Computing hierarchical surpluses took 0 [s].
 
Current level n = 1...
Warning: MaxDepth = 1 reached before accuracies RelTol = 0.01 or AbsTol = 1e-06 were achieved.
The current estimated relative accuracy is 0.51331.
Computing sparse grid points and evaluating function took 0.010756 [s].
Computing hierarchical surpluses took 0.000281 [s].
 
Current level n = 2...
Warning: MaxDepth = 2 reached before accuracies RelTol = 0.01 or AbsTol = 1e-06 were achieved.
The current estimated relative accuracy is 0.014598.
Computing sparse grid points and evaluating function took 0.064994 [s].
Computing hierarchical surpluses took 0.002089 [s].
 
Current level n = 3...
Computing sparse grid points and evaluating function took 0.46706 [s].
Computing hierarchical surpluses took 0.034737 [s].
 
Current level n = 4...
Computing sparse grid points and evaluating function took 2.8036 [s].
Computing hierarchical surpluses took 0.62889 [s].
```

## timespinterp

```
rand('seed',0); timespinterp
```

```
Current dim: 1
Current level n = 0...
Warning: MaxDepth = 0 reached before accuracies RelTol = 0.01 or AbsTol = 1e-06 were achieved.
The current estimated relative accuracy is Inf.
Computing sparse grid points and evaluating function took 0.004088 [s].
Computing hierarchical surpluses took 0 [s].
Performing 1000 evaluations took 0.000667 [s].
 
Current level n = 1...
Warning: MaxDepth = 1 reached before accuracies RelTol = 0.01 or AbsTol = 1e-06 were achieved.
The current estimated relative accuracy is 0.55111.
Computing sparse grid points and evaluating function took 0.008523 [s].
Computing hierarchical surpluses took 0.000286 [s].
Performing 1000 evaluations took 0.003661 [s].
 
Current level n = 2...
Warning: MaxDepth = 2 reached before accuracies RelTol = 0.01 or AbsTol = 1e-06 were achieved.
The current estimated relative accuracy is 0.012876.
Computing sparse grid points and evaluating function took 0.0127 [s].
Computing hierarchical surpluses took 0.000593 [s].
Performing 1000 evaluations took 0.004058 [s].
 
Current level n = 3...
Computing sparse grid points and evaluating function took 0.016869 [s].
Computing hierarchical surpluses took 0.000827 [s].
Performing 1000 evaluations took 0.006365 [s].
 
Current level n = 4...
Computing sparse grid points and evaluating function took 0.020991 [s].
Computing hierarchical surpluses took 0.001078 [s].
Performing 1000 evaluations took 0.008825 [s].
 
Current level n = 5...
Computing sparse grid points and evaluating function took 0.02543 [s].
Computing hierarchical surpluses took 0.001369 [s].
Performing 1000 evaluations took 0.010892 [s].
 
Current level n = 6...
Computing sparse grid points and evaluating function took 0.029582 [s].
Computing hierarchical surpluses took 0.001757 [s].
Performing 1000 evaluations took 0.013563 [s].
 
Current level n = 7...
Computing sparse grid points and evaluating function took 0.033737 [s].
Computing hierarchical surpluses took 0.00237 [s].
Performing 1000 evaluations took 0.015777 [s].
 
Current level n = 8...
Computing sparse grid points and evaluating function took 0.037924 [s].
Computing hierarchical surpluses took 0.003532 [s].
Performing 1000 evaluations took 0.018695 [s].
 
Current level n = 9...
Computing sparse grid points and evaluating function took 0.042223 [s].
Computing hierarchical surpluses took 0.005885 [s].
Performing 1000 evaluations took 0.020854 [s].
 
Current level n = 10...
Computing sparse grid points and evaluating function took 0.046725 [s].
Computing hierarchical surpluses took 0.010929 [s].
Performing 1000 evaluations took 0.024045 [s].
 
Current level n = 11...
Computing sparse grid points and evaluating function took 0.051049 [s].
Computing hierarchical surpluses took 0.0223 [s].
Performing 1000 evaluations took 0.024846 [s].
 
Current level n = 12...
Computing sparse grid points and evaluating function took 0.055802 [s].
Computing hierarchical surpluses took 0.046822 [s].
Performing 1000 evaluations took 0.027821 [s].
 
Current level n = 13...
Computing sparse grid points and evaluating function took 0.060671 [s].
Computing hierarchical surpluses took 0.10137 [s].
Performing 1000 evaluations took 0.030057 [s].
 
Current level n = 14...
Computing sparse grid points and evaluating function took 0.066442 [s].
Computing hierarchical surpluses took 0.21961 [s].
Performing 1000 evaluations took 0.032644 [s].
 
Current level n = 15...
Computing sparse grid points and evaluating function took 0.073874 [s].
Computing hierarchical surpluses took 0.47584 [s].
Performing 1000 evaluations took 0.034716 [s].
 
Current level n = 16...
Computing sparse grid points and evaluating function took 0.088178 [s].
Computing hierarchical surpluses took 1.0301 [s].
Performing 1000 evaluations took 0.037688 [s].
 
Current level n = 17...
Computing sparse grid points and evaluating function took 0.10986 [s].
Computing hierarchical surpluses took 2.2134 [s].
Performing 1000 evaluations took 0.04038 [s].
 
Done!
 
Current dim: 2
Current level n = 0...
Warning: MaxDepth = 0 reached before accuracies RelTol = 0.01 or AbsTol = 1e-06 were achieved.
The current estimated relative accuracy is Inf.
Computing sparse grid points and evaluating function took 0.004048 [s].
Computing hierarchical surpluses took 0 [s].
Performing 1000 evaluations took 0.000665 [s].
 
Current level n = 1...
Warning: MaxDepth = 1 reached before accuracies RelTol = 0.01 or AbsTol = 1e-06 were achieved.
The current estimated relative accuracy is 0.5429.
Computing sparse grid points and evaluating function took 0.008369 [s].
Computing hierarchical surpluses took 0.000238 [s].
Performing 1000 evaluations took 0.003602 [s].
 
Current level n = 2...
Warning: MaxDepth = 2 reached before accuracies RelTol = 0.01 or AbsTol = 1e-06 were achieved.
The current estimated relative accuracy is 0.040298.
Computing sparse grid points and evaluating function took 0.013158 [s].
Computing hierarchical surpluses took 0.000522 [s].
Performing 1000 evaluations took 0.010423 [s].
 
Current level n = 3...
Computing sparse grid points and evaluating function took 0.018326 [s].
Computing hierarchical surpluses took 0.000835 [s].
Performing 1000 evaluations took 0.020585 [s].
 
Current level n = 4...
Computing sparse grid points and evaluating function took 0.024004 [s].
Computing hierarchical surpluses took 0.001263 [s].
Performing 1000 evaluations took 0.036561 [s].
 
Current level n = 5...
Computing sparse grid points and evaluating function took 0.029991 [s].
Computing hierarchical surpluses took 0.002107 [s].
Performing 1000 evaluations took 0.054371 [s].
 
Current level n = 6...
Computing sparse grid points and evaluating function took 0.036798 [s].
Computing hierarchical surpluses took 0.00418 [s].
Performing 1000 evaluations took 0.077058 [s].
 
Current level n = 7...
Computing sparse grid points and evaluating function took 0.043692 [s].
Computing hierarchical surpluses took 0.010441 [s].
Performing 1000 evaluations took 0.1052 [s].
 
Current level n = 8...
Computing sparse grid points and evaluating function took 0.051164 [s].
Computing hierarchical surpluses took 0.025709 [s].
Performing 1000 evaluations took 0.13738 [s].
 
Current level n = 9...
Computing sparse grid points and evaluating function took 0.059284 [s].
Computing hierarchical surpluses took 0.07001 [s].
Performing 1000 evaluations took 0.17 [s].
 
Current level n = 10...
Computing sparse grid points and evaluating function took 0.068528 [s].
Computing hierarchical surpluses took 0.18167 [s].
Performing 1000 evaluations took 0.21156 [s].
 
Current level n = 11...
Computing sparse grid points and evaluating function took 0.08063 [s].
Computing hierarchical surpluses took 0.47154 [s].
Performing 1000 evaluations took 0.26051 [s].
 
Current level n = 12...
Computing sparse grid points and evaluating function took 0.095897 [s].
Computing hierarchical surpluses took 1.219 [s].
Performing 1000 evaluations took 0.30416 [s].
 
Current level n = 13...
Computing sparse grid points and evaluating function took 0.12085 [s].
Computing hierarchical surpluses took 3.0925 [s].
Performing 1000 evaluations took 0.35361 [s].
 
Done!
 
Current dim: 4
Current level n = 0...
Warning: MaxDepth = 0 reached before accuracies RelTol = 0.01 or AbsTol = 1e-06 were achieved.
The current estimated relative accuracy is Inf.
Computing sparse grid points and evaluating function took 0.004445 [s].
Computing hierarchical surpluses took 0 [s].
Performing 1000 evaluations took 0.000729 [s].
 
Current level n = 1...
Warning: MaxDepth = 1 reached before accuracies RelTol = 0.01 or AbsTol = 1e-06 were achieved.
The current estimated relative accuracy is 0.53823.
Computing sparse grid points and evaluating function took 0.009019 [s].
Computing hierarchical surpluses took 0.000244 [s].
Performing 1000 evaluations took 0.007043 [s].
 
Current level n = 2...
Warning: MaxDepth = 2 reached before accuracies RelTol = 0.01 or AbsTol = 1e-06 were achieved.
The current estimated relative accuracy is 0.037751.
Computing sparse grid points and evaluating function took 0.016166 [s].
Computing hierarchical surpluses took 0.000565 [s].
Performing 1000 evaluations took 0.029783 [s].
 
Current level n = 3...
Computing sparse grid points and evaluating function took 0.027756 [s].
Computing hierarchical surpluses took 0.00128 [s].
Performing 1000 evaluations took 0.089221 [s].
 
Current level n = 4...
Computing sparse grid points and evaluating function took 0.04708 [s].
Computing hierarchical surpluses took 0.003798 [s].
Performing 1000 evaluations took 0.21287 [s].
 
Current level n = 5...
Computing sparse grid points and evaluating function took 0.077304 [s].
Computing hierarchical surpluses took 0.015136 [s].
Performing 1000 evaluations took 0.43156 [s].
 
Current level n = 6...
Computing sparse grid points and evaluating function took 0.12347 [s].
Computing hierarchical surpluses took 0.056388 [s].
Performing 1000 evaluations took 0.80167 [s].
 
Current level n = 7...
Computing sparse grid points and evaluating function took 0.19107 [s].
Computing hierarchical surpluses took 0.20808 [s].
Performing 1000 evaluations took 1.3489 [s].
 
Current level n = 8...
Computing sparse grid points and evaluating function took 0.2904 [s].
Computing hierarchical surpluses took 0.74564 [s].
Performing 1000 evaluations took 2.1663 [s].
 
Current level n = 9...
Computing sparse grid points and evaluating function took 0.4363 [s].
Computing hierarchical surpluses took 2.5871 [s].
Performing 1000 evaluations took 3.3054 [s].
 
Current level n = 10...
Computing sparse grid points and evaluating function took 0.65262 [s].
Computing hierarchical surpluses took 8.6525 [s].
Performing 1000 evaluations took 4.7875 [s].
 
Done!
 
Current dim: 8
Current level n = 0...
Warning: MaxDepth = 0 reached before accuracies RelTol = 0.01 or AbsTol = 1e-06 were achieved.
The current estimated relative accuracy is Inf.
Computing sparse grid points and evaluating function took 0.004114 [s].
Computing hierarchical surpluses took 0 [s].
Performing 1000 evaluations took 0.000832 [s].
 
Current level n = 1...
Warning: MaxDepth = 1 reached before accuracies RelTol = 0.01 or AbsTol = 1e-06 were achieved.
The current estimated relative accuracy is 0.5278.
Computing sparse grid points and evaluating function took 0.009598 [s].
Computing hierarchical surpluses took 0.000251 [s].
Performing 1000 evaluations took 0.013067 [s].
 
Current level n = 2...
Warning: MaxDepth = 2 reached before accuracies RelTol = 0.01 or AbsTol = 1e-06 were achieved.
The current estimated relative accuracy is 0.027811.
Computing sparse grid points and evaluating function took 0.025728 [s].
Computing hierarchical surpluses took 0.000813 [s].
Performing 1000 evaluations took 0.10297 [s].
 
Current level n = 3...
Computing sparse grid points and evaluating function took 0.083297 [s].
Computing hierarchical surpluses took 0.005042 [s].
Performing 1000 evaluations took 0.4981 [s].
 
Current level n = 4...
Computing sparse grid points and evaluating function took 0.26234 [s].
Computing hierarchical surpluses took 0.041822 [s].
Performing 1000 evaluations took 1.81 [s].
 
Current level n = 5...
Computing sparse grid points and evaluating function took 0.74225 [s].
Computing hierarchical surpluses took 0.307 [s].
Performing 1000 evaluations took 5.4853 [s].
 
Current level n = 6...
Computing sparse grid points and evaluating function took 1.8787 [s].
Computing hierarchical surpluses took 1.9957 [s].
Performing 1000 evaluations took 14.4679 [s].
 
Done!
 
Current dim: 16
Current level n = 0...
Warning: MaxDepth = 0 reached before accuracies RelTol = 0.01 or AbsTol = 1e-06 were achieved.
The current estimated relative accuracy is Inf.
Computing sparse grid points and evaluating function took 0.004281 [s].
Computing hierarchical surpluses took 0 [s].
Performing 1000 evaluations took 0.001135 [s].
 
Current level n = 1...
Warning: MaxDepth = 1 reached before accuracies RelTol = 0.01 or AbsTol = 1e-06 were achieved.
The current estimated relative accuracy is 0.51331.
Computing sparse grid points and evaluating function took 0.010643 [s].
Computing hierarchical surpluses took 0.000271 [s].
Performing 1000 evaluations took 0.025778 [s].
 
Current level n = 2...
Warning: MaxDepth = 2 reached before accuracies RelTol = 0.01 or AbsTol = 1e-06 were achieved.
The current estimated relative accuracy is 0.014598.
Computing sparse grid points and evaluating function took 0.06666 [s].
Computing hierarchical surpluses took 0.001837 [s].
Performing 1000 evaluations took 0.37137 [s].
 
Current level n = 3...
Computing sparse grid points and evaluating function took 0.47255 [s].
Computing hierarchical surpluses took 0.033908 [s].
Performing 1000 evaluations took 3.2319 [s].
 
Current level n = 4...
Computing sparse grid points and evaluating function took 2.8053 [s].
Computing hierarchical surpluses took 0.62862 [s].
Performing 1000 evaluations took 20.2791 [s].
 
Done!
```

## timespvalsdct

```
rand('seed',0); timespvalsdct
```

```
Current dim: 1
Current level n = 0...
Warning: MaxDepth = 0 reached before accuracies RelTol = 0.01 or AbsTol = 1e-06 were achieved.
The current estimated relative accuracy is Inf.
Computing sparse grid points and evaluating function took 0.004079 [s].
Computing hierarchical surpluses took 0 [s].
 
Current level n = 1...
Warning: MaxDepth = 1 reached before accuracies RelTol = 0.01 or AbsTol = 1e-06 were achieved.
The current estimated relative accuracy is 0.71126.
Computing sparse grid points and evaluating function took 0.008644 [s].
Computing hierarchical surpluses took 0.000362 [s].
 
Current level n = 2...
Warning: MaxDepth = 2 reached before accuracies RelTol = 0.01 or AbsTol = 1e-06 were achieved.
The current estimated relative accuracy is 0.020001.
Computing sparse grid points and evaluating function took 0.012888 [s].
Computing hierarchical surpluses took 0.001174 [s].
 
Current level n = 3...
Computing sparse grid points and evaluating function took 0.01709 [s].
Computing hierarchical surpluses took 0.002206 [s].
 
Current level n = 4...
Computing sparse grid points and evaluating function took 0.022501 [s].
Computing hierarchical surpluses took 0.003588 [s].
 
Current level n = 5...
Computing sparse grid points and evaluating function took 0.026663 [s].
Computing hierarchical surpluses took 0.005768 [s].
 
Current level n = 6...
Computing sparse grid points and evaluating function took 0.030848 [s].
Computing hierarchical surpluses took 0.008959 [s].
 
Current level n = 7...
Computing sparse grid points and evaluating function took 0.035195 [s].
Computing hierarchical surpluses took 0.014815 [s].
 
Current level n = 8...
Computing sparse grid points and evaluating function took 0.039659 [s].
Computing hierarchical surpluses took 0.027538 [s].
 
Current level n = 9...
Computing sparse grid points and evaluating function took 0.044135 [s].
Computing hierarchical surpluses took 0.060433 [s].
 
Current level n = 10...
Computing sparse grid points and evaluating function took 0.04878 [s].
Computing hierarchical surpluses took 0.15324 [s].
 
Current level n = 11...
Computing sparse grid points and evaluating function took 0.053404 [s].
Computing hierarchical surpluses took 0.45475 [s].
 
Current level n = 12...
Computing sparse grid points and evaluating function took 0.058454 [s].
Computing hierarchical surpluses took 1.474 [s].
 
Current level n = 13...
Computing sparse grid points and evaluating function took 0.06441 [s].
Computing hierarchical surpluses took 5.2346 [s].
 
Current level n = 14...
Computing sparse grid points and evaluating function took 0.07212 [s].
Computing hierarchical surpluses took 19.4916 [s].
 
Current dim: 1
Current level n = 0...
Warning: MaxDepth = 0 reached before accuracies RelTol = 0.01 or AbsTol = 1e-06 were achieved.
The current estimated relative accuracy is Inf.
Computing sparse grid points and evaluating function took 0.004333 [s].
Computing hierarchical surpluses took 0 [s].
 
Current level n = 1...
Warning: MaxDepth = 1 reached before accuracies RelTol = 0.01 or AbsTol = 1e-06 were achieved.
The current estimated relative accuracy is 0.71126.
Computing sparse grid points and evaluating function took 0.008672 [s].
Computing hierarchical surpluses took 0.000325 [s].
 
Current level n = 2...
Warning: MaxDepth = 2 reached before accuracies RelTol = 0.01 or AbsTol = 1e-06 were achieved.
The current estimated relative accuracy is 0.020001.
Computing sparse grid points and evaluating function took 0.012987 [s].
Computing hierarchical surpluses took 0.001078 [s].
 
Current level n = 3...
Computing sparse grid points and evaluating function took 0.018445 [s].
Computing hierarchical surpluses took 0.00198 [s].
 
Current level n = 4...
Computing sparse grid points and evaluating function took 0.022595 [s].
Computing hierarchical surpluses took 0.003044 [s].
 
Current level n = 5...
Computing sparse grid points and evaluating function took 0.027014 [s].
Computing hierarchical surpluses took 0.004366 [s].
 
Current level n = 6...
Computing sparse grid points and evaluating function took 0.031324 [s].
Computing hierarchical surpluses took 0.006004 [s].
 
Current level n = 7...
Computing sparse grid points and evaluating function took 0.03549 [s].
Computing hierarchical surpluses took 0.007986 [s].
 
Current level n = 8...
Computing sparse grid points and evaluating function took 0.04058 [s].
Computing hierarchical surpluses took 0.010445 [s].
 
Current level n = 9...
Computing sparse grid points and evaluating function took 0.044945 [s].
Computing hierarchical surpluses took 0.013677 [s].
 
Current level n = 10...
Computing sparse grid points and evaluating function took 0.049339 [s].
Computing hierarchical surpluses took 0.018496 [s].
 
Current level n = 11...
Computing sparse grid points and evaluating function took 0.053938 [s].
Computing hierarchical surpluses took 0.0262 [s].
 
Current level n = 12...
Computing sparse grid points and evaluating function took 0.059023 [s].
Computing hierarchical surpluses took 0.04147 [s].
 
Current level n = 13...
Computing sparse grid points and evaluating function took 0.064895 [s].
Computing hierarchical surpluses took 0.0787 [s].
 
Current level n = 14...
Computing sparse grid points and evaluating function took 0.07268 [s].
Computing hierarchical surpluses took 0.17897 [s].
 
Current level n = 15...
Computing sparse grid points and evaluating function took 0.08438 [s].
Computing hierarchical surpluses took 0.41763 [s].
 
Current level n = 16...
Computing sparse grid points and evaluating function took 0.10683 [s].
Computing hierarchical surpluses took 0.97156 [s].
 
Current level n = 17...
Computing sparse grid points and evaluating function took 0.14733 [s].
Computing hierarchical surpluses took 2.266 [s].
 
Current dim: 2
Current level n = 0...
Warning: MaxDepth = 0 reached before accuracies RelTol = 0.01 or AbsTol = 1e-06 were achieved.
The current estimated relative accuracy is Inf.
Computing sparse grid points and evaluating function took 0.004187 [s].
Computing hierarchical surpluses took 0 [s].
 
Current level n = 1...
Warning: MaxDepth = 1 reached before accuracies RelTol = 0.01 or AbsTol = 1e-06 were achieved.
The current estimated relative accuracy is 0.56309.
Computing sparse grid points and evaluating function took 0.008658 [s].
Computing hierarchical surpluses took 0.000428 [s].
 
Current level n = 2...
Warning: MaxDepth = 2 reached before accuracies RelTol = 0.01 or AbsTol = 1e-06 were achieved.
The current estimated relative accuracy is 0.083534.
Computing sparse grid points and evaluating function took 0.013518 [s].
Computing hierarchical surpluses took 0.002254 [s].
 
Current level n = 3...
Computing sparse grid points and evaluating function took 0.018969 [s].
Computing hierarchical surpluses took 0.006725 [s].
 
Current level n = 4...
Computing sparse grid points and evaluating function took 0.026147 [s].
Computing hierarchical surpluses took 0.016687 [s].
 
Current level n = 5...
Computing sparse grid points and evaluating function took 0.032429 [s].
Computing hierarchical surpluses took 0.035816 [s].
 
Current level n = 6...
Computing sparse grid points and evaluating function took 0.039185 [s].
Computing hierarchical surpluses took 0.075219 [s].
 
Current level n = 7...
Computing sparse grid points and evaluating function took 0.046349 [s].
Computing hierarchical surpluses took 0.15502 [s].
 
Current level n = 8...
Computing sparse grid points and evaluating function took 0.054404 [s].
Computing hierarchical surpluses took 0.33933 [s].
 
Current level n = 9...
Computing sparse grid points and evaluating function took 0.063297 [s].
Computing hierarchical surpluses took 0.7912 [s].
 
Current level n = 10...
Computing sparse grid points and evaluating function took 0.073653 [s].
Computing hierarchical surpluses took 2.018 [s].
 
Current level n = 11...
Computing sparse grid points and evaluating function took 0.086266 [s].
Computing hierarchical surpluses took 5.4967 [s].
 
Current level n = 12...
Computing sparse grid points and evaluating function took 0.10466 [s].
Computing hierarchical surpluses took 16.201 [s].
 
Current dim: 2
Current level n = 0...
Warning: MaxDepth = 0 reached before accuracies RelTol = 0.01 or AbsTol = 1e-06 were achieved.
The current estimated relative accuracy is Inf.
Computing sparse grid points and evaluating function took 0.004061 [s].
Computing hierarchical surpluses took 0 [s].
 
Current level n = 1...
Warning: MaxDepth = 1 reached before accuracies RelTol = 0.01 or AbsTol = 1e-06 were achieved.
The current estimated relative accuracy is 1.
Computing sparse grid points and evaluating function took 0.010053 [s].
Computing hierarchical surpluses took 0.000357 [s].
 
Current level n = 2...
Warning: MaxDepth = 2 reached before accuracies RelTol = 0.01 or AbsTol = 1e-06 were achieved.
The current estimated relative accuracy is 0.42532.
Computing sparse grid points and evaluating function took 0.015059 [s].
Computing hierarchical surpluses took 0.001996 [s].
 
Current level n = 3...
Computing sparse grid points and evaluating function took 0.020541 [s].
Computing hierarchical surpluses took 0.005788 [s].
 
Current level n = 4...
Computing sparse grid points and evaluating function took 0.026361 [s].
Computing hierarchical surpluses took 0.013128 [s].
 
Current level n = 5...
Computing sparse grid points and evaluating function took 0.03293 [s].
Computing hierarchical surpluses took 0.026561 [s].
 
Current level n = 6...
Computing sparse grid points and evaluating function took 0.039568 [s].
Computing hierarchical surpluses took 0.049929 [s].
 
Current level n = 7...
Computing sparse grid points and evaluating function took 0.046745 [s].
Computing hierarchical surpluses took 0.085687 [s].
 
Current level n = 8...
Computing sparse grid points and evaluating function took 0.054862 [s].
Computing hierarchical surpluses took 0.14221 [s].
 
Current level n = 9...
Computing sparse grid points and evaluating function took 0.06401 [s].
Computing hierarchical surpluses took 0.22463 [s].
 
Current level n = 10...
Computing sparse grid points and evaluating function took 0.074325 [s].
Computing hierarchical surpluses took 0.35283 [s].
 
Current level n = 11...
Computing sparse grid points and evaluating function took 0.087094 [s].
Computing hierarchical surpluses took 0.55821 [s].
 
Current level n = 12...
Computing sparse grid points and evaluating function took 0.10634 [s].
Computing hierarchical surpluses took 0.92869 [s].
 
Current level n = 13...
Computing sparse grid points and evaluating function took 0.13691 [s].
Computing hierarchical surpluses took 1.6333 [s].
 
Current level n = 14...
Computing sparse grid points and evaluating function took 0.19287 [s].
Computing hierarchical surpluses took 3.2278 [s].
 
Current dim: 4
Current level n = 0...
Warning: MaxDepth = 0 reached before accuracies RelTol = 0.01 or AbsTol = 1e-06 were achieved.
The current estimated relative accuracy is Inf.
Computing sparse grid points and evaluating function took 0.004115 [s].
Computing hierarchical surpluses took 0 [s].
 
Current level n = 1...
Warning: MaxDepth = 1 reached before accuracies RelTol = 0.01 or AbsTol = 1e-06 were achieved.
The current estimated relative accuracy is 0.55517.
Computing sparse grid points and evaluating function took 0.008927 [s].
Computing hierarchical surpluses took 0.000484 [s].
 
Current level n = 2...
Warning: MaxDepth = 2 reached before accuracies RelTol = 0.01 or AbsTol = 1e-06 were achieved.
The current estimated relative accuracy is 0.064656.
Computing sparse grid points and evaluating function took 0.016187 [s].
Computing hierarchical surpluses took 0.005944 [s].
 
Current level n = 3...
Computing sparse grid points and evaluating function took 0.028284 [s].
Computing hierarchical surpluses took 0.034683 [s].
 
Current level n = 4...
Computing sparse grid points and evaluating function took 0.048562 [s].
Computing hierarchical surpluses took 0.13775 [s].
 
Current level n = 5...
Computing sparse grid points and evaluating function took 0.081154 [s].
Computing hierarchical surpluses took 0.45039 [s].
 
Current level n = 6...
Computing sparse grid points and evaluating function took 0.13185 [s].
Computing hierarchical surpluses took 1.3154 [s].
 
Current level n = 7...
Computing sparse grid points and evaluating function took 0.20431 [s].
Computing hierarchical surpluses took 3.643 [s].
 
Current level n = 8...
Computing sparse grid points and evaluating function took 0.30805 [s].
Computing hierarchical surpluses took 10.0334 [s].
 
Current level n = 9...
Computing sparse grid points and evaluating function took 0.46258 [s].
Computing hierarchical surpluses took 28.182 [s].
 
Current level n = 10...
Computing sparse grid points and evaluating function took 0.69607 [s].
Computing hierarchical surpluses took 82.2379 [s].
 
Current dim: 4
Current level n = 0...
Warning: MaxDepth = 0 reached before accuracies RelTol = 0.01 or AbsTol = 1e-06 were achieved.
The current estimated relative accuracy is Inf.
Computing sparse grid points and evaluating function took 0.004124 [s].
Computing hierarchical surpluses took 0 [s].
 
Current level n = 1...
Warning: MaxDepth = 1 reached before accuracies RelTol = 0.01 or AbsTol = 1e-06 were achieved.
The current estimated relative accuracy is 0.64154.
Computing sparse grid points and evaluating function took 0.009053 [s].
Computing hierarchical surpluses took 0.000424 [s].
 
Current level n = 2...
Warning: MaxDepth = 2 reached before accuracies RelTol = 0.01 or AbsTol = 1e-06 were achieved.
The current estimated relative accuracy is 0.14293.
Computing sparse grid points and evaluating function took 0.016344 [s].
Computing hierarchical surpluses took 0.004455 [s].
 
Current level n = 3...
Warning: MaxDepth = 3 reached before accuracies RelTol = 0.01 or AbsTol = 1e-06 were achieved.
The current estimated relative accuracy is 0.01402.
Computing sparse grid points and evaluating function took 0.028199 [s].
Computing hierarchical surpluses took 0.026563 [s].
 
Current level n = 4...
Computing sparse grid points and evaluating function took 0.048255 [s].
Computing hierarchical surpluses took 0.10959 [s].
 
Current level n = 5...
Computing sparse grid points and evaluating function took 0.081222 [s].
Computing hierarchical surpluses took 0.34808 [s].
 
Current level n = 6...
Computing sparse grid points and evaluating function took 0.13106 [s].
Computing hierarchical surpluses took 0.94729 [s].
 
Current level n = 7...
Computing sparse grid points and evaluating function took 0.20518 [s].
Computing hierarchical surpluses took 2.308 [s].
 
Current level n = 8...
Computing sparse grid points and evaluating function took 0.31302 [s].
Computing hierarchical surpluses took 5.1347 [s].
 
Current level n = 9...
Computing sparse grid points and evaluating function took 0.46996 [s].
Computing hierarchical surpluses took 10.6777 [s].
 
Current level n = 10...
Computing sparse grid points and evaluating function took 0.71273 [s].
Computing hierarchical surpluses took 21.2348 [s].
 
Current dim: 8
Current level n = 0...
Warning: MaxDepth = 0 reached before accuracies RelTol = 0.01 or AbsTol = 1e-06 were achieved.
The current estimated relative accuracy is Inf.
Computing sparse grid points and evaluating function took 0.004395 [s].
Computing hierarchical surpluses took 0 [s].
 
Current level n = 1...
Warning: MaxDepth = 1 reached before accuracies RelTol = 0.01 or AbsTol = 1e-06 were achieved.
The current estimated relative accuracy is 0.53445.
Computing sparse grid points and evaluating function took 0.009697 [s].
Computing hierarchical surpluses took 0.000755 [s].
 
Current level n = 2...
Warning: MaxDepth = 2 reached before accuracies RelTol = 0.01 or AbsTol = 1e-06 were achieved.
The current estimated relative accuracy is 0.037629.
Computing sparse grid points and evaluating function took 0.025905 [s].
Computing hierarchical surpluses took 0.021455 [s].
 
Current level n = 3...
Computing sparse grid points and evaluating function took 0.084669 [s].
Computing hierarchical surpluses took 0.23246 [s].
 
Current level n = 4...
Computing sparse grid points and evaluating function took 0.27017 [s].
Computing hierarchical surpluses took 1.5868 [s].
 
Current level n = 5...
Computing sparse grid points and evaluating function took 0.76538 [s].
Computing hierarchical surpluses took 8.4921 [s].
 
Current level n = 6...
Computing sparse grid points and evaluating function took 1.9675 [s].
Computing hierarchical surpluses took 39.7502 [s].
 
Current dim: 8
Current level n = 0...
Warning: MaxDepth = 0 reached before accuracies RelTol = 0.01 or AbsTol = 1e-06 were achieved.
The current estimated relative accuracy is Inf.
Computing sparse grid points and evaluating function took 0.004219 [s].
Computing hierarchical surpluses took 0 [s].
 
Current level n = 1...
Warning: MaxDepth = 1 reached before accuracies RelTol = 0.01 or AbsTol = 1e-06 were achieved.
The current estimated relative accuracy is 0.50126.
Computing sparse grid points and evaluating function took 0.009722 [s].
Computing hierarchical surpluses took 0.000547 [s].
 
Current level n = 2...
Computing sparse grid points and evaluating function took 0.027476 [s].
Computing hierarchical surpluses took 0.01428 [s].
 
Current level n = 3...
Computing sparse grid points and evaluating function took 0.085744 [s].
Computing hierarchical surpluses took 0.17305 [s].
 
Current level n = 4...
Computing sparse grid points and evaluating function took 0.2706 [s].
Computing hierarchical surpluses took 1.2263 [s].
 
Current level n = 5...
Computing sparse grid points and evaluating function took 0.76427 [s].
Computing hierarchical surpluses took 6.3337 [s].
 
Current level n = 6...
Computing sparse grid points and evaluating function took 1.9566 [s].
Computing hierarchical surpluses took 27.0535 [s].
```

## timespderiv

```
rand('seed',0); timespderiv
```

```
Current dim: 1
Current level n = 0...
Warning: MaxDepth = 0 reached before accuracies RelTol = 0.01 or AbsTol = 1e-06 were achieved.
The current estimated relative accuracy is Inf.
Computing sparse grid points and evaluating function took 0.004075 [s].
Computing hierarchical surpluses took 0 [s].
100 evals took 0.00045695 [s], with gradients 0.0017895 [s]. Ratio: 3.9163
 
Current level n = 1...
Warning: MaxDepth = 1 reached before accuracies RelTol = 0.01 or AbsTol = 1e-06 were achieved.
The current estimated relative accuracy is 0.55111.
Computing sparse grid points and evaluating function took 0.008469 [s].
Computing hierarchical surpluses took 0.000272 [s].
100 evals took 0.00067383 [s], with gradients 0.0020516 [s]. Ratio: 3.0447
 
Current level n = 2...
Warning: MaxDepth = 2 reached before accuracies RelTol = 0.01 or AbsTol = 1e-06 were achieved.
The current estimated relative accuracy is 0.012876.
Computing sparse grid points and evaluating function took 0.012643 [s].
Computing hierarchical surpluses took 0.000569 [s].
100 evals took 0.00092309 [s], with gradients 0.002317 [s]. Ratio: 2.51
 
Current level n = 3...
Computing sparse grid points and evaluating function took 0.017131 [s].
Computing hierarchical surpluses took 0.000803 [s].
100 evals took 0.0012274 [s], with gradients 0.002619 [s]. Ratio: 2.1337
 
Current level n = 4...
Computing sparse grid points and evaluating function took 0.021258 [s].
Computing hierarchical surpluses took 0.001051 [s].
100 evals took 0.0015538 [s], with gradients 0.0029309 [s]. Ratio: 1.8863
 
Current level n = 5...
Computing sparse grid points and evaluating function took 0.025394 [s].
Computing hierarchical surpluses took 0.001339 [s].
100 evals took 0.0018598 [s], with gradients 0.0032372 [s]. Ratio: 1.7407
 
Current level n = 6...
Computing sparse grid points and evaluating function took 0.02969 [s].
Computing hierarchical surpluses took 0.001726 [s].
100 evals took 0.0021708 [s], with gradients 0.0036167 [s]. Ratio: 1.6661
 
Current level n = 7...
Computing sparse grid points and evaluating function took 0.033919 [s].
Computing hierarchical surpluses took 0.002338 [s].
100 evals took 0.0024966 [s], with gradients 0.0038764 [s]. Ratio: 1.5527
 
Current level n = 8...
Computing sparse grid points and evaluating function took 0.038259 [s].
Computing hierarchical surpluses took 0.003471 [s].
100 evals took 0.0028167 [s], with gradients 0.0042379 [s]. Ratio: 1.5045
 
Current level n = 9...
Computing sparse grid points and evaluating function took 0.042658 [s].
Computing hierarchical surpluses took 0.005809 [s].
100 evals took 0.003103 [s], with gradients 0.0045436 [s]. Ratio: 1.4643
 
Current level n = 10...
Computing sparse grid points and evaluating function took 0.0469 [s].
Computing hierarchical surpluses took 0.011054 [s].
100 evals took 0.0034222 [s], with gradients 0.004879 [s]. Ratio: 1.4257
 
Current level n = 11...
Computing sparse grid points and evaluating function took 0.051526 [s].
Computing hierarchical surpluses took 0.024832 [s].
100 evals took 0.0037387 [s], with gradients 0.0051781 [s]. Ratio: 1.385
 
Current level n = 12...
Computing sparse grid points and evaluating function took 0.056049 [s].
Computing hierarchical surpluses took 0.054364 [s].
100 evals took 0.0040594 [s], with gradients 0.0055163 [s]. Ratio: 1.3589
 
Current level n = 13...
Computing sparse grid points and evaluating function took 0.061095 [s].
Computing hierarchical surpluses took 0.11932 [s].
100 evals took 0.0043172 [s], with gradients 0.0059312 [s]. Ratio: 1.3739
 
Current level n = 14...
Computing sparse grid points and evaluating function took 0.068028 [s].
Computing hierarchical surpluses took 0.26282 [s].
100 evals took 0.0046866 [s], with gradients 0.0062143 [s]. Ratio: 1.326
 
Current level n = 15...
Computing sparse grid points and evaluating function took 0.075897 [s].
Computing hierarchical surpluses took 0.56518 [s].
100 evals took 0.0049568 [s], with gradients 0.0065425 [s]. Ratio: 1.3199
 
Current level n = 16...
Computing sparse grid points and evaluating function took 0.090316 [s].
Computing hierarchical surpluses took 1.2148 [s].
100 evals took 0.0052234 [s], with gradients 0.0067695 [s]. Ratio: 1.296
 
Current level n = 17...
Computing sparse grid points and evaluating function took 0.11329 [s].
Computing hierarchical surpluses took 2.5951 [s].
100 evals took 0.005605 [s], with gradients 0.0071954 [s]. Ratio: 1.2837
 
Done!
 
Current dim: 2
Current level n = 0...
Warning: MaxDepth = 0 reached before accuracies RelTol = 0.01 or AbsTol = 1e-06 were achieved.
The current estimated relative accuracy is Inf.
Computing sparse grid points and evaluating function took 0.003901 [s].
Computing hierarchical surpluses took 0 [s].
100 evals took 0.00046739 [s], with gradients 0.0023673 [s]. Ratio: 5.0649
 
Current level n = 1...
Warning: MaxDepth = 1 reached before accuracies RelTol = 0.01 or AbsTol = 1e-06 were achieved.
The current estimated relative accuracy is 0.5429.
Computing sparse grid points and evaluating function took 0.008226 [s].
Computing hierarchical surpluses took 0.00028 [s].
100 evals took 0.00090272 [s], with gradients 0.0028605 [s]. Ratio: 3.1688
 
Current level n = 2...
Warning: MaxDepth = 2 reached before accuracies RelTol = 0.01 or AbsTol = 1e-06 were achieved.
The current estimated relative accuracy is 0.040298.
Computing sparse grid points and evaluating function took 0.013036 [s].
Computing hierarchical surpluses took 0.000591 [s].
100 evals took 0.0017195 [s], with gradients 0.0037244 [s]. Ratio: 2.1659
 
Current level n = 3...
Computing sparse grid points and evaluating function took 0.018185 [s].
Computing hierarchical surpluses took 0.000913 [s].
100 evals took 0.0031466 [s], with gradients 0.0052194 [s]. Ratio: 1.6587
 
Current level n = 4...
Computing sparse grid points and evaluating function took 0.02396 [s].
Computing hierarchical surpluses took 0.00134 [s].
100 evals took 0.0050401 [s], with gradients 0.0072563 [s]. Ratio: 1.4397
 
Current level n = 5...
Computing sparse grid points and evaluating function took 0.030002 [s].
Computing hierarchical surpluses took 0.002183 [s].
100 evals took 0.0074686 [s], with gradients 0.0099484 [s]. Ratio: 1.332
 
Current level n = 6...
Computing sparse grid points and evaluating function took 0.037934 [s].
Computing hierarchical surpluses took 0.004256 [s].
100 evals took 0.010509 [s], with gradients 0.013154 [s]. Ratio: 1.2518
 
Current level n = 7...
Computing sparse grid points and evaluating function took 0.044819 [s].
Computing hierarchical surpluses took 0.00998 [s].
100 evals took 0.01392 [s], with gradients 0.016743 [s]. Ratio: 1.2028
 
Current level n = 8...
Computing sparse grid points and evaluating function took 0.05257 [s].
Computing hierarchical surpluses took 0.026772 [s].
100 evals took 0.017979 [s], with gradients 0.021264 [s]. Ratio: 1.1827
 
Current level n = 9...
Computing sparse grid points and evaluating function took 0.060771 [s].
Computing hierarchical surpluses took 0.07594 [s].
100 evals took 0.02253 [s], with gradients 0.025619 [s]. Ratio: 1.1371
 
Current level n = 10...
Computing sparse grid points and evaluating function took 0.07017 [s].
Computing hierarchical surpluses took 0.2078 [s].
100 evals took 0.027447 [s], with gradients 0.031052 [s]. Ratio: 1.1314
 
Current level n = 11...
Computing sparse grid points and evaluating function took 0.08138 [s].
Computing hierarchical surpluses took 0.5474 [s].
100 evals took 0.032707 [s], with gradients 0.036898 [s]. Ratio: 1.1281
 
Current level n = 12...
Computing sparse grid points and evaluating function took 0.097435 [s].
Computing hierarchical surpluses took 1.4072 [s].
100 evals took 0.038305 [s], with gradients 0.043363 [s]. Ratio: 1.132
 
Current level n = 13...
Computing sparse grid points and evaluating function took 0.12283 [s].
Computing hierarchical surpluses took 3.575 [s].
100 evals took 0.045024 [s], with gradients 0.051109 [s]. Ratio: 1.1351
 
Done!
 
Current dim: 4
Current level n = 0...
Warning: MaxDepth = 0 reached before accuracies RelTol = 0.01 or AbsTol = 1e-06 were achieved.
The current estimated relative accuracy is Inf.
Computing sparse grid points and evaluating function took 0.004037 [s].
Computing hierarchical surpluses took 0 [s].
100 evals took 0.00050329 [s], with gradients 0.0024499 [s]. Ratio: 4.8678
 
Current level n = 1...
Warning: MaxDepth = 1 reached before accuracies RelTol = 0.01 or AbsTol = 1e-06 were achieved.
The current estimated relative accuracy is 0.53823.
Computing sparse grid points and evaluating function took 0.008767 [s].
Computing hierarchical surpluses took 0.000273 [s].
100 evals took 0.0013577 [s], with gradients 0.0033669 [s]. Ratio: 2.4799
 
Current level n = 2...
Warning: MaxDepth = 2 reached before accuracies RelTol = 0.01 or AbsTol = 1e-06 were achieved.
The current estimated relative accuracy is 0.037751.
Computing sparse grid points and evaluating function took 0.015825 [s].
Computing hierarchical surpluses took 0.000636 [s].
100 evals took 0.0043603 [s], with gradients 0.0067743 [s]. Ratio: 1.5536
 
Current level n = 3...
Computing sparse grid points and evaluating function took 0.028143 [s].
Computing hierarchical surpluses took 0.001373 [s].
100 evals took 0.011939 [s], with gradients 0.015334 [s]. Ratio: 1.2844
 
Current level n = 4...
Computing sparse grid points and evaluating function took 0.047459 [s].
Computing hierarchical surpluses took 0.003891 [s].
100 evals took 0.027684 [s], with gradients 0.032624 [s]. Ratio: 1.1784
 
Current level n = 5...
Computing sparse grid points and evaluating function took 0.077934 [s].
Computing hierarchical surpluses took 0.014268 [s].
100 evals took 0.057411 [s], with gradients 0.064629 [s]. Ratio: 1.1257
 
Current level n = 6...
Computing sparse grid points and evaluating function took 0.12404 [s].
Computing hierarchical surpluses took 0.059222 [s].
100 evals took 0.099686 [s], with gradients 0.1175 [s]. Ratio: 1.1787
 
Current level n = 7...
Computing sparse grid points and evaluating function took 0.194 [s].
Computing hierarchical surpluses took 0.23592 [s].
100 evals took 0.16896 [s], with gradients 0.19583 [s]. Ratio: 1.159
 
Current level n = 8...
Computing sparse grid points and evaluating function took 0.2937 [s].
Computing hierarchical surpluses took 0.85765 [s].
100 evals took 0.2707 [s], with gradients 0.30898 [s]. Ratio: 1.1414
 
Current level n = 9...
Computing sparse grid points and evaluating function took 0.43754 [s].
Computing hierarchical surpluses took 2.9695 [s].
100 evals took 0.40649 [s], with gradients 0.46734 [s]. Ratio: 1.1497
 
Current level n = 10...
Computing sparse grid points and evaluating function took 0.6595 [s].
Computing hierarchical surpluses took 9.9271 [s].
100 evals took 0.58196 [s], with gradients 0.67989 [s]. Ratio: 1.1683
 
Done!
 
Current dim: 8
Current level n = 0...
Warning: MaxDepth = 0 reached before accuracies RelTol = 0.01 or AbsTol = 1e-06 were achieved.
The current estimated relative accuracy is Inf.
Computing sparse grid points and evaluating function took 0.004352 [s].
Computing hierarchical surpluses took 0 [s].
100 evals took 0.00058673 [s], with gradients 0.0025611 [s]. Ratio: 4.3651
 
Current level n = 1...
Warning: MaxDepth = 1 reached before accuracies RelTol = 0.01 or AbsTol = 1e-06 were achieved.
The current estimated relative accuracy is 0.5278.
Computing sparse grid points and evaluating function took 0.009863 [s].
Computing hierarchical surpluses took 0.000281 [s].
100 evals took 0.0022602 [s], with gradients 0.0043944 [s]. Ratio: 1.9443
 
Current level n = 2...
Warning: MaxDepth = 2 reached before accuracies RelTol = 0.01 or AbsTol = 1e-06 were achieved.
The current estimated relative accuracy is 0.027811.
Computing sparse grid points and evaluating function took 0.026078 [s].
Computing hierarchical surpluses took 0.000888 [s].
100 evals took 0.013851 [s], with gradients 0.017803 [s]. Ratio: 1.2853
 
Current level n = 3...
Computing sparse grid points and evaluating function took 0.084724 [s].
Computing hierarchical surpluses took 0.005007 [s].
100 evals took 0.063399 [s], with gradients 0.076268 [s]. Ratio: 1.203
 
Current level n = 4...
Computing sparse grid points and evaluating function took 0.26351 [s].
Computing hierarchical surpluses took 0.045382 [s].
100 evals took 0.23024 [s], with gradients 0.27386 [s]. Ratio: 1.1894
 
Current level n = 5...
Computing sparse grid points and evaluating function took 0.73975 [s].
Computing hierarchical surpluses took 0.35072 [s].
100 evals took 0.68664 [s], with gradients 0.82054 [s]. Ratio: 1.195
 
Current level n = 6...
Computing sparse grid points and evaluating function took 1.8864 [s].
Computing hierarchical surpluses took 2.2376 [s].
100 evals took 1.7922 [s], with gradients 2.1573 [s]. Ratio: 1.2037
 
Done!
 
Current dim: 16
Current level n = 0...
Warning: MaxDepth = 0 reached before accuracies RelTol = 0.01 or AbsTol = 1e-06 were achieved.
The current estimated relative accuracy is Inf.
Computing sparse grid points and evaluating function took 0.004506 [s].
Computing hierarchical surpluses took 0 [s].
100 evals took 0.00072864 [s], with gradients 0.0027795 [s]. Ratio: 3.8146
 
Current level n = 1...
Warning: MaxDepth = 1 reached before accuracies RelTol = 0.01 or AbsTol = 1e-06 were achieved.
The current estimated relative accuracy is 0.51331.
Computing sparse grid points and evaluating function took 0.010972 [s].
Computing hierarchical surpluses took 0.000321 [s].
100 evals took 0.0041521 [s], with gradients 0.0065022 [s]. Ratio: 1.566
 
Current level n = 2...
Warning: MaxDepth = 2 reached before accuracies RelTol = 0.01 or AbsTol = 1e-06 were achieved.
The current estimated relative accuracy is 0.014598.
Computing sparse grid points and evaluating function took 0.065481 [s].
Computing hierarchical surpluses took 0.002024 [s].
100 evals took 0.049136 [s], with gradients 0.05788 [s]. Ratio: 1.178
 
Current level n = 3...
Computing sparse grid points and evaluating function took 0.46825 [s].
Computing hierarchical surpluses took 0.037619 [s].
100 evals took 0.40559 [s], with gradients 0.48539 [s]. Ratio: 1.1968
 
Current level n = 4...
Computing sparse grid points and evaluating function took 2.8221 [s].
Computing hierarchical surpluses took 0.70202 [s].
100 evals took 2.5106 [s], with gradients 3.0576 [s]. Ratio: 1.2179
 
Done!
```

|  |
| --- |
|  |
