# Supplementary material for: Multiple Model-Informed Open-Loop Control of Uncertain Intracellular Signaling Dynamics
Source: PLoS Comput Biol. 2014 Apr 10;10(4):e1003546. doi: 10.1371/journal.pcbi.1003546 (PMC3983080; doi:10.1371/journal.pcbi.1003546)
Supplement: Dataset S1 — Matlab code for proposed control algorithm and prediction models. Contains all Matlab code necessary to implement the proposed adaptive weighted multiple-model predictive control algorithm, as well as code for the prediction models. (ZIP) [file pcbi.1003546.s001.zip › AW_MMPC/spinterp_v5.1.1/help/spcgsearch.html]

spcgsearch :: (Sparse Grid Interpolation Toolbox)


|  |  |
| --- | --- |
| **Sparse Grid Interpolation Toolbox** |  |

# spcgsearch

Optimizes the sparse grid interpolant using the CG method. **Recommended for optimizing polynomial sparse grids (Chebyshev grid).** It is discouraged to apply this method to piecewise linear sparse grids since they are not smooth enough for the algorithm to perform well (use `spcompsearch` instead for these grid types).

## Syntax

`X = spcgsearch(Z)`  
`X = spcgsearch(Z,XBOX)`  
`X = spcgsearch(Z,XBOX,OPTIONS)`  
`[X,FVAL] = spcgsearch(...)`  
`[X,FVAL,EXITFLAG] = spcgsearch(...)`  
`[X,FVAL,EXITFLAG,OUTPUT] = spcgsearch(...)`  

## Description

`X = spcgsearch(Z)` Starts the search at the best available sparse grid point and attempts to find a local minimizer of the sparse grid interpolant `Z`. The entire range of the sparse grid interpolant is searched.

`X = spcgsearch(Z,XBOX)` Uses the search box `XBOX = [a1, b1; a2, b2; ...]`. The size of search box `XBOX` must be smaller than or equal to the range of the interpolant.

`X = spcgsearch(Z,XBOX,OPTIONS)` Minimizes with the default optimization parameters replaced by values in the structure `OPTIONS`, created with the `spoptimset` function. See `spoptimset` for details.

`[X,FVAL] = spcgsearch(...)` Returns the value of the sparse grid interpolant at `X`.

`[X,FVAL,EXITFLAG] = spcgsearch(...)` Returns an `EXITFLAG` that describes the exit condition of `spcgsearch`. Possible values of `EXITFLAG` and the corresponding exit conditions are

- `1`   `spcgsearch` converged to a solution `X`.- `0`   Maximum number of function evaluations or iterations reached.

`[X,FVAL,EXITFLAG,OUTPUT] = spcgsearch(...)` Returns a structure `OUTPUT` with the number of function evaluations in `OUTPUT.nFEvals`, the number of gradients in `.nGradEvals`, and the computing time in `.time`.

## Examples

Usually, the objective function will be expensive to evaluate. Here, we just consider the well-known the six-hump camel-back
for function simplicity.

```
f = @(x,y) (4-2.1.*x.^2+x.^4./3).*x.^2+x.*y+(-4+4.*y.^2).*y.^2;
```

Before applying the spcgsearch algorithm, we need to create a sparse grid interpolant of the objective function. This is done as usual using the spvals algorithm.

In preparation to calling spvals, we first set up the interpolant construction with adequate parameters. A conjugate gradient (CG) line search algorithm uses
derivatives to determine the search direction, it best to use the smooth Chebyshev grid in order to obtain an interpolant
with accurate, smooth derivatives. Furthermore, it is useful to keep the function values as they can be used by the optimization
algorithm to select good starting values for the optimization.

```
options = spset('keepFunctionValues','on', 'GridType', 'Chebyshev', ...
  'DimensionAdaptive', 'on', 'DimAdaptDegree', 1, 'MinPoints', 10);
```

We construct the interpolant for the range that we are interested in optimizing the objective function for.

```
range = [-3 3; -2 2];
```

Now, we are ready to construct the sparse grid interpolant.

```
z = spvals(f, 2, range, options)
```

```
z = 
               vals: {[37x1 double]}
           gridType: 'Chebyshev'
                  d: 2
              range: [2x2 double]
        estRelError: 6.7208e-16
        estAbsError: 1.1013e-13
         fevalRange: [-0.9706 162.9000]
         minGridVal: [0.5000 0.6913]
         maxGridVal: [0 0]
            nPoints: 37
          fevalTime: 0.0690
    surplusCompTime: 0.3137
            indices: [1x1 struct]
           maxLevel: [4 3]
      activeIndices: [4x1 uint32]
     activeIndices2: [11x1 uint32]
                  E: [Inf 108.9000 48 48.6000 10.7392 6 16.0000 7.1054e-15 1.1013e-13 7.1054e-15 1.4211e-14]
                  G: [11x1 double]
                 G2: [11x1 double]
       maxSetPoints: 4
           dimAdapt: 1
              fvals: {[37x1 double]}
```

Having obtained the interpolant, we can now search for the minimizer using spcgsearch. This is achieved by simply calling

```
[xopt, fval] = spcgsearch(z)
```

```
xopt =
   -0.0898
    0.7127
fval =
   -1.0316
```

There are multiple ways of configuring the search using an options structure defined with spoptimset. For instance, you can display information at each iteration. Additional information on the optimization can be obtained
by specifying optional left-hand parameters:

```
optoptions = spoptimset('Display', 'iter');
[xopt, fval, exitflag, output] = spcgsearch(z, [], optoptions)
```

```
 Iteration   Func-count Grad-count     f(x)            Procedure
     0            1         1        -0.970563         start point
     1           10         1           -1.024         line search
     2           17         2         -1.03161         line search
     3           24         3         -1.03163         line search
     4           29         4         -1.03163         line search
xopt =
   -0.0898
    0.7127
fval =
   -1.0316
exitflag =
     1
output = 
       nFEvals: 29
    nGradEvals: 4
          time: 0.2737
```

## See Also

`spoptimset`.

|  |
| --- |
|  |
