# Supplementary material for: Multiple Model-Informed Open-Loop Control of Uncertain Intracellular Signaling Dynamics
Source: PLoS Comput Biol. 2014 Apr 10;10(4):e1003546. doi: 10.1371/journal.pcbi.1003546 (PMC3983080; doi:10.1371/journal.pcbi.1003546)
Supplement: Dataset S1 — Matlab code for proposed control algorithm and prediction models. Contains all Matlab code necessary to implement the proposed adaptive weighted multiple-model predictive control algorithm, as well as code for the prediction models. (ZIP) [file pcbi.1003546.s001.zip › AW_MMPC/spinterp_v5.1.1/help/spcompsearch.html]

spcompsearch :: (Sparse Grid Interpolation Toolbox)


|  |  |
| --- | --- |
| **Sparse Grid Interpolation Toolbox** |  |

# spcompsearch

Optimizes the sparse grid interpolant using the compass (coordinate) search method. Best-suited for piecewise multilinear sparse grids.

## Syntax

`X = spcompsearch(Z)`  
`X = spcompsearch(Z,XBOX)`  
`X = spcompsearch(Z,XBOX,OPTIONS)`  
`[X,FVAL] = spcompsearch(...)`  
`[X,FVAL,EXITFLAG] = spcompsearch(...)`  
`[X,FVAL,EXITFLAG,OUTPUT] = spcompsearch(...)`  

## Description

`X = spcompsearch(Z)` Starts the search at the best available sparse grid point and attempts to find a local minimizer of the sparse grid interpolant `Z`. The entire range of the sparse grid interpolant is searched.

`X = spcompsearch(Z,XBOX)` Uses the search box `XBOX = [a1, b1; a2, b2; ...]`. The size of search box `XBOX` must be smaller than or equal to the range of the interpolant.

`X = spcompsearch(Z,XBOX,OPTIONS)` Minimizes with the default optimization parameters replaced by values in the structure `OPTIONS`, created with the `spoptimset` function. See `spoptimset` for details.

`[X,FVAL] = spcompsearch(...)` Returns the value of the sparse grid interpolant at `X`.

`[X,FVAL,EXITFLAG] = spcompsearch(...)` Returns an `EXITFLAG` that describes the exit condition of `spcompsearch`. Possible values of `EXITFLAG` and the corresponding exit conditions are

- `1`   `spcompsearch` converged to a solution `X`.- `0`   Maximum number of function evaluations or iterations reached.

`[X,FVAL,EXITFLAG,OUTPUT] = spcompsearch(...)` Returns a structure `OUTPUT` with the number of function evaluations in `OUTPUT.nFEvals` and the computing time in `.time`.

## Examples

A compass search algorithm is a direct search method that does not need derivatives, so it is well-suited to optimize a piecewise
multilinear sparse grid interpolant computed for the grid types Maximum, NoBoundary, or Clenshaw-Curtis.

As with the example presented for spcgsearch, we consider the six-hump camel-back function.

```
f = @(x,y) (4-2.1.*x.^2+x.^4./3).*x.^2+x.*y+(-4+4.*y.^2).*y.^2;
```

We create the sparse grid interpolant using spvals as follows. Note that it is useful to keep the function values as they can be used by the optimization algorithm to select
good starting values for the optimization without having to evaluate the interpolant.

```
options = spset('keepFunctionValues','on', 'GridType', 'Clenshaw-Curtis', ...
  'DimensionAdaptive', 'on', 'DimAdaptDegree', 1, 'MinPoints', 10);
```

The next steps are setting the interpolation range (the optimization range will be the same by default), constructing the
interpolant, providing additional (optional) optimization parameters, and finally, the call to the spcompsearch algorithm.

```
range = [-3 3; -2 2];
z = spvals(f, 2, range, options)
optoptions = spoptimset('Display', 'iter');
[xopt, fval] = spcompsearch(z, [], optoptions)
```

```
z = 
               vals: {[205x1 double]}
           gridType: 'Clenshaw-Curtis'
                  d: 2
              range: [2x2 double]
        estRelError: 0.0037
        estAbsError: 0.6031
         fevalRange: [-0.9970 162.9000]
         minGridVal: [0.5000 0.3281]
         maxGridVal: [0 0]
            nPoints: 205
          fevalTime: 0.1031
    surplusCompTime: 0.0077
            indices: [1x1 struct]
           maxLevel: [7 6]
      activeIndices: [4x1 uint32]
     activeIndices2: [17x1 uint32]
                  E: [Inf 108.9000 48 52.2844 45.8547 6 24 12.7500 22.3788 4.3594 7.6817 0 0 1.2568 2.2379 0.3364 0.6031]
                  G: [17x1 double]
                 G2: [17x1 double]
       maxSetPoints: 7
           dimAdapt: 1
              fvals: {[205x1 double]}
 Iteration   Func-count Grad-count     f(x)            Procedure
     0            0         0        -0.997009         start point
     1            4         0        -0.997009         contract step
     2            8         0        -0.997009         contract step
     3           12         0        -0.997009         contract step
     4           16         0         -1.02647         coordinate step
     5           20         0         -1.02647         contract step
     6           24         0         -1.02647         contract step
xopt =
    0.0938
   -0.6875
fval =
   -1.0265
```

## See Also

`spoptimset`.

|  |
| --- |
|  |
