# Supplementary material for: Multiple Model-Informed Open-Loop Control of Uncertain Intracellular Signaling Dynamics
Source: PLoS Comput Biol. 2014 Apr 10;10(4):e1003546. doi: 10.1371/journal.pcbi.1003546 (PMC3983080; doi:10.1371/journal.pcbi.1003546)
Supplement: Dataset S1 — Matlab code for proposed control algorithm and prediction models. Contains all Matlab code necessary to implement the proposed adaptive weighted multiple-model predictive control algorithm, as well as code for the prediction models. (ZIP) [file pcbi.1003546.s001.zip › AW_MMPC/spinterp_v5.1.1/help/spdim.html]

spdim :: (Sparse Grid Interpolation Toolbox)


|  |  |
| --- | --- |
| **Sparse Grid Interpolation Toolbox** |  |

# spdim

Computes the number of sparse grid points.

## Syntax

`P = spdim(N,D)`  
`P = spdim(N,D,OPTIONS)`  

## Description

`P = spdim(N,D)` Computes the number of points of the sparse grid of dimension `D` and level `N`.

`P = spdim(N,D,OPTIONS)` Computes the number of points as above, but
with the default grid type replaced by the grid type specified in
`OPTIONS/, an argument created with
spset. See spset for details.

## Examples

Compute the number of support nodes of the 10-dimensional sparse grid of level 7 for the Clenshaw-Curtis (default) grid with the following command:`

```
spdim(7,10)
```

```
ans =
      652065
```

For comparison, compute the number of nodes of the maximum-norm-based sparse grid:

```
options = spset('GridType','Maximum');
spdim(7,10,options)
```

```
ans =
   1.8317e+09
```

|  |
| --- |
|  |
