# Supplementary material for: Multiple Model-Informed Open-Loop Control of Uncertain Intracellular Signaling Dynamics
Source: PLoS Comput Biol. 2014 Apr 10;10(4):e1003546. doi: 10.1371/journal.pcbi.1003546 (PMC3983080; doi:10.1371/journal.pcbi.1003546)
Supplement: Text S1 — Additional methods and results. Contains details on specific model modification and additional simulated and experimental results. (DOC) [file pcbi.1003546.s002.doc]

Multiple model-informed open-loop control of uncertain intracellular signaling dynamics

Text S1

Jeffrey P. Perley^1^

Email: jperley@purdue.edu

Judith Mikolajczak^2^

Email: jmikola@purdue.edu

Marietta L. Harrison^2^

Email: harrisom@purdue.edu

Gregery T. Buzzard^3^

Email: buzzard@math.purdue.edu

Ann E. Rundell^1*^

^*^ Corresponding author

Email: rundell@purdue.edu

^1^Weldon School of Biomedical Engineering, Purdue University, West Lafayette, Indiana, USA 47906.

^2^Department of Medicinal Chemistry & Molecular Pharmacology, Purdue University, West Lafayette, Indiana, USA 47906.

^3^Department of Mathematics, Purdue University, West Lafayette, Indiana, USA 47906.

# Table of Contents

Table of Contents …..…………………………………………………………………………………………...2

1 Model Modifications ……………………………………………………………………………………………4

Control Reagent Dose Response Model ………………………………………………………………………..4

**Figure 1** Illustration of control reagent dose response model. (**A**) Effective dose as a function of time. (**B**) Effective parameter value as a function of time. …………………………………………………………4

**Figure 2** Illustration of Erk/MAPK activation cascade. …………………………………………………….4

**Table 1**  Model equations and parameters modified to simulate action of sanguinarine and U0126. ..……..5

**Table 2**  Model equations and parameters modified to simulate action of aZAP and iZAP. ………....……..6

Normalization of Output Trajectories for Comparison across Models …………………………………………6

**Table 3** Gain and offset factors for output trajectory normalization. ……………………………………….6

2 Additional Figures for *Materials and Methods* ...……………………………………………………………….7

**Figure 3** Akaike weight maps over Ω for (**A**) Model *Z*, (**B**) Model *L* and (**C**) Model *K*. …………………..7

**Figure 4** Illustration of the normalized normal constraint (NNC) method in a 2D objective space. ……….8

*3 In Silico* Experiments for Realistic Control Reagents …………………………………………………………..9

**Figure 5** Results for target (*t_off_*, *p_ss_*) = (8,0) and plant Model *Z*. …………………………..………………...9

**Figure 6** Results for target (*t_off_*, *p_ss_*) = (8,0) and plant Model *L*. …………………………..………………...9

**Figure 7** Results for target (*t_off_*, *p_ss_*) = (8,0) and plant Model *K*. …………………………..………………10

**Figure 8** Results for target (*t_off_*, *p_ss_*) = (15,0) and plant Model *Z*. …………………………..……………...10

**Figure 9** Results for target (*t_off_*, *p_ss_*) = (15,0) and plant Model *L*. ………………………….………………11

**Figure 10** Results for target (*t_off_*, *p_ss_*) = (15,0) and plant Model *K*. …………………………..……………11

**Figure 11** Results for target (*t_off_*, *p_ss_*) = (22,0) and plant Model *Z*. ………………………………………...12

**Figure 12** Results for target (*t_off_*, *p_ss_*) = (22,0) and plant Model *L*. …………………………...……….…...12

**Figure 13** Results for target (*t_off_*, *p_ss_*) = (22,0) and plant Model *K*. ………………………….…………….13

**Figure 14** Results for target (*t_off_*, *p_ss_*) = (8,0.25) and plant Model *Z*. …………………………..……..........13

**Figure 15** Results for target (*t_off_*, *p_ss_*) = (8,0.25) and plant Model *L*. …………………………..……..........14

**Figure 16** Results for target (*t_off_*, *p_ss_*) = (8,0.25) and plant Model *K*. …………………………..…….........14

**Figure 17** Results for target (*t_off_*, *p_ss_*) = (15,0.25) and plant Model *Z*. …………………………..…............15

**Figure 18** Results for target (*t_off_*, *p_ss_*) = (15,0.25) and plant Model *L*. …………………………..…............15

**Figure 19** Results for target (*t_off_*, *p_ss_*) = (15,0.25) and plant Model *K*. ………………………….…............16

**Figure 20** Results for target (*t_off_*, *p_ss_*) = (22,0.25) and plant Model *Z*. …………………………..…............16

**Figure 21** Results for target (*t_off_*, *p_ss_*) = (22,0.25) and plant Model *L*. …………………………..…............17

**Figure 22** Results for target (*t_off_*, *p_ss_*) = (22,0.25) and plant Model *K*. …………………………...…..........17

**Figure 23** Results for target (*t_off_*, *p_ss_*) = (8,0.5) and plant Model *Z*. ……………………………..…………18

**Figure 24** Results for target (*t_off_*, *p_ss_*) = (8,0.5) and plant Model *L*. ……………………………..…………18

**Figure 25** Results for target (*t_off_*, *p_ss_*) = (8,0.5) and plant Model *K*. …………………………….…………19

**Figure 26** Results for target (*t_off_*, *p_ss_*) = (15,0.5) and plant Model *Z*. …………………………..……..........19

**Figure 27** Results for target (*t_off_*, *p_ss_*) = (15,0.5) and plant Model *L*. …………………………..…….........20

**Figure 28** Results for target (*t_off_*, *p_ss_*) = (15,0.5) and plant Model *K*. …………………………..…….........20

**Figure 29** Results for target (*t_off_*, *p_ss_*) = (22,0.5) and plant Model *Z*. …………………………..……..........21

**Figure 30** Results for target (*t_off_*, *p_ss_*) = (22,0.5) and plant Model *L*. …………………………..……..........21

**Figure 31** Results for target (*t_off_*, *p_ss_*) = (22,0.5) and plant Model *K*. ………………………….……..........22

**Figure 32** Results for target (*t_off_*, *p_ss_*) = (30,1) and plant Model *Z*. ……………………………..………….22

**Figure 33** Results for target (*t_off_*, *p_ss_*) = (30,1) and plant Model *L*. ……………………………..………….23

**Figure 34** Results for target (*t_off_*, *p_ss_*) = (30,1) and plant Model *K*. ……………………………..…………23

*4 In Silico* Experiments for Hypothetical Control Reagents …………….………………………………………24

**Figure 35** Results for target (*t_off_*, *p_ss_*) = (8,0) and plant Model *Z*. …………………………..……………...24

**Figure 36** Results for target (*t_off_*, *p_ss_*) = (8,0) and plant Model *L*. …………………………..……………...24

**Figure 37** Results for target (*t_off_*, *p_ss_*) = (8,0) and plant Model *K*. …………………………..……………..25

**Figure 38** Results for target (*t_off_*, *p_ss_*) = (15,0) and plant Model *Z*. …………………………..…………….25

**Figure 39** Results for target (*t_off_*, *p_ss_*) = (15,0) and plant Model *L*. ………………………….……………..26

**Figure 40** Results for target (*t_off_*, *p_ss_*) = (15,0) and plant Model *K*. …………………………..……………26

**Figure 41** Results for target (*t_off_*, *p_ss_*) = (22,0) and plant Model *Z*. ………………………………………...27

**Figure 42** Results for target (*t_off_*, *p_ss_*) = (22,0) and plant Model *L*. …………………………...……….…...27

**Figure 43** Results for target (*t_off_*, *p_ss_*) = (22,0) and plant Model *K*. ………………………….………….....28

**Figure 44** Results for target (*t_off_*, *p_ss_*) = (8,0.25) and plant Model *Z*. .…………………………..…….........28

**Figure 45** Results for target (*t_off_*, *p_ss_*) = (8,0.25) and plant Model *L*. …………………………..……..........29

**Figure 46** Results for target (*t_off_*, *p_ss_*) = (8,0.25) and plant Model *K*. …………………………..…….........29

**Figure 47** Results for target (*t_off_*, *p_ss_*) = (15,0.25) and plant Model *Z*. …………………………..…............30

**Figure 48** Results for target (*t_off_*, *p_ss_*) = (15,0.25) and plant Model *L*. …………………………..…............30

**Figure 49** Results for target (*t_off_*, *p_ss_*) = (15,0.25) and plant Model *K*. ………………………….…............31

**Figure 50** Results for target (*t_off_*, *p_ss_*) = (22,0.25) and plant Model *Z*. …………………………..…............31

**Figure 51** Results for target (*t_off_*, *p_ss_*) = (22,0.25) and plant Model *L*. …………………………..…............32

**Figure 52** Results for target (*t_off_*, *p_ss_*) = (22,0.25) and plant Model *K*. …………………………...…..........32

**Figure 53** Results for target (*t_off_*, *p_ss_*) = (8,0.5) and plant Model *Z*. ……………………………..…………33

**Figure 54** Results for target (*t_off_*, *p_ss_*) = (8,0.5) and plant Model *L*. ……………………………..…………33

**Figure 55** Results for target (*t_off_*, *p_ss_*) = (8,0.5) and plant Model *K*. …………………………….…………34

**Figure 56** Results for target (*t_off_*, *p_ss_*) = (15,0.5) and plant Model *Z*. …………………………..……..........34

**Figure 57** Results for target (*t_off_*, *p_ss_*) = (15,0.5) and plant Model *L*. …………………………..……..........35

**Figure 58** Results for target (*t_off_*, *p_ss_*) = (15,0.5) and plant Model *K*. …………………………..…….........35

**Figure 59** Results for target (*t_off_*, *p_ss_*) = (22,0.5) and plant Model *Z*. …………………………..……..........36

**Figure 60** Results for target (*t_off_*, *p_ss_*) = (22,0.5) and plant Model *L*. …………………………..……..........36

**Figure 61** Results for target (*t_off_*, *p_ss_*) = (22,0.5) and plant Model *K*. ………………………….……..........37

**Figure 62** Results for target (*t_off_*, *p_ss_*) = (30,1) and plant Model *Z*. ……………………………..………….37

**Figure 63** Results for target (*t_off_*, *p_ss_*) = (30,1) and plant Model *L*. ……………………………..………….38

**Figure 64** Results for target (*t_off_*, *p_ss_*) = (30,1) and plant Model *K*. ……………………………..…………38

5 *In Vitro* Experiments using Jurkat T Lymphocytes …………………………………………………………....39

**Table 4** Control reagent dosing schedules for *in vitro* experiments. ..……………………………………..39

**Table 5** Akaike weights (ω) determined by *M_aw_* for *in vitro* experiments. .……………………………….40

# 1 Model Modifications

### Control Reagent Dose Response Model

In this section we describe our modifications to the original mathematical models in order to simulate the observed behaviors of the chosen reagents as a practical means of control. To do this, we make several assumptions. First, we assume that each control reagent dose is administered in bolus form, but the effect of the control reagent is not instantaneous. After peak activity is reached, the control reagent is metabolized and the concentration returns to zero after a considerable period of time. According to these assumptions, we formulated the ‘effective’ dose concentration profile as the following:

$\mu_{j}(t)=\sum_{k=1}^{n_{_{\tau}}} u_{j}(k)\cdot\left(1-\exp(-(t-\tau_{j,i}(k))/\tau_{j,r})\right)\cdot\exp(-(t-\tau_{j,i}(k))/\tau_{j,d})\cdot1^{+}(t-\tau_{j,i}(k))$ (1)

where $u_{j}(k)$ is the administered concentration of the *j*^th^ control reagent at the *k*^th^ administration time point $\tau_{j,i}(k)$ and $\tau_{j,r}$ and $\tau_{j,d}$ are parameters characterizing the response time and duration of action for the *j*^th^ control reagent (Figure 1A). The Heaviside step function, denoted by $1^{+}(\cdot)$, ensures that control reagents are only effective after administration. To characterize the effect of the control reagent concentration on the dynamics of the simulated system, we refer to Figure 2, which shows a generalized diagram of the Erk activation cascade and the substrates upon which the control reagents act. Sanguinarine is an inhibitor of Erk phosphatase, which is responsible for catalyzing the dephosphorylation of Erk. Therefore, the addition of sanguinarine serves to reduce the rate of this reaction. Likewise, U0126 is an inhibitor of Mek, which catalyzes the phosphorylation of Erk. Thus, the addition of U0126 serves to reduce this reaction rate. The hypothetical reagents aZAP and iZAP have similar effects, but act on the targets of ZAP70 activity. These effects are implemented by modulating the rate parameters of the corresponding reactions as follows:

$k_{j,eff}=\frac{k_{j}}{1+c_{j}\mu_{j}(t)}$ (2)

where *k_j_* is the nominal value of the affected parameter and *c_j_* is a scaling factor used to adjust the sensitivity of the reaction rate to the reagent concentration. The generalized profile of *k_j_*,*_eff_* is illustrated in Figure 1B. The affected model equations, parameters and their nominal values and associated scaling factors are presented in Table 1.

**Figure 1** Illustration of control reagent dose response model. (**A**) Effective dose as a function of time. (**B**) Effective parameter value as a function of time.


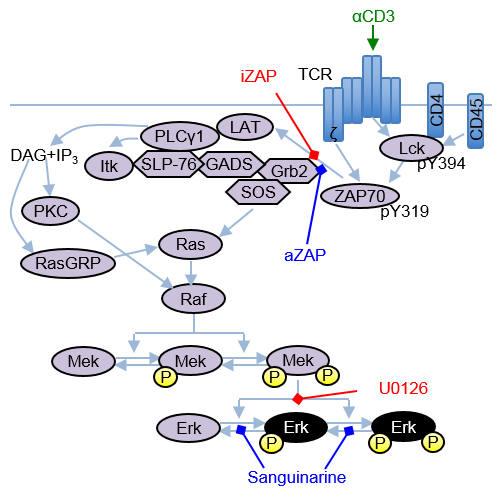


**Figure 2** Illustration of Erk/MAPK activation cascade.

| **Table 1** Model equations and parameters modified to simulate action of sanguinarine and U0126 | | | | |
| --- | --- | --- | --- | --- |
|  | Model | Equations | Parameters and Nominal Values |  |
|  | Zheng^*^ | $\frac{d[Erk*]}{dt}=k_{2}\cdot Mek*\cdot(Erk_{Total}-Erk*)-k_{1}\cdot(Erk*-Erk_{0}*)$ | *k*_1_ = 2.6880  *c*_1_ = 5  *k*_2_ = 8.7599e-5  *c*_2_ = 10 |  |
|  | Lipniacki^**^ | $\begin{matrix} \frac{d[Erk]}{dt}=k_{1}\cdot pErk-2\cdot k_{2}\cdot ppMek\cdot Erk \\ \frac{d[pErk]}{dt}=2\cdot k_{2}\cdot ppMek\cdot Erk+2\cdot k_{1}\cdot ppErk-k_{1}\cdot pErk-k_{2}\cdot ppMek\cdot pErk \\ \frac{d[ppErk]}{dt}=k_{2}\cdot ppMek\cdot pErk-2\cdot k_{1}\cdot ppErk \end{matrix}$ | *k*_1_ = 0.02  *c*_1_ = 0.3  *k*_2_ = 1.6667e-5  *c*_2_ = 60 |  |
|  | Klamt^***^ | $\frac{d[Erk*]}{dt}=\left(k_{2}\cdot\frac{Mek*^{n}}{Mek*^{n}+k_{Mek*}^{n}}-k_{1}Erk*\right)\cdot1/\tau_{Erk*}$ | *k*_1_ = 1  *c*_1_ = 0.9  *k*_2_ = 1  *c*_2_ = 9  *τ_dx_* = 11.0503 |  |
| ^*^For the Zheng model, *Erk** corresponds to activated Erk (i.e. [pErk] + [ppErk]) and *k*_1_ and *k*_2_ to parameters r18kr and r18kf, respectively. ^**^For the Lipniacki model, *k*_1_ and *k*_2_ correspond to parameters *e*_2_ and *e*_1_, respectively. ^***^For the Klamt model, *Erk** corresponds to activated Erk (i.e. [pErk] + [ppErk]) and *k*_1_ and *k*_2_ are new parameters. Additional parameter *τ_dx_* is multiplied to all reaction rates to ensure that dynamics are on the same time scale as other models and pre-existing experimental data. | | | | |

| **Table 2** Model equations and parameters modified to simulate action of aZAP and iZAP | | | | |
| --- | --- | --- | --- | --- |
|  | Model | Parameters | Nominal Values |  |
|  | Zheng | aZAP (r11_kf):  iZAP (1/r11_kf): | *k*_1_ = 7.8768e-8; *c*_1_ = 1;  *k*_2_ = 1/7.8768e-8; *c*_2_ = 10; |  |
|  | Lipniacki | aZAP (m­1):  iZAP (1/m1): | *k*_1_ = 2.5252e-5; *c*_1_ = 1;  *k*_2_ = 1/2.5252e-5; *c*_2_ = 10; |  |
|  | Klamt | aZAP (k_zap70):  iZAP (1/k_zap70): | *k*_1_ = 1; *c*_1_ = 1;  *k*_2_ = 1; *c*_2_ = 1; |  |
|  | | | | |

### Normalization of Output Trajectories for Comparison across Models

All model output trajectories were normalized with respect to the unperturbed output trajectory (i.e. no input reagents given) according to the following:

$\bar{y}_{j}^{(i)}(u,t)=\frac{y_{j}^{(i)}(u,t)-b_{j}^{(i)}}{a_{j}^{(i)}}$ (3)

where $b_{j}^{(i)}=y_{j}^{(i)}(0,t_{0})$ is the offset factor and $a_{j}^{(i)}=\max(y_{j}^{(i)}(0,t))-b_{j}^{(i)}$ is the gain factor. These constants correspond to the initial value and the range of the unperturbed trajectory, respectively. The factors for all three models are provided in Table 3.

| **Table 3** Gain and offset factors for output trajectory normalization | | | | |  |
| --- | --- | --- | --- | --- | --- |
|  | Model | Parameter | Value |  | |
|  | Zheng | $a_{1}^{(1)}$ $b_{1}^{(1)}$ | 9.9621e6  2096000 |  | |
|  | Lipniacki | $a_{1}^{(2)}$ $b_{1}^{(2)}$ | 2.7195e5  27670 |  | |
|  | Klamt | $a_{1}^{(3)}$ $b_{1}^{(3)}$ | 0.8848  0 |  | |
|  | | | | |  |

# 2 Supplemental Figures for *Materials and Methods*

The following figures are supplementary to the *Materials and Methods* section in the main text. Figure 3 accompanies *Model Weight Maps* and shows the weight maps for all three prediction models based on the training data displayed in Figure 7 in the main text. Figure 4 accompanies *Pareto Front Identification* and provides an intuitive geometric representation of the normalized normal constraint (NNC) algorithm for Pareto front identification.

**Figure 3** Akaike weight maps over Ω for (**A**) Model *Z*, (**B**) Model *L* and (**C**) Model *K*.

**Figure 4** Illustration of the normalized normal constraint (NNC) method in a 2D objective space.

# 3 *In Silico* Experiments for Realistic Control Reagents

The following figures are the results of all simulated experiments. Each figure corresponds to a different combination of the target output trajectory (i.e. $s_{i}(t)=(1-e^{-t})\cdot((1-p_{ss,i})(1+e^{t-t_{off,i}})^{-1}+p_{ss,i})$ where (*t_off_*, *p_ss_*) = {(8,0), (15,0), (22,0) , (8,0.25), (15,0.25), (22,0.25), (8,0.5), (15,0.5), (22,0.5), (30,1)}) and the plant or simulated experimental system (i.e. Model *Z*, Model *L* and Model *K*). Each figure shows (**A**-**E**) the control input dosing schedules for the matched and mismatched single-model controllers (*S_Z_*, *S_L_* and *S_K_*) and the multiple-model controllers with fixed equal weights (*M_eq_*) and with adaptive Akaike weights (*M_aw_*), (**F**) the Akaike weights for *M_aw_*, (**G**) the target trajectory (black) and simulated plant responses controlled by *S_Z_* (blue), *S_L_* (green), *S_K_* (red), *M_eq_* (cyan) and *M_aw_* (magenta), and (**H**) the target tracking performance as measured by squared error.

**Figure 5** Results for target (*t_off_*, *p_ss_*) = (8,0) and plant Model *Z*: (**A-E**) Control inputs, (**F**) Model weights, (**G**) plant simulations, and (**H**) target tracking performance.

**Figure 6** Results for target (*t_off_*, *p_ss_*) = (8,0) and plant Model *L*: (**A-E**) Control inputs, (**F**) Model weights, (**G**) plant simulations, and (**H**) target tracking performance.

**Figure 7** Results for target (*t_off_*, *p_ss_*) = (8,0) and plant Model *K*: (**A-E**) Control inputs, (**F**) Model weights, (**G**) plant simulations, and (**H**) target tracking performance.

**Figure 8** Results for target (*t_off_*, *p_ss_*) = (15,0) and plant Model *Z*: (**A-E**) Control inputs, (**F**) Model weights, (**G**) plant simulations, and (**H**) target tracking performance.

**Figure 9** Results for target (*t_off_*, *p_ss_*) = (15,0) and plant Model *L*: (**A-E**) Control inputs, (**F**) Model weights, (**G**) plant simulations, and (**H**) target tracking performance.

**Figure 10** Results for target (*t_off_*, *p_ss_*) = (15,0) and plant Model *K*: (**A-E**) Control inputs, (**F**) Model weights, (**G**) plant simulations, and (**H**) target tracking performance.

**Figure 11** Results for target (*t_off_*, *p_ss_*) = (22,0) and plant Model *Z*: (**A-E**) Control inputs, (**F**) Model weights, (**G**) plant simulations, and (**H**) target tracking performance.

**Figure 12** Results for target (*t_off_*, *p_ss_*) = (22,0) and plant Model *L*: (**A-E**) Control inputs, (**F**) Model weights, (**G**) plant simulations, and (**H**) target tracking performance.

**Figure 13** Results for target (*t_off_*, *p_ss_*) = (22,0) and plant Model *K*: (**A-E**) Control inputs, (**F**) Model weights, (**G**) plant simulations, and (**H**) target tracking performance.

**Figure 14** Results for target (*t_off_*, *p_ss_*) = (8,0.25) and plant Model *Z*: (**A-E**) Control inputs, (**F**) Model weights, (**G**) plant simulations, and (**H**) target tracking performance.

**Figure 15** Results for target (*t_off_*, *p_ss_*) = (8,0.25) and plant Model *L*: (**A-E**) Control inputs, (**F**) Model weights, (**G**) plant simulations, and (**H**) target tracking performance.

**Figure 16** Results for target (*t_off_*, *p_ss_*) = (8,0.25) and plant Model *K*: (**A-E**) Control inputs, (**F**) Model weights, (**G**) plant simulations, and (**H**) target tracking performance.

**Figure 17** Results for target (*t_off_*, *p_ss_*) = (15,0.25) and plant Model *Z*: (**A-E**) Control inputs, (**F**) Model weights, (**G**) plant simulations, and (**H**) target tracking performance.

**Figure 18** Results for target (*t_off_*, *p_ss_*) = (15,0.25) and plant Model *L*: (**A-E**) Control inputs, (**F**) Model weights, (**G**) plant simulations, and (**H**) target tracking performance.

**Figure 19** Results for target (*t_off_*, *p_ss_*) = (15,0.25) and plant Model *K*: (**A-E**) Control inputs, (**F**) Model weights, (**G**) plant simulations, and (**H**) target tracking performance.

**Figure 20** Results for target (*t_off_*, *p_ss_*) = (22,0.25) and plant Model *Z*: (**A-E**) Control inputs, (**F**) Model weights, (**G**) plant simulations, and (**H**) target tracking performance.

**Figure 21** Results for target (*t_off_*, *p_ss_*) = (22,0.25) and plant Model *L*: (**A-E**) Control inputs, (**F**) Model weights, (**G**) plant simulations, and (**H**) target tracking performance.

**Figure 22** Results for target (*t_off_*, *p_ss_*) = (22,0.25) and plant Model *K*: (**A-E**) Control inputs, (**F**) Model weights, (**G**) plant simulations, and (**H**) target tracking performance.

**Figure 23** Results for target (*t_off_*, *p_ss_*) = (8,0.5) and plant Model *Z*: (**A-E**) Control inputs, (**F**) Model weights, (**G**) plant simulations, and (**H**) target tracking performance.

**Figure 24** Results for target (*t_off_*, *p_ss_*) = (8,0.5) and plant Model *L*: (**A-E**) Control inputs, (**F**) Model weights, (**G**) plant simulations, and (**H**) target tracking performance.

**Figure 25** Results for target (*t_off_*, *p_ss_*) = (8,0.5) and plant Model *K*: (**A-E**) Control inputs, (**F**) Model weights, (**G**) plant simulations, and (**H**) target tracking performance.

**Figure 26** Results for target (*t_off_*, *p_ss_*) = (15,0.5) and plant Model *Z*: (**A-E**) Control inputs, (**F**) Model weights, (**G**) plant simulations, and (**H**) target tracking performance.

**Figure 27** Results for target (*t_off_*, *p_ss_*) = (15,0.5) and plant Model *L*: (**A-E**) Control inputs, (**F**) Model weights, (**G**) plant simulations, and (**H**) target tracking performance.

**Figure 28** Results for target (*t_off_*, *p_ss_*) = (15,0.5) and plant Model *K*: (**A-E**) Control inputs, (**F**) Model weights, (**G**) plant simulations, and (**H**) target tracking performance.

**Figure 29** Results for target (*t_off_*, *p_ss_*) = (22,0.5) and plant Model *Z*: (**A-E**) Control inputs, (**F**) Model weights, (**G**) plant simulations, and (**H**) target tracking performance.

**Figure 30** Results for target (*t_off_*, *p_ss_*) = (22,0.5) and plant Model *L*: (**A-E**) Control inputs, (**F**) Model weights, (**G**) plant simulations, and (**H**) target tracking performance.

**Figure 31** Results for target (*t_off_*, *p_ss_*) = (8,0.5) and plant Model *K*: (**A-E**) Control inputs, (**F**) Model weights, (**G**) plant simulations, and (**H**) target tracking performance.

**Figure 32** Results for target (*t_off_*, *p_ss_*) = (30,1) and plant Model *Z*: (**A-E**) Control inputs, (**F**) Model weights, (**G**) plant simulations, and (**H**) target tracking performance.

**Figure 33** Results for target (*t_off_*, *p_ss_*) = (30,1) and plant Model *L*: (**A-E**) Control inputs, (**F**) Model weights, (**G**) plant simulations, and (**H**) target tracking performance.

**Figure 34** Results for target (*t_off_*, *p_ss_*) = (30,1) and plant Model *K*: (**A-E**) Control inputs, (**F**) Model weights, (**G**) plant simulations, and (**H**) target tracking performance.

# 4 *In Silico* Experiments for Hypothetical Control Reagents

The following figures are the results of all simulated experiments. Each figure corresponds to a different combination of the target output trajectory (i.e. $s_{i}(t)=(1-e^{-t})\cdot((1-p_{ss,i})(1+e^{t-t_{off,i}})^{-1}+p_{ss,i})$ where (*t_off_*, *p_ss_*) = {(8,0), (15,0), (22,0) , (8,0.25), (15,0.25), (22,0.25), (8,0.5), (15,0.5), (22,0.5), (30,1)}) and the plant or simulated experimental system (i.e. Model *Z*, Model *L* and Model *K*). Each figure shows (**A**-**E**) the control input dosing schedules for the matched and mismatched single-model controllers (*S_Z_*, *S_L_* and *S_K_*) and the multiple-model controllers with fixed equal weights (*M_eq_*) and with adaptive Akaike weights (*M_aw_*), (**F**) the Akaike weights for *M_aw_*, (**G**) the target trajectory (black) and simulated plant responses controlled by *S_Z_* (blue), *S_L_* (green), *S_K_* (red), *M_eq_* (cyan) and *M_aw_* (magenta), and (**H**) the target tracking performance as measured by squared error.

**Figure 35** Results for target (*t_off_*, *p_ss_*) = (8,0) and plant Model *Z*: (**A-E**) Control inputs, (**F**) Model weights, (**G**) plant simulations, and (**H**) target tracking performance.

**Figure 36** Results for target (*t_off_*, *p_ss_*) = (8,0) and plant Model *L*: (**A-E**) Control inputs, (**F**) Model weights, (**G**) plant simulations, and (**H**) target tracking performance.

**Figure 37** Results for target (*t_off_*, *p_ss_*) = (8,0) and plant Model *K*: (**A-E**) Control inputs, (**F**) Model weights, (**G**) plant simulations, and (**H**) target tracking performance.

**Figure 38** Results for target (*t_off_*, *p_ss_*) = (15,0) and plant Model *Z*: (**A-E**) Control inputs, (**F**) Model weights, (**G**) plant simulations, and (**H**) target tracking performance.

**Figure 39** Results for target (*t_off_*, *p_ss_*) = (15,0) and plant Model *L*: (**A-E**) Control inputs, (**F**) Model weights, (**G**) plant simulations, and (**H**) target tracking performance.

**Figure 40** Results for target (*t_off_*, *p_ss_*) = (15,0) and plant Model *K*: (**A-E**) Control inputs, (**F**) Model weights, (**G**) plant simulations, and (**H**) target tracking performance.

**Figure 41** Results for target (*t_off_*, *p_ss_*) = (22,0) and plant Model *Z*: (**A-E**) Control inputs, (**F**) Model weights, (**G**) plant simulations, and (**H**) target tracking performance.

**Figure 42** Results for target (*t_off_*, *p_ss_*) = (22,0) and plant Model *L*: (**A-E**) Control inputs, (**F**) Model weights, (**G**) plant simulations, and (**H**) target tracking performance.

**Figure 43** Results for target (*t_off_*, *p_ss_*) = (22,0) and plant Model *K*: (**A-E**) Control inputs, (**F**) Model weights, (**G**) plant simulations, and (**H**) target tracking performance.

**Figure 44** Results for target (*t_off_*, *p_ss_*) = (8,0.25) and plant Model *Z*: (**A-E**) Control inputs, (**F**) Model weights, (**G**) plant simulations, and (**H**) target tracking performance.

**Figure 45** Results for target (*t_off_*, *p_ss_*) = (8,0.25) and plant Model *L*: (**A-E**) Control inputs, (**F**) Model weights, (**G**) plant simulations, and (**H**) target tracking performance.

**Figure 46** Results for target (*t_off_*, *p_ss_*) = (8,0.25) and plant Model *K*: (**A-E**) Control inputs, (**F**) Model weights, (**G**) plant simulations, and (**H**) target tracking performance.

**Figure 47** Results for target (*t_off_*, *p_ss_*) = (15,0.25) and plant Model *Z*: (**A-E**) Control inputs, (**F**) Model weights, (**G**) plant simulations, and (**H**) target tracking performance.

**Figure 48** Results for target (*t_off_*, *p_ss_*) = (15,0.25) and plant Model *L*: (**A-E**) Control inputs, (**F**) Model weights, (**G**) plant simulations, and (**H**) target tracking performance.

**Figure 49** Results for target (*t_off_*, *p_ss_*) = (15,0.25) and plant Model *K*: (**A-E**) Control inputs, (**F**) Model weights, (**G**) plant simulations, and (**H**) target tracking performance.

**Figure 50** Results for target (*t_off_*, *p_ss_*) = (22,0.25) and plant Model *Z*: (**A-E**) Control inputs, (**F**) Model weights, (**G**) plant simulations, and (**H**) target tracking performance.

**Figure 51** Results for target (*t_off_*, *p_ss_*) = (22,0.25) and plant Model *L*: (**A-E**) Control inputs, (**F**) Model weights, (**G**) plant simulations, and (**H**) target tracking performance.

**Figure 52** Results for target (*t_off_*, *p_ss_*) = (22,0.25) and plant Model *K*: (**A-E**) Control inputs, (**F**) Model weights, (**G**) plant simulations, and (**H**) target tracking performance.

**Figure 53** Results for target (*t_off_*, *p_ss_*) = (8,0.5) and plant Model *Z*: (**A-E**) Control inputs, (**F**) Model weights, (**G**) plant simulations, and (**H**) target tracking performance.

**Figure 54** Results for target (*t_off_*, *p_ss_*) = (8,0.5) and plant Model *L*: (**A-E**) Control inputs, (**F**) Model weights, (**G**) plant simulations, and (**H**) target tracking performance.

**Figure 55** Results for target (*t_off_*, *p_ss_*) = (8,0.5) and plant Model *K*: (**A-E**) Control inputs, (**F**) Model weights, (**G**) plant simulations, and (**H**) target tracking performance.

**Figure 56** Results for target (*t_off_*, *p_ss_*) = (15,0.5) and plant Model *Z*: (**A-E**) Control inputs, (**F**) Model weights, (**G**) plant simulations, and (**H**) target tracking performance.

**Figure 57** Results for target (*t_off_*, *p_ss_*) = (15,0.5) and plant Model *L*: (**A-E**) Control inputs, (**F**) Model weights, (**G**) plant simulations, and (**H**) target tracking performance.

**Figure 58** Results for target (*t_off_*, *p_ss_*) = (15,0.5) and plant Model *K*: (**A-E**) Control inputs, (**F**) Model weights, (**G**) plant simulations, and (**H**) target tracking performance.

**Figure 59** Results for target (*t_off_*, *p_ss_*) = (22,0.5) and plant Model *Z*: (**A-E**) Control inputs, (**F**) Model weights, (**G**) plant simulations, and (**H**) target tracking performance.

**Figure 60** Results for target (*t_off_*, *p_ss_*) = (22,0.5) and plant Model *L*: (**A-E**) Control inputs, (**F**) Model weights, (**G**) plant simulations, and (**H**) target tracking performance.

**Figure 61** Results for target (*t_off_*, *p_ss_*) = (8,0.5) and plant Model *K*: (**A-E**) Control inputs, (**F**) Model weights, (**G**) plant simulations, and (**H**) target tracking performance.

**Figure 62** Results for target (*t_off_*, *p_ss_*) = (30,1) and plant Model *Z*: (**A-E**) Control inputs, (**F**) Model weights, (**G**) plant simulations, and (**H**) target tracking performance.

**Figure 63** Results for target (*t_off_*, *p_ss_*) = (30,1) and plant Model *L*: (**A-E**) Control inputs, (**F**) Model weights, (**G**) plant simulations, and (**H**) target tracking performance.

**Figure 64** Results for target (*t_off_*, *p_ss_*) = (30,1) and plant Model *K*: (**A-E**) Control inputs, (**F**) Model weights, (**G**) plant simulations, and (**H**) target tracking performance.

# 5 *In Vitro* Experiments using Jurkat T Lymphocytes

This section summarizes the results for all of our *in vitro* experiments using Jurkat cell. The single-model controllers based on Model *Z* (*S_Z_*), Model *L* (*S_L_*) and Model *K* (*S_K_*), and the multiple-model control with adaptive weights (*M_aw_*) were tested using three different target profiles: (*t_off_*, *p_ss_*) = {(8,0), (15,0), (22,0)}. These targets correspond to full termination after 8, 15 and 22 min, respectively. Potential control input dosing times were 3, 8, 13, 18 and 23 min post-stimulation. The inputs quantities determined by each controller for all targets and time points are presented in Table 4. Table 5 shows the Akaike weights computed for *M_aw_* for all targets and time points. Figure 6 in the main text shows the quantitative Western blot data for all experiments.

| **Table 4** Control reagent dosing schedules for *in vitro* experiments | | | | | | | | | | | | | | | | | |
| --- | --- | --- | --- | --- | --- | --- | --- | --- | --- | --- | --- | --- | --- | --- | --- | --- | --- |
|  |  | | Controller | | Reagent (µM) | | 3 min | | 8 min | | 13 min | | 18 min | | 23 min | |  |
|  | Target 1 | | *S_Z_* | | Sanguinarine | | 0 | | 0 | | 0 | | 0 | | 0 | |  |
|  |  |  |  |  | U0126 | | 0.2 | | 6.0 | | 2.0 | | 0.5 | | 0.3 | |  |
|  |  |  | S_L_ | | Sanguinarine | | 0 | | 0 | | 1.0 | | 0.6 | | 0.7 | |  |
|  |  |  |  |  | U0126 | | 1.4 | | 3.2 | | 0 | | 0 | | 0 | |  |
|  |  |  | *S_K_* | | Sanguinarine | | 0 | | 0 | | 0 | | 0 | | 0 | |  |
|  |  |  |  |  | U0126 | | 0.2 | | 6.0 | | 5.5 | | 2.9 | | 2.1 | |  |
|  |  |  | *M_aw_* | | Sanguinarine | | 0 | | 0 | | 0.5 | | 0 | | 0.4 | |  |
|  |  |  |  |  | U0126 | | 0.2 | | 5.9 | | 5.2 | | 3.0 | | 2.0 | |  |
|  | Target 2 | | *S_Z_* | | Sanguinarine | | 1.1 | | 5.3 | | 0 | | 0 | | 0 | |  |
|  |  |  |  |  | U0126 | | 0 | | 0 | | 2.1 | | 6.8 | | 0.9 | |  |
|  |  |  | S_L_ | | Sanguinarine | | 0 | | 0.6 | | 0 | | 2.7 | | 1.2 | |  |
|  |  |  |  |  | U0126 | | 0.1 | | 0.2 | | 2.1 | | 0 | | 0 | |  |
|  |  |  | *S_K_* | | Sanguinarine | | 0 | | 0.1 | | 0 | | 0 | | 0 | |  |
|  |  |  |  |  | U0126 | | 0 | | 0 | | 1.8 | | 8.4 | | 3.5 | |  |
|  |  |  | *M_aw_* | | Sanguinarine | | 1.1 | | 5.3 | | 0 | | 0 | | 0 | |  |
|  |  |  |  |  | U0126 | | 0 | | 0 | | 2.2 | | 8.7 | | 3.7 | |  |
|  | Target 3 | | *S_Z_* | | Sanguinarine | | 1.0 | | 6.4 | | 12.4 | | 0 | | 0 | |  |
|  |  |  |  |  | U0126 | | 0 | | 0 | | 0 | | 0.3 | | 9.1 | |  |
|  |  |  | S_L_ | | Sanguinarine | | 0 | | 0.4 | | 0.7 | | 0 | | 0 | |  |
|  |  |  |  |  | U0126 | | 0.1 | | 0 | | 0 | | 0.8 | | 0.5 | |  |
|  |  |  | *S_K_* | | Sanguinarine | | 0 | | 0.3 | | 0.1 | | 0 | | 0 | |  |
|  |  |  |  |  | U0126 | | 0 | | 0 | | 0 | | 0.5 | | 8.4 | |  |
|  |  |  | *M_aw_* | | Sanguinarine | | 1.0 | | 6.4 | | 12.4 | | 0 | | 0 | |  |
|  |  |  |  |  | U0126 | | 0 | | 0 | | 0 | | 0.3 | | 10.0 | |  |
|  | | | | | | | | | | | | | | | | | |
| **Table 5** Akaike weights (ω) determined by *M_aw_* for *in vitro* experiments | | | | | | | | | | | | | | | | | |
|  | |  | | Prediction Model | | 3 min | | 8 min | | 13 min | | 18 min | | 23 min | |  | |
|  | | Target 1 | | *Z* | | 1.00 | | 0.00 | | 0.00 | | 0.00 | | 0.04 | |  | |
|  | |  |  | *L* | | 0.00 | | 0.00 | | 0.00 | | 0.00 | | 0.00 | |  | |
|  | |  |  | *K* | | 0.00 | | 1.00 | | 1.00 | | 1.00 | | 0.96 | |  | |
|  | | Target 2 | | *Z* | | 1.00 | | 1.00 | | 0.06 | | 0.01 | | 0.00 | |  | |
|  | |  |  | *L* | | 0.00 | | 0.00 | | 0.00 | | 0.00 | | 0.00 | |  | |
|  | |  |  | *K* | | 0.00 | | 0.00 | | 0.94 | | 0.99 | | 1.00 | |  | |
|  | | Target 3 | | *Z* | | 1.00 | | 1.00 | | 1.00 | | 1.00 | | 0.00 | |  | |
|  | |  |  | *L* | | 0.00 | | 0.00 | | 0.00 | | 0.00 | | 0.00 | |  | |
|  | |  |  | *K* | | 0.00 | | 0.00 | | 0.00 | | 0.00 | | 1.00 | |  | |
|  | | | | | | | | | | | | | | | | | |
