# Supplementary material for: Multiple Model-Informed Open-Loop Control of Uncertain Intracellular Signaling Dynamics
Source: PLoS Comput Biol. 2014 Apr 10;10(4):e1003546. doi: 10.1371/journal.pcbi.1003546 (PMC3983080; doi:10.1371/journal.pcbi.1003546)
Supplement: Dataset S1 — Matlab code for proposed control algorithm and prediction models. Contains all Matlab code necessary to implement the proposed adaptive weighted multiple-model predictive control algorithm, as well as code for the prediction models. (ZIP) [file pcbi.1003546.s001.zip › AW_MMPC/spinterp_v5.1.1/help/spfminsearch.html]

spfminsearch :: (Sparse Grid Interpolation Toolbox)


|  |  |
| --- | --- |
| **Sparse Grid Interpolation Toolbox** |  |

# spfminsearch

Optimizes the sparse grid interpolant using MATLAB's `fminsearch` method.

## Syntax

`X = spfminsearch(Z)`  
`X = spfminsearch(Z,XBOX)`  
`X = spfminsearch(Z,XBOX,OPTIONS)`  
`[X,FVAL] = spfminsearch(...)`  
`[X,FVAL,EXITFLAG] = spfminsearch(...)`  
`[X,FVAL,EXITFLAG,OUTPUT] = spfminsearch(...)`  

## Description

`X = spfminsearch(Z)` Starts the search at the best available sparse grid point and attempts to find a local minimizer of the sparse grid interpolant `Z`. The entire range of the sparse grid interpolant is searched.

`X = spfminsearch(Z,XBOX)` Uses the search box `XBOX = [a1, b1; a2, b2; ...]`. The size of search box `XBOX` must be smaller than or equal to the range of the interpolant.

`X = spfminsearch(Z,XBOX,OPTIONS)` Minimizes with the default optimization parameters replaced by values in the structure `OPTIONS`, created with the `spoptimset` function. See `spoptimset` for details.

`[X,FVAL] = spfminsearch(...)` Returns the value of the sparse grid interpolant at `X`.

`[X,FVAL,EXITFLAG] = spfminsearch(...)` Returns an `EXITFLAG` that describes the exit condition of `spfminsearch`. Possible values of `EXITFLAG` and the corresponding exit conditions are

- `1`   `spfminsearch` converged to a solution `X`.- `0`   Maximum number of function evaluations or iterations reached.

`[X,FVAL,EXITFLAG,OUTPUT] = spfminsearch(...)` Returns a structure `OUTPUT` with the number of function evaluations in `OUTPUT.nFEvals` and the computing time in `.time`. The `OUTPUT` result from the `fminsearch` call are returned as `OUTPUT.fminsearchOutput`.

## Examples

spfminsearch internally calls MATLAB's fminsearch function to perform the search. The sparse grid interpolant is modified by a penalty function such that the search is restricted
to the provided search box.

spfminsearch is a derivative-free method that is suitable for all sparse grid types. However, it is usually outperformed by spcompsearch for the grid types Maximum, NoBoundary, or Clenshaw-Curtis, and by spcgsearch for the grid type Chebyshev.

As with the example presented for spcgsearch, we consider the six-hump camel-back function (see that example for further details).

```
f = @(x,y) (4-2.1.*x.^2+x.^4./3).*x.^2+x.*y+(-4+4.*y.^2).*y.^2;
```

Interpolant creation and setting optional parameters:

```
options = spset('keepFunctionValues','on', 'GridType', 'Chebyshev', ...
  'DimensionAdaptive', 'on', 'DimAdaptDegree', 1, 'MinPoints', 10);
range = [-3 3; -2 2];
z = spvals(f, 2, range, options);
optoptions = spoptimset('Display', 'iter');
```

Performing the optimization:

```
[xopt, fval] = spfminsearch(z, [], optoptions)
```

```
 Iteration   Func-count     min f(x)         Procedure
     0            1        -0.970563         
     1            3        -0.970563         initial simplex
     2            5        -0.997137         expand
     3            7         -0.99731         reflect
     4            9         -0.99731         contract inside
     5           11        -0.999861         contract inside
     6           13         -1.00004         reflect
     7           15         -1.00004         contract inside
     8           17         -1.00004         contract inside
     9           19         -1.00004         contract inside
    10           21          -1.0002         expand
    11           23         -1.00055         expand
    12           25         -1.00087         expand
    13           27         -1.00192         expand
    14           29         -1.00227         expand
    15           31         -1.00483         expand
    16           32         -1.00483         reflect
    17           34         -1.00771         expand
    18           36         -1.01172         expand
    19           38         -1.01615         expand
    20           40         -1.02567         expand
    21           41         -1.02567         reflect
    22           43         -1.03063         reflect
    23           44         -1.03063         reflect
    24           46         -1.03083         reflect
    25           48         -1.03119         contract inside
    26           50         -1.03155         contract inside
    27           52         -1.03155         contract inside
    28           54         -1.03155         contract inside
    29           56         -1.03162         contract inside
    30           58         -1.03162         contract inside
    31           60         -1.03162         contract inside
    32           62         -1.03162         reflect
    33           64         -1.03163         contract inside
    34           66         -1.03163         contract inside
    35           68         -1.03163         contract inside
    36           70         -1.03163         contract inside
    37           72         -1.03163         contract inside
    38           74         -1.03163         contract inside
    39           76         -1.03163         contract inside
    40           78         -1.03163         contract inside
    41           80         -1.03163         contract inside
    42           82         -1.03163         reflect
    43           84         -1.03163         contract inside
 
Optimization terminated:
 the current x satisfies the termination criteria using OPTIONS.TolX of 1.000000e-04 
 and F(X) satisfies the convergence criteria using OPTIONS.TolFun of 1.000000e-04 

xopt =
   -0.0899
    0.7127
fval =
   -1.0316
```

## See Also

`spoptimset`.
