# Supplementary material for: Multiple Model-Informed Open-Loop Control of Uncertain Intracellular Signaling Dynamics
Source: PLoS Comput Biol. 2014 Apr 10;10(4):e1003546. doi: 10.1371/journal.pcbi.1003546 (PMC3983080; doi:10.1371/journal.pcbi.1003546)
Supplement: Dataset S1 — Matlab code for proposed control algorithm and prediction models. Contains all Matlab code necessary to implement the proposed adaptive weighted multiple-model predictive control algorithm, as well as code for the prediction models. (ZIP) [file pcbi.1003546.s001.zip › AW_MMPC/spinterp_v5.1.1/help/spget.html]

spget :: (Sparse Grid Interpolation Toolbox)


|  |  |
| --- | --- |
| **Sparse Grid Interpolation Toolbox** |  |

# spget

Get sparse grid interpolation `OPTIONS` parameters.

## Syntax

`VAL = spget(OPTIONS, 'NAME')`  
`VAL = spget(OPTIONS, 'NAME', DEFAULT)`  

## Description

`VAL = spget(OPTIONS, 'NAME')` Extracts the value of the property `NAME` from the sparse grid options structure `OPTIONS`, returning an empty matrix if the property value is not specified in `OPTIONS`. It is sufficient to type only the leading characters that uniquely identify the property. Case is ignored for property names. `[]` is a valid `OPTIONS` argument.

`VAL = spget(OPTIONS, 'NAME', DEFAULT)` Extracts the named property as above, but returns `VAL = DEFAULT` if the named property is not specified in `OPTIONS`.

## Examples

Assume that an options structure has been created using the spset command:

```
options = spset('GridType', 'Maximum', 'MaxDepth', 4)
```

```
options = 
              GridType: 'Maximum'
                RelTol: []
                AbsTol: []
            Vectorized: []
              MinDepth: []
              MaxDepth: 4
     VariablePositions: []
       NumberOfOutputs: []
           PrevResults: []
       FunctionArgType: []
    KeepFunctionValues: []
              KeepGrid: []
     DimensionAdaptive: []
             MinPoints: []
             MaxPoints: []
        DimadaptDegree: []
         SparseIndices: []
```

Using spget, we can extract the contents of the structure:

```
gridType = spget(options, 'GridType')
```

```
gridType =
Maximum
```

By using the third argument, we can set a default in case the according property of the structure is empty:

```
minDepth = spget(options, 'MinDepth')
minDepth = spget(options, 'MinDepth', 2)
```

```
minDepth =
     []

minDepth =
     2
```

Note that the default argument has no effect if the accessed property contains a value.

```
gridType = spget(options, 'GridType', 'Clenshaw-Curtis')
```

```
gridType =
Maximum
```

## See Also

`spset`.
