# Supplementary material for: Multiple Model-Informed Open-Loop Control of Uncertain Intracellular Signaling Dynamics
Source: PLoS Comput Biol. 2014 Apr 10;10(4):e1003546. doi: 10.1371/journal.pcbi.1003546 (PMC3983080; doi:10.1371/journal.pcbi.1003546)
Supplement: Dataset S1 — Matlab code for proposed control algorithm and prediction models. Contains all Matlab code necessary to implement the proposed adaptive weighted multiple-model predictive control algorithm, as well as code for the prediction models. (ZIP) [file pcbi.1003546.s001.zip › AW_MMPC/spinterp_v5.1.1/help/spgrid.html]

spgrid :: (Sparse Grid Interpolation Toolbox)


|  |  |
| --- | --- |
| **Sparse Grid Interpolation Toolbox** |  |

# spgrid

Compute the sparse grid point coordinates.

## Syntax

`X = spgrid(N,D)`  
`X = spgrid(N,D,OPTIONS)`  

## Description

`X = spgrid(N,D)` Computes the sparse grid points of level `N` and problem dimension `D`. The coordinate value of dimension `i = 1..d` is stored in column `i` of the matrix `X`. One row of the matrix `X` represents one grid point.

`X = spgrid(N,D,OPTIONS)` computes the sparse grid points as
above, but with default grid type replaced by the grid type specified in `OPTIONS`, an argument created with the `spset` function.

**Remark.** Note that `spgrid` only computes the grid points that are **added** to the interpolant at level `N`.

## Examples

Compute the grid points of the Clenshaw-Curtis (default) grid for the first 3 levels (n = 0..2), dimension d = 2, and display them:

```
d = 2;
for n = 0:2
  x = spgrid(n,d)
end
```

```
x =
    0.5000    0.5000

x =
         0    0.5000
    1.0000    0.5000
    0.5000         0
    0.5000    1.0000

x =
    0.2500    0.5000
    0.7500    0.5000
         0         0
    1.0000         0
         0    1.0000
    1.0000    1.0000
    0.5000    0.2500
    0.5000    0.7500
```

## See Also

`cmpgrids`,
`plotgrid`,
`spset`.
