# Supplementary material for: Multiple Model-Informed Open-Loop Control of Uncertain Intracellular Signaling Dynamics
Source: PLoS Comput Biol. 2014 Apr 10;10(4):e1003546. doi: 10.1371/journal.pcbi.1003546 (PMC3983080; doi:10.1371/journal.pcbi.1003546)
Supplement: Dataset S1 — Matlab code for proposed control algorithm and prediction models. Contains all Matlab code necessary to implement the proposed adaptive weighted multiple-model predictive control algorithm, as well as code for the prediction models. (ZIP) [file pcbi.1003546.s001.zip › AW_MMPC/spinterp_v5.1.1/help/spinit.html]

spinit :: (Sparse Grid Interpolation Toolbox)


|  |  |
| --- | --- |
| **Sparse Grid Interpolation Toolbox** |  |

# spinit

Initialize the Sparse Grid Interpolation toolbox.

## Syntax

`spinit`  

## Description

`spinit` Simply adds the sparse grid toolbox directories to the Matlab path. This ensures that the sparse grid routines are found when working in other directories.

**Remark.** To run the sparse grid interpolation demos from the help browser, `spinit` must be started first.
