# Supplementary material for: Multiple Model-Informed Open-Loop Control of Uncertain Intracellular Signaling Dynamics
Source: PLoS Comput Biol. 2014 Apr 10;10(4):e1003546. doi: 10.1371/journal.pcbi.1003546 (PMC3983080; doi:10.1371/journal.pcbi.1003546)
Supplement: Dataset S1 — Matlab code for proposed control algorithm and prediction models. Contains all Matlab code necessary to implement the proposed adaptive weighted multiple-model predictive control algorithm, as well as code for the prediction models. (ZIP) [file pcbi.1003546.s001.zip › AW_MMPC/spinterp_v5.1.1/help/spinterp.html]

spinterp :: (Sparse Grid Interpolation Toolbox)


|  |  |
| --- | --- |
| **Sparse Grid Interpolation Toolbox** |  |

# spinterp

Evaluation of the sparse grid interpolant.

## Syntax

`IP = spinterp(Z,Y1,...,YD)`  
`[IP,IPGRAD] = spinterp(Z,Y1,...,YD)`  

## Description

`IP = spinterp(Z,Y1,...,YD)` Computes the interpolated values `IP` at the point(s) `(Y1, ..., YD)` over the sparse grid. The input parameters `Yi` may be double arrays of equal size for vectorized processing. The sparse grid data must be given as a structure `Z` containing the hierarchical surpluses (computed with `spvals`).

`[IP,IPGRAD] = spinterp(Z,Y1,...,YD)` Computes interpolated values `IP` and derivatives `IPGRAD` at the specified points. The derivatives are returned as `D x 1` gradient vectors inside of a cell array that has equal size as the double array `IP`. See Section "Derivatives" for additional information.

Two additional options are available with `spinterp` that are set by adding a field to the structure `Z`:

- `selectOutput [ integer {1} ]` Set the output variable number if an interpolant with multiple output variables was constructed with `spvals`.- `continuousDerivatives [ 'on' | {'off'} ]` Enable augmented continuous derivatives for the Clenshaw-Curtis grid.

## Examples

Assume a sparse grid interpolant of the Matlab peaks function has been computed for the domain [0,2]^2 using the following command:

```
z = spvals(@(x,y) peaks(x,y), 2, [0,2; 0,2]);
```

Then, we can evaluate z at a single point, e.g. the point (0.5, 0.5), simply like this:

```
ip = spinterp(z, 0.5, 0.5);
```

```
ip =
    0.3754
```

If multiple evaluations of the interpolant are required, it is best to use a vectorized call to spinterp for fast processing.
For example, to evaluate the interpolant at the full grid [0,2]x[0,2] at 50x50 points (equidistant spacing), we can proceed as follows:

```
x = linspace(0,2,50); y = linspace(0,2,50);
[xmat,ymat] = meshgrid(x,y);
tic; ip = spinterp(z, xmat, ymat); toc
```

```
Elapsed time is 0.202514 seconds.
```

Note that the output size of ip matches the size of the input matrices:

```
size(xmat)
size(ip)
```

```
ans =
    50    50
ans =
    50    50
```

We could visualize the result using the `surf` command:

```
surf(xmat, ymat, ip);
axis tight;
```

## See Also

`spvals`.
