# Supplementary material for: Multiple Model-Informed Open-Loop Control of Uncertain Intracellular Signaling Dynamics
Source: PLoS Comput Biol. 2014 Apr 10;10(4):e1003546. doi: 10.1371/journal.pcbi.1003546 (PMC3983080; doi:10.1371/journal.pcbi.1003546)
Supplement: Dataset S1 — Matlab code for proposed control algorithm and prediction models. Contains all Matlab code necessary to implement the proposed adaptive weighted multiple-model predictive control algorithm, as well as code for the prediction models. (ZIP) [file pcbi.1003546.s001.zip › AW_MMPC/spinterp_v5.1.1/help/spinterptool.html]

Sparse Grid Interpolation Toolbox


**Sparse Grid Interpolation Web page**

## Functions

|  |  |
| --- | --- |
|  | **In Alphabetical Order** |

---

## Documentation

|  |  |
| --- | --- |
|  | **Getting Started** Introduces the Sparse Grid Interpolation Toolbox and gets you started using it |
|  | **Advanced Topics** Introduces advanced features and explains how to use the toolbox for solving real-world problems |
|  | **Examples in Documentation** Lists major examples in the Sparse Grid Interpolation Toolbox documentation |

---

## Demos

|  |  |
| --- | --- |
|  | **Sparse Grid Interpolation Toolbox Demos** Presents a collection of demos that you can run from the Help browser to help you learn the product |
|  | **Demos: Reference Results** Reference results of the provided demos |

---

## What's New

|  |  |
| --- | --- |
|  | **Release Notes**  Summarizes new features, bug fixes, upgrade issues, etc. |

---

## Printing the Documentation Set

|  |  |
| --- | --- |
| These links open PDF files provided with the software: | |
|  | **Sparse Grid Interpolation Toolbox Printed Documentation** |

(c) 2006-2008 W. Andreas Klimke, Universitaet Stuttgart | License
