# Supplementary material for: Multiple Model-Informed Open-Loop Control of Uncertain Intracellular Signaling Dynamics
Source: PLoS Comput Biol. 2014 Apr 10;10(4):e1003546. doi: 10.1371/journal.pcbi.1003546 (PMC3983080; doi:10.1371/journal.pcbi.1003546)
Supplement: Dataset S1 — Matlab code for proposed control algorithm and prediction models. Contains all Matlab code necessary to implement the proposed adaptive weighted multiple-model predictive control algorithm, as well as code for the prediction models. (ZIP) [file pcbi.1003546.s001.zip › AW_MMPC/spinterp_v5.1.1/help/spmultistart.html]

spmultistart :: (Sparse Grid Interpolation Toolbox)


|  |  |
| --- | --- |
| **Sparse Grid Interpolation Toolbox** |  |

# spmultistart

Attemps to find a global optimizer of the sparse grid interpolant by performing multiple local searches starting from random start points.

## Syntax

`X = spmultistart(Z)`  
`X = spmultistart(Z,XBOX)`  
`X = spmultistart(Z,XBOX,OPTIONS)`  
`[X,FVAL] = spmultistart(...)`  
`[X,FVAL,EXITFLAG] = spmultistart(...)`  
`[X,FVAL,EXITFLAG,OUTPUT] = spmultistart(...)`  

## Description

`X = spmultistart(Z)` Attemps to find a global optimizer `X` of the sparse grid interpolant `Z` by performing multiple local searches starting from random start points. The entire range of the sparse grid interpolant is searched. Using the default settings, the first start point is not random but the best available sparse grid point. By default, `spcompsearch` is used for the local searches if the grid type is not of type`Chebyshev`. If it is, `spcgsearch` is used.

`X = spmultistart(Z,XBOX)` Uses the search box `XBOX = [a1, b1; a2, b2; ...]`. The size of search box `XBOX` must be smaller than or equal to the range of the interpolant.

`X = spmultistart(Z,XBOX,OPTIONS)` Minimizes with the default optimization parameters replaced by values in the structure `OPTIONS`, created with the `spoptimset` function. For instance, the local optimization method can be selected. See `spoptimset` for details.

`[X,FVAL] = spmultistart(...)` Returns the value of the sparse grid interpolant at `X`.

`[X,FVAL,EXITFLAG] = spmultistart(...)` Returns an `EXITFLAG` that describes the exit condition of `spmultistart`. Possible values of `EXITFLAG` and the corresponding exit conditions are

- `1`   `spmultistart` converged to at least one solution `X`.- `0`   Maximum number of function evaluations or iterations reached for all local searches.

`[X,FVAL,EXITFLAG,OUTPUT] = spmultistart(...)` Returns a structure `OUTPUT` with the total computing time in `.time`, and a cell array of all local search results `.allResults`.

## Examples

The following example demonstrates the usage of spmultistart with its default options. Many parameters can be modified using spoptimset.

Here, we optimize Branin's function, a function with three global optimizers.

```
f = inline(['(5/pi*x-5.1/(4*pi^2)*x.^2+y-6).^2 +' ...
            '10*(1-1/(8*pi))*cos(x)+10']);
```

Interpolant creation:

```
range = [-5 10; 0 15];
options = spset('keepFunctionValues','on', 'GridType', 'Chebyshev', ...
                'DimensionAdaptive', 'on', 'DimAdaptDegree', 1, ...
                'MinPoints', 10);
z = spvals(f, 2, range, options);
```

Performing the optimization:

```
[xopt, fval, exitflag, output] = spmultistart(z)
```

```
xopt =
   -3.1416
   12.2751
fval =
    0.3978
exitflag =
     1
output = 
          time: 4.0003
    allResults: [1x1 struct]
```

All local optimization results are available from the output structure:

```
output.allResults.x
output.allResults.fval
```

```
ans =
    3.1416    9.4248    3.1416    9.4248    3.1416    9.4248   -3.1416    9.4248   -3.1416    9.4248
    2.2750    2.4750    2.2750    2.4750    2.2750    2.4750   12.2751    2.4750   12.2751    2.4750
ans =
    0.3980    0.3979    0.3980    0.3979    0.3980    0.3979    0.3978    0.3979    0.3978    0.3979
```

## See Also

`spoptimset`.
