# Supplementary material for: Multiple Model-Informed Open-Loop Control of Uncertain Intracellular Signaling Dynamics
Source: PLoS Comput Biol. 2014 Apr 10;10(4):e1003546. doi: 10.1371/journal.pcbi.1003546 (PMC3983080; doi:10.1371/journal.pcbi.1003546)
Supplement: Dataset S1 — Matlab code for proposed control algorithm and prediction models. Contains all Matlab code necessary to implement the proposed adaptive weighted multiple-model predictive control algorithm, as well as code for the prediction models. (ZIP) [file pcbi.1003546.s001.zip › AW_MMPC/spinterp_v5.1.1/help/spoptimget.html]

spoptimget :: (Sparse Grid Interpolation Toolbox)


|  |  |
| --- | --- |
| **Sparse Grid Interpolation Toolbox** |  |

# spoptimget

Get sparse grid optimization `OPTIONS` parameters.

## Syntax

`VAL = spoptimget(OPTIONS, 'NAME')`  
`VAL = spoptimget(OPTIONS, 'NAME', DEFAULT)`  

## Description

`VAL = spoptimget(OPTIONS, 'NAME')` Extracts the value of the property `NAME` from the sparse grid optimization options structure `OPTIONS`, returning an empty matrix if the property value is not specified in `OPTIONS`. It is sufficient to type only the leading characters that uniquely identify the property. Case is ignored for property names. `[]` is a valid `OPTIONS` argument.

`VAL = spoptimget(OPTIONS, 'NAME', DEFAULT)` Extracts the named property as above, but returns `VAL = DEFAULT` if the named property is not specified in `OPTIONS`.

## Examples

Assume that an options structure has been created using the spoptimset command:

```
options = spoptimset('TolFun', 1e-4, 'MaxIter', 200)
```

```
options = 
           Minimize: []
           Maximize: []
             TolFun: 1.0000e-04
               TolX: []
            MaxIter: 200
         StartPoint: []
        TestCorners: []
         PrevResult: []
             Method: []
          NumStarts: []
    OptimsetOptions: []
            Display: []
```

Using spoptimget, we can extract the contents of the structure:

```
tolFun = spoptimget(options, 'TolFun')
```

```
tolFun =
   1.0000e-04
```

By using the third argument, we can set a default in case the according property of the structure is empty:

```
startPoint = spoptimget(options, 'StartPoint')
startPoint = spoptimget(options, 'StartPoint', 'best')
```

```
startPoint =
     []
startPoint =
best
```

Note that the default argument has no effect if the accessed property contains a value.

```
maxIter = spoptimget(options, 'MaxIter', 500)
```

```
maxIter =
   200
```

## See Also

`spoptimset`.
