# Supplementary material for: Multiple Model-Informed Open-Loop Control of Uncertain Intracellular Signaling Dynamics
Source: PLoS Comput Biol. 2014 Apr 10;10(4):e1003546. doi: 10.1371/journal.pcbi.1003546 (PMC3983080; doi:10.1371/journal.pcbi.1003546)
Supplement: Dataset S1 — Matlab code for proposed control algorithm and prediction models. Contains all Matlab code necessary to implement the proposed adaptive weighted multiple-model predictive control algorithm, as well as code for the prediction models. (ZIP) [file pcbi.1003546.s001.zip › AW_MMPC/spinterp_v5.1.1/help/spoptimset.html]

spoptimset :: (Sparse Grid Interpolation Toolbox)


|  |  |
| --- | --- |
| **Sparse Grid Interpolation Toolbox** |  |

# spoptimset

Create/alter a sparse grid optimization `OPTIONS` structure.

## Syntax

`spoptimset`  
`OPTIONS = spoptimset('NAME1',VALUE1,'NAME2',VALUE2,...)`  
`OPTIONS = spoptimset(OLDOPTS,'NAME1',VALUE1,...)`  
`OPTIONS = spoptimset(OLDOPTS,NEWOPTS)`

## Description

`spoptimset` with no input arguments displays all property names and their possible values.

`OPTIONS = spoptimset('NAME1',VALUE1,'NAME2',VALUE2,...)` creates an options structure `OPTIONS` in which the named properties have the specified values. Any unspecified properties have default values. It is sufficient to type only the leading characters that uniquely identify the property. Case is ignored for property names.

`OPTIONS = spoptimset(OLDOPTS,'NAME1',VALUE1,...)` alters an existing options structure `OLDOPTS`.

`OPTIONS = spoptimset(OLDOPTS,NEWOPTS)` combines an existing options structure `OLDOPTS` with a new options structure `NEWOPTS`. Any new properties overwrite corresponding old properties.

## Properties

| **Property** | **Value {default}** | **Description** |
| --- | --- | --- |
| `Minimize` | {on} \| off | If set to `on`, the optimization algorithm will search for a minimizer. |
| `Maximize` | on \| {off} | If set to `on`, the optimization algorithm will search for a maximizer (searching for the minimizer and the maximizer at the same time is allowed). Note that searching for a maximizer is currently not supported by `spcgsearch`. |
| `TolX` | positive scalar {1e-4} | Termination tolerance on `X`. Note that the tolerance on `X` is taken with respect to the problem being re-scaled to the unit interval in each coordinate direction. That is, for instance, a sparse grid interpolant defined for the box `[0,1e6]x[0,1e-6]` with `TolX = 0.1` would mean a break tolerance of `1e5` in `x1` and a tolerance of `1e-7` in `x2`-direction. This parameter does not apply to `spcgsearch`. |
| `TolFun` | positive scalar {1e-6} | The search is terminated when the change of the function value from one iteration to the next is smaller than `TolFun`. |
| `MaxIter` | integer {100} | Maximum number of allowed iterations. |
| `StartPoint` | {best} \| random \| `Dx1` vector | Start search from best available, random, or specified start point. |
| `TestCorners` | on \| {off} | Specifically includes the `2^D` corner points of the search box as potential start points of the search. |
| `PrevResult` | `(d+1)x{1\|2}` double array | Specifies a possible best start point, such as from a previous search over a subdomain of the current search box. Format: `[xoptmin;ymin xoptmax;ymax]`, where `xoptmin` and xoptmax are column vectors. Depending on the contents of the `Minimize` and `Maximize` fields, minima and/or maxima information should be provided. `PrevResult` is only considered as a start point if `StartPoint` is set to `best`. |
| `Method` | {spcgsearch} \| {spcompsearch} \| spfminsearch | Specifies the method used by the multiple random start search `spmultistart`. `spcgsearch` is the default for the Chebyshev grid, otherwise, it is `spcompsearch`. |
| `NumStarts` | integer {10} | Number of local searches to perform for the multiple random start method `spmultistart`. The following points are considered: (best) + (`NumStarts-1` random points). |
| `OptimsetOptions` | struct {[]} | This feature is useful if additional configuration of the `fminsearch` algorithm used by `spfminsearch` is required beyond the parameters available through `spoptimset`.  Example: `opions = spoptimset('Optimset', optimset('FunValCheck','on'));` |
| `Display` | {off} \| iter | Optionally, displays information at each iteration. |

## Examples

As a preliminary to the following example, we construct a sparse grid interpolant of a test function (Branin's function) as
follows.

```
f = inline(['(5/pi*x-5.1/(4*pi^2)*x.^2+y-6).^2 +' ...
            '10*(1-1/(8*pi))*cos(x)+10']);
range = [-5 10; 0 15];
options = spset('keepFunctionValues','on', 'GridType', 'Chebyshev', ...
  'DimensionAdaptive', 'on', 'DimAdaptDegree', 1, 'MinPoints', 10, ...
        'RelTol', 1e-6);
z = spvals(f, 2, range, options);
```

A typical case of a modification of the sparse grid optimization options structure is given by the need to specify a more
stringent error tolerance on the function value to be used by the spcgsearch algorithm.

```
optoptions = spoptimset('TolFun', 1e-10);
format long e;
[xopt, fval] = spcgsearch(z, [], optoptions)
format short e;
```

```
xopt =
     3.141592655097273e+00
     2.274999997132531e+00
fval =
     3.978873577297303e-01
```

## See Also

`spoptimget`.
