# Supplementary material for: Multiple Model-Informed Open-Loop Control of Uncertain Intracellular Signaling Dynamics
Source: PLoS Comput Biol. 2014 Apr 10;10(4):e1003546. doi: 10.1371/journal.pcbi.1003546 (PMC3983080; doi:10.1371/journal.pcbi.1003546)
Supplement: Dataset S1 — Matlab code for proposed control algorithm and prediction models. Contains all Matlab code necessary to implement the proposed adaptive weighted multiple-model predictive control algorithm, as well as code for the prediction models. (ZIP) [file pcbi.1003546.s001.zip › AW_MMPC/spinterp_v5.1.1/help/sppurge.html]

sppurge :: (Sparse Grid Interpolation Toolbox)


|  |  |
| --- | --- |
| **Sparse Grid Interpolation Toolbox** |  |

# sppurge

Purge sparse grid data.

## Syntax

`Z = sppurge(Z)`  
`Z = sppurge(Z,OPTIONS)`  

## Description

`Z = sppurge(Z)` Marks indices that have corresponding hierarchical surplus values larger than the default drop tolerance `[0, 100*eps]`. The `sppurge` function returns the same sparse grid interpolant data `Z`, but enhanced by a field `purgeData` that is used by `spinterp` to only consider the marked indices in the interpolation process, thus saving computing time.

`Z = sppurge(Z,OPTIONS)` The parameter `OPTIONS` must be an options structure generated with `spset`. Only the value of the `DropTol` property is used, which enables the user to set any absolute and relative drop tolerance to be used by the purging algorithm.

## Examples

We consider the quadratic test function:

Implemented in Matlab by the following code:

```
type('trid.m')
```

```
function y = trid(x)
% TRID   Quadratic function with a tridiagonal Hessian.
%   Y = TRID(X)   returns the function value Y for a D-
%   dimenional input vector X.
%
% The test function is due to Arnold Neumaier, listed
% on the global optimization Web page at 
%   http://www.mat.univie.ac.at/~neum/glopt/

d = length(x);
y = sum((x-1).^2) - sum(x(2:d).*x(1:d-1));
```

During the construction of the interpolant, many subgrids are encountered that do no contribute to the interpolant, i.e.,
they have hierarchical surpluses that are all zero (up to floating point accuracy). An adaptive algorithm cannot know these
non-contributing subgrids in advance. However, using the DropTol feature, we can tell the interpolation function spinterp to neglect the subgrids that do not contribute, and thus, save a significant amount of computing time.

We consider the high-dimensional case d=100. With the dimension-adaptive algorithm, the problem structure is automatically detected, and the function is successfully
recovered using just O(d^2) function evaluations. For the interpolation domain, we use [-d^2, d^2] in each dimension.

```
d = 100;
range = repmat([-d^2 d^2],d,1);
options = spset('DimensionAdaptive', 'on', ...
                'DimadaptDegree', 1, ...
                'FunctionArgType', 'vector', ...
                'RelTol', 1e-3, ...
                'MaxPoints', 40000);
z = spvals('trid',d,range,options);
```

We now evaluate the obtained interpolant, first without, and thereafter, with the DropTol feature set to the default value
of `[0, 100*eps]` (absolute drop tolerance is zero, relative drop tolerance is `100*eps`). We evaluate the interpolant at 100
random points, measure the time, the absolute error, and compare the timing results in a plot.

```
% Compute 100 randomly sampled points
p = 100;
rand('state', 0);
x = -d^2 + 2*d^2*rand(p,d);

% Compute exact function values
y = zeros(p,1);
for k = 1:p
  y(k) = trid(x(k,:));
end

xcell = num2cell(x,1);
tic;
% Compute interpolated function values, no dropped indices
ip1 = spinterp(z, xcell{:});
t1 = toc

% Perform purging of interpolant data
tic;
z = sppurge(z);
t2 = toc
tic;
% Compute interpolated function values
% Some indices dropped according to drop tolerance
ip2 = spinterp(z, xcell{:});
t3 = toc

% Compute relative errors
err_ndt = max(abs(y-ip1))/(z.fevalRange(2)-z.fevalRange(1))
err_wdt = max(abs(y-ip2))/(z.fevalRange(2)-z.fevalRange(1))
```

```
t1 =
    2.1393

t2 =
    0.0128

t3 =
    0.2933

err_ndt =
    0.0061

err_wdt =
    0.0061
```

The result is quite impressing: Without loosing accuracy (which is no surprise considering the very low drop tolerance of
100\*eps compared to the relative error tolerance 1e-3), for the 100 sampled points, a speedup by a factor of about 7 is achieved
(including the cost of the sppurge function).

```
bar([NaN t1; t2 t3],'stacked');
legend('spurge', 'spinterp');
set(gca,'XTickLabel',{'without','with purging'});
ylabel('time [s]');
```

## See Also

`spset`.
