# Supplementary material for: Multiple Model-Informed Open-Loop Control of Uncertain Intracellular Signaling Dynamics
Source: PLoS Comput Biol. 2014 Apr 10;10(4):e1003546. doi: 10.1371/journal.pcbi.1003546 (PMC3983080; doi:10.1371/journal.pcbi.1003546)
Supplement: Dataset S1 — Matlab code for proposed control algorithm and prediction models. Contains all Matlab code necessary to implement the proposed adaptive weighted multiple-model predictive control algorithm, as well as code for the prediction models. (ZIP) [file pcbi.1003546.s001.zip › AW_MMPC/spinterp_v5.1.1/help/spquad.html]

spquad :: (Sparse Grid Interpolation Toolbox)


|  |  |
| --- | --- |
| **Sparse Grid Interpolation Toolbox** |  |

# spquad

Compute integral value of sparse grid interpolant.

## Syntax

`Q = spquad(Z)`  

## Description

`Q = spquad(Z)` Computes the integral over the sparse grid domain. The sparse grid data must be given as a structure `Z` containing the hierarchical surpluses (computed with with `spvals`).

The following additional option is available with `spquad` that is set by adding a field to the structure `Z`:

- `selectOutput [ integer {1} ]` Set the output variable number if an interpolant with multiple output variables was constructed with `spvals`. This determines which ouput variable the integral is computed for.

## Examples

Assume a sparse grid interpolant of the Matlab peaks function has been computed for the domain [0,2]^2 using the following commands:

```
options = spset('DimensionAdaptive', 'on', 'RelTol', 1e-4, ...
  'GridType', 'Chebyshev');
z = spvals(@(x,y) peaks(x,y), 2, [0,2; 0,2], options);
```

Then, we can compute the integral for this domain simply like this:

```
spquad(z)
```

```
ans =
    9.9553
```

For comparison, let us compute the integral value with Matlab's dblquad:

```
dblquad(@(x,y) peaks(x,y), 0, 2, 0, 2)
```

```
ans =
    9.9553
```

## See Also

`spvals`.
