# Supplementary material for: Multiple Model-Informed Open-Loop Control of Uncertain Intracellular Signaling Dynamics
Source: PLoS Comput Biol. 2014 Apr 10;10(4):e1003546. doi: 10.1371/journal.pcbi.1003546 (PMC3983080; doi:10.1371/journal.pcbi.1003546)
Supplement: Dataset S1 — Matlab code for proposed control algorithm and prediction models. Contains all Matlab code necessary to implement the proposed adaptive weighted multiple-model predictive control algorithm, as well as code for the prediction models. (ZIP) [file pcbi.1003546.s001.zip › AW_MMPC/spinterp_v5.1.1/help/spset.html]

spset :: (Sparse Grid Interpolation Toolbox)


|  |  |
| --- | --- |
| **Sparse Grid Interpolation Toolbox** |  |

# spset

Create/alter a sparse grid interpolation `OPTIONS` structure.

## Syntax

`spset`  
`OPTIONS = spset('NAME1',VALUE1,'NAME2',VALUE2,...)`  
`OPTIONS = spset(OLDOPTS,'NAME1',VALUE1,...)`  
`OPTIONS = spset(OLDOPTS,NEWOPTS)`

## Description

`spset` with no input arguments displays all property names and their possible values.

`OPTIONS = spset('NAME1',VALUE1,'NAME2',VALUE2,...)` creates an options structure `OPTIONS` in which the named properties have the specified values. Any unspecified properties have default values. It is sufficient to type only the leading characters that uniquely identify the property. Case is ignored for property names.

`OPTIONS = spset(OLDOPTS,'NAME1',VALUE1,...)` alters an existing options structure `OLDOPTS`.

`OPTIONS = spset(OLDOPTS,NEWOPTS)` combines an existing options structure `OLDOPTS` with a new options structure `NEWOPTS`. Any new properties overwrite corresponding old properties.

## Properties

| **Property** | **Value {default}** | **Description** |
| --- | --- | --- |
| `GridType` | {Clenshaw-Curtis} \| Maximum \| NoBoundary \| Chebyshev \| Gauss-Patterson | Sparse grid type and basis functions to use by `spvals`. For an illustration of the grid types, run `cmpgrids`. |
| `RelTol` | positive scalar {1e-2} | A relative error tolerance that applies to all hierarchical surpluses of the sparse grid representation. The grid is further refined until all hierarchical surpluses are less than max(`RelTol`\*(max(`fevalRange`)-min(`fevalRange`)),`AbsTol`), with `fevalRange` containing all results evaluating `FUN` up to that point. |
| `AbsTol` | positive scalar {1e-6} | Absolute error tolerance, used by the error criterion stated under the property `RelTol`. |
| `Vectorized` | on \| {off} | Indicates if `FUN` is available for vectorized evaluation. Vectorized coding of `FUN` can significantly reduce the computation time used by `spvals`. For an example using a vectorized function, please see `spdemo`. |
| `MinDepth` | integer {2} | Minimum interpolation depth, specifies the minimum number of hierarchical interpolation levels `N` to compute.  **Remark:** `MinDepth` has no effect if the dimension-adaptive grid refinement is switched on. An example is provided below. |
| `MaxDepth` | integer {8} | Maximum interpolation depth, specifies the maximum number of hierarchical interpolation levels `N` to compute.  **Remark:** Since version 5.0, `MaxDepth` also applies to the dimension-adaptive algorithm. If `MaxDepth` is reached with respect to a coordinate direction, this direction is no longer refined further. |
| `VariablePositions` | `1xD` vector {[]} | Position of the ranges in the argument list when `FUN` is evaluated. By setting `VariablePositions`, `spvals` will evaluate `FUN` with respect to some of its input parameters, but not necessarily the first `D` ones. The actual position is assigned by providing the number in the input argument list of the function `FUN`. This number must be provided for each interpolation dimension. Therefore, the value of `VariablePositions` must be a `1xD` array. An example is provided below. |
| `NumberOfOutputs` | integer {1} | If `FUN` produces multiple outputs (where all must be scalar!), indicate this here to perform the sparse grid computation for many output variables at once. Also see the example `spdemovarout`. |
| `PrevResults` | struct {[]} | Previous sparse grid data. An existing result structure obtained from `spvals` may be provided to further refine an existing sparse grid. An example is provided below. |
| `FunctionArgType` | {list} \| vector | Indicates whether the objective function takes the input parameters as a comma-separated list (default) or as a vector. |
| `KeepFunctionValues` | {off} \| on | If this parameter is set, a structure field `fvals` is returned, containing a cell array with the function values at the sparse grid points. |
| `KeepGrid` | {off} \| on | If this parameter is set, a structure field `grid` is returned, containing a cell array with the the sparse grid points. |
| `DimensionAdaptive` | {off} \| on | Dimension-adaptive grids try to adaptively find important dimensions and adjust the sparse grid structure accordingly. Especially in case of higher-dimensional problems, a dimension-adaptive strategy can significantly reduce the number of support nodes required to achieve a good interpolant. |
| `DimadaptDegree` | positive scalar {0.9} | Fine-tuning parameter to alter the degree of dimensional adaptivity. A value of 1 places strong emphasis on the error estimates, and thus leads to strong dimensional adaptivity. A value of 0 disregards the error estimates, and constructs a conventional sparse grid based on the amount of work involved. |
| `DegreeStrategy` | {balancing} \| depth | Strategy for the degree of dimensional adaptivity.  The **balancing** strategy balances the number of grid points generated according to the greedy, error estimate-based refine ment rule compared to the number of points generated by the conventional (regular) sparse grid refinement rule. I.e., a `DimadaptDegree` value of 0.9 would mean that around 90% of the grid points are generated by the error estimate-based rule, and the remaining points are selected according to the regular rule. See Advanced Topics, Degree of Dimensional Adaptivity for additional information.  The **depth** strategy makes sure that the maximum level depth reached by the error estimate-based refinement in one dimension does not get too deep compared to the depth reached in the other dimensions. This strategy is the one used prior to version 5.1 of the toolbox, and is described in [3, ch. 3]. This approach is still supported but not the default strategy. |
| `MinPoints` | integer {100} | This parameter only applies to dimension-adaptive sparse grids, and indicates the minimum number of support nodes (i.e. function evaluations to perform). An example is provided below. |
| `MaxPoints` | integer {10000} | This parameter only applies to dimension-adaptive sparse grids. The dimension-adaptive algorithm is aborted once the function evaluation count exceeds this number. |
| `SparseIndices` | {auto} \| off \| on | Manually turn the efficient sparse storage scheme (new feature since version 3.0) of the multi-index arrays on or off. The default switch `auto` uses the new scheme for the `ClenshawCurtis`, the `Chebyshev`, and the `Gauss-Patterson` grid, and the old (full) storage scheme from version 2.x for the `Maximum` and the `NoBoundary` grid (the sparse grid storage scheme is not supported for these two grid types). |
| `DropTol` | `1x2` vector \| {auto} \| off | During the sparse grid construction progress, the `spvals` algorithm may add subgrids with hierarchical surpluses that are all close to zero or of negligible magnitude compared to the surpluses of other sub-grids. In particular, this occurs when additive structure is present in the objective function. To increase the performance of the `spinterp` algorithm, you may run the `sppurge` algorithm that marks subgrids to be neglected where all hierarchical surpluses `w` are less than max(relDropTol\*(max(fevalRange)-min(fevalRange),absDropTol)). You may specify the absolute and the relative drop tolerance as a vector `[absDropTol, relDropTol]`, or turn it `off` completely (= behavior of version 3.0 and earlier). The switch `auto` uses the values `absDropTol = 0`, `relDropTol = 100*eps`, that is, by default, only a relative drop tolerance is used. |
| `EnableDCT` | {on} \| off | Enables/disables the DCT-based algorithm when constructing the Chebyshev-Gauss-Lobatto type sparse grid. |

## Examples

Since spset offers many possibilities to alter the behavior of the sparse grid interpolant construction, we provide several typical examples
in the following.

- Example 1: Basic usage of spset
- Example 2: Providing previous results
- Example 3: Using the VariablePositions property

### Example 1: Basic usage of spset

As an example for a typical task requiring the modification of the sparse grid options structure. we construct an interpolant
with a specified number of function evaluations. The dimension-adaptive approach permits to do this in an elegant manner.
The following code constructs an interpolant with about 100 nodes, since both MinPoints as well as MaxPoints are set to 100.

```
f = @(x,y) exp(x+y);
z = spvals(f, 2, [], spset('DimensionAdaptive', 'on', ...
                     'MinPoints', 100, 'MaxPoints', 100))
```

```
z = 
               vals: {[129x1 double]}
           gridType: 'Clenshaw-Curtis'
                  d: 2
              range: []
        estRelError: 8.3520e-04
        estAbsError: 0.0053
         fevalRange: [1 7.3891]
         minGridVal: [0 0]
         maxGridVal: [1 1]
            nPoints: 129
          fevalTime: 0.0546
    surplusCompTime: 0.0058
            indices: [1x1 struct]
           maxLevel: [5 5]
      activeIndices: [5x1 uint32]
     activeIndices2: [13x1 uint32]
                  E: [1x19 double]
                  G: [19x1 double]
                 G2: [19x1 double]
       maxSetPoints: 5
           dimAdapt: 1
```

Note that the MinPoints and MaxPoints properties only work for dimension-adaptive grids. If we want to construct a non-adaptive grid of a certain depth, the MinDepth and MaxDepth options can be used. Recall that the number of points of a regular sparse grid can be determined a priori with the spdim function.

```
n = 4; d=2;
spdim(4,2);
z = spvals(f, 2, [], spset('MinDepth', n, 'MaxDepth', n))
```

```
z = 
               vals: {[65x1 double]}
           gridType: 'Clenshaw-Curtis'
                  d: 2
              range: []
           maxLevel: 4
        estRelError: 0.0031
        estAbsError: 0.0201
         fevalRange: [1 7.3891]
         minGridVal: [0 0]
         maxGridVal: [1 1]
            nPoints: 65
          fevalTime: 0.1066
    surplusCompTime: 0.0029
            indices: [1x1 struct]
```

### Example 2: Providing previous results

After computing an interpolant with a certain accuracy, it is often required to improve it further later on. Due to the hierarchical
construction scheme, the previous results are not lost but can be passed to spvals for further refinement, as the following code illustrates.

```
f = @(x,y) exp(x+y);
z = [];
for n = 1:4
  z = spvals(f, 2, [], spset('MinDepth', n, 'MaxDepth', n, ...
                             'PrevResults', z));
  disp(['n = ' num2str(z.maxLevel) ', estimated rel. error: ', ...
        num2str(z.estRelError)]);
end
```

```
Warning: MaxDepth = 1 reached before accuracies
    RelTol = 0.01 or AbsTol = 1e-06 were achieved.
The current estimated relative accuracy is 0.62246.
n = 1, estimated rel. error: 0.62246
Warning: MaxDepth = 2 reached before accuracies 
   RelTol = 0.01 or AbsTol = 1e-06 were achieved.
The current estimated relative accuracy is 0.17905.
n = 2, estimated rel. error: 0.17905
Warning: MaxDepth = 3 reached before accuracies
   RelTol = 0.01 or AbsTol = 1e-06 were achieved.
The current estimated relative accuracy is 0.011133.
n = 3, estimated rel. error: 0.011133
n = 4, estimated rel. error: 0.0031415
```

### Example 3: Using the VariablePositions property

Consider the case of a function of four parameters, e.g.

Suppose that the parameters a and b are fixed to a = 0.5 and b = 0.2, and we wish to compute an approximation of f for x, y in [0,1]^2. The default syntax of spvals would require the interpolated parameters to appear at the start of the argument list, i.e. would require an argument list
(x,y,a,b) to enable the call spvals(f,2,[],[],a,b).

By using VariablePositions, we can use the function as it is defined above, as the following code shows.

```
f = inline('a.*(x.^2+y.^2) + b.*exp(x+y)','a','b','x','y')
a = 0.5; b = 0.2;
options = spset('VariablePositions', [3 4]);
z = spvals(f,2,[],options,a,b)
```

```
f =
     Inline function:
     f(a,b,x,y) = a.*(x.^2+y.^2) + b.*exp(x+y)
z = 
               vals: {[29x1 double]}
           gridType: 'Clenshaw-Curtis'
                  d: 2
              range: []
           maxLevel: 3
        estRelError: 0.0062
        estAbsError: 0.0142
         fevalRange: [0.2000 2.4778]
         minGridVal: [0 0]
         maxGridVal: [1 1]
            nPoints: 29
          fevalTime: 0.0508
    surplusCompTime: 0.0015
            indices: [1x1 struct]
```

Since the interpolation problem is two-dimensional, we assign a 1x2 vector to the VariablePositions property, indicating that the first parameter an interpolation of which is required is located at position 3 of the argument
list of f, and the second one at position 4. Note that since the function f takes four input paramters, the remaining parameters are appended to the argument list of spvals after the options argument.

## See Also

`spget`,
`spvals`.
