# Supplementary material for: Multiple Model-Informed Open-Loop Control of Uncertain Intracellular Signaling Dynamics
Source: PLoS Comput Biol. 2014 Apr 10;10(4):e1003546. doi: 10.1371/journal.pcbi.1003546 (PMC3983080; doi:10.1371/journal.pcbi.1003546)
Supplement: Dataset S1 — Matlab code for proposed control algorithm and prediction models. Contains all Matlab code necessary to implement the proposed adaptive weighted multiple-model predictive control algorithm, as well as code for the prediction models. (ZIP) [file pcbi.1003546.s001.zip › AW_MMPC/spinterp_v5.1.1/help/spsurfun.html]

spsurfun :: (Sparse Grid Interpolation Toolbox)


|  |  |
| --- | --- |
| **Sparse Grid Interpolation Toolbox** |  |

# spsurfun

Evaluate the sparse grid interpolant at a single point.

`spsurfun` is provided for conveniece to be used as an alternative to `spinterp`, where the point `Y` to be evaluated is given as a row or column vector. This functional form is often adopted by multivariate optimization algorithms in Matlab.

Note that this form allows the evaluation of the sparse grid interpolant at a single point only. Therefore, It is recommended to use `spinterp` instead if multiple evaluations of the interpolant can be performed simultaneously.

## Syntax

`IP = spsurfun(Y,Z)`  
`[IP,IPGRAD] = spsurfun(Y,Z)`  

## Description

`IP = spsurfun(Y,Z)` Computes the interpolated value IP at the single point `[Y1, ..., YD]` for the sparse grid interpolant `Z`.

`[IP,IPGRAD] = spsurfun(...)` Computes the interpolated value `IP` and the gradient vector `IPGRAD`.

Two additional options are available with `spsurfun` that are set by adding a field to the structure `Z`:

- `selectOutput [ integer {1} ]` Set the output variable number if an interpolant with multiple output variables was constructed with `spvals`.- `continuousDerivatives [ 'on' | {'off'} ]` Enable augmented continuous derivatives for the Clenshaw-Curtis grid.

## Examples

The following code shows how to use spsurfun. Note that as opposed to the spinterp syntax, the **second** argument is the sparse grid interpolant, not the first one.

```
f = inline('x.^2 + y.^2 - 2.*z');
z = spvals(f,3,[],spset('GridType','Chebyshev'));
[ip,ipgrad] = spinterp(z, 0.5, 0.2, 0.2)
[ip,ipgrad] = spsurfun([0.5, 0.2, 0.2], z)
```

```
in =
   -0.1100
ipgrad = 
    [3x1 double]
ip =
   -0.1100
ipgrad =
    1.0000
    0.4000
   -2.0000
```

See the help page on sparse grid optimization for an example where spsurfun is used with an optimization method from Mathwork's Optimization Toolbox.

## See Also

`spinterp`.
