# Supplementary material for: Multiple Model-Informed Open-Loop Control of Uncertain Intracellular Signaling Dynamics
Source: PLoS Comput Biol. 2014 Apr 10;10(4):e1003546. doi: 10.1371/journal.pcbi.1003546 (PMC3983080; doi:10.1371/journal.pcbi.1003546)
Supplement: Dataset S1 — Matlab code for proposed control algorithm and prediction models. Contains all Matlab code necessary to implement the proposed adaptive weighted multiple-model predictive control algorithm, as well as code for the prediction models. (ZIP) [file pcbi.1003546.s001.zip › AW_MMPC/spinterp_v5.1.1/help/spvals.html]

spvals :: (Sparse Grid Interpolation Toolbox)


|  |  |
| --- | --- |
| **Sparse Grid Interpolation Toolbox** |  |

# spvals

Construct a sparse grid interpolant.

## Syntax

`Z = spvals(FUN,D)`  
`Z = spvals(FUN,D,RANGE)`  
`Z = spvals(FUN,D,RANGE,OPTIONS)`  
`Z = spvals(FUN,D,RANGE,OPTIONS,P1,P2, ...)`

## Description

`Z = spvals(FUN,D)` Compute the sparse grid representation `Z` for multi-linear sparse grid interpolation of the function `FUN`. The grid is computed over the d-dimensional unit cube `[0,1]^D`.

`Z = spvals(FUN,D,RANGE)` In addition to the syntax above, the
interpolation box dimensions may be specified. `RANGE` is a `2xD` array, e.g. to compute the sparse grid representation over the domain `[0,1]x[2,4]x[1,5]` of `FUN`, `RANGE` must be `[0 1; 2 4; 1 5]`. If `RANGE` is empty (=`[]`), it is assumed to be `[0,1]^D`.

`Z = spvals(FUN,D,RANGE,OPTIONS)` Computes the sparse grid representation as above, but with default interpolation properties replaced by values in `OPTIONS`, an argument created with `spset`.

`Z = spvals(FUN,D,RANGE,OPTIONS,P1,P2, ...)` passes the parameters `P1,P2,...` to the objective function `FUN`.

## Examples

The following examples demonstrate the generation of sparse grid interpolants under a variety of different parameters. The
extensive configurability of spvals is achieved via the spset function. Additional examples of constructing interpolants of
external functions, models with several output parameters, ODEs, etc. are provided in the advanced topics section.

We first define the test function. In the examples below, we use Branin's test function:

We set the dimension to d = 2, and the interpolation domain to range = [-5,10; 0,15].

```
fun = inline(['(5/pi*x-5.1/(4*pi^2)*x.^2+y-6).^2 + ' ...
              '10*(1-1/(8*pi))*cos(x)+10']);
d = 2;
range = [-5, 10; 0, 15];
```

Now, we compute a regular (i.e. non-adaptive) sparse grid interpolant of fun using the default settings of the Sparse Grid Interpolation toolbox. This will compute a piecewise linear interpolant at
the Clenshaw-Curtis sparse grid.

```
z1 = spvals(fun, d, range)
```

```
z1 = 
               vals: {[145x1 double]}
           gridType: 'Clenshaw-Curtis'
                  d: 2
              range: [2x2 double]
           maxLevel: 5
        estRelError: 0.0087
        estAbsError: 2.6622
         fevalRange: [1.3697 308.1291]
         minGridVal: [0.1250 0.7500]
         maxGridVal: [0 0]
            nPoints: 145
          fevalTime: 0.2437
    surplusCompTime: 0.0040
            indices: [1x1 struct]
```

For comparison, we now compute two additional interpolants, one being a regular Chebyshev-Gauss-Lobatto grid, the other one
being a dimension-adaptive sparse grid interpolant of the same grid type. To do this, we must pass an according options structure
to the spvals routine. We do not have to store this options structure- it is possible to pass a structure generated on-the-fly to the function.

```
z2 = spvals(fun, d, range, spset('GridType', 'Chebyshev'))
z3 = spvals(fun, d, range, spset('GridType', 'Chebyshev', ...
                                 'DimensionAdaptive', 'on', ...
                                 'DimAdaptDegree', 1, ...
                                 'MinPoints', 20))
```

```
z2 = 
               vals: {[65x1 double]}
           gridType: 'Chebyshev'
                  d: 2
              range: [2x2 double]
           maxLevel: 4
        estRelError: 0.0095
        estAbsError: 2.9017
         fevalRange: [2.5620 308.1291]
         minGridVal: [0.5000 0.2222]
         maxGridVal: [0 0]
            nPoints: 65
          fevalTime: 0.1211
    surplusCompTime: 0.0225
            indices: [1x1 struct]

z3 = 
               vals: {[29x1 double]}
           gridType: 'Chebyshev'
                  d: 2
              range: [2x2 double]
        estRelError: 0.0095
        estAbsError: 2.9017
         fevalRange: [2.7065 308.1291]
         minGridVal: [0.5000 0.1464]
         maxGridVal: [0 0]
            nPoints: 29
          fevalTime: 0.0468
    surplusCompTime: 0.0094
            indices: [1x1 struct]
           maxLevel: [4 2]
      activeIndices: [3x1 uint32]
     activeIndices2: [9x1 uint32]
                  E: [1x9 double]
                  G: [9x1 double]
                 G2: [9x1 double]
       maxSetPoints: 4
           dimAdapt: 1
```

The following code generates a plot comparing the three interpolants. Furthermore, the error is plotted compared to the original
function.

```
z = {z1, z2, z3};
for k = 1:3
  f_z = @(x,y) spinterp(z{k}, x, y);
  error_z = @(x,y) fun(x,y) - spinterp(z{k}, x, y);
  subplot(2,3,k);
  ezmesh(f_z, [range(1,:),range(2,:)]);
  title(['interpolant z_' num2str(k)]);
  view(20,30);
  subplot(2,3,k+3);
  ezmesh(error_z, [range(1,:),range(2,:)]);
  title(['error of z_' num2str(k)]);
  view(20,30);
end
```

## See Also

`spinterp`,
`spset`.
