# Supplementary material for: Multiple Model-Informed Open-Loop Control of Uncertain Intracellular Signaling Dynamics
Source: PLoS Comput Biol. 2014 Apr 10;10(4):e1003546. doi: 10.1371/journal.pcbi.1003546 (PMC3983080; doi:10.1371/journal.pcbi.1003546)
Supplement: Dataset S1 — Matlab code for proposed control algorithm and prediction models. Contains all Matlab code necessary to implement the proposed adaptive weighted multiple-model predictive control algorithm, as well as code for the prediction models. (ZIP) [file pcbi.1003546.s001.zip › AW_MMPC/spinterp_v5.1.1/help/whatis.html]

What is the Sparse Grid Interpolation Toolbox? (Sparse Grid Interpolation Toolbox)


|  |  |
| --- | --- |
| **Sparse Grid Interpolation Toolbox** |  |

# What is the Sparse Grid Interpolation Toolbox?

## Introduction

The interpolation problem considered with sparse grid interpolation is an optimal recovery problem (i.e. the selection of points such that a smooth multivariate function can be approximated with a suitable interpolation formula). Depending on the characteristics of the function to interpolate (degree of smoothness, periodicity), various interpolation techniques based on sparse grids exist. All of them employ Smolyak's construction, which forms the basis of all sparse grid methods. With Smolyak's famous method, well-known univariate interpolation formulas are extended to the multivariate case by using tensor products in a special way. As a result, one obtains a powerful interpolation method that requires significantly fewer support nodes than conventional interpolation on a full grid. The points comprising the multidimensional sparse grid are selected in a predefined fashion. The difference in the number of required points can be several orders of magnitude with increasing problem dimension. The most important property of the method constitutes the fact that the asymptotic error decay of full grid interpolation with increasing grid resolution is preserved up to a logarithmic factor. An additional benefit of the method is its hierarchical structure, which can be used to obtain an estimate of the current approximation error. Thus, one can easily develop an interpolation algorithm that aborts automatically when a desired accuracy is reached.

## Major Features

This Matlab toolbox includes hierarchical sparse grid interpolation algorithms based on both piecewise multilinear and polynomial basis functions. Special emphasis is placed on an efficient implementation that performs well even for very large dimensions `d > 10`.

There are many ways to customize the behavior of the interpolation routines. Furthermore, additional tasks involving the interpolants can be performed, such as computing derivatives or performing an optimization or integration. The following list gives an overview of the options that are available:

- Enable vectorized processing. Speed up the construction of the interpolant for functions that allow for vectorized evaluation.- Create multiple interpolants at once for functions with multiple output arguments.- Choose from three different grid types for piecewise linear interpolation. Depending on your objective function, a certain grid type may perform better than the others.- If very high accuracies are required, you may use the Chebyshev-Gauss-Lobatto sparse grid, which employs polynomial basis functions.- Compute gradients along with the interpolated values at just a small additional cost.- Integrate the interpolant.- Perform a search for minima and maxima using several efficient algorithms.- Use the dimension-adaptive algorithm to automatically detect separability, and to take the importance of the dimensions into account when constructing the interpolant. This is especially useful in case of very high-dimensional problems when a regular sparse grid refinement leads to too many support nodes.- Specify the minimum or maximum sparse grid depth to compute, or specify the minimum and maximum number of function evaluations to use (for the dimension-adaptive sparse grid).- Last but not least, the Sparse Grid Interpolation toolbox is designed to easily integrate with your models in Matlab
                    as well as external models.

For additional information on the theoretical and algorithmic aspects of sparse grid interpolation, please refer to the references provided at the end of this chapter.

|  |  |  |  |  |
| --- | --- | --- | --- | --- |
|  | Sparse Grid Interpolation basics |  | Initialization of the toolbox |  |
